# Mapping and Predicting Non-Linear *Brassica rapa* Growth Phenotypes Based on Bayesian and Frequentist Complex Trait Estimation

**DOI:** 10.1534/g3.117.300350

**Published:** 2018-02-26

**Authors:** R. L Baker, W. F. Leong, S. Welch, C. Weinig

**Affiliations:** * Department of Biology, Miami University, Oxford, OH 45056,; † Department of Agronomy, Kansas State University, Manhattan, KS 66506,; ‡ Department of Molecular Biology and; § Department of Botany, University of Wyoming, Laramie, WY 82071

**Keywords:** Function-Valued Traits, quantitative genetics, *Brassica rapa*, leaf development, high-throughput phenotyping, phenotypic plasticity, Bayesian *vs.* frequentist, genotype to phenotype

## Abstract

Predicting phenotypes based on genotypes and understanding the effects of complex multi-locus traits on plant performance requires a description of the underlying developmental processes, growth trajectories, and their genomic architecture. Using data from *Brassica rapa* genotypes grown in multiple density settings and seasons, we applied a hierarchical Bayesian Function-Valued Trait (FVT) approach to fit logistic growth curves to leaf phenotypic data (length and width) and characterize leaf development. We found evidence of genetic variation in phenotypic plasticity of rate and duration of leaf growth to growing season. In contrast, the magnitude of the plastic response for maximum leaf size was relatively small, suggesting that growth dynamics *vs.* final leaf sizes have distinct patterns of environmental sensitivity. Consistent with patterns of phenotypic plasticity, several QTL-by-year interactions were significant for parameters describing leaf growth rates and durations but not leaf size. In comparison to frequentist approaches for estimating leaf FVT, Bayesian trait estimation resulted in more mapped QTL that tended to have greater average LOD scores and to explain a greater proportion of trait variance. We then constructed QTL-based predictive models for leaf growth rate and final size using data from one treatment (uncrowded plants in one growing season). Models successfully predicted non-linear developmental phenotypes for genotypes not used in model construction and, due to a lack of QTL-by-treatment interactions, predicted phenotypes across sites differing in plant density.

Expressed phenotypes reflect the independent and combined effects of genetic and environmental inputs over time. However, understanding the relationship between genotype, the environment, and phenotype can be complicated. For example, phenotypic plasticity generated via genotype-by-environment interactions can alter the course of development, allowing a single genotype to exhibit multiple distinct phenotypes (Bradshaw 1965; Schlichting and Pigliucci 1998; Diggle 2002). Alternatively, phenotypic traits may be buffered against environmental effects, a phenomenon referred to as canalization (Waddington 1954; Rice 1998; Debat and David 2001; Hall *et al.* 2007). Predicting phenotypes based on genotypes across multiple environments is therefore complicated by differential environmental sensitivity, yet is critical for understanding and predicting crop yields and evolutionary outcomes (Yang and Zhu 2005; van Eeuwijk *et al.* 2010; Ober *et al.* 2012; Crossa *et al.* 2013; Zhang *et al.* 2015).

Understanding the agronomic and evolutionary performance of complex traits requires a description of the developmental processes and growth trajectories that generate these phenotypes (Kell 2002; Onogi *et al.* 2016). An ontogenetic description is necessary, in part because selection does not act only on final phenotypes. Instead, selection acts continuously on phenotypes throughout organismal ontogeny as well as directly on growth rates themselves (Prusinkiewicz *et al.* 2007; Baker *et al.* 2015). Furthermore, in comparison to single time point measurements when growth has ceased, characterizing the processes that produce a final phenotype leads to a more complete description of the genomic architecture underlying a given trait (Baker *et al.* 2015). Analysis of developmental traits is, however, complicated by the fact that these processes are continuous while the data collected are typically discrete. Characterizing a developmental process necessitates a large number of measurements, which, among other statistical effects, reduces the power of significance testing under multivariate analyses (Griswold *et al.* 2008). Function-Valued Trait (FVT) estimation fits mathematical functions to measurements collected over time, and thereby reduces the number of independent traits. Under one variant of FVT modeling, parameters that characterize the function are estimated and then analyzed as phenotypic trait data (Jaffrézic and Pletcher 2000; Kingsolver *et al.* 2001; Stinchcombe and Kirkpatrick 2012; Baker *et al.* 2018). While there are advantages and limitations to each of several approaches, one advantage to the “parameters as traits” approach is that different parameters have ontogenetically interpretable features. For functions characterizing leaf growth, for example, one can distinguish between the slope (rate of growth), growth duration, and asymptote (final size), and examine the potentially unique genetic architectures, inter-relationships, and environmental sensitivities of these traits.

In additional to the external environment, developmental phenotypes and growth processes can be influenced by a plant’s internal physiological status (Hammer *et al.* 2005). For example, manipulating photosynthetic efficiency and carbon availability via transgenesis dramatically affects leaf morphology (Raines and Paul 2006; Schneidereit *et al.* 2006). Recent advances in FVT modeling use hierarchical Bayesian approaches to incorporate genotype-specific cofactors such as maximum photosynthetic capacity (*A_max_*) and thereby factor out genotypic variation in carbon pools available during development and growth (Baker *et al.* 2018). Residual genetic variation that remains after statistically factoring out *A_max_* should reflect factors that more directly affect leaf expansion independent of genotypic-specific carbon resources (Baker *et al.* 2018). Further, FVT modeling in a Bayesian framework leads to increased estimates of trait heritability compared to frequentist approaches, because the former takes into account the underlying structure of the data and provides for improved error handling (Baker *et al.* 2018). Whether Bayesian approaches to FVT estimation also afford concomitant improvements in QTL mapping remains an open question.

Quantitative-genetic analyses of both FVT and other complex traits require high genotypic replication. The challenge of phenotyping throughput of numerous genotypes can be addressed for some traits by remote sensing approaches (Weber *et al.* 2012). In *Brassica rapa*, spectroradiometric indices are genetically correlated with leaf-level physiological measurements and aspects of final leaf morphology, such that genotypes with high values of, for instance, the MERIS Terrestrial Chlorophyll Index (Dash and Curran 2004) have high values of *A_max_* and large leaf sizes (Baker *et al.* 2018). Whether QTL can be identified that jointly affect spectroradiometric indices as well as physiological traits or leaf FVT remains unexplored. Such QTL could allow high-throughput remote sensing to be used as a proxy for more time-consuming direct measurements of leaf gas-exchange and morphological development, and these QTL could be used in molecular plant breeding programs (Yin *et al.* 2000). Notably, simulation studies demonstrate that accounting for genotypic variation in gas-exchange can accelerate plant breeding (Hammer *et al.* 2005).

Data collected over ontogeny and in multiple environments may contribute to realistic models of population dynamics and of crop performance (Chenu *et al.* 2009). More generally, predicting phenotypes based on genotypes is a focal area of current genomics research, in part because it forms the basis of highly efficient genomic selection strategies for crop improvement (Jannink *et al.* 2010; Bustos-Korts *et al.* 2016). However, few studies have predicted ontogenetic growth trajectories based on genotypic information (for an exception, see Reymond *et al.* 2003). Here, we report on improved characterization of developmental and growth processes and their environmental dependencies, which are relevant for both evolutionary ecologists and agronomists. Specifically, we expand upon the data and analyses presented in Baker *et al.* (2015) by applying new Bayesian FVT estimation routines (developed in Baker *et al.* 2018) to existing leaf data, leaf phenotypic data collected in a separate season, and high-throughput spectroradiometric data. We combine ontogenetic and physiological data in hierarchical Bayesian models to estimate leaf FVT in the annual species, *Brassica rapa*. Using these FVT estimates, we test for phenotypic plasticity expressed in response to season and crowding and ask whether the magnitude of plasticity differs among FVT parameters (*e.g.*, growth rates *vs.* durations). We then map QTL to assess FVT genomic architecture including QTL-by-environment interactions that underlie plastic responses. We test if Bayesian FVT estimation approaches improve QTL identification compared to frequentist approaches. Finally, we test whether QTL-based (genotypic) models can predict complex, multi-locus, non-linear patterns of leaf developmental phenotypes in *B. rapa*.

## Materials And Methods

### Species description

*Brassica rapa* (Brassicaceae) is an annual to biennial herbaceous crop. We utilize Recombinant Inbred Lines (RILs) generated by crossing the R500 × IMB211 genotypes. R500 is a late-flowering yellow sarson oil seed with relatively large, broad leaves, while IMB211 is derived from a Wisconsin Fast Plant (WFP) and selected for rapid cycling, flowers early, and has relatively small, lanceolate leaves (Baker *et al.* 2015). All RILs are expected to be >99% homozygous (as described in Hinata and Prakash 1984; Brock and Weinig 2007; Iniguez-Luy *et al.* 2009). This experiment includes 119 RILs and genotypes representative of the R500 and IMB211 parents.

### Experimental Design and Data Collection

#### Plant growth:

To test for plastic responses to inter-annual microclimatic variation (year) and crowding, we grew plants during three growing seasons and in two density treatments. Plant growth and experimental design follow protocols described in Baker *et al.* (2015). Briefly, in 2010, 2011, and 2012, the IMB211 × R500 RILs were germinated in the greenhouse in pots filled with fertilized field soil, and then transplanted into the field at the University of Wyoming Agricultural Experiment Station. The crowded (CR) treatment consisted of 5 plants of the same genotype per 4” peat pot with the central plant designated as a focal individual on which measurements were collected. The uncrowded (UN) treatment consisted of a single plant per pot. Each block consisted of one replicate of each RIL and each parental genotype. Locations of replicates within each block were randomized, and each block was randomly assigned to a treatment (CR or UN). Plants were transplanted into the field with 25 cm between each focal plant. In 2010, 12 UN and 12 CR blocks were transplanted into the field. In 2011, 6 UN and 6 CR blocks were transplanted into the field and in 2012 8 CR and 8 UN blocks were transplanted. All plants were watered to field capacity, and pesticides were applied as needed. Temperature data from 2011 and 2012 were collected (File S1) and used to generate degree days (DD, hereafter) assuming genotypes share the same *B. rapa*-specific base value of 0.96° as described in Baker *et al.* (2015) and Vigil *et al.* (1997).

#### Physiological data:

Photosynthetic capacity (*A_max_*; μmol m^−2^ sec^−1^) was collected from UN and CR plants in 2010 as described in Edwards *et al.* (2012) genotypic means were calculated as described in and Baker *et al.* (2015) and below.

#### Spectroradiometric data:

Spectral reflectance measurements were collected in 2010 and 2011 as described in Baker *et al.* (2018). The spectroradiometer collected wavelengths from 350 to 2500 nm with a 1.4 nm sampling interval for the visible/NIR spectral region (350 nm – 1000 nm) and 2 nm for the short-wave infrared spectral region (1000 nm – 2500 nm) (An *et al.* 2015); when interpolated, there is one spectral data point per nanometer, for a total of 2151 spectral data points per measurement. For each replicate plant, 10 measurements were collected and averaged to produce a single measurement (An *et al.* 2015).

Raw spectroradiometric data were used to calculate common remote sensing vegetation indices. These include: mcari1 and mcari2 (Modified Chlorophyll Absorption Ratio Index), mtci (Transformed Chlorophyll Absorption Ratio Index) (Haboudane *et al.* 2004), sipi2 (Structure Insensitive Pigment Index) (Haboudane *et al.* 2002), tcari (Transformed Chlorophyll Absorption Ratio Index) (Peñuelas *et al.* 1995), ari1 and ari2 (Anthocyanin Reflectance Index) (Haboudane *et al.* 2002), cri (Carotenoid Reflectance Index) (Gitelson *et al.* 2001), npci (Normalized Pigment Chlorophyll Index)(Gitelson *et al.* 2002), pri2 (photochemical reflectance) (Peñuelas *et al.* 1994), psri (Plant Senescence Reflectance Index) (Garbulsky *et al.* 2011) and wi (water index) (Merzlyak *et al.* 1999). We binned raw spectroradiometric data and calculated the reflected red to far red (R:FR) ratio for each plant as (655nm-665nm)/(725nm-735nm) (Penuelas *et al.* 1997).

#### Morphological data:

Leaf lengths and widths (LL and LW, respectively) were recorded on the second epicotylar leaf for all plants in 2011 and 2012. Data collection started at leaf emergence and was conducted 2-3 times per week as described in Baker *et al.* (2015).

### Data analysis

#### Function-Valued Trait (FVT) Modeling:

FVT modeling for trait estimation used Bayesian approaches that fit logistic growth curves to longitudinal LL and LW data (Equations 1 & 2, respectively) as described in Baker *et al.* (2018). LW and LL for each individual replicate plant is represented by a minimum of 5 and maximum of 16 sequential measurements. Briefly, we utilized a three-level hierarchical Bayesian model that retains the measurement data structure to account for information across all plants and genetic lines within the population, (including replicate plants within each line) and a global mean parameter.

$$\mathrm{Equation 1}:\frac{d}{dt}LL=rLL\left(\frac{LL_{Lmax}-LL}{LL_{Lmax}}\right)$$

$$\mathrm{Equation 2}:\frac{d}{dt}LW=rLW\left(\frac{LW_{Lmax}-LW}{LW_{Lmax}}\right)$$

Leaf length and width were modeled independently across treatments and growing seasons. The Function-Valued Trait (FVT) parameters *Lmax* and *r* estimate the *L*eaf *max*imum size (in mm) and *r*ate of growth, (mm/DD) respectively. Two parameters estimated the duration of growth. The first, *d*, was calculated as the *duration* (in Degree Days, DD) of time between germination and 95% of leaf growth. The second, *iD*, was algebraically extracted from the growth curve and describes when the growth curves reached their *inflection* point in *Degree Days* and transitioned from exponentially accelerating to decelerating growth rates.

The hierarchical Bayesian model was implemented using the Bayesian Statistical Modeling Python module PyMC and the model parameters were estimated via MCMC using the Metropolis-Hastings algorithm (Chib and Greenberg 1995; Patil *et al.* 2010). The MCMC estimations were performed using a single chain to sample 500,000 iterations, which includes the first discarded 440,000 burn-in iterations; the remaining 60,000 iterations were retained. By thinning to 1 iteration in 20, the retained iterations were reduced to 3,000 samples for every FVT parameter from which the posterior distributions were tabulated. All parameters’ trace and auto-correlation plots were examined to ensure that the MCMC chain had adequate mixing and had reached convergence (Baker *et al.* 2018). All observed data for each genotype were plotted with two 95% credible interval envelopes. The inner, yellow envelope represents the credible intervals for the model based on the observed data. The outer, green envelopes are computed from the posterior distributions of the model parameter values. The green envelopes in effect correspond to the 95% credible intervals within which one expects any future observations to fall if a new experiment were performed using the same genotype and environment (Kruschke 2015; SAS Institute 2017).

#### Inclusion of Photosynthetic Capacity as a co-factor:

To account for potential carbon limitation caused by differences in photosynthetic capacity, we included the genotype-specific genotypic means for photosynthetic capacity (*A_max_*) as a co-factor in the prior distributions of the individual plant effects (Baker *et al.* 2018).

#### Phenotypic plasticity:

Prior to all analyses, phenotypic data were subjected to outlier analyses. Any observations more than three standard deviations from the mean were omitted. Visual inspection of histograms and quantile-quantile plots indicated that outlier exclusion yielded distributions closer to the normal distribution and never resulted in increased departure from the normal distribution (Baker *et al.* 2017; Baker *et al.* 2018). To detect environmental factors that might affect the correspondence between genotype and phenotype, we analyzed phenotypic datasets (File S2) from all years and both density treatments and tested for the main effects of genotype, treatment, and year and all possible interactions using the *lme4* and *lmerTest* packages in the R statistical environment (Bates *et al.* 2014; Kuznetsova *et al.* 2015; R core Team 2015). In these tests, all effects were considered random and block was nested within a year × treatment interaction. Significant main effects of environment (either year or treatment) were considered evidence of phenotypic plasticity, and interactions of treatment × genotype, year × genotype, or the three-way interaction of treatment × year × genotype were considered evidence for genetic variation in phenotypic plasticity.

#### Best Linear Unbiased Predictions (BLUPs):

Genotypic means were estimated by calculating BLUPs for all leaf FVT and spectral reflectance traits. BLUPs were calculated independently for each year and for UN and CR treatments in R using the *lmer* function in the *lme4* package while controlling for block effects (Bates *et al.* 2014; Kuznetsova *et al.* 2015). The random effects of block (nested within a year × treatment interaction) and genotype, treatment and year (and their two- and three-way interactions) were assessed using a series of chi-square tests that compare models with and without the given random effect (*rand* function in *lmerTest*).

#### QTL mapping:

QTL analyses were performed in R/qtl (Broman and Sen 2009) based on an updated and highly resolved RNA-seq based SNP map with an average distance of 0.7 cM between informative markers (Baker *et al.* 2015; Markelz *et al.* 2017). The *scanone* function was used to perform an initial round of interval mapping (1cM resolution with estimated genotyping errors of 0.001 using Haley Knott regression) to identify additive QTL. QTL model space was searched using an iterative process (*fitqtl*, *refineqtl*, and *addqtl)* to identify additional QTL while taking into account the effects of QTL identified by *scanone* and *addqtl*. All significance thresholds (0.95) were obtained using 10,000 *scanone* permutations (Broman *et al.* 2003; Broman and Sen 2009). QTL and their 1.5LOD confidence intervals are displayed using MapChart2.0 (Voorrips 2002).

We hypothesized that Bayesian methods might better estimate trait values in comparison to frequentist trait estimation, thereby improving phenotype-genotype associations estimated under QTL mapping. To compare QTL identified for Bayesian FVT model parameters with previous frequentist (least squares, LS) FVT model parameters (Baker *et al.*, 2015), we remapped the previously published LS parameters using the updated *B. rapa* genetic map and the same protocol as described above for the Bayesian parameters. Percent variance explained (PVE) is calculated as PVE = 100 × (1 - 10^(-2 LOD/ n)), where n is the number of genotypes.

#### QTL-by-environment interactions:

The effects of environment and year on QTL were assessed using a series of linear regression models and two-way ANOVAs in the *car* package for R (Fox and Weisberg 2011). P-values <0.05 for type III F-values of treatment × QTL maker, year × QTL maker, or the three-way interaction of treatment × year × QTL marker interactions were considered evidence of QTL by environment interactions. To avoid testing the same QTL multiple times, when QTL for the same trait across different treatments and years colocalized (as defined by overlapping 1.5 LOD confidence intervals) and expressed the same direction of effect, only one QTL × environment interaction is reported.

#### Predictive analyses:

For the purposes of predictive modeling, we chose uncrowded leaf widths (UNLW) from 2012 because we detected the most QTL for this trait. To predict growth phenotypes based on genotypes, we used a resampling approach. For each sample, we randomly excluded 5 genotypes (without replacement). We re-evaluated our Bayesian FVT models, extracted trait parameters, and calculated trait BLUPs as described above. We re-mapped QTL using the same methods as above, except we set the significance threshold for QTL detection to 0.90. Including QTL of marginal statistical significance but with potentially biologically meaningful effects increased the precision with which we could perform phenotypic predictions. We extracted the effect size and direction of each QTL identified for *Lmax* and *r* using the *effectplot* function in R/qtl and constructed predictive models for *Lmax* and *r* (Equation 3 and 4, respectively) where _i_ indicates the genotype in question, *$L\bar{m}ax$* and $\bar{r}$ are the population means for *Lmax* and *r*, respectively, which are added to the sum of the products of each QTL direction and effect size. We estimated values for *r* and *Lmax* parameter for each of the 5 randomly excluded genotypes based strictly on genotypic information and independent of any phenotypic information. We repeated this process 20 times, each time randomly selecting a different set of RILs to predict phenotypes based on genotypes (and to exclude from Bayesian modeling, BLUP estimation, and QTL mapping procedures). This approach allowed us to predict phenotypes based on genotypes for ∼100 different genotypes. Analyses departed slightly from n = 100 because we dropped genotypes when, for example, randomly selected genotypes represented parents of the RIL population and could not be used for mapping or had no genotypic information.

$$\mathrm{Equation 3}:L\max_{i}=\underline{L\max}+\sum_{j=1}^{n}\left(Direction_{\text{QTL}}\right)\left(Effect_{QTLj}\right)$$

$$\mathrm{Equation 4}:r_{i}=\bar{r}+\sum_{j=1}^{n}\left(Direction_{QTLj}\right)\left(Effect_{QTLj}\right)$$

We evaluated the success of our predictive models across environments in two ways. First, we examined the correlations between predicted (based on 2012 UN data) and observed *r* and *Lmax* (in 2012 UN and in 2012 CR). Second, we used predicted values for *r* and *Lmax* (along with a constant, *L_0_*, which estimated the initial value for leaf width) to predict logistic growth curves describing the increase in leaf width over time for each genotype. We plotted predicted growth curves (red line) in conjunction with measured phenotypic data (colored circles) for all replicate plants per genotype. We applied our Bayesian models to the observed phenotypic data (excluding the 5 predicted genotypes) and estimated growth curves (black lines) and credible intervals. We visually compared predicted growth curves to credible intervals surrounding observed phenotypes. When comparing predicted to observed growth curves for 2012 uncrowded data, we considered predictions successful if they fell within the 95% Bayesian credible regions for the model (yellow envelopes, Figure 2) and marginally successful if they fell within the 95% credible limits for future observation (green envelopes, Figure 2). When projecting across treatments (from uncrowded to crowded environments), we considered predictions successful if they fell within the green envelopes. We scored the proportion of successful or unsuccessful predictions for *r* and *Lmax* within each sample and assessed whether the proportion of successful predictions was greater than that expected by chance (0.50) using a Z-statistic. Replicate level phenotypic FVT data used in all analyses are available in File S2 and upon request.

### Data Availability

Data and code used in these analyses are previously published and can be found in the relevant citations or are included in the supplemental files of this publication including environmental data from the experimental field in 2010, 2011, and 2012 (Online Resource File S1) and replicate level phenotypic data used for analyses (Online Resource File S2).

## Results

### Phenotypic plasticity

We partitioned variation among genetic and environmental sources. There were significant main effects of block (nested within the treatment-by-year interaction) for all model parameters, indicating plasticity to unmeasured microenvironmental variation. The main effect of genotype was significant for all leaf parameters other than LL_*r* and LW_*r*, for which genetic variance was subsumed into environmental interaction terms. We found no evidence of phenotypic plasticity to the crowding treatment for any leaf trait (Table 1). By contrast, there were significant main effects of year on all leaf FVT parameters except leaf length duration of growth (LL_*d*), leaf width maximum size (LW_*Lmax*), and duration of growth (LW_*d*). We also observed evidence for genetic variation in the expression of phenotypic plasticity among years (genotype × year interactions) for all leaf FVT traits (Table 1). Interestingly, for leaf FVT estimating growth rates and durations (*d*, *iD*, *r*) the interaction term was always several fold larger in magnitude than the main effect of genotype as estimated by the test statistic value. In contrast, the main effect of genotype for FVT estimating final leaf size (*Lmax*) was ∼15-20 fold larger in magnitude than the corresponding genotype × year interaction term (Table 1). The three-way density treatment × year × genotype showed a similar pattern of strong significance for *iD*, *d* and *r* parameters and either non-significance for LW_*Lmax* or low magnitude in comparison to the main effect of genotype for LL_*Lmax* (Table 1). Notably, the patterns of plasticity were similar for LL and LW parameters, *e.g.*, the effect of genotype for both LL_*r* and LW_*r* is only significant in the two environmental interactions and *iD* and *d* both show similarly large effects of the two and three-way interactions involving year. In sum, the results suggest that the trajectory of leaf growth (estimated from *iD*, *d* and *r*) may be modulated by inter-annual environmental variation, but that the final size of leaves (estimated from *Lmax*) is less so.

**Table 1:** Block (nested within the interaction of Year and Treatment), treatment (treat), year, genotype, and their interactive effects in *Brassica rapa* inbred lines (RILs)

Trait	Model t-value (df)	Random Effects – Chi Square value (degrees of freedom)							
		Block (Treat × Year)	Treat	Year	Geno-type	Treat× Year	Geno-type × Treat	Geno-type × Year	Geno-type × Treat × Year
LL_*r*	5.63 (1.1)	112 (2)	2.23 (1)	6.75 (1)	0.03 (1)	0.00 (1)	1.68 (1)	34.3 (2)	95.7 (1)
	.	***	NS	**	NS	NS	NS	***	***
LL_*Lmax*	12.98 (1.2)	154 (2)	0.97 (1)	4.03 (1)	89.5 (1)	0.00 (1)	3.48 (1)	3.98 (1)	14.7 (1)
	*	***	NS	*	***	NS	.	*	***
LL_*iD*	6.45 (1.2)	94.83 (2)	1.37 (1)	8.80 (1)	28.17 (1)	0.00 (1)	0.00 (1)	74.5 (1)	11.61 (1)
	.	***	NS	**	***	NS	NS	***	***
LL_*d*	30.73 (2.0)	107.66 (2)	1.79 (1)	1.73 (1)	16.03 (1)	0.00 (1)	0.15 (1)	39.26 (1)	29.06 (1)
	***	***	NS	NS	***	NS	NS	***	***
LW_*r*	4.75 (1.5)	71.54 (2)	1.76 (1)	5.31 (1)	4.87 (1)	1.34 (1)	4.18 (1)	21.69 (1)	121.91 (1)
	.	***	NS	*	*	NS	*	***	***
LW_*Lmax*	13.34 (1.9)	95.0 (2)	1.84 (1)	2.51 (1)	118.0 (1)	0.00 (1)	10.5 (1)	7.93 (1)	5.26 (1)
	**	***	NS	NS	***	NS	**	**	*
LW_*iD*	5.63 (1.3)	59.8 (2)	1.25 (1)	9.61 (1)	25.8 (1)	0.00 (1)	0.64 (1)	97.4 (1)	13.4 (1)
	.	***	NS	**	***	NS	NS	***	***
LW_*d*	24.47 (2.0)	58.82 (2)	2.71 (1)	2.53 (1)	20.56 (2)	0.00 (1)	1.39 (1)	46.66 (1)	41.75 (1)
	**	***	.	NS	***	NS	NS	***	***

Signif. codes: 0 ‘***’ 0.001 ‘**’ 0.01 ‘*’ 0.05 ‘.’ 0.1 ‘ ’ 1.

We detected significant genetic variation among genotypes for all spectroradiometric indices except ari2 (which was marginally significant, *P* < 0.01) and cri. We found no evidence for phenotypic plasticity for population mean trait values for any of the indices or R:FR ratio, because the main effects of treatment and year were always non-significant (Table 2). However, we found evidence for genetic variation in phenotypic plasticity across density treatments for mcari, sipi2, tcari, and psri as well as across years for npci. There were no significant three-way interactions of genotype × year × treatment.

**Table 2:** Fixed and random effects for spectroradiometric data. Note that there is no data from 2011 for CR spectral indices

Trait	Model t-value (df)	**Random Effects – Chi Square value (degrees of freedom)**							
		Block (Treat × Year)	Treat	Year	Geno-type	Treat × Year	Treat × Geno-type	Geno-type × Year	Treat × Year × Genotype
mcari1	3.49 (1.9)	47.9 (2)	0.63 (2)	0.94 (1)	17.6 (1)	0.00 (1)	6.58 (1)	0.00 (1)	0.00 (1)
	.	***	NS	NS	***	NS	*	NS	NS
mcari2	3.70 (1.2)	89.6 (2)	0.34 (1)	1.85 (1)	33.7 (1)	0.00 (1)	2.23 (1)	0.00 (1)	0.00 (1)
		***	NS	NS	***	NS	NS	NS	NS
Mtci	6.67 (1.0)	555.0 (2)	0.00 (1)	3.31 (2)	17.5 (1)	0.00 (1)	0.88 (1)	3.13 (1)	0.00 (1)
	.	***	NS	.	***	NS	NS	.	NS
sipi2	9.57 (1.1)	42.2 (2)	0.00 (1)	2.19 (1)	10.29 (1)	0.00 (1)	9.61 (1)	0.00 (1)	0.00 (1)
	.	***	NS	NS	**	NS	**	NS	NS
Tcari	4.78 (1.6)	21.2 (2)	0.47 (1)	1.15 (1)	8.07 (1)	0.00 (1)	5.37 (1)	0.00 (1)	0.00 (1)
	.	***	NS	NS	**	NS	*	NS	NS
ari1	2.90 (1.1)	299.0 (2)	0.13 (1)	2.19 (1)	47.8 (1)	0.00 (1)	0.00 (1)	0.24 (1)	2.50 (1)
	NS	***	NS	NS	***	NS	NS	NS	NS
ari2	−12.00 (1.0)	339.0 (2)	0.4 (1)	0.26 (1)	3.0 (1)	0.00 (1)	0.00 (1)	0.00 (1)	0.07 (1)
	*	***	NS	NS	.	NS	NS	NS	NS
Cri	5.00 (1.5)	329.0 (2)	0.38 (1)	1.06 (1)	0.00 (1)	0.00 (1)	0.01 (1)	0.55 (1)	0.00 (1)
	.	***	NS	NS	NS	NS	NS	NS	NS
Npci	3.54 (1.1)	261.6 (2)	0.13 (1)	2.65 (1)	19.1 (1)	0.00 (1)	0.00 (1)	4.11 (1)	0.49 (1)
	NS	***	NS	NS	***	NS	NS	*	NS
pri2	1.70 (1.2)	503.0 (2)	0.26 (1)	1.75 (1)	47.1 (1)	0.00 (1)	1.71 (1)	1.57 (1)	0.12 (1)
	NS	***	NS	NS	***	NS	NS	NS	NS
Psri	1.81 (1.1)	2.65 (2)	0.12 (1)	2.36 (1)	28.3 (1)	0.00 (1)	5.22 (1)	3.41 (1)	0.00 (1)
	NS	***	NS	NS	***	NS	*	.	NS
Wi	17.6 (1.3)	614.8 (2)	0.24 (1)	1.92 (1)	13.93 (1)	0.00 (1)	0.22 (1)	0.00 (1)	1.27 (1)
	*	***	NS	NS	***	NS	NS	NS	NS
RFR	3.26 (1.0) NS	442.0 (2)	0.07 (1)	2.58 (1)	15.07 (1)	0.0 (1)	1.49 (1)	0.0 (1)	0.83 (1)
		***	NS	NS	***	NS	NS	NS	NS

Signif. codes: 0 ‘***’ 0.001 ‘**’ 0.01 ‘*’ 0.05 ‘.’ 0.1 ‘ ’ 1.

### Comparison of QTL mapping using Frequentist *vs.* Bayesian trait estimation

To assess the effectiveness of Frequentist (least square or LS) *vs*. Bayesian procedures in estimating FVT, we mapped 2012 FVT parameters using genotypic values arising from LS (Baker *et al.* 2015) and Bayesian FVT estimation procedures, the same map, and the same mapping procedures as previously described. Bayesian FVT estimation outperformed LS FVT modeling for all metrics considered. Bayesian trait estimation yielded 33 QTL compared to 26 identified via LS approaches. On average, Bayesian QTL tended to have greater LOD scores than QTL mapped for LS-estimated traits (4.53 *vs.* 4.25, respectively; *P* = 0.065) and tended to explained a greater percent variance (16.0% *vs.* 15.8%, respectively; *P* = 0.067) based on a Wilcox rank-order tests. Bayesian and LS FVT modeling often identified the same QTL, based on overlapping confidence intervals (14 QTL). However, each method also identified unique QTL (with non-overlapping confidence intervals). Bayesian modeling yielded 19 unique QTL (File S3).

#### Additive QTL:

**Figure 1:**
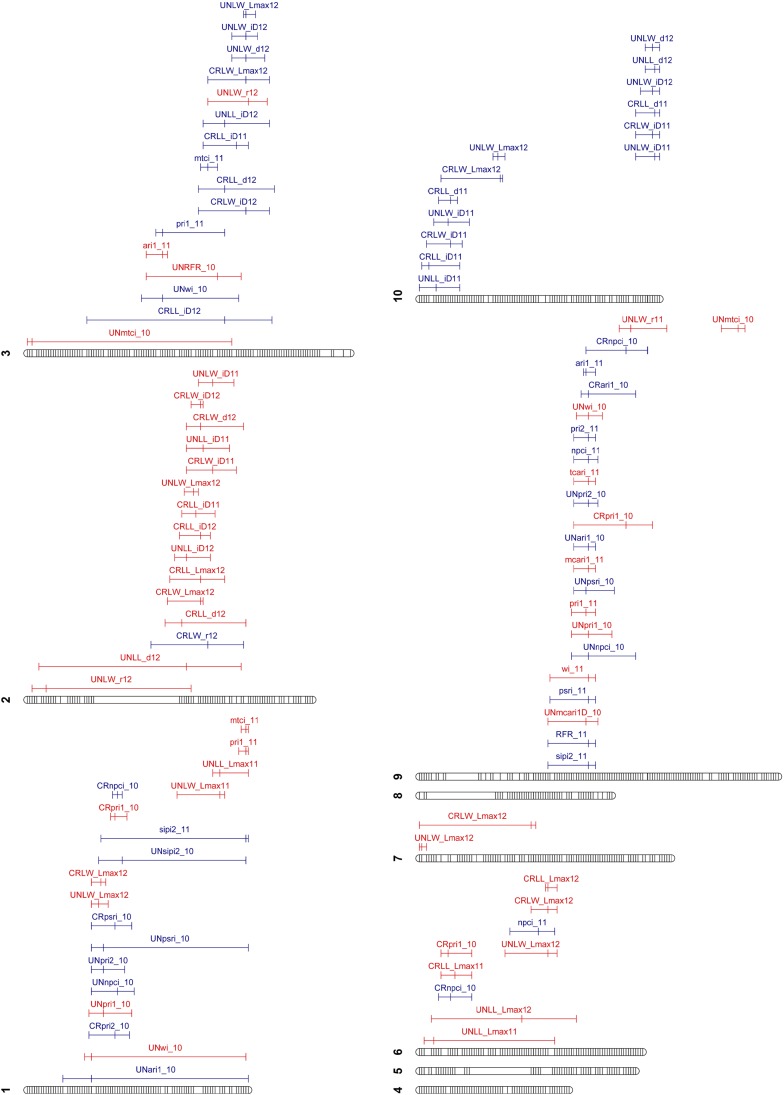
RNA-seq based linkage map of *Brassica rapa* with QTL and their 1.5 LOD confidence intervals. The 1482 SNP-based markers are relatively evenly dispersed across each of the ten chromosomes. Gaps in markers are indications of low expression associated with centromeres (or ancient centromeres). Positive QTL (with respect to IMB211) are red and negative QTL are blue. Overlapping 1.5 LOD confidence intervals are interpreted as evidence for colocalization of QTL. UN, uncrowded and CR, crowded treatments; LW, leaf width; LL, leaf length; *Lmax*, maximum estimated leaf size (mm); *r*, growth rate; *d*, duration of growth; *iD*, timing of the switch between accelerating and decelerating growth; 10, 11, and 12 represent QTL identified in 2010, 2011, and 2012 field seasons, respectively. Specific cM positions, percent variance explained, and genomic markers can be found in File S3.

Having compared the mapping results under the two trait estimation procedures, we proceeded with analyses of QTL based on Bayesian trait estimation. We detected 96 additive QTL across seven of the ten *B. rapa* chromosomes with no QTL detected on chromosomes four, five and eight (Figure 1 and File S3). However, because many of the 1.5 LOD support limits for individual QTL overlap, an alternative interpretation is that we identified as few as 14 highly pleiotropic QTL. Individual QTL had LOD scores of between 3.12 and 10 representing between 8.07 (UNLL_*iD11*) and 32.1% (UNLW_*d12*) of the genotypic variation. Consistent with greater genetic variances for 2011 compared to 2012 leaf FVT traits (Table 1), we detected more QTL for the 2012 than 2011 field seasons (41 *vs.* 15).

The genetic architecture underlying LL and LW FVT is similar as demonstrated by colocalization of many LL and LW FVT QTL. For instance, QTL jointly affect *Lmax* for leaf length and width (bottom of chromosome 1, middle of chromosome 2, middle of chromosome 6) as well as *d* or *iD* for leaf length and width (middle of chromosome 2, middle of chromosome 3, and top and bottom of chromosome 10; Figure 1 and File S3). QTL frequently colocalized across density treatments, that is, a QTL affecting an LL parameter in the CR treatment tended to also have a significant additive effect in the UN treatment (*e.g.*, for *Lmax* UN and CR QTL colocalize in the middle of chromosomes 3 and 6, bottom of chromosome 7 and the top of chromosome 10; Figure 1 and File S3).

QTL for spectroradiometric indices often formed clusters within the genome (*e.g.*, QTL on the top of chromosomes 1 and middle of chromosome 9). QTL for spectroradiometric data also colocalize with *Lmax* FVT QTL (*e.g.*, UNLW_*Lmax12*, CRLW*_Lmax12*, UNLW_*Lmax11*, and UNLL_*Lmax11* with mtci, pri1, sipi2, psri, pri2, wi, and ari1 on chromosome 1). Spectroradiometric indices, however, rarely colocalize with FVT parameters describing leaf growth rates or duration (for an exception, see mtci, pri1, ari1, RFR, and wi on chromosome 3, which co-localize with multiple estimates of duration; Figure 1).

### QTL-by-environment interactions

Consistent with the limited genotype × treatment interactions, we found no significant QTL × density treatment effects for FVT parameters describing leaf development. An additional field season worth of data allowed us to analyze QTL × year and QTL × treatment × year interactions for leaf FVT parameters. Consistent with the highly significant genotype × year interactions (Table 1), multiple QTL underlying leaf growth rates (*r)*, durations (*d*), and inflection sizes (*iD*) exhibited year × QTL interactions (Table 3). By contrast, FVT estimating final leaf sizes (*Lmax*) did not exhibit any significant QTL × year interactions (Table 3).

**Table 3:** Significant QTL × Environment (density treatment and year) interactions based on Type III sums of squares. Note that for spectral indices and RFR, there are no three-way interactions

		**Treat** × **QTL**	**Year** × **QTL**	**Year** × **QTL** × **Treatment**
**Trait**	**QTL marker**	**F(Df)**	**F(Df)**	**F(Df)**
***LL_d***	A06x16894473	0.19 (1,464)	4.97 (1, 464) *	0.19 (1,464)
***LL_iD***	A10x2471393	0.14 (1,464)	8.17 (1,464) *	0.14 (1,464)
***LW_d***	A01x8348377	0.89 (2, 460)	3.72 (2,460) *	0.89 (2,460)
	A03x17233425	0.87 (1,464)	6.19 (1,464) *	0.87 (1, 464)
	A06x17027456	1.01(1,464)	6.41 (1,464) *	1.01 (1,464)
***LW_iD***	A10x2471393	0.26 (1,464)	9.15 (1,464) **	0.26 (1,464)
***LW_r***	A02x12174045	0.24 (1,464)	9.66 (1,464) *	0.24 (1,464)
	A02x966946	1.40 (2,464)	4.46 (2,464) *	1.40 (2,464)
**mtci**	A03x356060	5.36 (1,234) * (*)	5.47 (1,239) *	
	A09x34851227	6.22 (1,234) * (*)	5.48 (1,239) *	
**npci**	A01x9511676	0.26 (1,234)	15.31 (1,239) *	
	A06x19335038	1.61 (1,234)	4.51 (1,329) *	
**pri1**	A01x26649666	10.89 (1,234) **	14.93 (1,238) ***	
	A01x9400632	31.54 (1,234) ***	31.68 (1,239) ***	
	A03x10583907	8.66 (1,234) **	12.01 (1,239) ***	
	A09x16619967	21.28 (1,234) ***	27.65 (1,239) ***	
***sipi2***	A01x9927004	14.72 (1,234) ***	0.00 (1,238)	
	A09x16619967	8.55 (1,234) **	5.94 (1,239) *	
**tcari**	A09x16619967	1.00 (1,234)	13.31 (1,239) ***	
**wi**	A01x8029418	3.19 (2,232) *	3.57 (2,237) *	
	A03x10583907	4.86 (1,234) *	7.22 (1,239) **	
	A09x16619967	3.93 (1,234) *	2.75 (1,329)	
**RFR**	A03x15439617	14.47 (1,234) ***	0.15 (1,239)	
	A09x16619967	5.78 (1,234) *	4.00 (1,239) *	

Signif. codes: 0 ‘***’ 0.001 ‘**’ 0.01 ‘*’ 0.05 ‘.’ 0.1 ‘ ’ 1.

For several spectroradiometric QTL, significant QTL × density treatment (2011 only) and QTL × year (uncrowded treatment only) effects were detected (Table 3). Three-way interactions for spectroradiometric traits and environmental variables were not tested because spectroradiometric data were only collected on both treatments in one year.

### Predictive modeling

**Figure 2:**
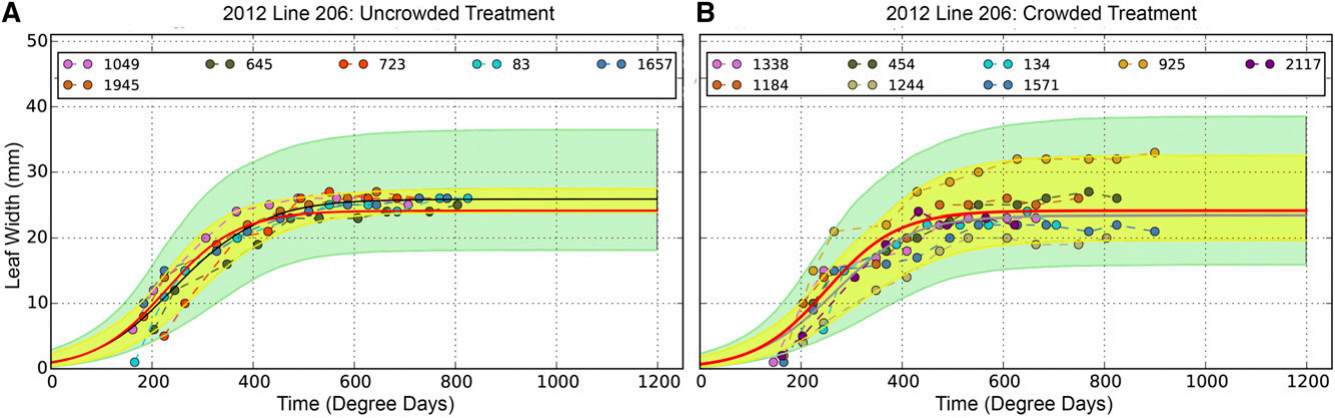
Predicted and measured phenotypes for genotype 206 in (A) Uncrowded and (B) Crowded treatment during the 2012 growing season. Colored circles represent measured phenotypic data from multiple individuals of genotype 206. The black line is a logistic growth curve fitted to the genotypic mean using Bayesian routines. Any new observation for an individual from genotype 206 grown in the same conditions is predicted to fall within the green 95% credible envelope. Our predicted phenotype (based on QTL data and incorporated into the logistic functions from Equation 3 and 4; red growth curve) falls within the yellow 95% credible envelope for the fitted logistic growth curve.

We predicted leaf width growth dynamics (*r* and *Lmax*) for randomly chosen genotypes grown in the uncrowded treatment in the 2012 field season. We evaluated our predictions using two methods. First, we compared our predictions to genotypic means estimated from the corresponding phenotypic values (BLUPs) using correlation analyses. Values of *r* and *Lmax* predicted from QTL-based genotypic models were significantly genetically correlated with observed trait means for UN 2012 LW_*r* (r = 0.26, df = 94, *P* = 0.01) and UN 2012 LW_*Lmax* (r = 0.61, df = 94, *P* = 3.9e-11). The relatively low correlation value for LW_*r* likely reflects the fact that we detected fewer QTL for LW_*r* and therefore our predicted values for LW_*r* were less precise than those for *Lmax*. Second, we asked whether our estimated predictions fell within the credible envelopes surrounding replicate-level phenotypic data. Predicted LW_*r* and LW_*Lmax* fell within the yellow credible envelope 87% and 85% of the time, respectively, and these predictions were considered successful (Figure 2 & File S4). For both *r* and *Lmax*, our success rates for prediction were significantly greater than chance (*i.e.*, 50%; Z = 3.29, *P* = 5e-04). Predicted values of *r* and *Lmax* fell within the green credible envelope for predicted future phenotypes for a given genotype grown in the same conditions (Figure 2) 97% and 100% of the time, respectively, and these were considered marginally successful.

We asked whether our models based on UN QTL could predict phenotypes expressed across density environments, given the similarity in genetic architecture between the UN and CR treatments (lack of QTL × E in 2012; Table 3). Predicted values of LW_*r* were uncorrelated with CR observed trait means of *r* (r = 0.16, df = 94, *P* = 0.1205), but predicted values of LW_*Lmax* were significantly correlated with observed CR *Lmax* means (r = 0.58, df = 94, *P* = 4.19e-10). We considered predictions across the density treatments successful based on less stringent criteria than within a single environment. Specifically, we used the wider green credible intervals. When evaluating our predictions of *r* and *Lmax* based on credible envelopes, our predictions were successful 100% and 98% of the time, respectively. Our successful prediction rates were significantly greater than chance (0.50) for LW_*r* (Z = 4.9, *P* < 0.0003) and LW_*Lmax* (Z = 2.5, *P* = 0.0052). The high rate of successful predictions despite lack of genetic correlation for LW_*r* is likely because the credible envelopes are generated from distributions based on observed phenotypic data whereas the correlations are based solely on means of predicted values *vs.* observed phenotypes.

## Discussion

Predicting phenotypes based on genotypes is a central objective of evolutionary developmental biology and a key component of breeding programs (Moczek *et al.* 2015). However, predictive models require understanding the genetic architecture of phenotypes throughout ontogeny because the trait of interest may be influenced by different combinations of genetic and environmental factors at different times during growth and development (Benjamini and Hochberg 1995; Benjamini and Yekutieli 2001). Quantifying growth trajectories is also important because selection may act throughout ontogeny rather than on final phenotypes alone (Dobzhansky 1956; Moose and Mumm 2008; Baker *et al.* 2015). Final phenotypes may also be contingent upon prior environments and developmental events (Diggle 1997; Weinig and Delph 2001; Dechaine *et al.* 2014). To predict growth phenotypes, we explored the genetic basis and environmental dependencies (plasticity) of non-linear leaf length and width growth trajectories utilizing mathematical modeling to fit logistic growth curves to ontogenetic data in a population of *Brassica rapa* RILs. We evaluated the effectiveness of Bayesian *vs.* frequentist approaches to Function-Valued Trait (FVT) estimation by mapping QTL for FVT parameters. Our “parameters as data” approach to FVT modeling allowed us to detect differences in the genetic architecture and environmental sensitivities among growth parameters (i.g. rates *vs.* durations *vs.* final sizes; Jaffrézic and Pletcher 2000; Kingsolver *et al.* 2001; Wu and Lin 2006; Stinchcombe *et al.* 2010; Baker *et al.* 2015; Jiang *et al.* 2015; Zhang *et al.* 2017). Notably, although growth parameters (*r*, *Lmax*, *d*, *iD*) showed different genetic architectures, the genetic basis of any single parameter was very similar between leaf length and width. Finally, we built and tested QTL-based models for predicting growth phenotypes from genotypic data.

### Phenotypic plasticity of leaf FVT

Environmental inputs can have profound influences on phenotypic expression. Understanding how genotypes plastically respond to the environment over the course of development is a first step in developing cultivars that are optimally suited to their growth conditions (Kang 1997). In our study, spectroradiometric indices did not often express phenotypic plasticity in response to either density or interannual microclimatic variation (*i.e.*, year; Table 3). For leaf FVT, the plastic responses to interannual microclimatic variation were much more pronounced than responses to density. The main effect of crowding was never significant for any leaf FVT, while plasticity to year was often significant. Additionally, we identified genetic variation for phenotypic plasticity (that is, changes in rank-order genotypic values and variances) among all leaf FVT (Tables 1), indicating that different genotypes respond to inter-annual variation in different ways. However, the magnitude of the genotype-by-environment response was several times larger for growth parameters (*r*, *d*, and *iD*) compared to final size (*Lmax*), indicating that ontogenetic aspects of leaf size (for both length and width) are more plastic than final sizes (Table 1).

### Bayesian *vs.* frequentists Function Valued Trait approaches

To investigate the genomic architecture underlying leaf FVT across environments, we mapped QTL for FVT and asked whether Bayesian approaches to FVT modeling and trait estimation resulted in improved mapping compared to frequentist (least squares) approaches (Baker *et al.*, 2015). When comparing mapping results from Bayesian and frequentist approaches for 2012 data, Bayesian routines yielded higher estimates of FVT trait heritability (Baker *et al.* 2018). In side-by-side comparisons in the current study, parameters extracted from Bayesian FVT models mapped a greater number of QTL than those estimated from frequentist FVT models (33 *vs.* 26), had marginally higher LOD scores (*P* = 0.065) and tended to explain a larger proportion of phenotypic variance (*P* = 0.067). We attribute these improvements in QTL detection and resolution to the fact that hierarchical Bayesian models extract global information more efficiently from the data and reduce error propagation compared to frequentist approaches (Charmet 2000; Baker *et al.* 2018) thereby improving trait estimation and phenotype-genotype association tests that are the basis of QTL mapping.

### Genetic architecture of spectroradiometric indices and leaf length and width FVT

Few studies have compared the genetic architecture of spectroradiometric reflectance patterns, developmental dynamics, and final phenotypes. Our QTL analysis of spectroradiometric indices and a R:FR ratio indicates that these indices are largely genetically distinct from FVT parameters of leaf development. When QTL for spectroradiometric indices do colocalize with QTL for FVT parameters, these tend to be QTL for final sizes rather than parameters that describe growth dynamics (*e.g.*, chromosome 1 in Figure 1), indicating that single time point spectral indices may serve as suitable proxies for final leaf sizes, but not growth dynamics. With regard to leaf length and width FVT, functional annotation of individual genes have identified loci that specifically affect leaf length and have little to no effect on leaf width (Tsukaya 2005). However, quantitative genetic studies often find that LL and LW are tightly genetically correlated and may be pleiotropically regulated (Fujita *et al.* 2009; Baker *et al.* 2015; Drost *et al.* 2015). Previously, we found that FVT parameters for leaf length and leaf width (*e.g.*, *LW_r* and *LL_r*; LW_*d* and LL_*d*) were highly genetically correlated within an environment and showed similar plasticities across environments. In addition to correlations of the *same* FVT parameter between LL and LW, we found that *different* FVT parameters were moderately correlated (Baker *et al.* 2015; Baker *et al.* 2018). Estimates of final sizes (*Lmax*) were genetically correlated with aspects of growth such as rates (*r*) and duration (*d*); however, these correlations were not perfect (Baker *et al.*, 2015). Here, we found that QTL for Bayesian FVT parameters describing *r* and *Lmax* rarely colocalized (see LW_*r* and LW_*Lmax* from 2012 on chromosome 3 (UN) and 2 (CR) for exceptions; Figure 1). Our results confirm previous studies of the genetic architecture of leaf growth dynamics *vs.* final size (Baker *et al.*, 2015) and support the general hypothesis that leaf growth and final size have different genetic architectures. The independent genetic control of final size and growth dynamics implies that these may be independently selected upon to increase a plant’s space occupancy or light harvesting ability (reviewed in Wu *et al.* 2004; Wu and Lin 2006).

### Genetic and environmental interactions for leaf FVT

Within a single organ, environmental sensitivities may differ among traits and across developmental time (Wright and McConnaughay 2002; Sugiyama and Gotoh 2010). Robust QTL-by-environment detection is dependent upon large sample sizes and multiple environments. In our RIL population of over 100 lines, we observed QTL-by-environment interactions that corresponded with genotype-by-environment interactions for leaf FVT (Compare Tables 1 and 3). Specifically, we found no QTL-by-density treatment interactions. Two traits (LW_*iD* and LW_*r*) had significant QTL-by-year interactions, and these QTL-by-environment interactions likely contribute to the population-level phenotypic plasticity exhibited by LW*_iD* and LW*_r* in response to interannual environmental variation (year). We also observed significant QTL-by-year interactions for QTL underlying traits that did not exhibit phenotypic plasticity as assessed by the main effect of year. For example, QTL-by-year interactions for growth duration (*d*) likely underlie rank order differences in genotypic means and changes in trait variances across growing seasons (compare Tables 1 and 3). We found strong evidence for QTL-by-environment interactions for aspects of growth dynamics (rates, durations, and inflection points), but not for final leaf sizes. Notably, the differing extent of QTL-by-environment interactions among FVT parameters further supports the hypothesis that leaf growth dynamics and final size have independent genetic underpinnings.

### Predicting phenotypes based on genotypes (QTL)

Reliably predicting phenotypes is important to understanding evolutionary dynamics, selecting seed sources for ecological restoration, and designing molecular breeding programs. Predictions of fitness and yield may be improved by incorporating information about plant growth (Assefa *et al.* 2015) and physiology (Amelong *et al.* 2015). However, most models constructed to predict phenotypes based on genomic data focus on single-time-points such as seedling traits (Cooper *et al.* 2005; Xu *et al.* 2011), disease resistance (Pace *et al.* 2015), or components of yield (Yin *et al.* 2000; Bao *et al.* 2015). Models that do incorporate ontogeny tend to treat sequential time points independently (*e.g.*, Reymond *et al.* 2003) rather than incorporating interdependence of ontogenetic data via FVT models. Further, FVT modeling can describe non-linear growth curves that capture the potentially independent aspects of exponential and asymptotic growth phases. We developed models to predict complete growth curves based on QTL underlying FVT parameters (*r* and *Lmax*; Equations 6 & 7). In the same (uncrowded) environment, FVT parameters predicted based on genotypic information were significantly correlated with independently observed FVT phenotypes and components of our predicted logistic growth curves fell within 95% credible intervals for the actual phenotypic data 85% of the time. These success rates are significantly greater than chance and equivalent to predictive models for grain yield that integrate crop growth models and whole genome information (Technow *et al.* 2015).

We also asked whether our models could provide accurate predictions across environments. Consistent with the lack of significant main effect of density treatment and QTL-by-density interactions, our models successfully predicted phenotypes in 2012 crowded conditions 98-100% of the time. As for other complex traits, future work on predictive modeling for multi-locus non-linear growth traits should focus on expanding beyond additive models to include interactive effects such as epistasis and to explicitly incorporate environmental parameters to account for phenotypic plasticity and gene by environment interactions (Yin *et al.* 2004; Tardieu *et al.* 2005; Chenu *et al.* 2009; Bustos-Korts *et al.* 2016).

### Conclusions

A major goal in evolutionary biology and crop science is understanding the connections between genotypes and phenotypes. Our Bayesian FVT trait estimation approach to leaf growth isolates leaf developmental genetic programs by factoring out endogenous influences on growth and development such as genotype-specific photosynthetic capacities. We detect differences in the environmental sensitivity among traits: the strength of the plastic response was much stronger in growth traits compared to final sizes. We also detect distinct genomic architectures underlying different components of leaf growth curves. The Bayesian approach to FVT modeling provides superior QTL detection compared to previous frequentist approaches. Together, our data indicate that leaf growth dynamics and final sizes have different genomic architectures and different patterns of environmental sensitivity. Genotypic models effectively predicted FVT phenotypes, suggesting that comparatively simple QTL models can capture the non-linearity intrinsic to some FVT in this species; elaboration of these models to include environmental effects and expression data are a topic of ongoing research.

## Supplementary Material

Supplemental Material is available online at www.g3journal.org/lookup/suppl/doi:10.1534/g3.117.300350/-/DC1

## References

[bib2] Amelong A. Gambín B. L. Severini A. D. Borrás L., 2015 Predicting maize kernel number using QTL information. Field Crops Res. 172: 119–131. 10.1016/j.fcr.2014.11.014

[bib3] An N. Goldsby A. L. Price K. P. Bremer D. J., 2015 Using hyperspectral radiometry to predict the green leaf area index of turfgrass. Int. J. Remote Sens. 36(5): 1470–1483. 10.1080/01431161.2015.1014971

[bib4] Assefa T. Wu J. Beebe S. E. Rao I. M. Marcomin D., 2015 Improving adaptation to drought stress in small red common bean: phenotypic differences and predicted genotypic effects on grain yield, yield components and harvest index. Euphytica 203(3): 477–489. 10.1007/s10681-014-1242-x

[bib5] Baker R. L. Leong W. F. An N. Brock M. T. Rubin M. J., 2018 Bayesian estimation and use of high-throughput remote sensing indices for quantitative genetic analyses of leaf growth. Theor. Appl. Genet. 131(2): 283–298. 10.1007/s00122-017-3001-6 29058049

[bib6] Baker R. L. Leong W. F. Brock M. T. Markelz R. Covington M. F., 2015 Modeling development and quantitative trait mapping reveal independent genetic modules for leaf size and shape. New Phytol. 208(1): 257–268. 10.1111/nph.13509 26083847

[bib7] Baker R. L. Yarkhunova Y. Vidal K. Ewers B. E. Weinig C., 2017 Polyploidy and the relationship between leaf structure and function: implications for correlated evolution of anatomy, morphology, and physiology in *Brassica*. BMC Plant Biol. 17(1): 3 10.1186/s12870-016-0957-3 28056801 PMC5217196

[bib8] Bao Y. Kurle J. E. Anderson G. Young N. D., 2015 Association mapping and genomic prediction for resistance to sudden death syndrome in early maturing soybean germplasm. Mol. Breed. 35(6): 128 10.1007/s11032-015-0324-3 25999779 PMC4434860

[bib9] Bates, D., Maechler, M., Bolker, B. and Walker, S., 2014 lmer: Linear mixed-effects models using Eigen and S4., pp.

[bib10] Benjamini Y. Hochberg Y., 1995 Controlling the False Discovery Rate: A Practical and Powerful Approach to Multiple Testing. J. R. Stat. Soc. B 57: 289–300.

[bib11] Benjamini, Y., and D. Yekutieli, 2001 The control of the false discovery rate in multiple testing under dependency. 1165–1188.

[bib12] Bradshaw A. D., 1965 Evolutionary significance of phenotypic plasticity in plants. Adv. Genet. 13: 115–155.

[bib13] Brock M. T. Weinig C., 2007 Plasticity and environment-specific covariances: An investigation of floral-vegetative and within flower correlations. Evolution 61(12): 2913–2924. 10.1111/j.1558-5646.2007.00240.x 17941839

[bib14] Broman K. W. Sen S., 2009 A Guide to QTL Mapping with R/qtl, Springer, New York 10.1007/978-0-387-92125-9

[bib15] Broman K. W. Wu H. Sen S. Churchill G. A., 2003 R/qtl: QTL mapping in experimental crosses. Bioinformatics 19(7): 889–890. 10.1093/bioinformatics/btg112 12724300

[bib16] Bustos-Korts D. Malosetti M. Chapman S. van Eeuwijk F., 2016 Modelling of Genotype by Environment Interaction and Prediction of Complex Traits across Multiple Environments as a Synthesis of Crop Growth Modelling, Genetics and Statistics, pp. 55–82 in Crop Systems Biology: Narrowing the gaps between crop modelling and genetics, edited by Yin X. Struik P. C. Springer, Heidelberg 10.1007/978-3-319-20562-5_3

[bib17] Charmet G., 2000 Power and accuracy of QTL detection: simulation studies of one-QTL models. Agronomie 20(3): 309–323. 10.1051/agro:2000129

[bib18] Chenu K. Chapman S. C. Tardieu F. McLean G. Welcker C., 2009 Simulating the Yield Impacts of Organ-Level Quantitative Trait Loci Associated With Drought Response in Maize: A “Gene-to-Phenotype” Modeling Approach. Genetics 183(4): 1507–1523. 10.1534/genetics.109.105429 19786622 PMC2787435

[bib88] Chib S. Greenberg E., 1995 Understanding the metropolis-hasting algorithm. The American Statistician. 49(4): 327–335. 10.2307/2684568

[bib19] Cooper M. Podlich D. W. Smith O. S., 2005 Gene-to-phenotype models and complex trait genetics. Aust. J. Agric. Res. 56(9): 895–918. 10.1071/AR05154

[bib20] Crossa, J., Y. Beyene, S. Kassa, P. Pérez, J. M. Hickey *et al.*, 2013 Genomic Prediction in Maize Breeding Populations with Genotyping-by-Sequencing. G3: Genes|Genomes|Genetics 3: 1903–1926. DOI: 10.1534/g3.113.008227 PMC3815055 24022750

[bib21] Dash J. Curran P. J., 2004 The MERIS terrestrial chlorophyll index. Int. J. Remote Sens. 25(23): 5403–5413. 10.1080/0143116042000274015

[bib22] Debat V. David P., 2001 Mapping phenotypes: canalization, plasticity and developmental stability. Trends Ecol. Evol. 16(10): 555–561. 10.1016/S0169-5347(01)02266-2

[bib23] Dechaine J. M. Brock M. T. Iniguez-Luy F. L. Weinig C., 2014 Quantitative trait loci × environment interactions for plant morphology vary over ontogeny in *Brassica rapa*. New Phytol. 201(2): 657–669. 10.1111/nph.12520 26012723

[bib24] Diggle P. K., 1997 Ontogenetic contingency and floral morphology: The effects of architecture and resource limitation. Int. J. Plant Sci. 158(S6): S99–S107. 10.1086/297510

[bib25] Diggle P. K., 2002 A developmental morphologist’s perspective on plasticity. Evol. Ecol. 16(3): 267–283. 10.1023/A:1019680527788

[bib26] Dobzhansky T., 1956 What is an Adaptive Trait? Am. Nat. 90(855): 337–347. 10.1086/281944

[bib27] Drost D. R. Puranik S. Novaes E. Novaes C. R. D. B. Dervinis C., 2015 Genetical genomics of *Populus* leaf shape variation. BMC Plant Biol. 15(1): 166 10.1186/s12870-015-0557-7 26122556 PMC4486686

[bib28] Edwards C. E. Ewers B. E. McClung C. R. Lou P. Weinig C., 2012 Quantitative Variation in Water-Use Efficiency across Water Regimes and Its Relationship with Circadian, Vegetative, Reproductive, and Leaf Gas-Exchange Traits. Mol. Plant 5(3): 653–668. 10.1093/mp/sss004 22319207

[bib29] Fox J. Weisberg S., 2011 *An {R} companion to applied regression*, Sage, Thousand Oaks, CA.

[bib30] Fujita D. Santos R. E. Ebron L. A. Telebanco-Yanoria M. J. Kato H., 2009 Development of introgression lines of an Indica-type rice variety, IR64, for unique agronomic traits and detection of the responsible chromosomal regions. Field Crops Res. 114(2): 244–254. 10.1016/j.fcr.2009.08.004

[bib31] Garbulsky M. F. Peñuelas J. Gamon J. Inoue Y. Filella I., 2011 The photochemical reflectance index (PRI) and the remote sensing of leaf, canopy and ecosystem radiation use efficiencies: A review and meta-analysis. Remote Sens. Environ. 115(2): 281–297. 10.1016/j.rse.2010.08.023

[bib32] Gitelson A. A. Merzlyak M. N. Chivkunova O. B., 2001 Optical Properties and Nondestructive Estimation of Anthocyanin Content in Plant Leaves. Photochem. Photobiol. 74(1): 38–45. 10.1562/0031-8655(2001)074<0038:OPANEO>2.0.CO;2 11460535

[bib33] Gitelson A. A. Zur Y. Chivkunova O. B. Merzlyak M. N., 2002 Assessing Carotenoid Content in Plant Leaves with Reflectance Spectroscopy. Photochem. Photobiol. 75(3): 272–281. 10.1562/0031-8655(2002)075<0272:ACCIPL>2.0.CO;2 11950093

[bib34] Griswold C. K. Gomulkiewicz R. Heckman N., 2008 Hypothesis testing in comparative and experimental studies of function-valued traits. Evolution 62(5): 1229–1242. 10.1111/j.1558-5646.2008.00340.x 18266991

[bib35] Haboudane D. Miller J. R. Pattey E. Zarco-Tejada P. J. Strachan I. B., 2004 Hyperspectral vegetation indices and novel algorithms for predicting green LAI of crop canopies: Modeling and validation in the context of precision agriculture. Remote Sens. Environ. 90(3): 337–352. 10.1016/j.rse.2003.12.013

[bib36] Haboudane D. Miller J. R. Tremblay N. Zarco-Tejada P. J. Dextraze L., 2002 Integrated narrow-band vegetation indices for prediction of crop chlorophyll content for application to precision agriculture. Remote Sens. Environ. 81(2-3): 416–426. 10.1016/S0034-4257(02)00018-4

[bib37] Hall M. C. Dworkin I. Ungerer M. C. Purugganan M., 2007 Genetics of microenvironmental canalization in *Arabidopsis thaliana*. Proc. Natl. Acad. Sci. USA 104(34): 13717–13722. 10.1073/pnas.0701936104 17698961 PMC1959448

[bib38] Hammer G. L. Chapman S. van Oosterom E. Podlich D. W., 2005 Trait physiology and crop modelling as a framework to link phenotypic complexity to underlying genetic systems. Aust. J. Agric. Res. 56(9): 947–960. 10.1071/AR05157

[bib39] Hinata K. Prakash S., 1984 Ethnobotany and evolutionary origin of Indian oleiferous Brassicae. Indian J. Genet. Plant Breed. 44: 102–112.

[bib40] Iniguez-Luy F. Lukens L. Farnham M. Amasino R. Osborn T., 2009 Development of public immortal mapping populations, molecular markers and linkage maps for rapid cycling *Brassica rapa* and *B. oleracea*. Theor. Appl. Genet. 120(1): 31–43. 10.1007/s00122-009-1157-4 19784615

[bib41] Jaffrézic F. Pletcher S. D., 2000 Statistical Models for Estimating the Genetic Basis of Repeated Measures and Other Function-Valued Traits. Genetics 156: 913–922. 11014836 10.1093/genetics/156.2.913 PMC1461268

[bib42] Jannink J.-L. Lorenz A. J. Iwata H., 2010 Genomic selection in plant breeding: from theory to practice. Brief. Funct. Genomics 9(2): 166–177. 10.1093/bfgp/elq001 20156985

[bib43] Jiang L. Clavijo J. A. Sun L. Zhu X. Bhakta M. S., 2015 Plastic expression of heterochrony quantitative trait loci (hQTLs) for leaf growth in the common bean (*Phaseolus vulgaris*). New Phytol. 207(3): 872–882. 10.1111/nph.13386 25816915 PMC6681149

[bib44] Kang M. S., 1997 Using Genotype-by-Environment Interaction for Crop Cultivar Development. Adv. Agron. 62: 199–252. 10.1016/S0065-2113(08)60569-6

[bib45] Kell D. B., 2002 Genotype–phenotype mapping: genes as computer programs. Trends Genet. 18(11): 555–559. 10.1016/S0168-9525(02)02765-8 12414184

[bib46] Kingsolver J. Gomulkiewicz R. Carter P. A., 2001 Variation, selection and evolution of function-valued traits, pp. 87–104 in *Microevolution Rate*, *Pattern*, *Process*, edited by Hendry A. P. Kinnison M. T. Springer, Netherlands 10.1007/978-94-010-0585-2_7 11838789

[bib47] Kruschke J. K., 2015 *Doing Bayesian Data Analysis: A Tutorial with R*, *BUGS*, *and Stan*, Academic Press, San Diego, CA.

[bib48] Kuznetsova, A., P. B. Brockhoff, and C. R. H. Bojesen, 2015 lmerTest: Tests in linear mixed effects models. R package version 2.0–25. https://CRAN.R-project.org/package=lmerTest, pp.

[bib49] Markelz, R. J. C., M. F. Covington, M. T. Brock, U. K. Devisetty, D. J. Kliebenstein *et al.*, 2017 Using RNA-seq for Genomic Scaffold Placement, Correcting Assemblies, and Genetic Map Creation in a Common *Brassica rapa* Mapping Population. G3: Genes|Genomes|Genetics. DOI: 10.1534/g3.117.043000 PMC5499133 28546385

[bib50] Merzlyak M. N. Gitelson A. A. Chivkunova O. B. Rakitin V. Y. U., 1999 Non-destructive optical detection of pigment changes during leaf senescence and fruit ripening. Physiol. Plant. 106(1): 135–141. 10.1034/j.1399-3054.1999.106119.x

[bib51] Moczek A. P. Sears K. E. Stollewerk A. Wittkopp P. J. Diggle P., 2015 The significance and scope of evolutionary developmental biology: a vision for the 21st century. Evol. Dev. 17(3): 198–219. 10.1111/ede.12125 25963198

[bib52] Moose S. P. Mumm R. H., 2008 Molecular Plant Breeding as the Foundation for 21st Century Crop Improvement. Plant Physiol. 147(3): 969–977. 10.1104/pp.108.118232 18612074 PMC2442525

[bib53] Ober U. Ayroles J. F. Stone E. A. Richards S. Zhu D., 2012 Using Whole-Genome Sequence Data to Predict Quantitative Trait Phenotypes in *Drosophila melanogaster*. PLoS Genet. 8(5): e1002685 10.1371/journal.pgen.1002685 22570636 PMC3342952

[bib54] Onogi A. Watanabe M. Mochizuki T. Hayashi T. Nakagawa H., 2016 Toward integration of genomic selection with crop modelling: the development of an integrated approach to predicting rice heading dates. Theor. Appl. Genet. 129(4): 805–817. 10.1007/s00122-016-2667-5 26791836

[bib55] Pace J. Yu X. Lübberstedt T., 2015 Genomic prediction of seedling root length in maize (*Zea mays* L.). Plant J. 83(5): 903–912. 10.1111/tpj.12937 26189993

[bib89] Patil A. Huard D. Fonnesbeck C. J., 2010 PyMC: Bayesian Stochastic Modelling in Phython. J. Stat Softw. 34(4): 1–81. PMCID: PMC3097064 EMSID: UKMS31574. PMC3097064 21603108

[bib56] Peñuelas J. Baret F. Filella I., 1995 Semi-empirical indices to assess carotenoids/chlorophyll *a* ratio from leaf spectral reflectance. Photosynthetica 31: 221–230.

[bib57] Peñuelas J. Gamon J. A. Fredeen A. L. Merino J. Field C. B., 1994 Reflectance indices associated with physiological changes in nitrogen- and water-limited sunflower leaves. Remote Sens. Environ. 48(2): 135–146. 10.1016/0034-4257(94)90136-8

[bib58] Penuelas J. Pinol J. Ogaya R. Filella I., 1997 Estimation of plant water concentration by the reflectance Water Index WI (R900/R970). Int. J. Remote Sens. 18(13): 2869–2875. 10.1080/014311697217396

[bib59] Prusinkiewicz P. Erasmus Y. Lane B. Harder L. D. Coen E., 2007 Evolution and Development of Inflorescence Architectures. Science 316(5830): 1452–1456. 10.1126/science.1140429 17525303

[bib60] R core Team, 2015 R: A language and environment for statistical computing, pp., Vienna, Austria.

[bib61] Raines C. Paul M., 2006 Products of leaf primary carbon metabolism modulate the developmental programme determining plant morphology. J. Exp. Bot. 57(9): 1857–1862. 10.1093/jxb/erl011 16714302

[bib62] Reymond M. Muller B. Leonardi A. Charcosset A. Tardieu F., 2003 Combining Quantitative Trait Loci Analysis and an Ecophysiological Model to Analyze the Genetic Variability of the Responses of Maize Leaf Growth to Temperature and Water Deficit. Plant Physiol. 131(2): 664–675. 10.1104/pp.013839 12586890 PMC166842

[bib63] Rice S. H., 1998 The Evolution of Canalization and the Breaking of Von Baer’s Laws: Modeling the Evolution of Development with Epistasis. Evolution 52(3): 647–656. 10.1111/j.1558-5646.1998.tb03690.x 28565257

[bib64] SAS Institute, 2017 *Posterior Predictive Distribution*. SAS Institute, Inc, Cary, NC.

[bib65] Schlichting C. D. Pigliucci M., 1998 *Phenotypic Evolution: A reaction norm perspective*, Sinauer Associates, Inc, Sunderland, MA.

[bib66] Schneidereit J. Häusler R. E. Fiene G. Kaiser W. M. Weber A. P. M., 2006 Antisense repression reveals a crucial role of the plastidic 2-oxoglutarate/malate translocator DiT1 at the interface between carbon and nitrogen metabolism. Plant J. 45(2): 206–224. 10.1111/j.1365-313X.2005.02594.x 16367965

[bib67] Stinchcombe J. R. Izem R. Heschel M. S. McGoey B. V. Schmitt J., 2010 Across-environment genetic correlations and the frequency of selective environments shape the evolutionary dynamics of growth rate in *Impatiens capensis*. Evolution 64: 2887–2903. DOI: 10.1111/j.1558-5646.2010.01060.x 20662920

[bib68] Stinchcombe J. R. Kirkpatrick M., 2012 Genetics and evolution of function-valued traits: understanding environmentally responsive phenotypes. Trends Ecol. Evol. 27(11): 637–647. 10.1016/j.tree.2012.07.002 22898151

[bib69] Sugiyama S.-i. Gotoh M., 2010 How meristem plasticity in response to soil nutrients and light affects plant growth in four Festuca grass species. New Phytol. 185(3): 747–758. 10.1111/j.1469-8137.2009.03090.x 19925556

[bib70] Tardieu F. Reymond M. Muller B. Granier C. Simonneau T., 2005 Linking physiological and genetic analyses of the control of leaf growth under changing environmental conditions. Aust. J. Agric. Res. 56(9): 937–946. 10.1071/AR05156

[bib71] Technow F. Messina C. D. Totir L. R. Cooper M., 2015 Integrating Crop Growth Models with Whole Genome Prediction through Approximate Bayesian Computation. PLoS One 10(6): e0130855 10.1371/journal.pone.0130855 26121133 PMC4488317

[bib72] Tsukaya, H., 2005 Leaf shape: genetic controls and environmental factors. the International Journal of Developmental Biology 49: 547–555. 10.1387/ijdb.041921ht 16096964

[bib73] van Eeuwijk F. A. Bink M. C. A. M. Chenu K. Chapman S. C., 2010 Detection and use of QTL for complex traits in multiple environments. Curr. Opin. Plant Biol. 13(2): 193–205. 10.1016/j.pbi.2010.01.001 20137999

[bib74] Vigil M. F. Anderson R. L. Beard W. E., 1997 Base temperature and growing-degree-hour requirements for the emergence of canola. Crop Sci. 37(3): 844–849. 10.2135/cropsci1997.0011183X003700030025x

[bib75] Voorrips R. E., 2002 MapChart: Software for the Graphical Presentation of Linkage Maps and QTLs. J. Hered. 93(1): 77–78. 10.1093/jhered/93.1.77 12011185

[bib76] Waddington C. H., 1954 The integration of gene-controlled processes and its bearing on evolution. Proceedings of the 9th Internatinal Congress of Genetics 9: 232–245.

[bib77] Weber V. S. Araus J. L. Cairns J. E. Sanchez C. Melchinger A. E., 2012 Prediction of grain yield using reflectance spectra of canopy and leaves in maize plants grown under different water regimes. Field Crops Res. 128: 82–90. 10.1016/j.fcr.2011.12.016

[bib78] Weinig C. Delph L. F., 2001 Phenotypic plasticity early in life constrains developmental responses later. Evolution 55(5): 930–936. 10.1554/0014-3820(2001)055[0930:PPEILC]2.0.CO;2 11430653

[bib79] Wright S. D. McConnaughay K. D. M., 2002 Interpreting phenotypic plasticity: the importance of ontogeny. Plant Species Biol. 17(2-3): 119–131. 10.1046/j.1442-1984.2002.00082.x

[bib80] Wu R. Lin M., 2006 Functional mapping — how to map and study the genetic architecture of dynamic complex traits. Nat. Rev. Genet. 7(3): 229–237. 10.1038/nrg1804 16485021

[bib81] Wu R. Ma C.-X. Lin M. Casella G., 2004 A General Framework for Analyzing the Genetic Architecture of Developmental Characteristics. Genetics 166(3): 1541–1551. 10.1534/genetics.166.3.1541 15082567 PMC1470782

[bib82] Xu L. Henke M. Zhu J. Kurth W. Buck-Sorlin G., 2011 A functional–structural model of rice linking quantitative genetic information with morphological development and physiological processes. Ann. Bot. (Lond.) 107(5): 817–828. 10.1093/aob/mcq264 PMC3077984 21247905

[bib83] Yang J. Zhu J., 2005 Methods for predicting superior genotypes under multiple environments based on QTL effects. Theor. Appl. Genet. 110(7): 1268–1274. 10.1007/s00122-005-1963-2 15806347

[bib84] Yin X. Chasalow S. D. Dourleijn C. J. Stam P. Kropff M. J., 2000 Coupling estimated effects of QTLs for physiological traits to a crop growth model: predicting yield variation among recombinant inbred lines in barley. Heredity 85(6): 539–549. 10.1046/j.1365-2540.2000.00790.x 11240620

[bib85] Yin X. Struik P. C. Kropff M. J., 2004 Role of crop physiology in predicting gene-to-phenotype relationships. Trends Plant Sci. 9(9): 426–432. 10.1016/j.tplants.2004.07.007 15337492

[bib86] Zhang M. Bo W. Xu F. Li H. Ye M., 2017 The genetic architecture of shoot–root covariation during seedling emergence of a desert tree, Populus euphratica. Plant J. 90(5): 918–928. 10.1111/tpj.13518 28244225

[bib87] Zhang X. Perez-Rodriguez P. Semagn K. Beyene Y. Babu R., 2015 Genomic prediction in biparental tropical maize populations in water-stressed and well-watered environments using low-density and GBS SNPs. Heredity 114(3): 291–299. 10.1038/hdy.2014.99 25407079 PMC4815582

