# Supplementary material for: Mapping and Predicting Non-Linear Brassica rapa Growth Phenotypes Based on Bayesian and Frequentist Complex Trait Estimation
Source: G3 (Bethesda). 2018 Feb 26;8(4):1247–58. doi: 10.1534/g3.117.300350 (PMC5873914; doi:10.1534/g3.117.300350)
Supplement: Supplementary file 3 [file 1247FileS3.docx]

Table S1. Location, LOD scores, and percent variance explained in *Brassica rapa* RILs for additive QTLs

| **Trait** | **Treat** | **Ye-ar** | **N** | **LOD** | **Var**  **(%) and direction (-/+)** | **Chrom-osome** | **Start marker** | **Start position** | **QTL marker** | **QTL position** | **End marker** | **End position** |
| --- | --- | --- | --- | --- | --- | --- | --- | --- | --- | --- | --- | --- |
| **Leaf Traits** | | |  |  |  |  |  |  |  |  |  |  |
| LL_d | CR | 11 | 114 | 3.46 | -13.06 | 10 | A10x3102185 | 1.198926583 | A10x2044606 | 12.82602422 | A10x1623273 | 17.75438048 |
| LL_d† | CR | 12 | 119 | 4.43 | +15.75 | 2 | c2.loc60 | 60 | A02x11484559 | 73.13388355 | c2.loc83 | 83 |
| LL_d* | CR | 12 | 119 | 4.65 | +16.47 | 6 | A06x20238371 | 52.53387666 | A06x20713866 | 54.33875139 | A06x20857317 | 57.64683012 |
| LL_d* | UN | 12 | 119 | 3.38 | +12.25 | 6 | c6.loc5 | 5 | A06x16894473 | 42.70732634 | A06x21659075 | 65.87746433 |
| LL_iD | CR | 11 | 114 | 3.50 | -13.18 | 10 | A10x3102185 | 1.198926583 | A10x2741921 | 3.270837966 | A10x1623273 | 17.75438048 |
| LL_iD | UN | 11 | 114 | 5.96 | +15.46 | 2 | A02x10165707 | 67.33734309 | A02x11746307 | 74.39008961 | A02x13567707 | 78.95126166 |
| LL_iD | UN | 11 | 114 | 4.42 | -11.11 | 3 | A03x16016626 | 83.48353292 | A03x16921981 | 89.68889962 | A03x17891230 | 92.9851068 |
| LL_iD | UN | 11 | 114 | 4.47 | -11.24 | 4 | c4.loc51 | 51 | A04x15617584 | 58.28542156 | A04x16182858 | 63.2607492 |
| LL_iD | UN | 11 | 114 | 3.29 | -8.07 | 10 | A10x3289895 | 0 | A10x2471393 | 7.46867342 | c10.loc12 | 12 |
| LL_iD | CR | 12 | 114 | 7.19 | +23.01 | 2 | A02x9341556 | 64.48281921 | A02x11484559 | 73.13388355 | A02x12745444 | 76.88008977 |
| LL_iD | CR | 12 | 114 | 3.36 | -9.92 | 3 | A03x13531256 | 69.45129618 | A03x15983737 | 83.07557336 | A03x19572534 | 102.0679349 |
| LL_iD | UN | 12 | 114 | 4.05 | +13.13 | 2 | A02x9341556 | 64.48281921 | A02x10165707 | 67.33734309 | A02x12745444 | 76.88008977 |
| LL_iD | UN | 12 | 114 | 3.49 | -11.18 | 3 | A03x14585658 | 76.45189043 | A03x16016626 | 83.48353292 | A03x17891230 | 92.9851068 |
| LL_Lmax | CR | 11 | 114 | 4.41 | +16.33 | 6 | A06x5123487 | 9.120281056 | A06x5980083 | 14.82887427 | c6.loc30 | 30 |
| LL_Lmax | UN | 11 | 114 | 5.05 | +15.86 | 1 | A01x24975652 | 78.44073289 | A01x25154492 | 80.95269655 | A01x25419156 | 82.59508111 |
| LL_Lmax | UN | 11 | 114 | 4.50 | +13.97 | 6 | A06x3966070 | 2.025785201 | A06x4290899 | 6.243892076 | c6.loc56 | 56 |
| LL_Lmax† | CR | 12 | 119 | 4.43 | +15.75 | 2 | c2.loc60 | 60 | A02x11484559 | 73.13388355 | c2.loc83 | 83 |
| LL_Lmax† | CR | 12 | 119 | 4.65 | +16.48 | 6 | A06x20238371 | 52.53387666 | A06x20713866 | 54.33875139 | A06x20857317 | 57.64683012 |
| LL_Lmax* | UN | 12 | 119 | 3.38 | +12.25 | 6 | c6.loc5 | 5 | A06x16894473 | 42.70732634 | A06x21659075 | 65.87746433 |
| LW_d* | CR | 12 | 119 | 4.85 | +17.12 | 2 | A01x8029418 | 27.28663362 | A01x8409488 | 30.50529205 | A01x9047566 | 33.39309516 |
| LW_d* | CR | 12 | 119 | 4.67 | +16.54 | 3 | c2.loc60 | 60 | A02x11484559 | 73.13388355 | A02x11746307 | 74.39008961 |
| LW_d* | CR | 12 | 119 | 5.11 | -17.94 | 7 | A03x14585658 | 76.45189043 | A03x17428794 | 92.16876707 | A03x19572534 | 102.0679349 |
| LW_d* | CR | 12 | 119 | 5.66 | +19.68 | 1 | A06x19065113 | 49.25839089 | A06x20713866 | 54.33875139 | A06x20857317 | 57.64683012 |
| LW_d* | CR | 12 | 119 | 4.32 | +15.41 | 6 | A07x13241485 | 38.1564765 | A07x16094553 | 46.90648841 | A07x16383905 | 48.95769519 |
| LW_d* | CR | 12 | 119 | 3.52 | -12.72 | 10 | A10x2285628 | 9.519459583 | A10x7550539 | 33.91770504 | c10.loc35 | 35 |
| LW_d* | UN | 12 | 119 | 10 | +32.10 | 1 | A01x8057967 | 29.14378319 | A01x8348377 | 30.05156394 | A01x8479578 | 31.33196221 |
| LW_d† | UN | 12 | 119 | 9.48 | +30.71 | 3 | A02x9558103 | 66.51043559 | A02x10819082 | 70.21417413 | A02x22120337 | 90.51428166 |
| LW_d* | UN | 12 | 119 | 7.77 | +25.95 | 7 | A03x10877209 | 59.5732028 | A03x11791866 | 62.43972759 | A03x12997092 | 67.80869053 |
| LW_d* | UN | 12 | 119 | 4.82 | -17.00 | 2 | A03x17151125 | 90.50481525 | A03x17233425 | 91.76102131 | A03x17945633 | 93.39327667 |
| LW_d* | UN | 12 | 119 | 6.41 | +21.98 | 10 | c6.loc40 | 40 | A06x17027456 | 43.11549621 | A06x20857317 | 57.64683012 |
| LW_d* | UN | 12 | 119 | 4.89 | +17.23 | 6 | A07x121679 | 0 | A07x385264 | 1.208778824 | c7.loc3 | 3 |
| LW_d* | UN | 12 | 119 | 3.24 | -11.76 | 3 | A10x7124005 | 31.8768557 | A10x7420243 | 33.1013653 | A10x7761819 | 34.3258749 |
| LW_iD | CR | 11 | 114 | 3.26 | -12.35 | 10 | A10x3289895 | 0 | A10x2044606 | 12.82602422 | c10.loc35 | 35 |
| LW_iD | UN | 11 | 114 | 4.22 | +13.52 | 2 | A02x9558103 | 66.51043559 | A02x11746307 | 74.39008961 | A02x19548615 | 84.69703268 |
| LW_ID | UN | 11 | 114 | 3.68 | -11.69 | 10 | A10x3289895 | 0 | A10x2471393 | 7.46867342 | A10x1631569 | 16.92780686 |
| LW_iD | CR | 12 | 114 | 8.57 | +27.01 | 2 | A02x10812958 | 69.38791895 | A02x11484559 | 73.13388355 | A02x11746307 | 74.39008961 |
| LW_iD | CR | 12 | 114 | 3.40 | -9.63 | 3 | A03x14164882 | 73.15566946 | A03x16016626 | 83.48353292 | A03x18787974 | 98.78207137 |
| LW_iD | UN | 12 | 115 | 4.21 | -13.12 | 3 | A03x16497890 | 86.3603748 | A03x16763491 | 88.05686162 | A03x18279509 | 96.7317272 |
| LW_iD | UN | 12 | 115 | 4.00 | -12.40 | 10 | c10.loc95 | 95 | A10x15869982 | 98.00763303 | A10x16318399 | 101.2477804 |
| LW_Lmax | CR | 11 | 114 | 3.12 | +11.83 | 2 | A02x106635 | 0 | A02x11182956 | 71.91043952 | A02x22788955 | 94.67833362 |
| LW_Lmax | UN | 11 | 114 | 4.43 | +16.38 | 1 | A01x25071323 | 79.25643116 | c1.loc81 | 81 | A01x25419156 | 82.59508111 |
| LW_Lmax† | CR | 12 | 119 | 4.86 | +17.12 | 2 | A01x8029418 | 27.28663362 | A01x8409488 | 30.50529205 | A01x9047566 | 33.39309516 |
| LW_Lmax† | CR | 12 | 119 | 4.67 | +16.54 | 3 | c2.loc60 | 60 | A02x11484559 | 73.13388355 | A02x11746307 | 74.39008961 |
| LW_Lmax* | CR | 12 | 119 | 5.11 | -17.94 | 7 | A03x14585658 | 76.45189043 | A03x17428794 | 92.16876707 | A03x19572534 | 102.0679349 |
| LW_Lmax* | CR | 12 | 119 | 5.66 | +19.68 | 1 | A06x19065113 | 49.25839089 | A06x20713866 | 54.33875139 | A06x20857317 | 57.64683012 |
| LW_Lmax* | CR | 12 | 119 | 4.33 | +15.41 | 6 | A07x13241485 | 38.1564765 | A07x16094553 | 46.90648841 | A07x16383905 | 48.95769519 |
| LW_Lmax* | CR | 12 | 119 | 3.52 | -12.72 | 10 | A10x2285628 | 9.519459583 | A10x7550539 | 33.91770504 | c10.loc35 | 35 |
| LW_Lmax† | UN | 12 | 119 | 7.88 | +26.27 | 1 | A01x8029418 | 27.28663362 | A01x8348377 | 30.05156394 | c1.loc34 | 34 |
| LW_Lmax† | UN | 12 | 119 | 7.17 | +24.24 | 3 | A02x9477901 | 66.10247604 | A02x10819082 | 70.21417413 | c2.loc74 | 74 |
| LW_Lmax† | UN | 12 | 119 | 6.68 | -22.78 | 7 | A03x17151125 | 90.50481525 | A03x17636243 | 92.57693694 | A03x18279509 | 96.7317272 |
| LW_Lmax† | UN | 12 | 119 | 5.33 | +18.62 | 2 | A06x14144472 | 36.05639231 | A06x20125742 | 50.9012175 | A06x20857317 | 57.64683012 |
| LW_Lmax† | UN | 12 | 119 | 4.47 | +15.89 | 10 | A07x121679 | 0 | A07x385264 | 1.208778824 | c7.loc6 | 6 |
| LW_Lmax† | UN | 12 | 119 | 4.11 | -14.71 | 6 | A10x7116906 | 31.46889614 | A10x7420243 | 33.1013653 | A10x8155487 | 35.55038451 |
| LW_r | UN | 11 | 114 | 4.35 | +16.13 | 9 | A09x27136204 | 84.04074352 | A09x28366447 | 88.57159302 | A09x31260039 | 103.9861868 |
| LW_r† | CR | 12 | 119 | 3.99 | -14.31 | 2 | c2.loc52 | 52 | A02x12174045 | 75.62410129 | A02x22120337 | 90.51428166 |
| LW_r† | UN | 12 | 119 | 3.48 | +12.59 | 2 | A02x309377 | 1.631432566 | A02x966946 | 7.576571749 | c2.loc68 | 68 |
| **Spectroradiometric indices** | | | |  |  |  |  |  |  |  |  |  |
| mcari1 | UN | 10 | 120 | 3.85 | +13.75 | 9 | A09x8613899 | 54.03182698 | A09x17079326 | 70.48946753 | c9.loc75 | 75 |
| mcari1 | UN | 10 | 120 | 4.62 | +16.26 | 9 | A09x10932468 | 63.10163907 | A09x17079326 | 70.48946753 | c9.loc75 | 75 |
| mtci | UN | 10 | 120 | 4.64 | +12.92 | 1 | A01x26061276 | 89.23236458 | A01x26495518 | 91.30032376 | A01x26761091 | 92.52001138 |
| mtci | UN | 10 | 120 | 4.71 | -13.15 | 3 | A03x118566 | 0.382958487 | A03x14164882 | 73.15566946 | A03x16497890 | 86.3603748 |
| mtci | UN | 10 | 120 | 3.72 | +10.19 | 9 | c9.loc127 | 127 | A09x34851227 | 133.9063516 | A09x38067529 | 148.7607719 |
| sipi2 | UN | 10 | 120 | 3.69 | -13.21 | 1 | A01x8186237 | 29.59767357 | A01x9927004 | 40.36242354 | c1.loc92 | 92 |
| ari1 | UN | 10 | 120 | 3.91 | -11.44 | 1 | c1.loc15 | 15 | A01x8029418 | 27.28663362 | A01x26761091 | 92.52001138 |
| ari1 | UN | 10 | 120 | 5.32 | -16.01 | 9 | A09x11922390 | 65.15220447 | A09x16619967 | 70.89721329 | A09x22639439 | 74.19384458 |
| ari1 | CR | 10 | 120 | 4.78 | -16.75 | 9 | A09x14328090 | 68.44861819 | A09x16619967 | 70.89721329 | c9.loc91 | 91 |
| ari2 | UN | 10 | 120 | 3.59 | -12.87 | 5 | c5.loc35 | 35 | A05x18948334 | 47.86536713 | c5.loc58 | 58 |
| npci | UN | 10 | 120 | 5.13 | -15.55 | 1 | A01x7975940 | 26.83321867 | A01x9511676 | 37.90325798 | A01x12003346 | 44.85229129 |
| npci | UN | 10 | 120 | 3.66 | -10.76 | 9 | c9.loc65 | 65 | c9.loc71 | 71 | A09x28737750 | 89.38793276 |
| npci | CR | 10 | 120 | 5.99 | -18.93 | 1 | A01x9187356 | 35.02556432 | A01x9621651 | 38.31121753 | A01x10719350 | 42.40327209 |
| npci | CR | 10 | 120 | 3.85 | -11.66 | 9 | A09x17079326 | 70.48946753 | A09x27954383 | 87.34750752 | A09x29800389 | 95.57872475 |
| pri2 | UN | 10 | 120 | 4.73 | -14.20 | 1 | A01x7876881 | 26.37927965 | c1.loc32 | 32 | A01x10008696 | 40.77059341 |
| pri2 | UN | 10 | 120 | 4.29 | -12.75 | 9 | A09x11922390 | 65.15220447 | A09x16619967 | 70.89721329 | c9.loc75 | 75 |
| pri2 | CR | 10 | 120 | 6.21 | -18.14 | 1 | A01x9187356 | 35.02556432 | A01x9400632 | 37.08712855 | A01x10144579 | 41.58693236 |
| pri2 | CR | 10 | 120 | 3.41 | -9.42 | 6 | A06x4556763 | 7.060021503 | A06x5582169 | 12.13916317 | A06x7379978 | 22.33104744 |
| pri2 | CR | 10 | 120 | 3.47 | -9.59 | 9 | A09x17690478 | 70.08129766 | A09x28911244 | 90.61244236 | A09x29836334 | 95.98729048 |
| psri | UN | 10 | 120 | 3.46 | -10.94 | 1 | A01x7876881 | 26.37927965 | A01x8682528 | 32.15844197 | A01x26761091 | 92.52001138 |
| psri | UN | 10 | 120 | 3.38 | -10.67 | 9 | A09x11485309 | 64.32572825 | A09x17079326 | 70.48946753 | c9.loc82 | 82 |
| psri | CR | 10 | 120 | 3.73 | -13.34 | 1 | A01x7657237 | 25.56296907 | A01x9400632 | 37.08712855 | c1.loc49 | 49 |
| wi | UN | 10 | 120 | 4.54 | +9.56 | 1 | c1.loc28 | 28 | A01x8348377 | 30.05156394 | A01x8479578 | 31.33196221 |
| wi | UN | 10 | 120 | 5.41 | +11.60 | 1 | c1.loc88 | 88 | A01x25975189 | 88.40951797 | A01x26027978 | 88.82083523 |
| wi | UN | 10 | 120 | 3.51 | +7.26 | 1 | A01x26027978 | 88.82083523 | c1.loc89 | 89 | A01x26061276 | 89.23236458 |
| wi | UN | 10 | 120 | 5.09 | -10.84 | 3 | A03x8812715 | 48.01733245 | A03x8966639 | 49.34747583 | A03x9208458 | 50.22592511 |
| wi | UN | 10 | 120 | 3.21 | +6.58 | 9 | A09x9450643 | 56.51085029 | A09x32285047 | 111.8262005 | c9.loc113 | 113 |
| wi | CR | 10 | 120 | 3.52 | +12.64 | 9 | c9.loc4 | 4 | A09x21954187 | 73.78567471 | A09x25735582 | 82.40827436 |
| mcari1 | UN | 11 | 125 | 6.19 | +20.38 | 9 | c9.loc65 | 65 | A09x16619967 | 70.89721329 | A09x21954187 | 73.78567471 |
| mcari2 | UN | 11 | 125 | 6.43 | +21.10 | 9 | A09x11922390 | 65.15220447 | A09x16619967 | 70.89721329 | A09x21954187 | 73.78567471 |
| mtci | UN | 11 | 125 | 6.32 | +18.65 | 1 | A01x26248761 | 90.05542328 | A01x26649666 | 92.13390709 | A01x26761091 | 92.52001138 |
| mtci | UN | 11 | 125 | 4.79 | -13.73 | 3 | A03x14114548 | 72.7477099 | c3.loc76 | 76 | A03x15289860 | 79.79035169 |
| sipi2 | UN | 11 | 125 | 4.70 | -14.22 | 1 | A01x8479578 | 31.33196221 | A01x26649666 | 92.13390709 | A01x26761091 | 92.52001138 |
| sipi2 | UN | 11 | 125 | 3.36 | -9.92 | 9 | A09x8395309 | 53.62365711 | A09x16619967 | 70.89721329 | A09x21954187 | 73.78567471 |
| tcari | UN | 11 | 125 | 6.57 | +21.49 | 9 | A09x11922390 | 65.15220447 | A09x16619967 | 70.89721329 | A09x21954187 | 73.78567471 |
| ari1 | UN | 11 | 125 | 6.49 | +19.31 | 3 | A03x9208458 | 50.22592511 | A03x10583907 | 57.11403724 | c3.loc59 | 59 |
| ari1 | UN | 11 | 125 | 3.54 | -9.96 | 9 | A09x14328090 | 68.44861819 | A09x17079326 | 70.48946753 | A09x21954187 | 73.78567471 |
| npci | UN | 11 | 125 | 4.95 | -15.19 | 6 | A06x15625710 | 38.19695322 | A06x19335038 | 50.49325794 | c6.loc57 | 57 |
| pri2 | UN | 11 | 125 | 4.95 | -16.67 | 9 | A09x11922390 | 65.15220447 | A09x16619967 | 70.89721329 | A09x21954187 | 73.78567471 |
| psri | UN | 11 | 125 | 4.97 | -16.74 | 9 | c9.loc55 | 55 | A09x16619967 | 70.89721329 | A09x21954187 | 73.78567471 |

* indicates a QTL uniquely identified under Bayesian FVT trait estimation

† indicates a QTL identified under both Bayesian and frequentist trait estimation

Note that only previously published frequentist trait estimates were compared to Bayesian trait estimates: only *r*, *d*, and *Lmax* from 2012.
