# Supplementary material for: Mapping and Predicting Non-Linear Brassica rapa Growth Phenotypes Based on Bayesian and Frequentist Complex Trait Estimation
Source: G3 (Bethesda). 2018 Feb 26;8(4):1247–58. doi: 10.1534/g3.117.300350 (PMC5873914; doi:10.1534/g3.117.300350)
Supplement: Supplementary file 4 [file 1247FileS4.compressed.pdf]

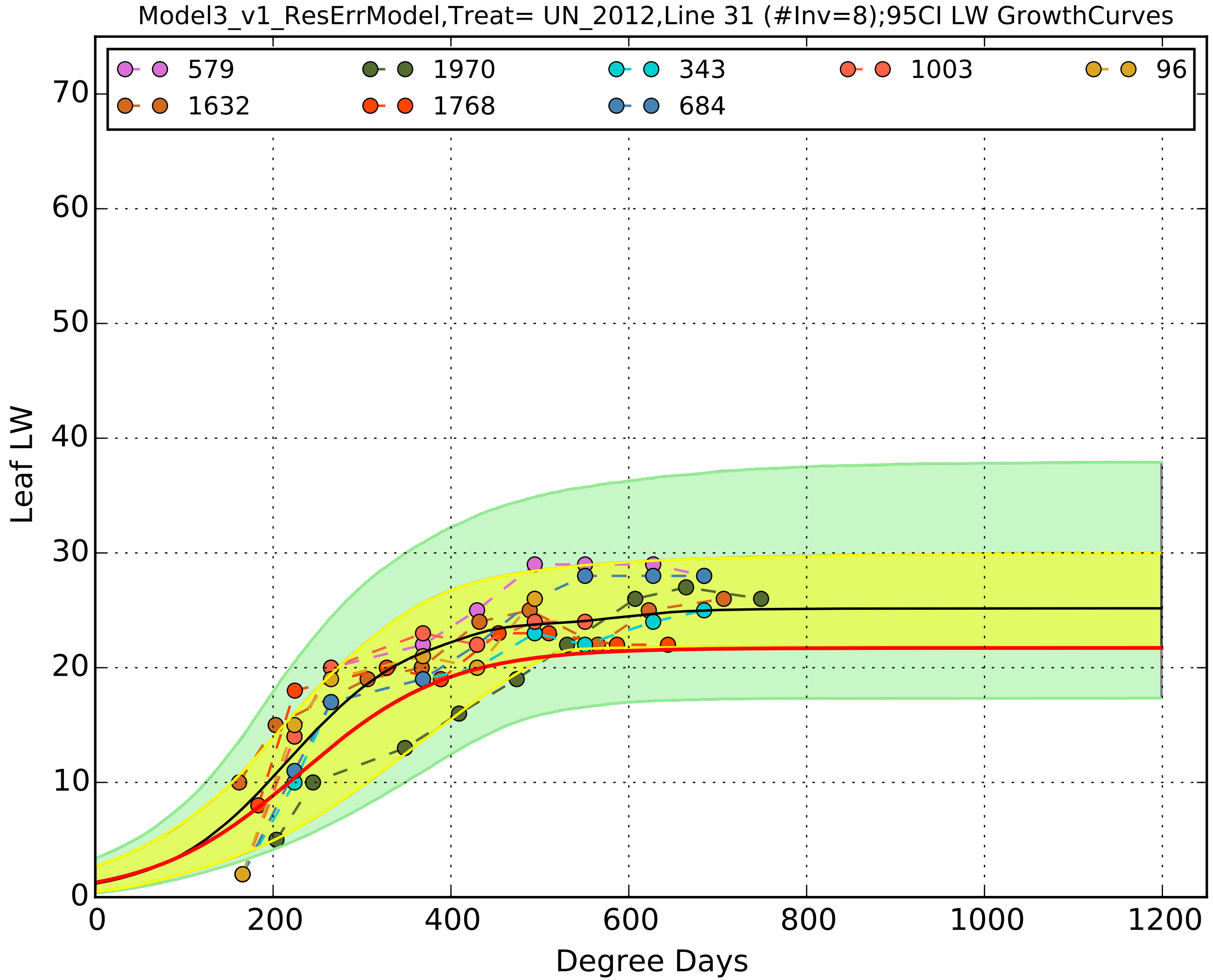

Model3_v1_ResErrModel,Treat= UN_2012,Line 31 (#Inv=8);95CI LW GrowthCurves
579
1970
343
1003
96
1632
1768
684
Leaf LW
Degree Days
0
10
20
30
40
50
60
70
0
200
400
600
800
1000
1200

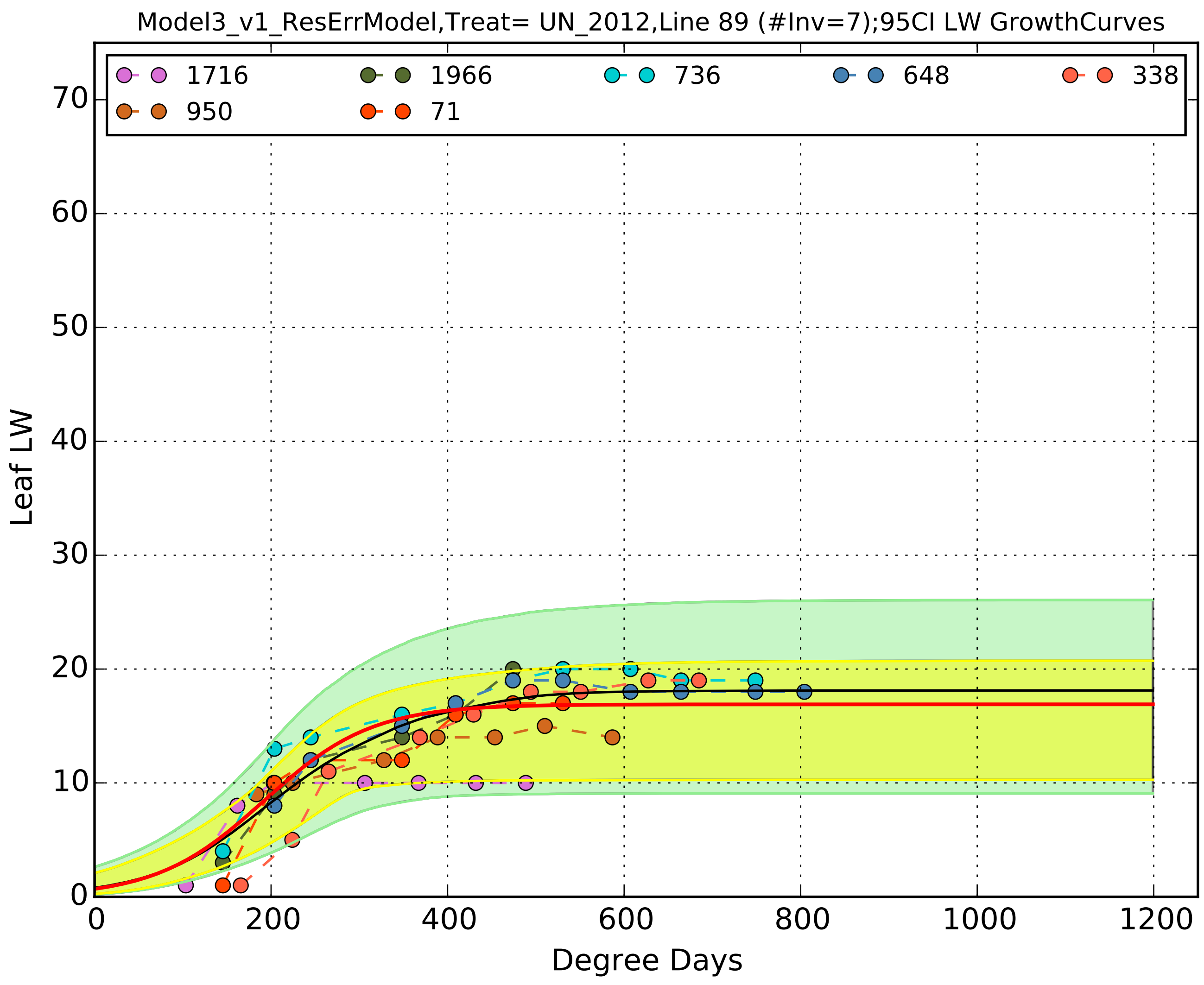

Model3_v1_ResErrModel,Treat= UN_2012,Line 89 (#Inv=7);95CI LW GrowthCurves
1716
1966
736
648
338
950
71
Leaf LW
Degree Days
0
10
20
30
40
50
60
70
0
200
400
600
800
1000
1200

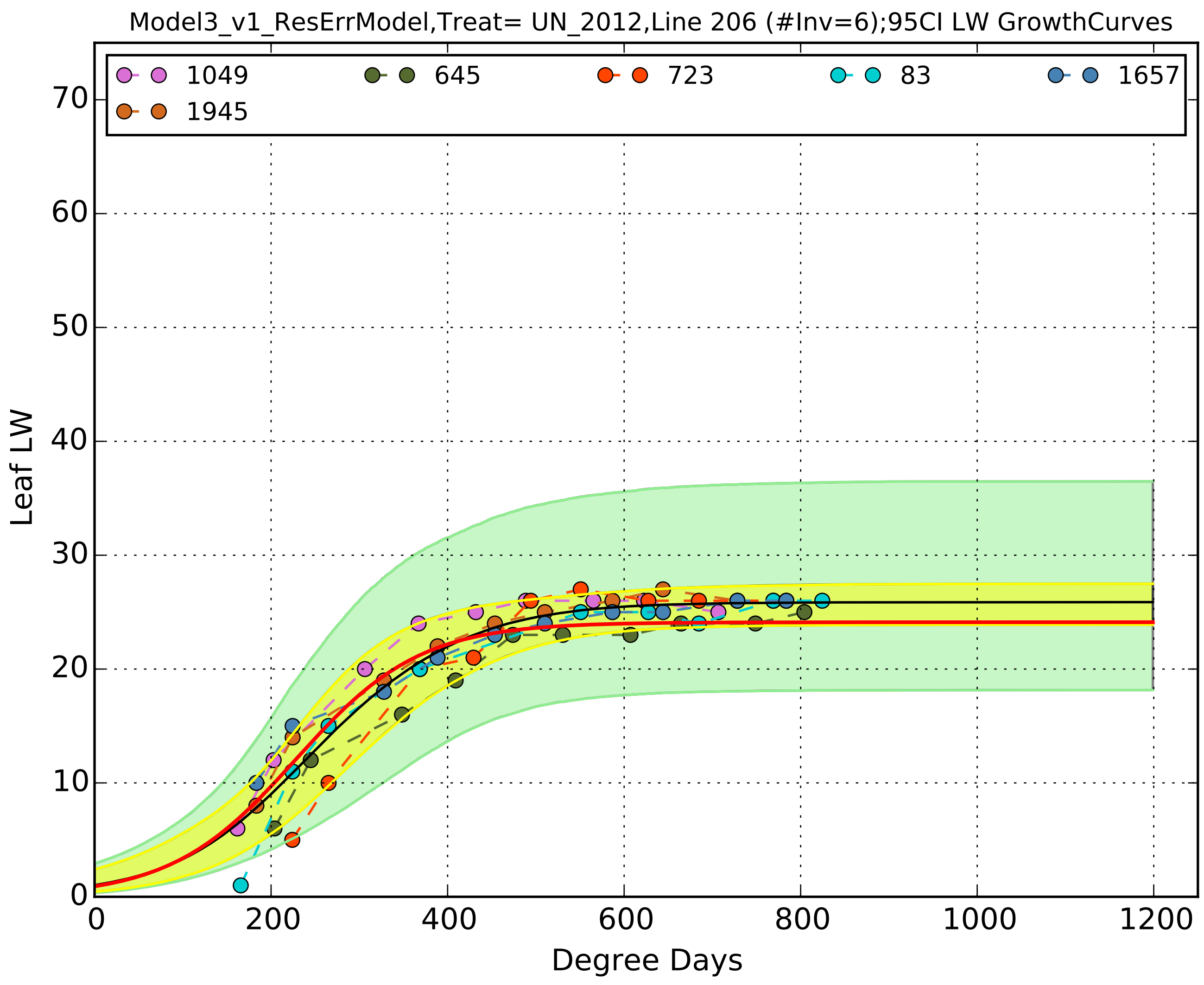

Model3_v1_ResErrModel,Treat= UN_2012,Line 206 (#Inv=6);95CI LW GrowthCurves
1049
645
723
83
1657
1945
Leaf LW
Degree Days
0
10
20
30
40
50
60
70
0
200
400
600
800
1000
1200

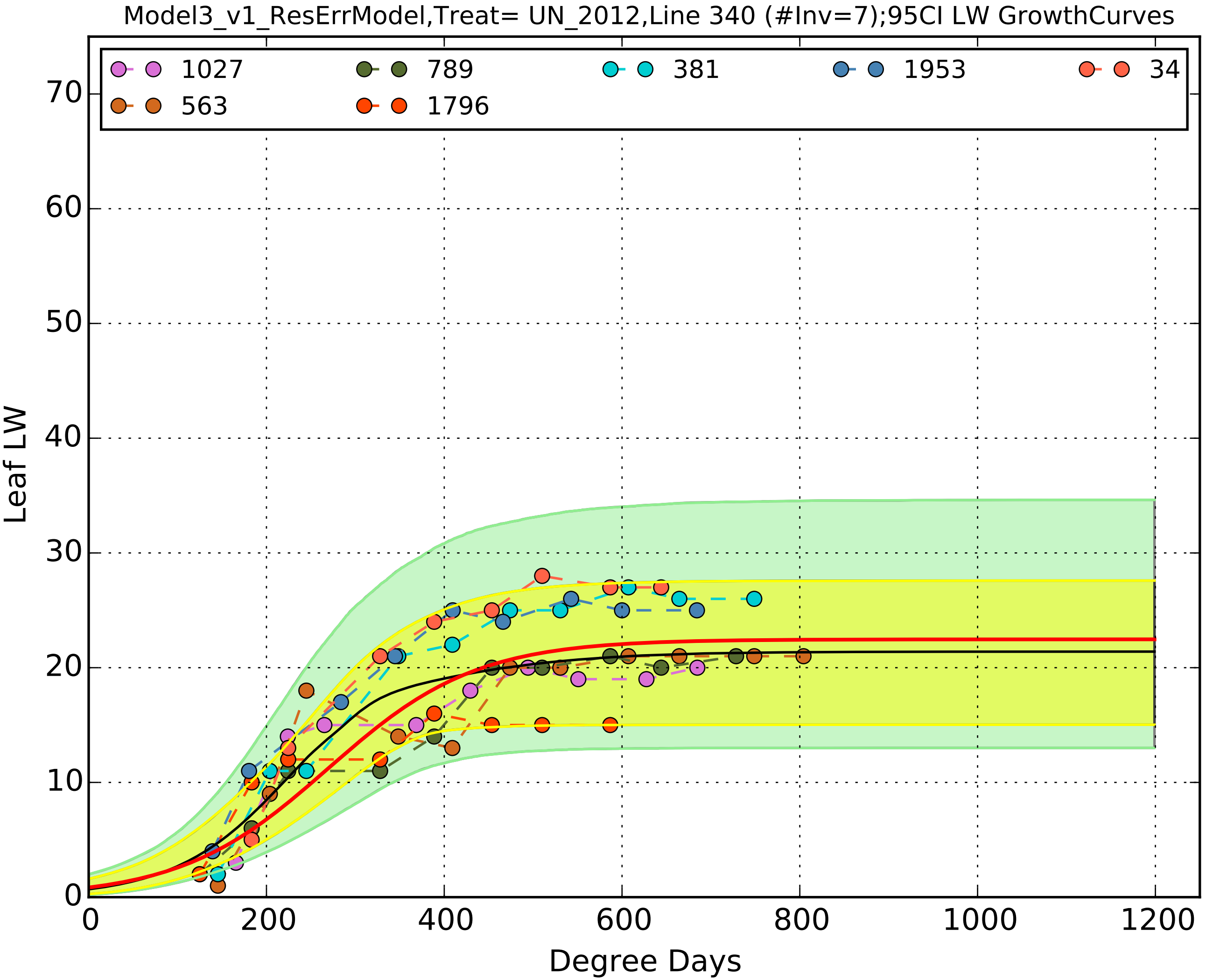
Model3_v1_ResErrModel,Treat= UN_2012,Line 340 (#Inv=7);95CI LW GrowthCurves
1027
789
381
1953
34
563
1796
Leaf LW
Degree Days
0
10
20
30
40
50
60
70
0
200
400
600
800
1000
1200

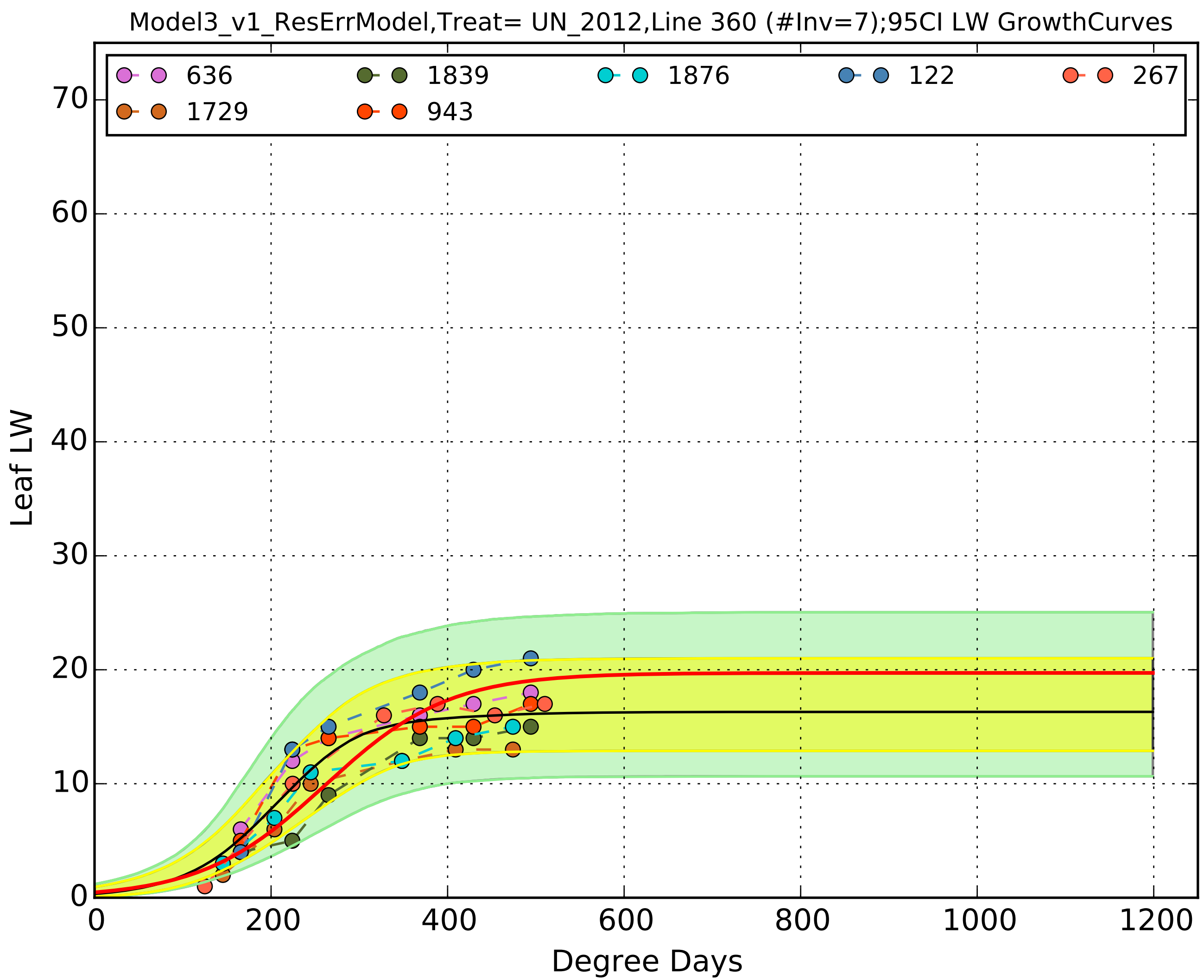
Model3_v1_ResErrModel,Treat= UN_2012,Line 360 (#Inv=7);95CI LW GrowthCurves
636
1839
1876
122
267
1729
943
Leaf LW
Degree Days
0
10
20
30
40
50
60
70
0
200
400
600
800
1000
1200

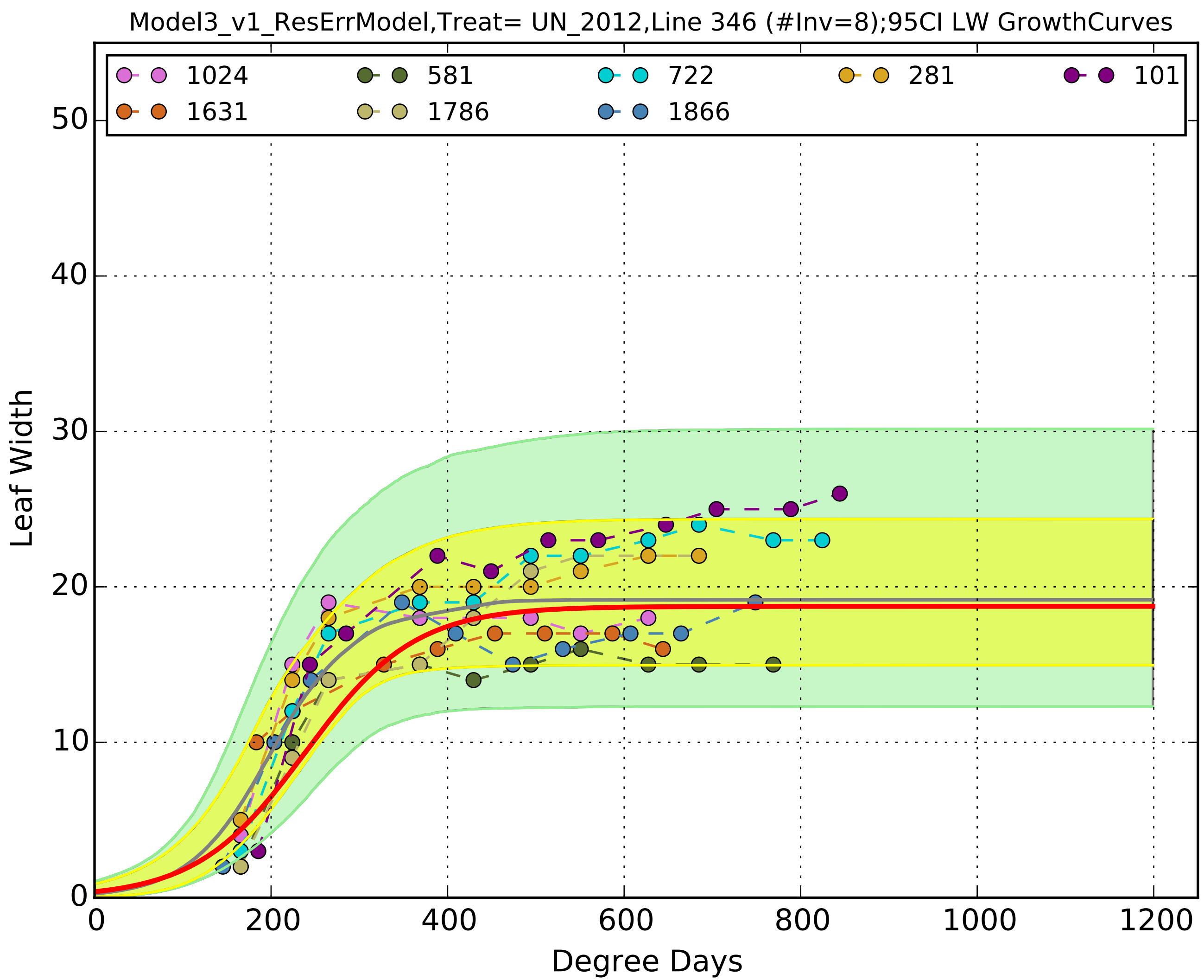

Model3_v1_ResErrModel,Treat= UN_2012,Line 346 (#Inv=8);95CI LW GrowthCurves
1024
581
722
281
101
1631
1786
1866
Leaf Width
Degree Days
0
10
20
30
40
50
0
200
400
600
800
1000
1200

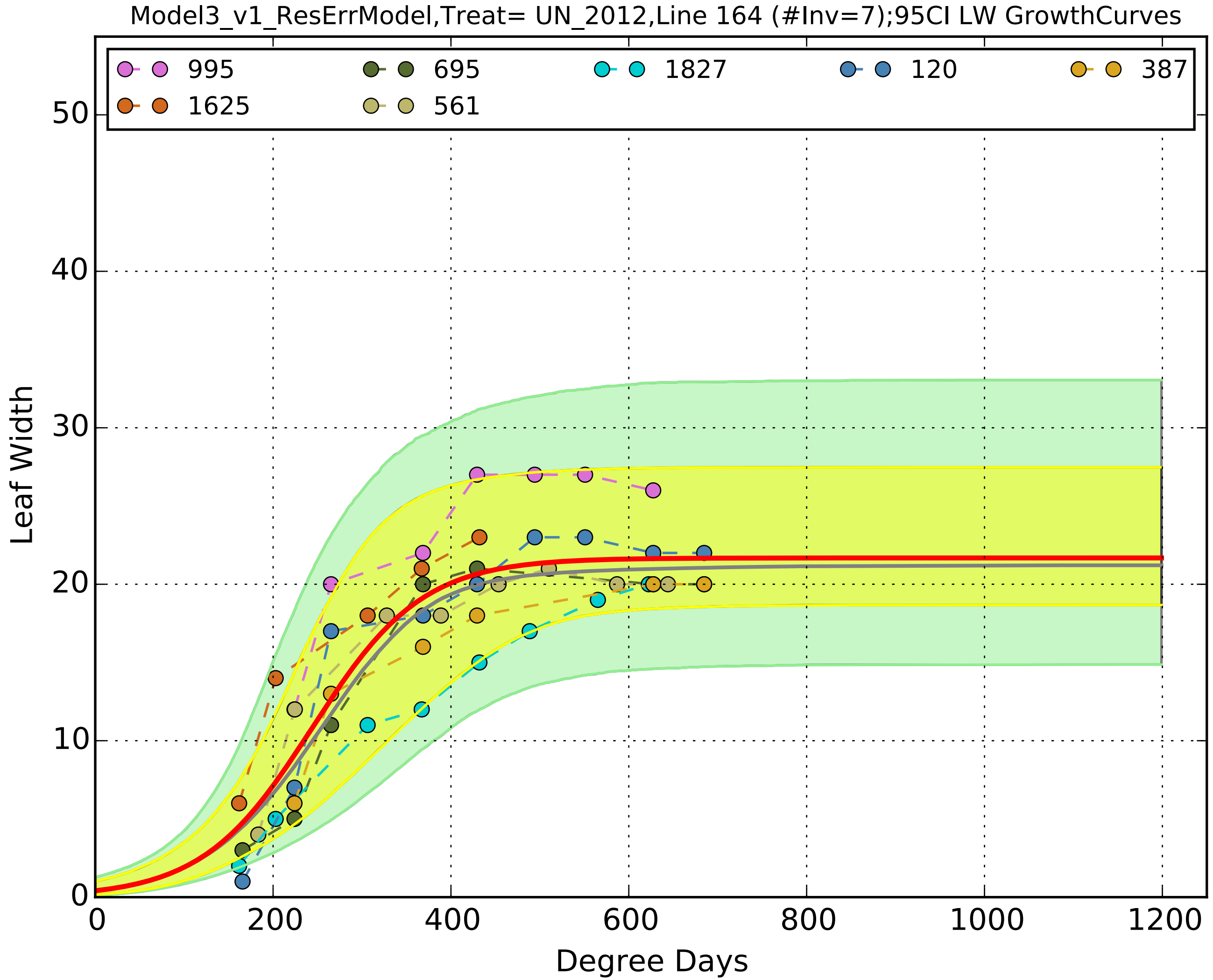

Model3_v1_ResErrModel,Treat= UN_2012,Line 164 (#Inv=7);95CI LW GrowthCurves
995
695
1827
120
387
1625
561
Leaf Width
Degree Days
0
10
20
30
40
50
0
200
400
600
800
1000
1200

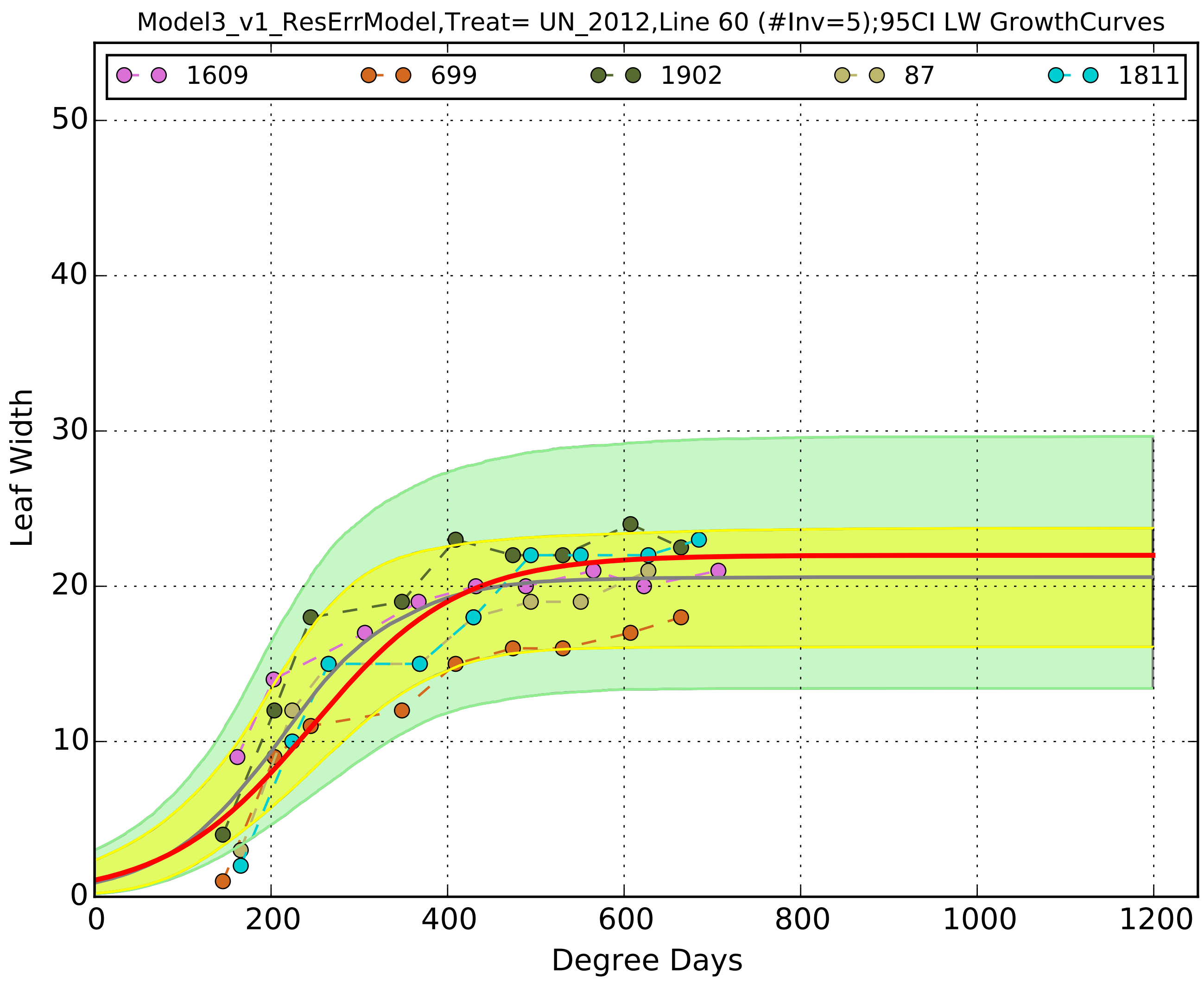

Model3_v1_ResErrModel,Treat= UN_2012,Line 60 (#Inv=5);95CI LW GrowthCurves
1609
699
1902
87
1811
Leaf Width
Degree Days
0
10
20
30
40
50
0
200
400
600
800
1000
1200

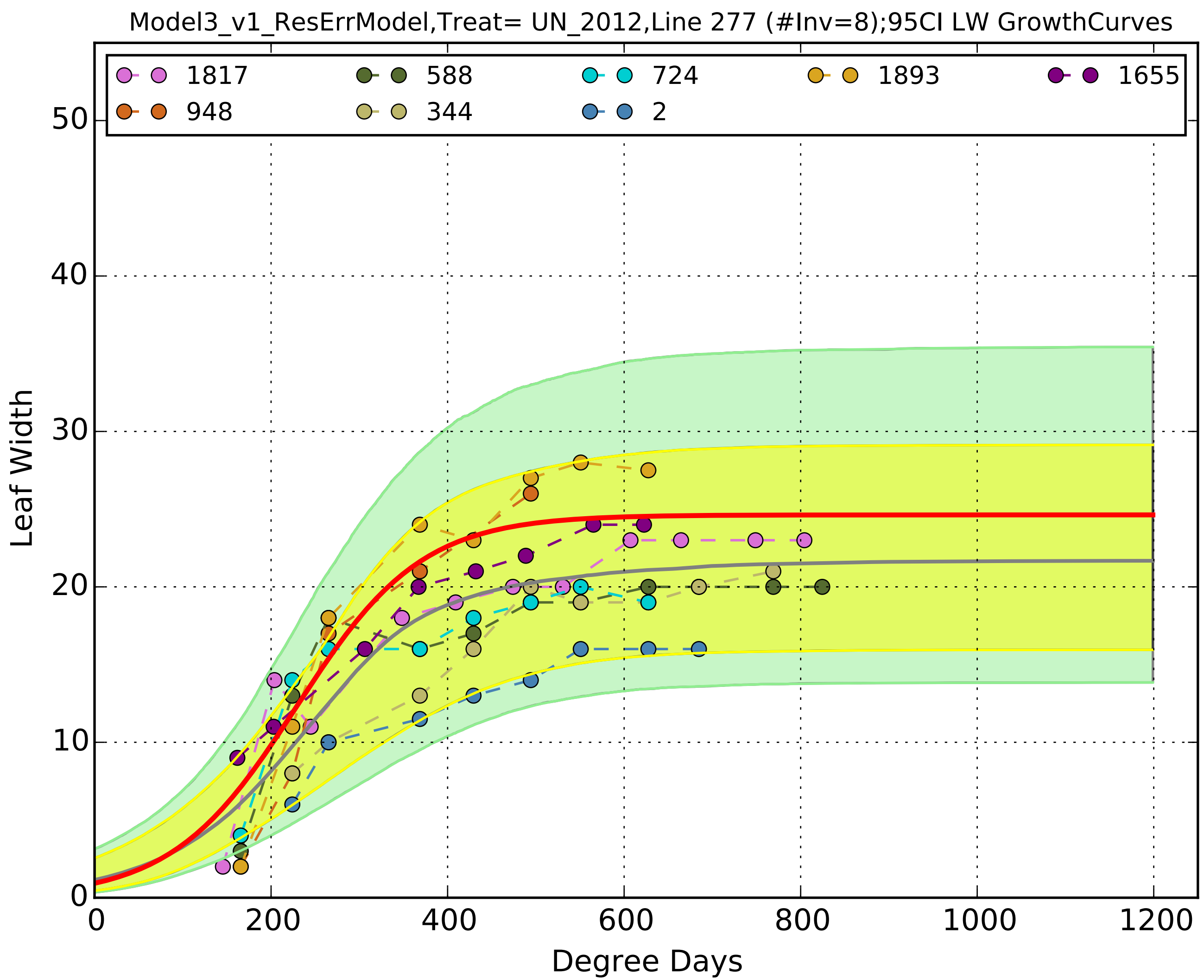
Model3_v1_ResErrModel,Treat= UN_2012,Line 277 (#Inv=8);95CI LW GrowthCurves
1817
588
724
1893
1655
948
344
2
Leaf Width
Degree Days
0
10
20
30
40
50
0
200
400
600
800
1000
1200

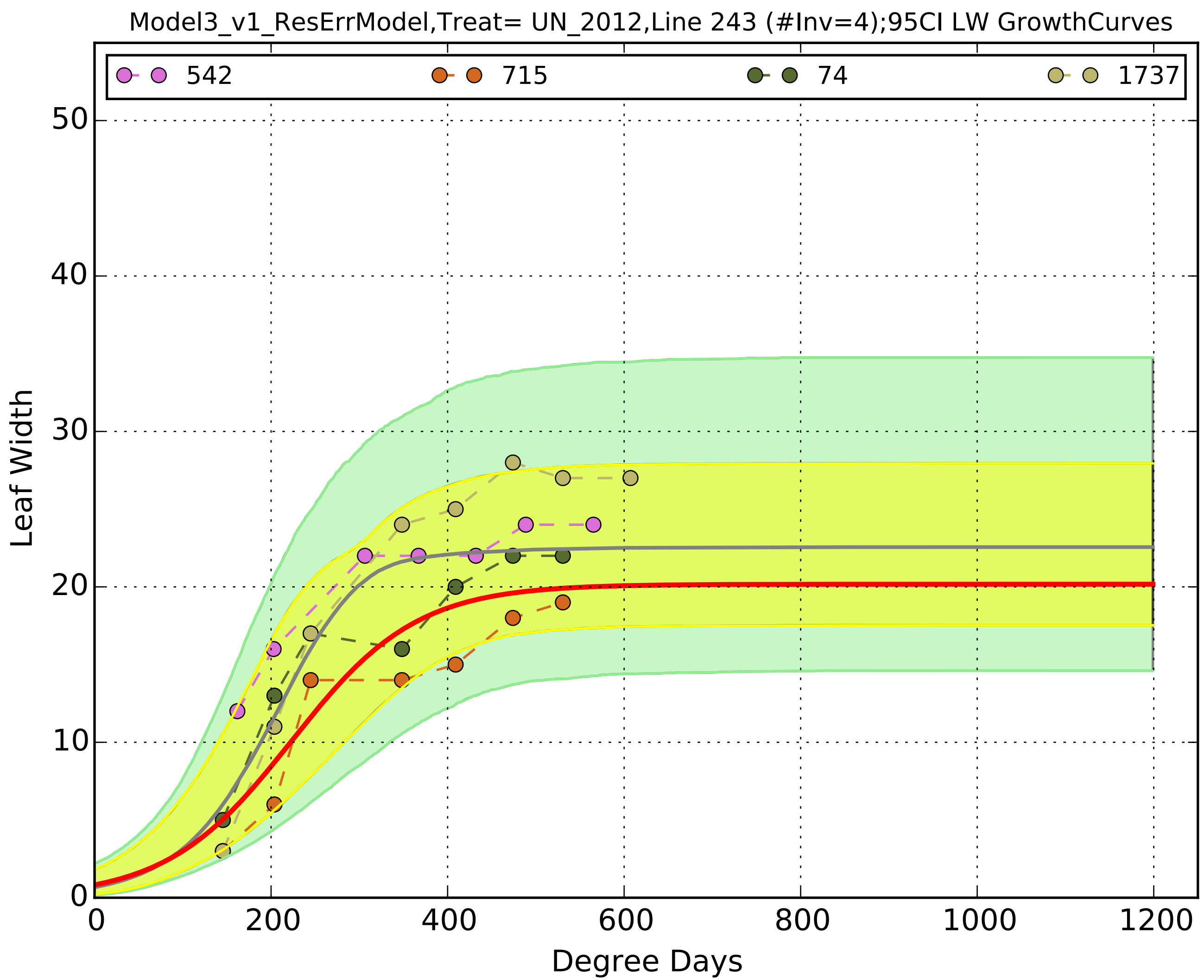
Model3_v1_ResErrModel,Treat= UN_2012,Line 243 (#Inv=4);95CI LW GrowthCurves
542
715
74
1737
Leaf Width
Degree Days
0
10
20
30
40
50
0
200
400
600
800
1000
1200

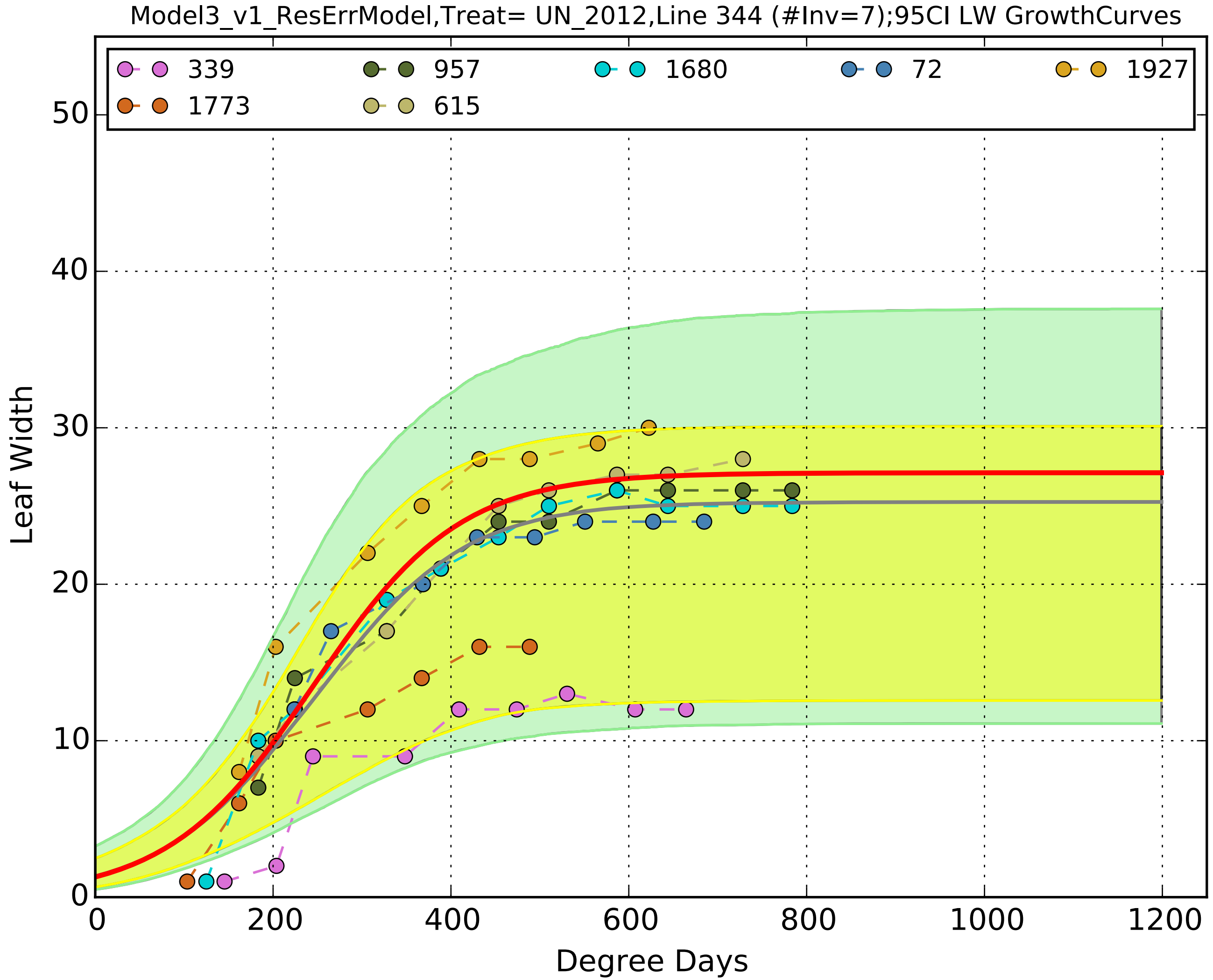
Model3_v1_ResErrModel,Treat= UN_2012,Line 344 (#Inv=7);95CI LW GrowthCurves
339
957
1680
72
1927
1773
615
Leaf Width
Degree Days
0
10
20
30
40
50
0
200
400
600
800
1000
1200

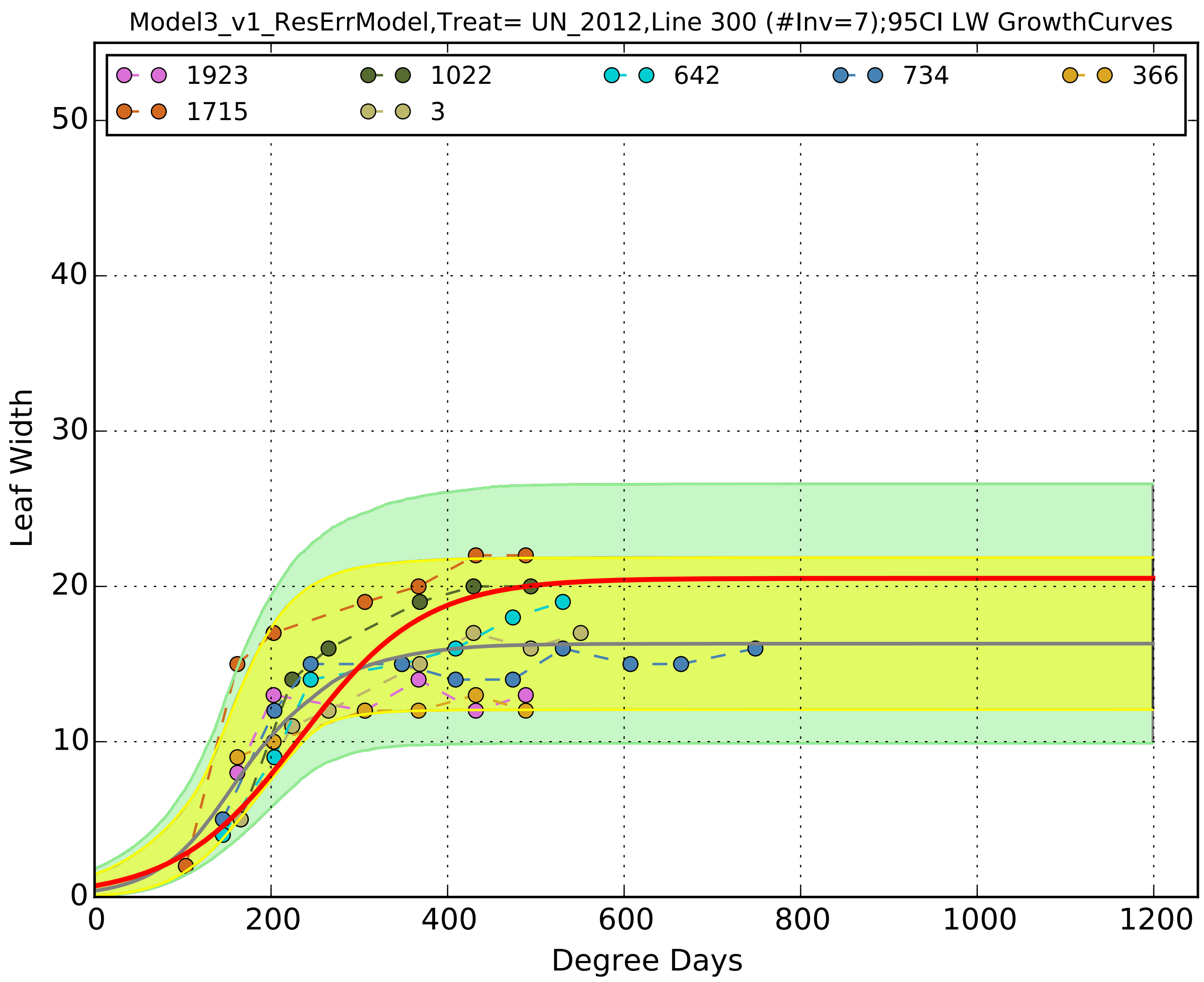

Model3_v1_ResErrModel,Treat= UN_2012,Line 300 (#Inv=7);95CI LW GrowthCurves
1923
1022
642
734
366
1715
3
Leaf Width
Degree Days
0
10
20
30
40
50
0
200
400
600
800
1000
1200

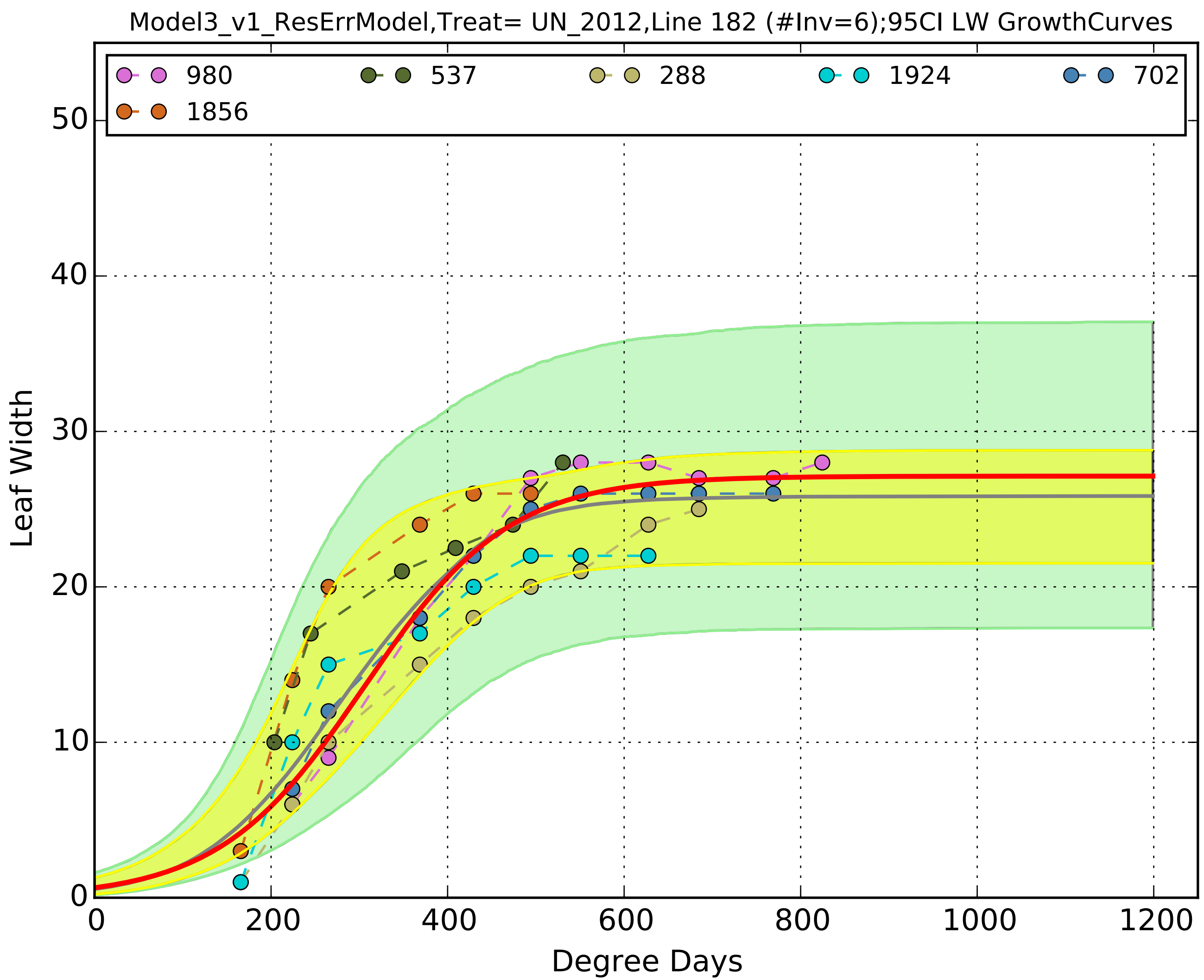

Model3_v1_ResErrModel,Treat= UN_2012,Line 182 (#Inv=6);95CI LW GrowthCurves
980
537
288
1924
702
1856
Leaf Width
Degree Days
0
10
20
30
40
50
0
200
400
600
800
1000
1200

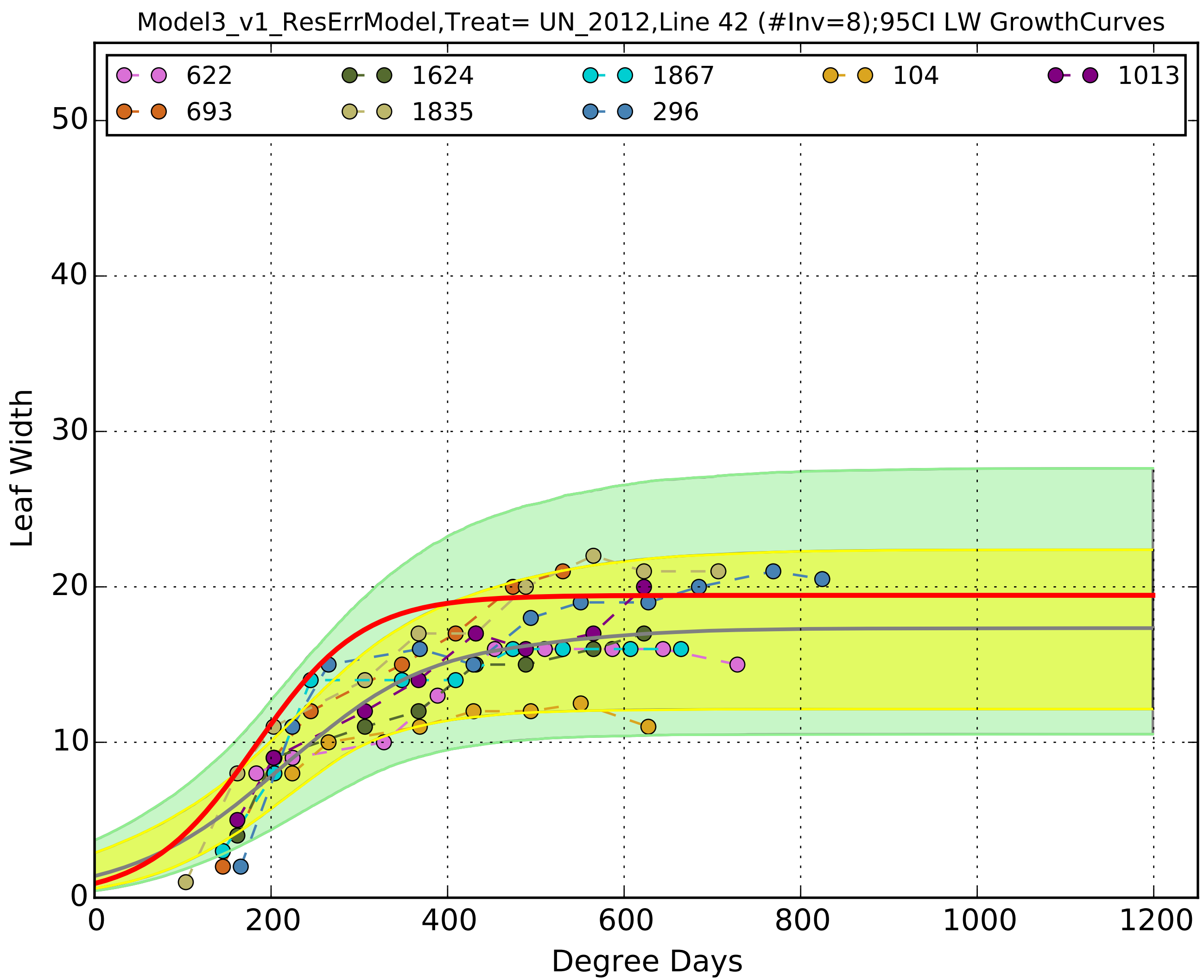

Model3_v1_ResErrModel,Treat= UN_2012,Line 42 (#Inv=8);95CI LW GrowthCurves
622
693
1624
1835
1867
296
104
1013
Leaf Width
Degree Days
0
10
20
30
40
50
0
200
400
600
800
1000
1200

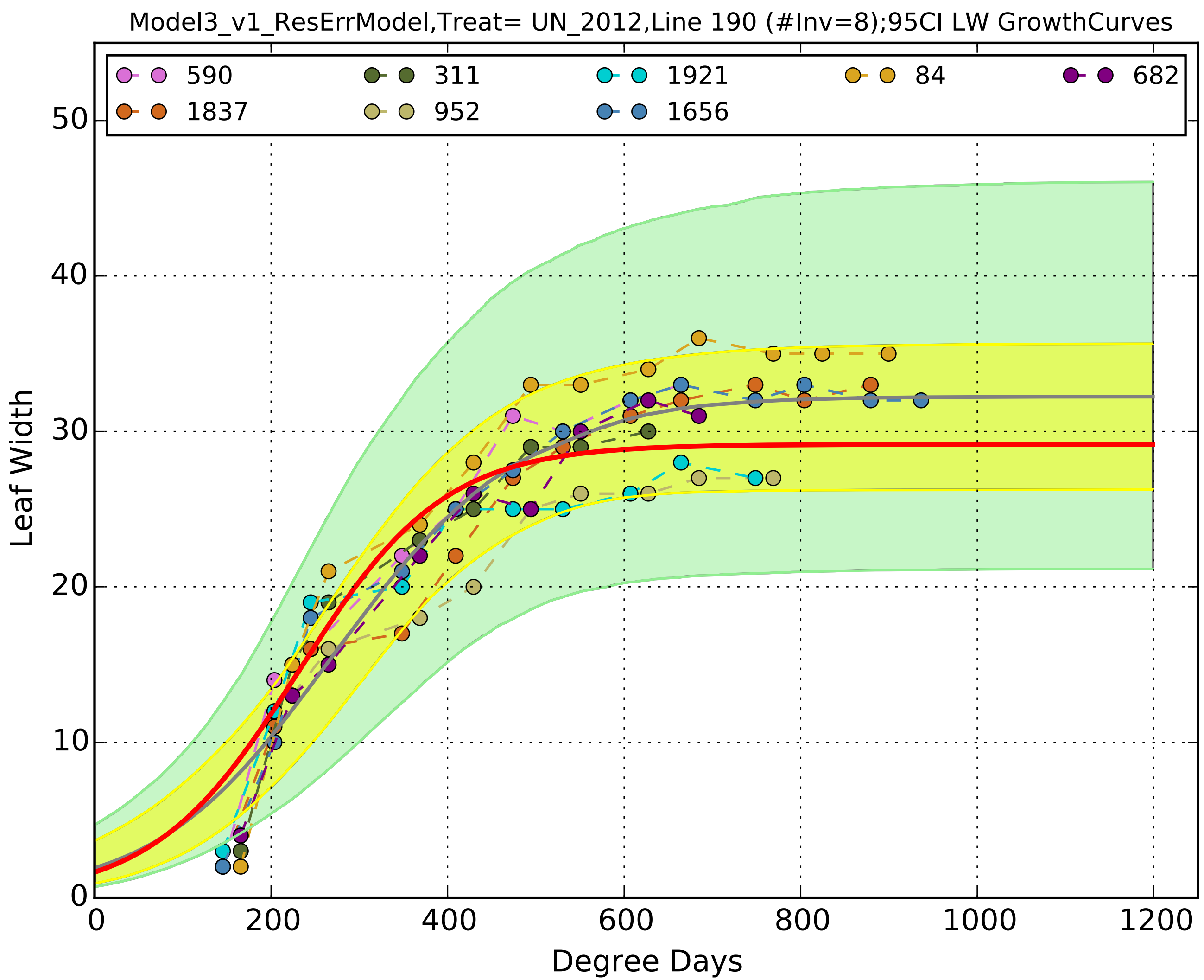

Model3_v1_ResErrModel,Treat= UN_2012,Line 190 (#Inv=8);95CI LW GrowthCurves
590
311
1921
84
682
1837
952
1656
Leaf Width
Degree Days
0
10
20
30
40
50
0
200
400
600
800
1000
1200

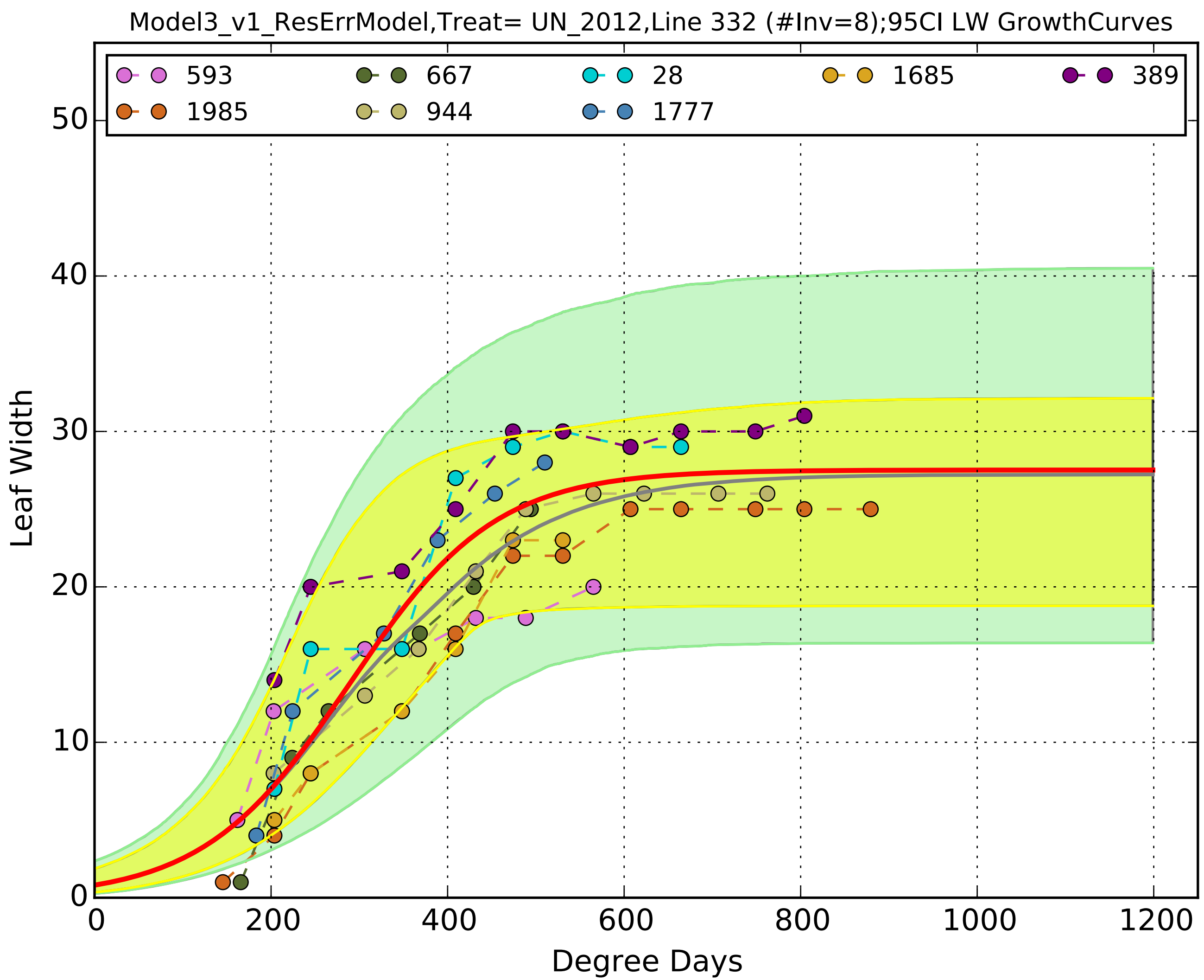
Model3_v1_ResErrModel,Treat= UN_2012,Line 332 (#Inv=8);95CI LW GrowthCurves
593
667
28
1685
389
1985
944
1777
Leaf Width
Degree Days
0
10
20
30
40
50
0
200
400
600
800
1000
1200

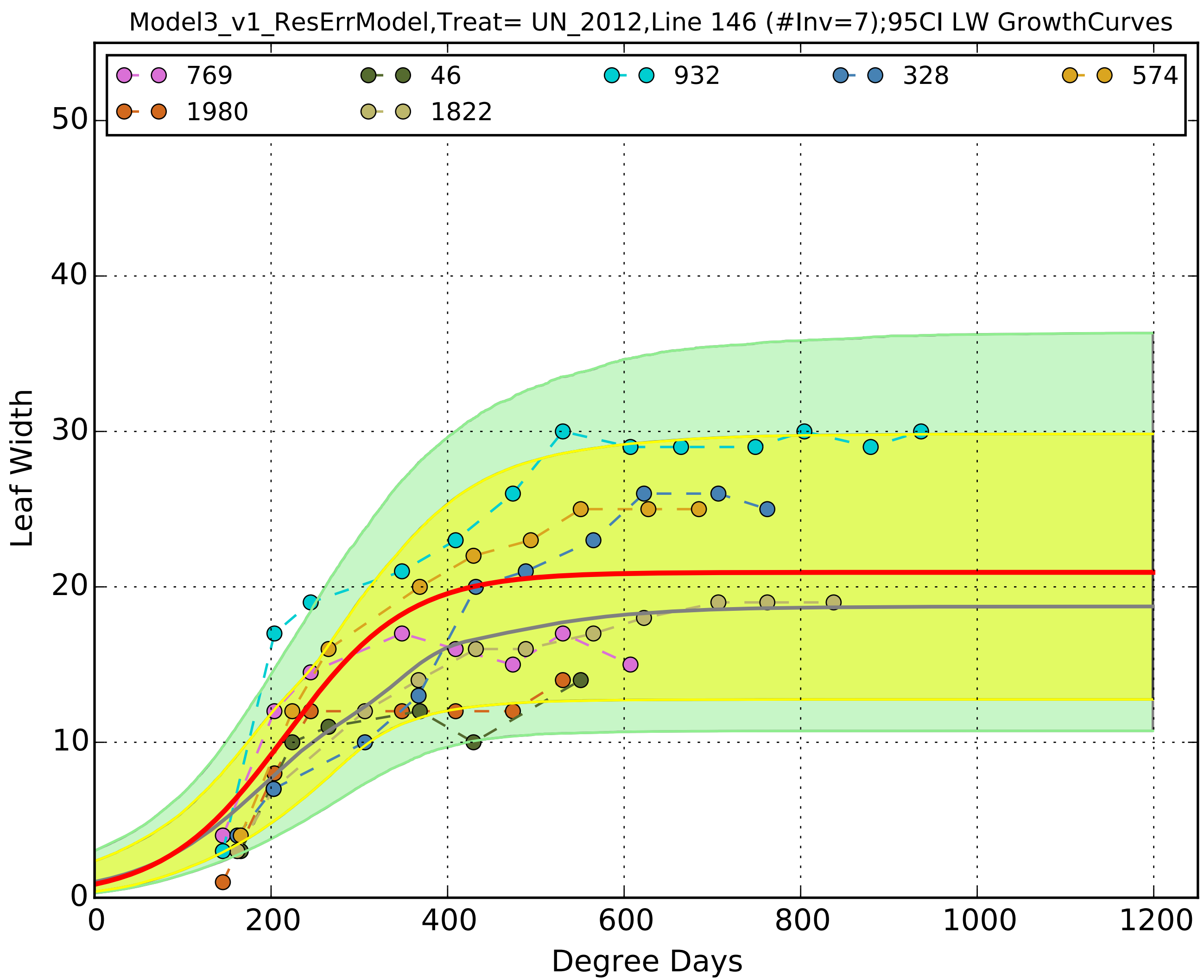

Model3_v1_ResErrModel,Treat= UN_2012,Line 146 (#Inv=7);95CI LW GrowthCurves
769
46
932
328
574
1980
1822
Leaf Width
Degree Days
0
10
20
30
40
50
0
200
400
600
800
1000
1200

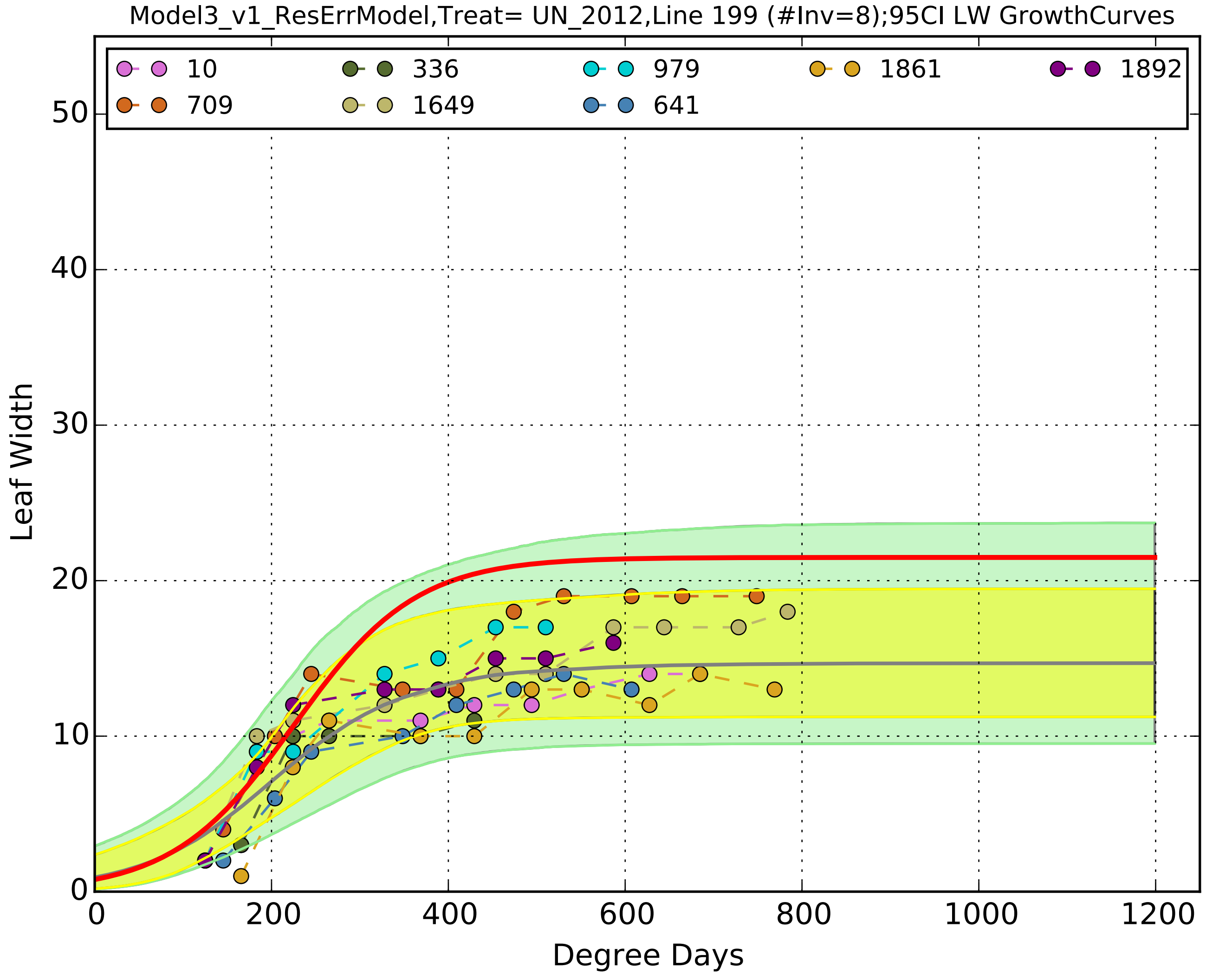

Model3_v1_ResErrModel,Treat= UN_2012,Line 199 (#Inv=8);95CI LW GrowthCurves
10
336
979
1861
1892
709
1649
641
Leaf Width
Degree Days
0
10
20
30
40
50
0
200
400
600
800
1000
1200

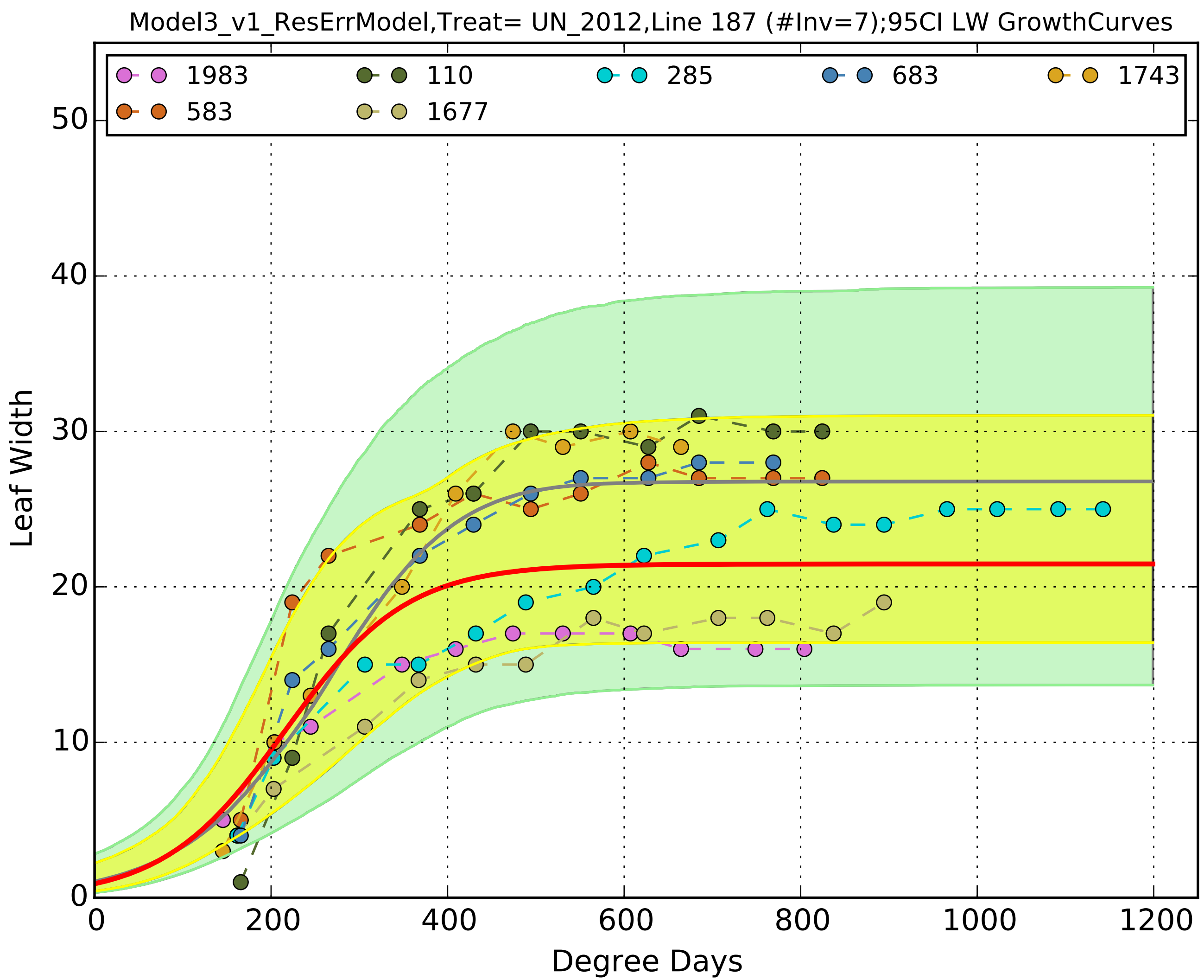

Model3_v1_ResErrModel,Treat= UN_2012,Line 187 (#Inv=7);95CI LW GrowthCurves
1983
110
285
683
1743
583
1677
Leaf Width
Degree Days
0
10
20
30
40
50
0
200
400
600
800
1000
1200

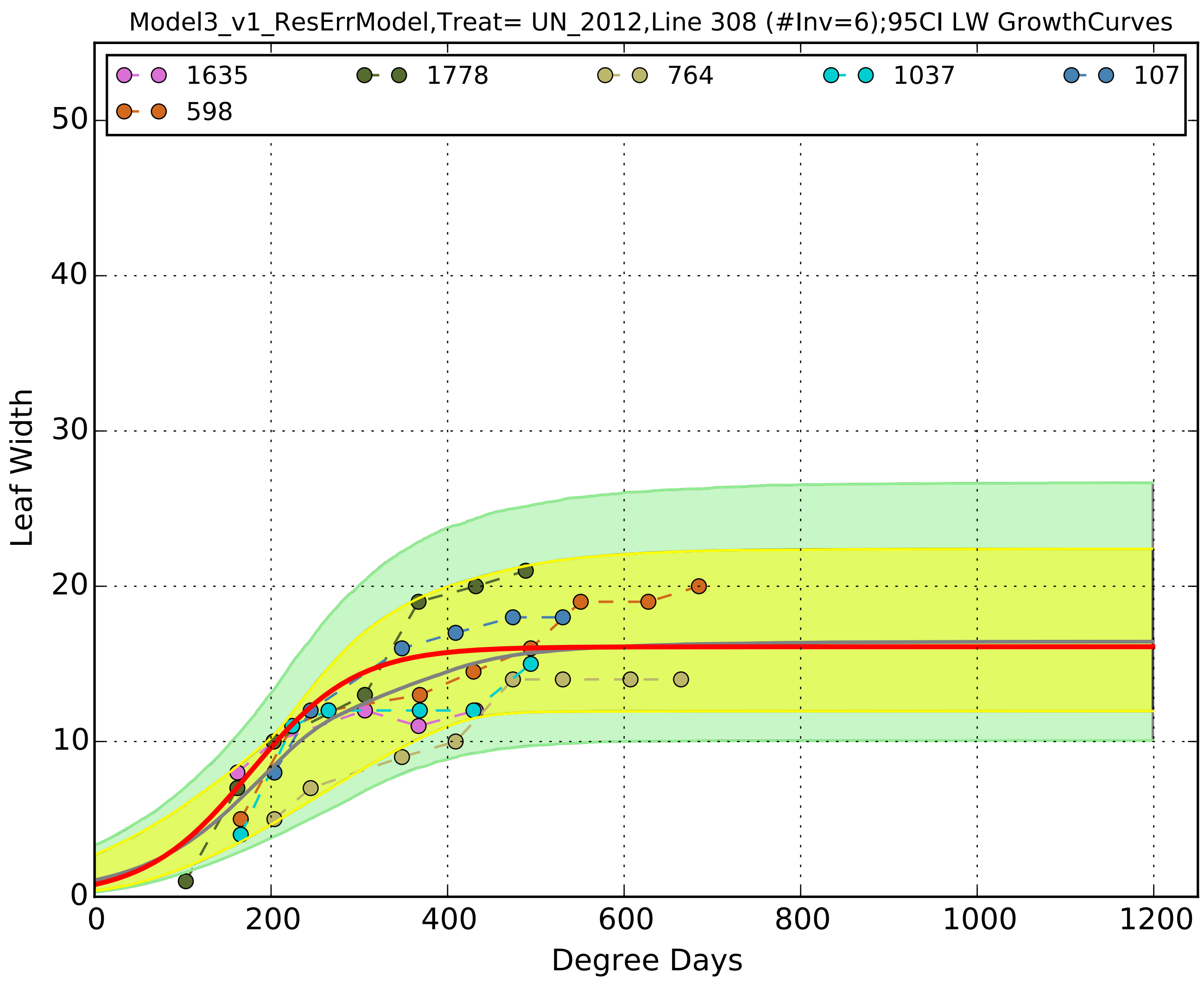
Model3_v1_ResErrModel,Treat= UN_2012,Line 308 (#Inv=6);95CI LW GrowthCurves
1635
1778
764
1037
107
598
Leaf Width
Degree Days
0
10
20
30
40
50
0
200
400
600
800
1000
1200

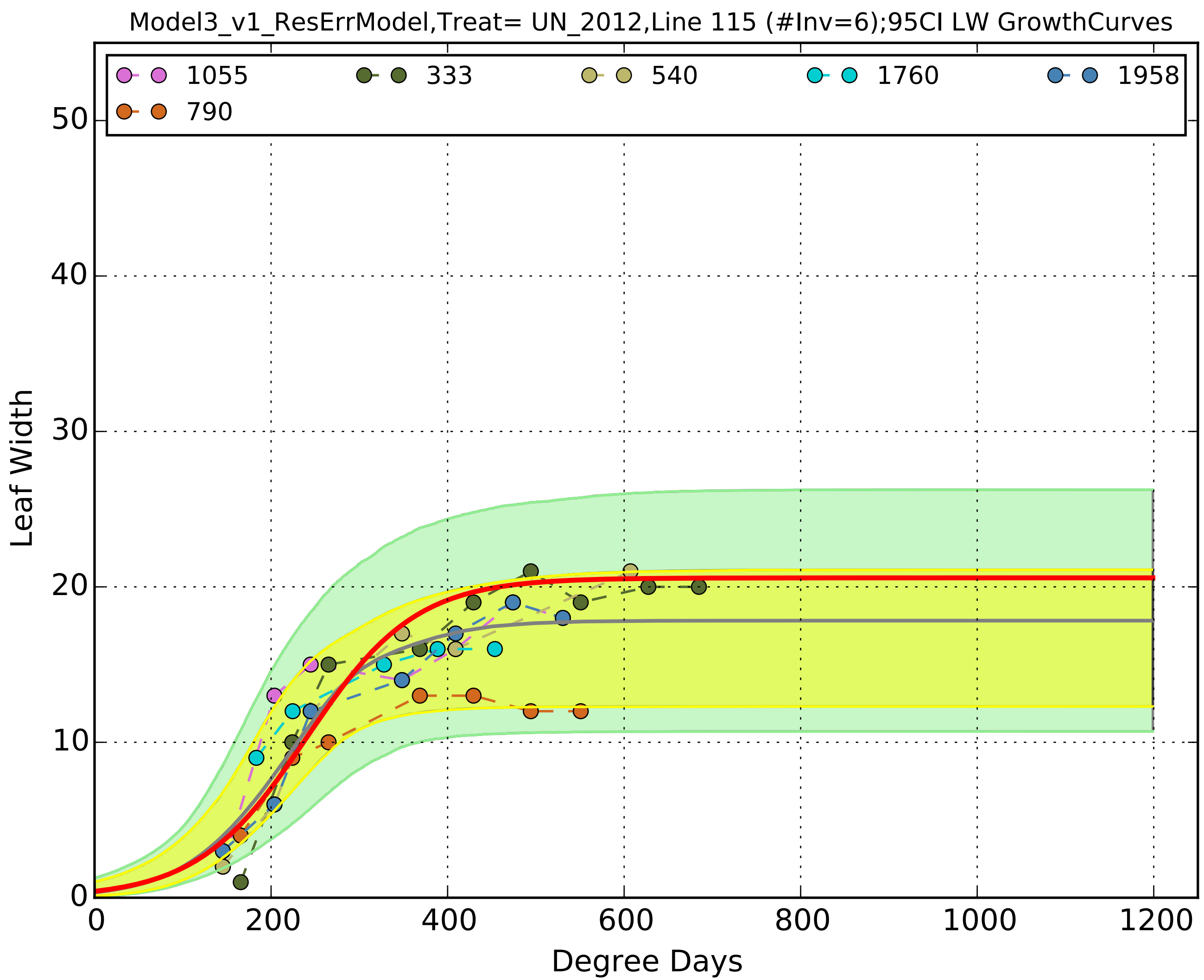
Model3_v1_ResErrModel,Treat= UN_2012,Line 115 (#Inv=6);95CI LW GrowthCurves
1055
333
540
1760
1958
790
Leaf Width
Degree Days
0
10
20
30
40
50
0
200
400
600
800
1000
1200

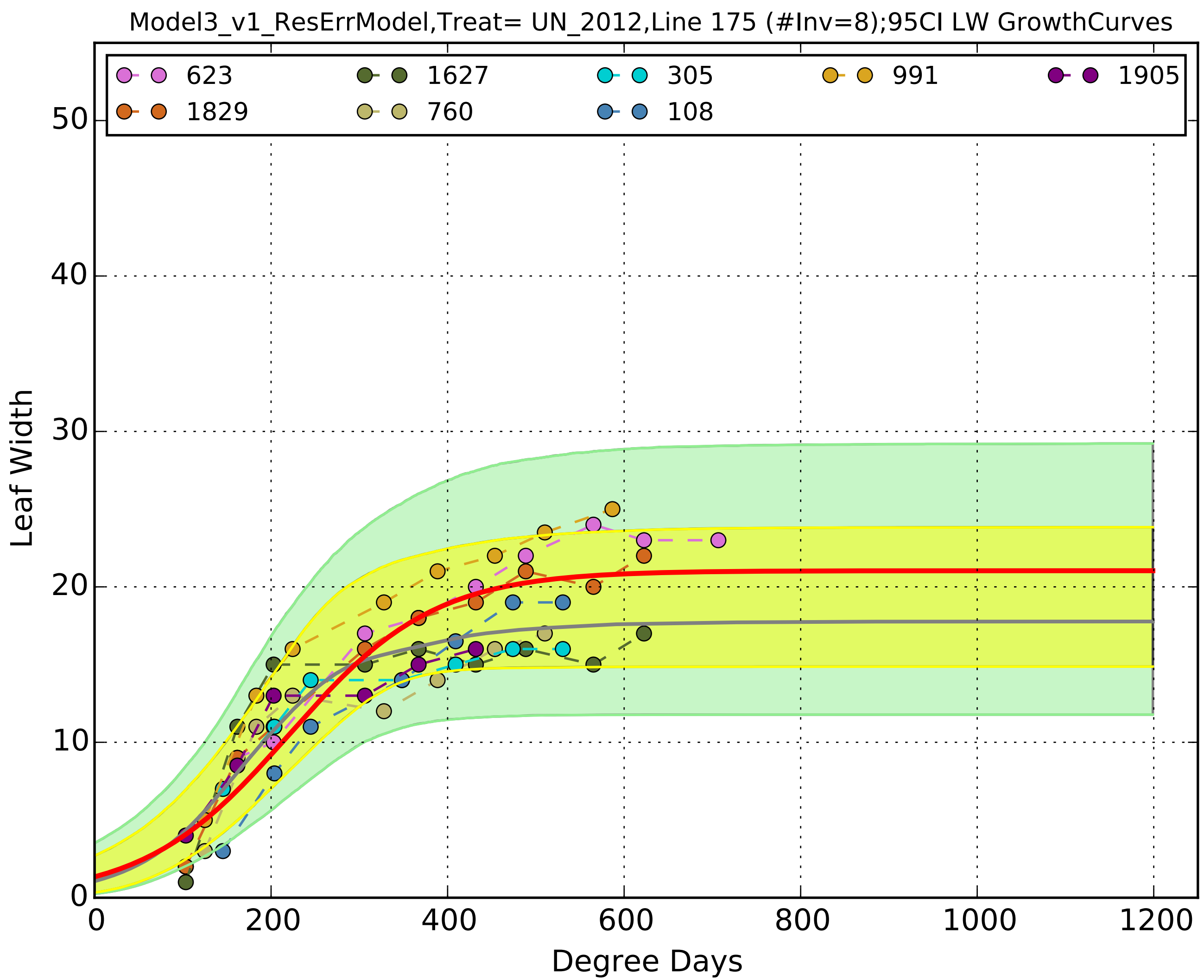

Model3_v1_ResErrModel,Treat= UN_2012,Line 175 (#Inv=8);95CI LW GrowthCurves
623
1627
305
991
1905
1829
760
108
Leaf Width
Degree Days
0
10
20
30
40
50
0
200
400
600
800
1000
1200

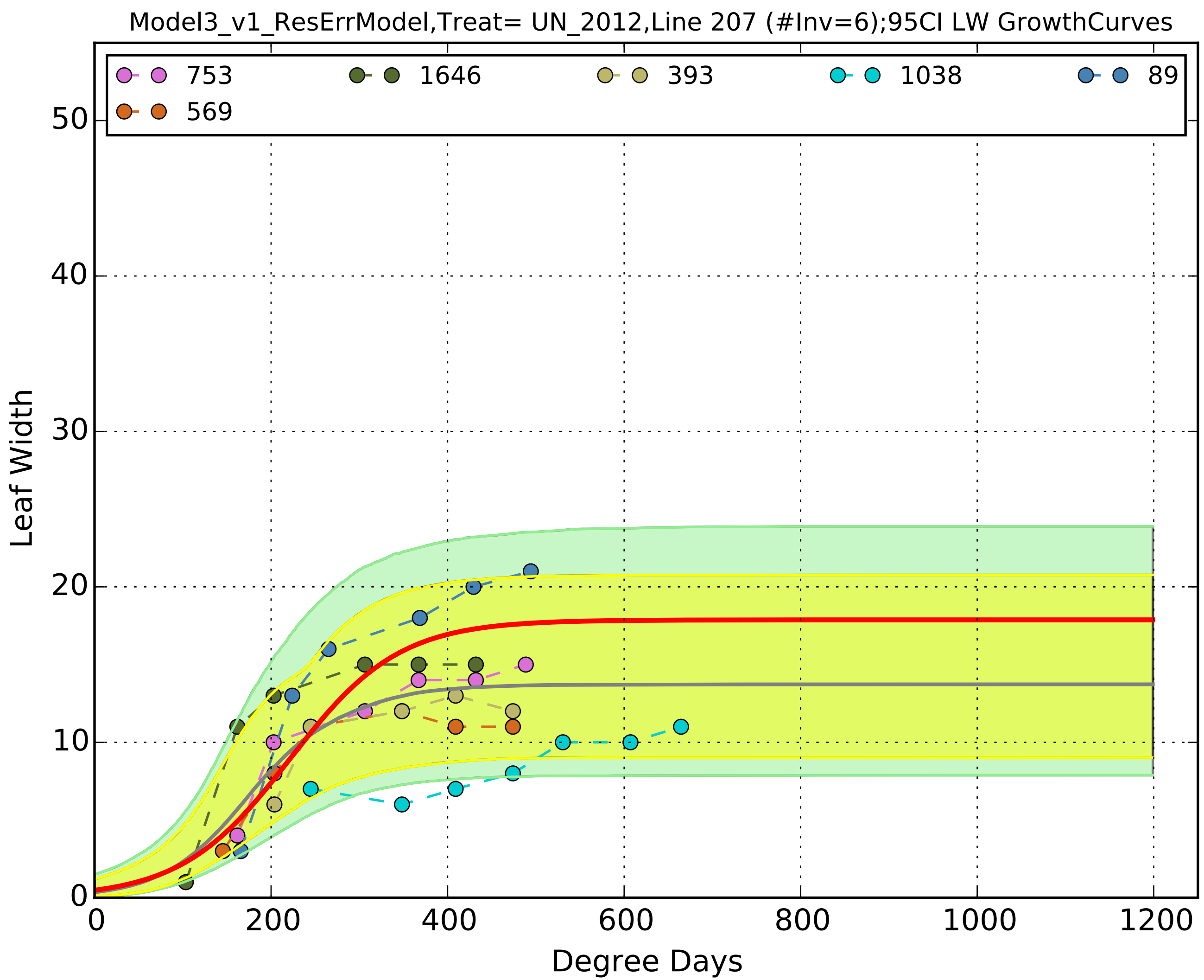

Model3_v1_ResErrModel,Treat= UN_2012,Line 207 (#Inv=6);95CI LW GrowthCurves
753
1646
393
1038
89
569
Leaf Width
0
10
20
30
40
50
0
200
400
600
800
1000
1200
Degree Days

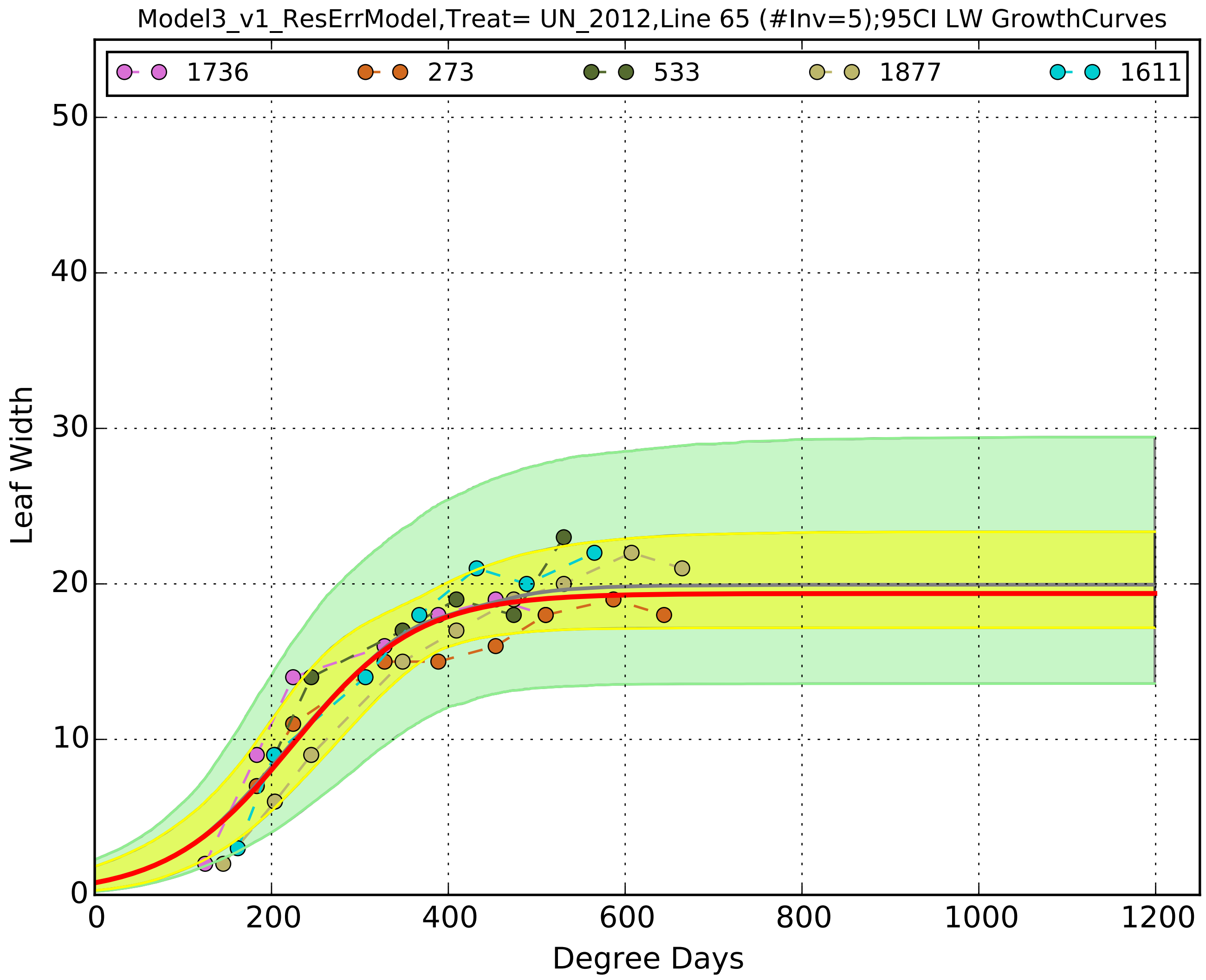
Model3_v1_ResErrModel,Treat= UN_2012,Line 65 (#Inv=5);95CI LW GrowthCurves
1736
273
533
1877
1611
Leaf Width
Degree Days
0
10
20
30
40
50
0
200
400
600
800
1000
1200

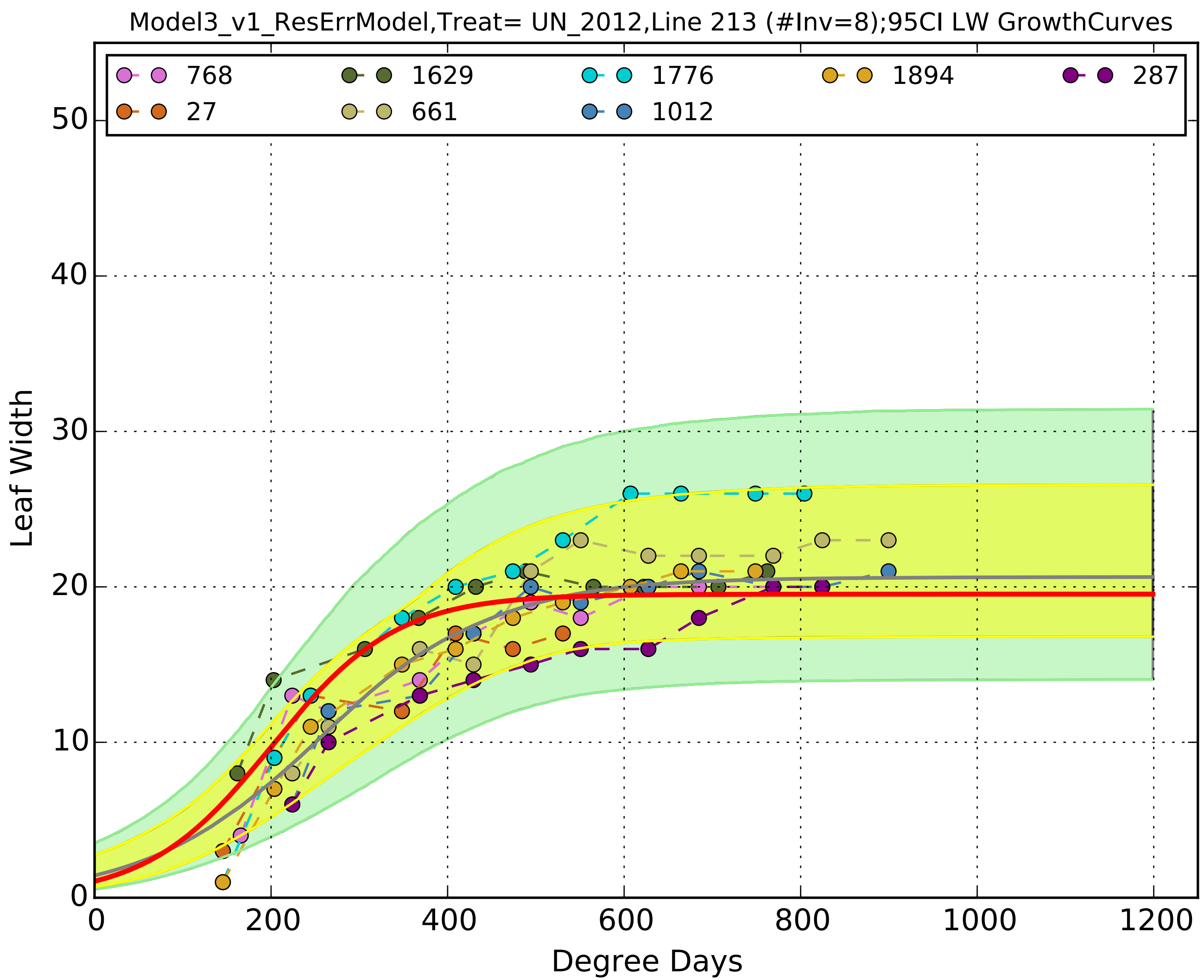
Model3_v1_ResErrModel,Treat= UN_2012,Line 213 (#Inv=8);95CI LW GrowthCurves
768
1629
1776
1894
287
27
661
1012
Leaf Width
Degree Days
0
10
20
30
40
50
0
200
400
600
800
1000
1200

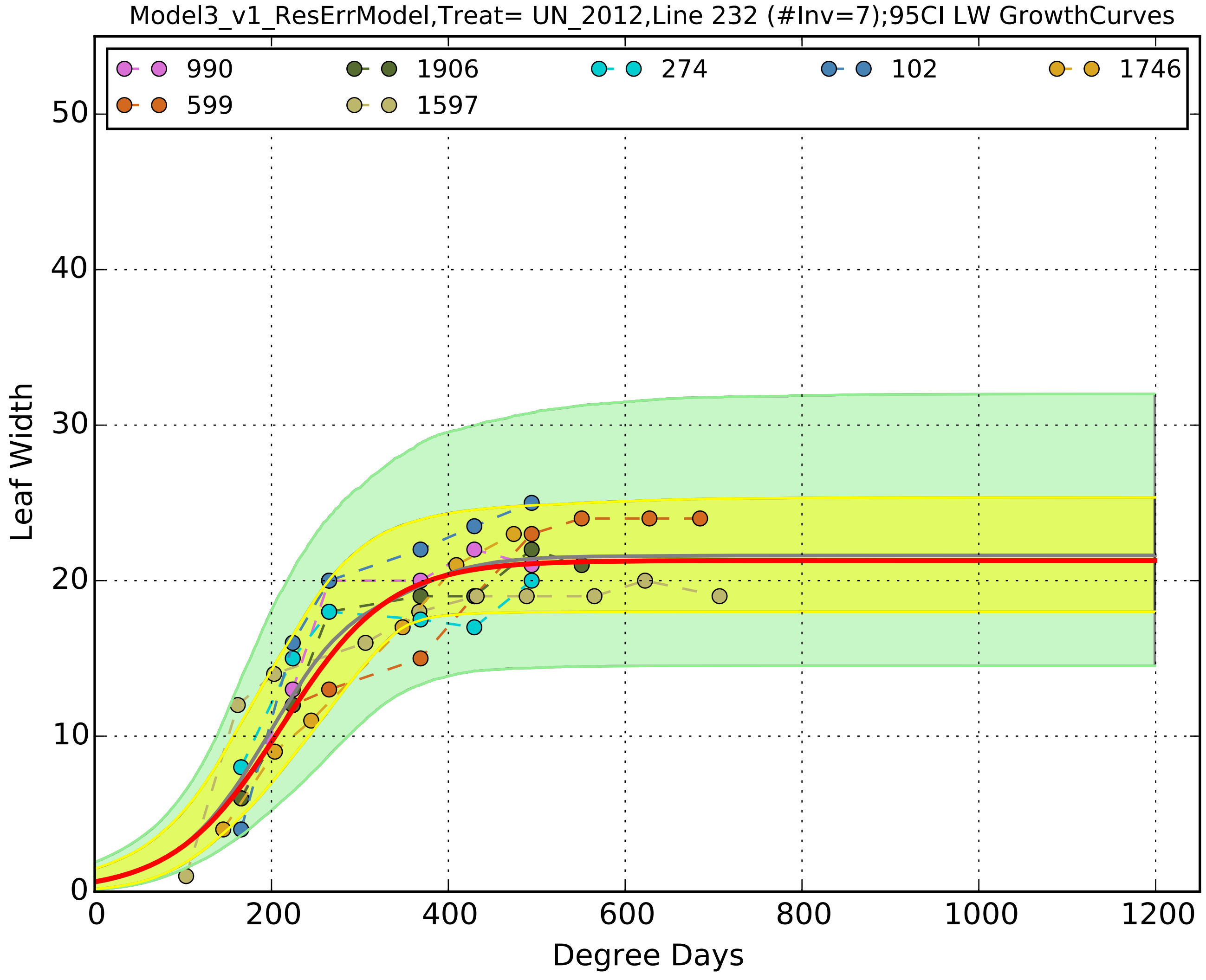

Model3_v1_ResErrModel,Treat= UN_2012,Line 232 (#Inv=7);95CI LW GrowthCurves
990
1906
274
102
1746
599
1597
Leaf Width
Degree Days
0
10
20
30
40
50
0
200
400
600
800
1000
1200

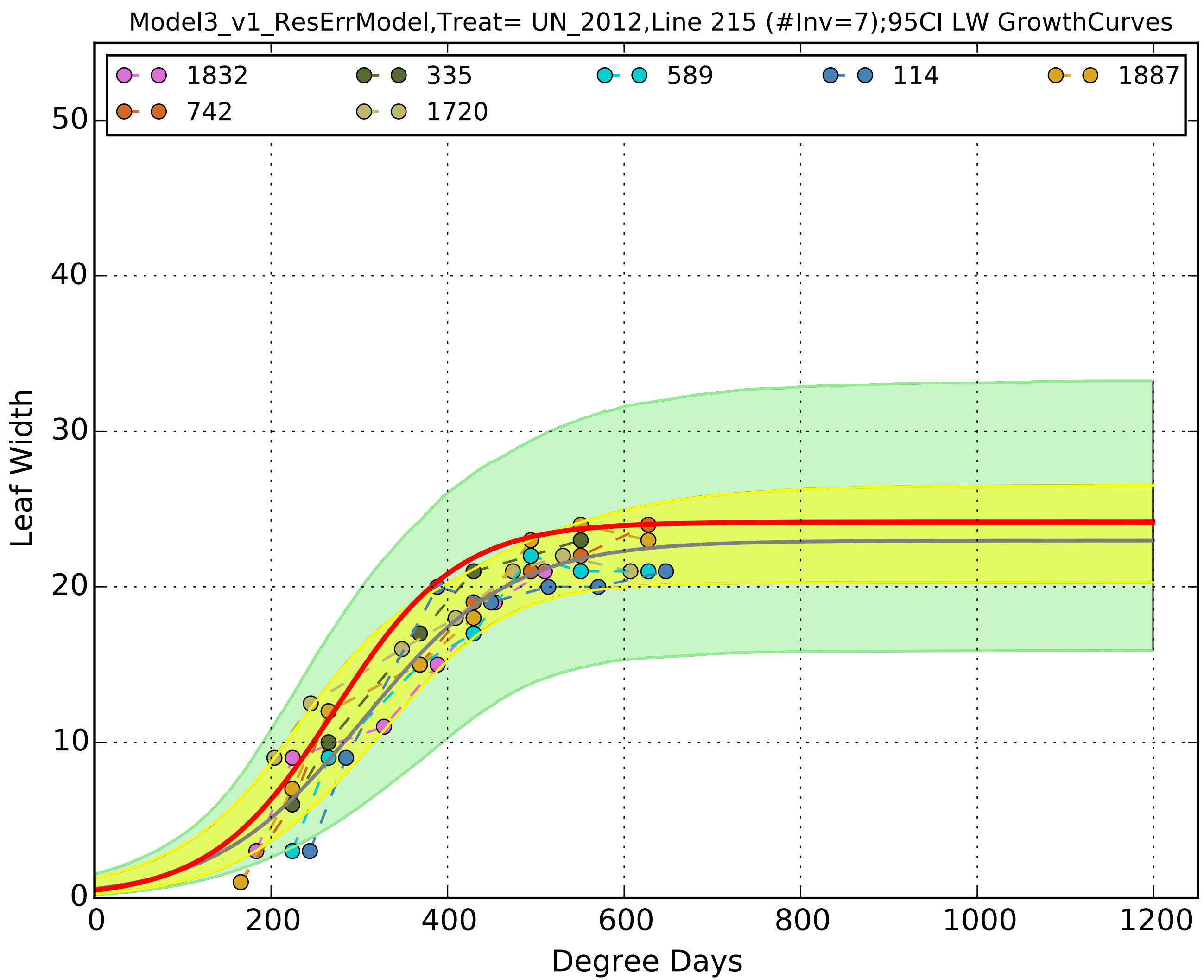
Model3_v1_ResErrModel,Treat= UN_2012,Line 215 (#Inv=7);95CI LW GrowthCurves
1832
335
589
114
1887
742
1720
Leaf Width
Degree Days
0
10
20
30
40
50
0
200
400
600
800
1000
1200

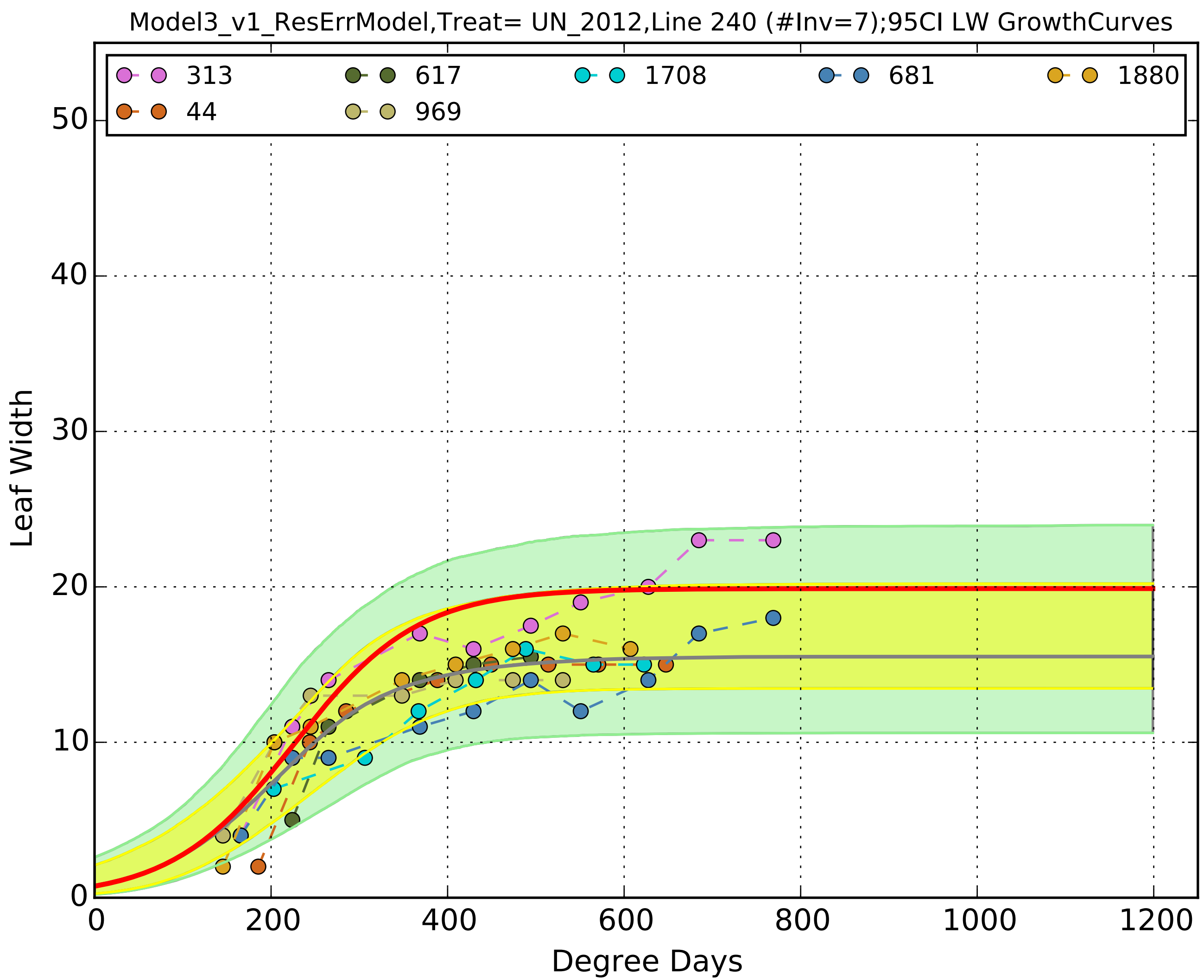

Model3_v1_ResErrModel,Treat= UN_2012,Line 240 (#Inv=7);95CI LW GrowthCurves
313
617
1708
681
1880
44
969
Leaf Width
Degree Days
0
10
20
30
40
50
0
200
400
600
800
1000
1200

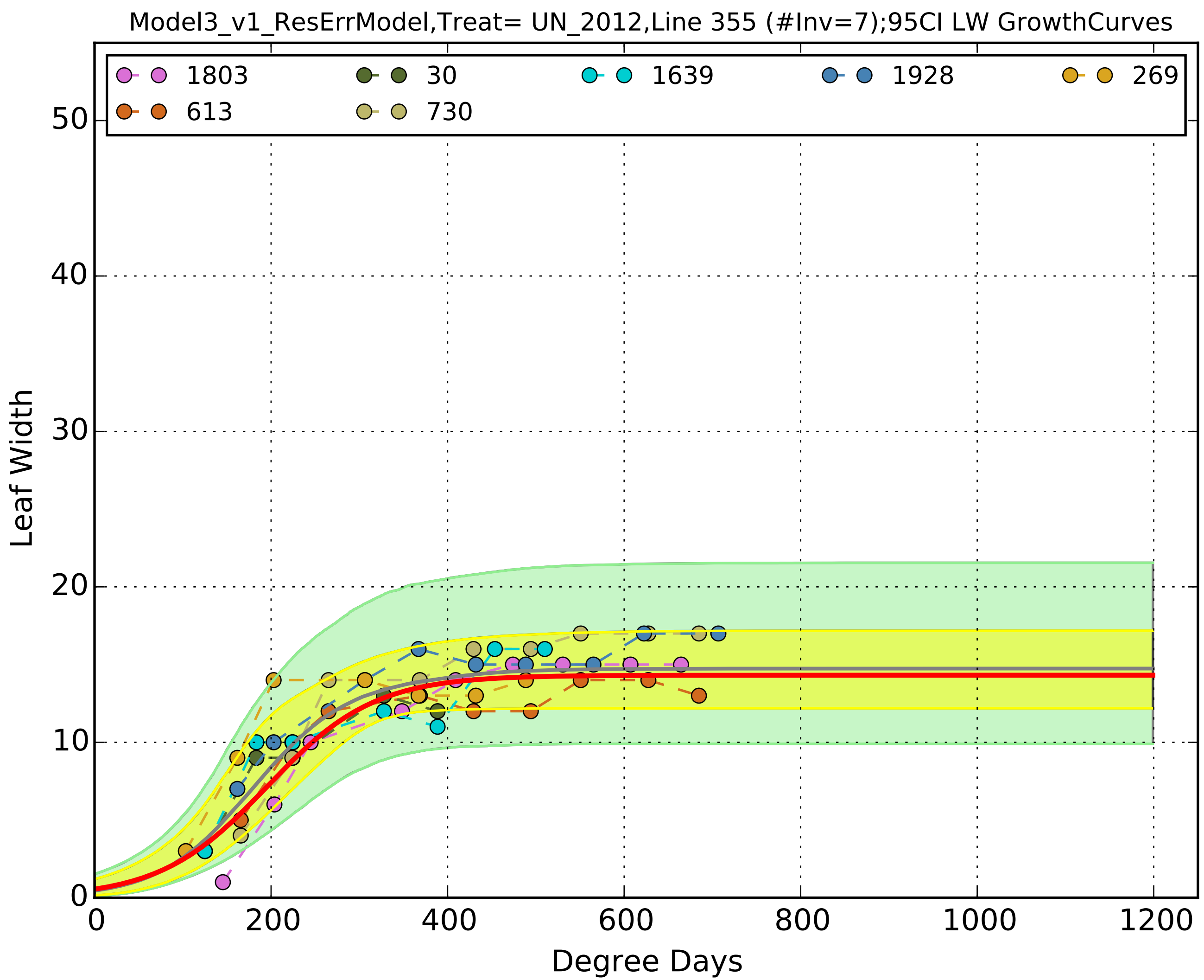
Model3_v1_ResErrModel,Treat= UN_2012,Line 355 (#Inv=7);95CI LW GrowthCurves
1803
30
1639
1928
269
613
730
Leaf Width
Degree Days
0
10
20
30
40
50
0
200
400
600
800
1000
1200

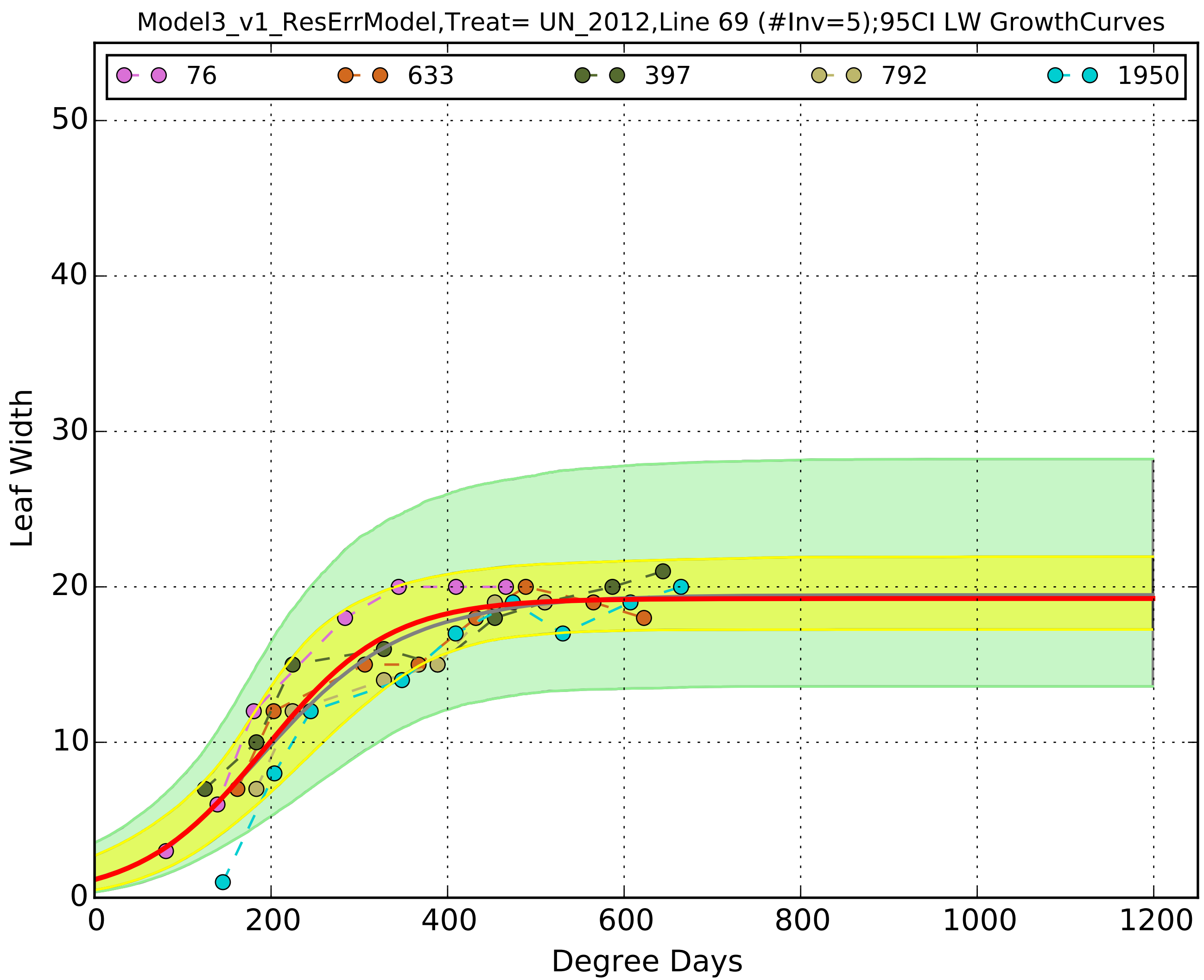
Model3_v1_ResErrModel,Treat= UN_2012,Line 69 (#Inv=5);95CI LW GrowthCurves
76
633
397
792
1950
Leaf Width
Degree Days
0
10
20
30
40
50
0
200
400
600
800
1000
1200

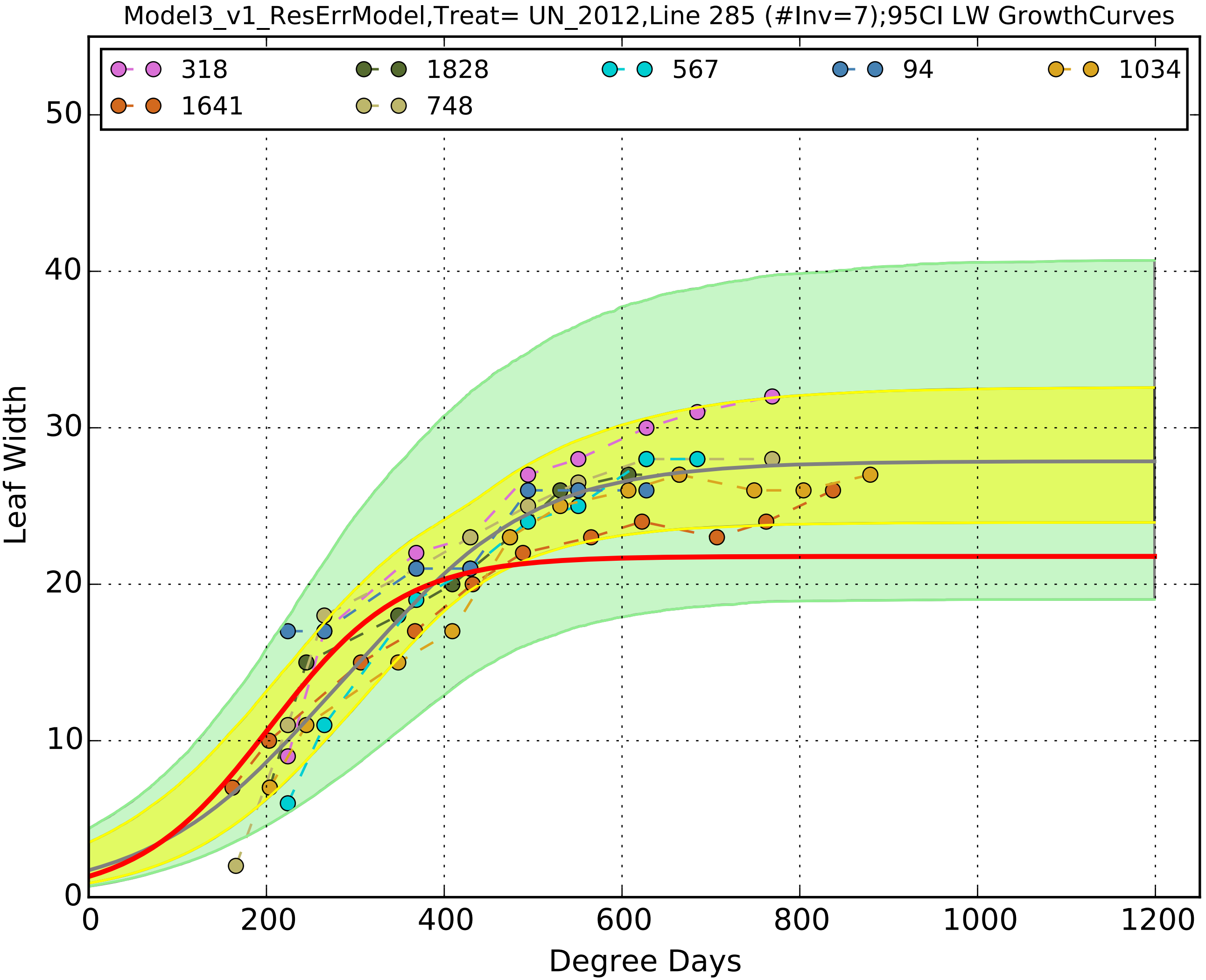

Model3_v1_ResErrModel,Treat= UN_2012,Line 285 (#Inv=7);95CI LW GrowthCurves
318
1828
567
94
1034
1641
748
Leaf Width
Degree Days
0
10
20
30
40
50
0
200
400
600
800
1000
1200

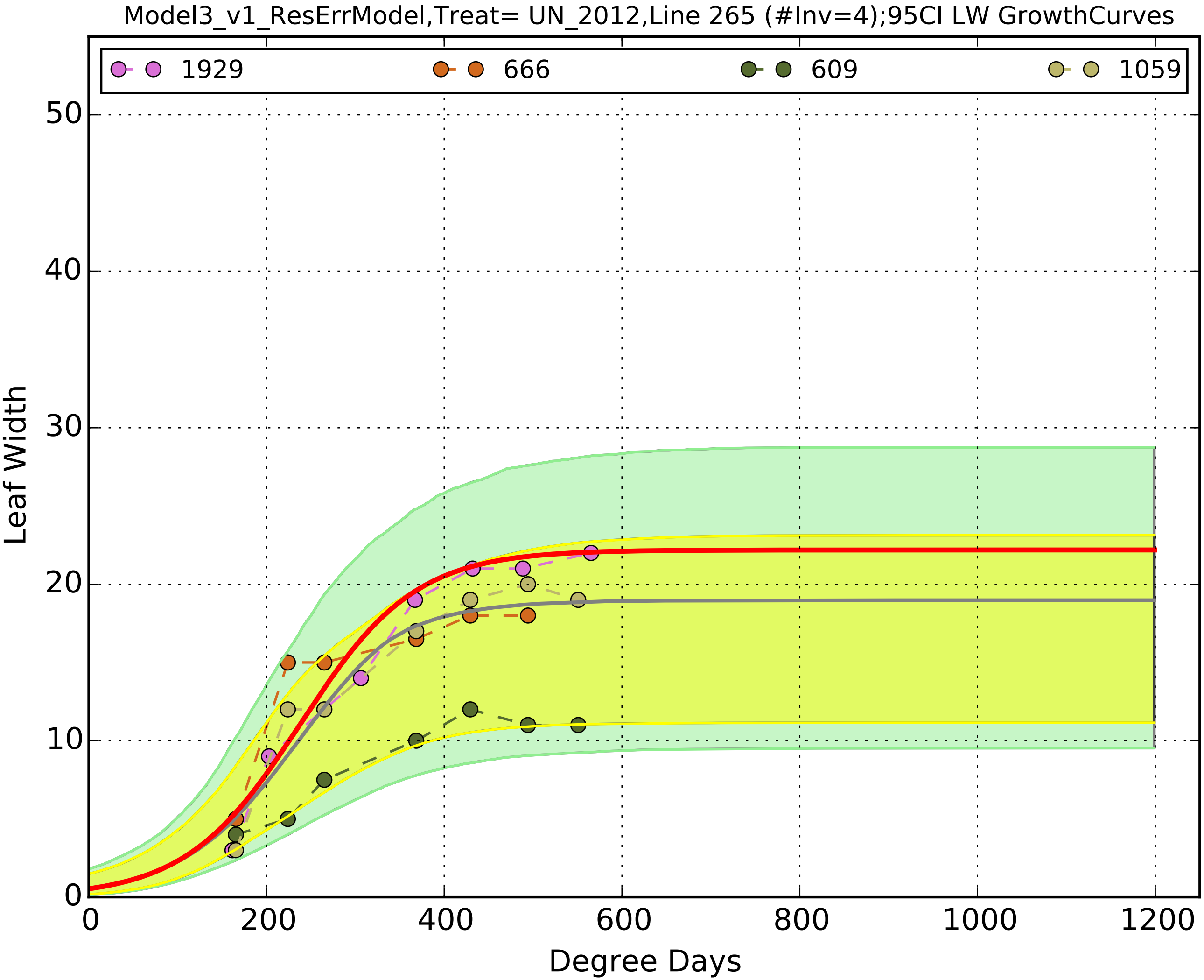

Model3_v1_ResErrModel,Treat= UN_2012,Line 265 (#Inv=4);95CI LW GrowthCurves
1929
666
609
1059
Leaf Width
Degree Days
0
10
20
30
40
50
0
200
400
600
800
1000
1200

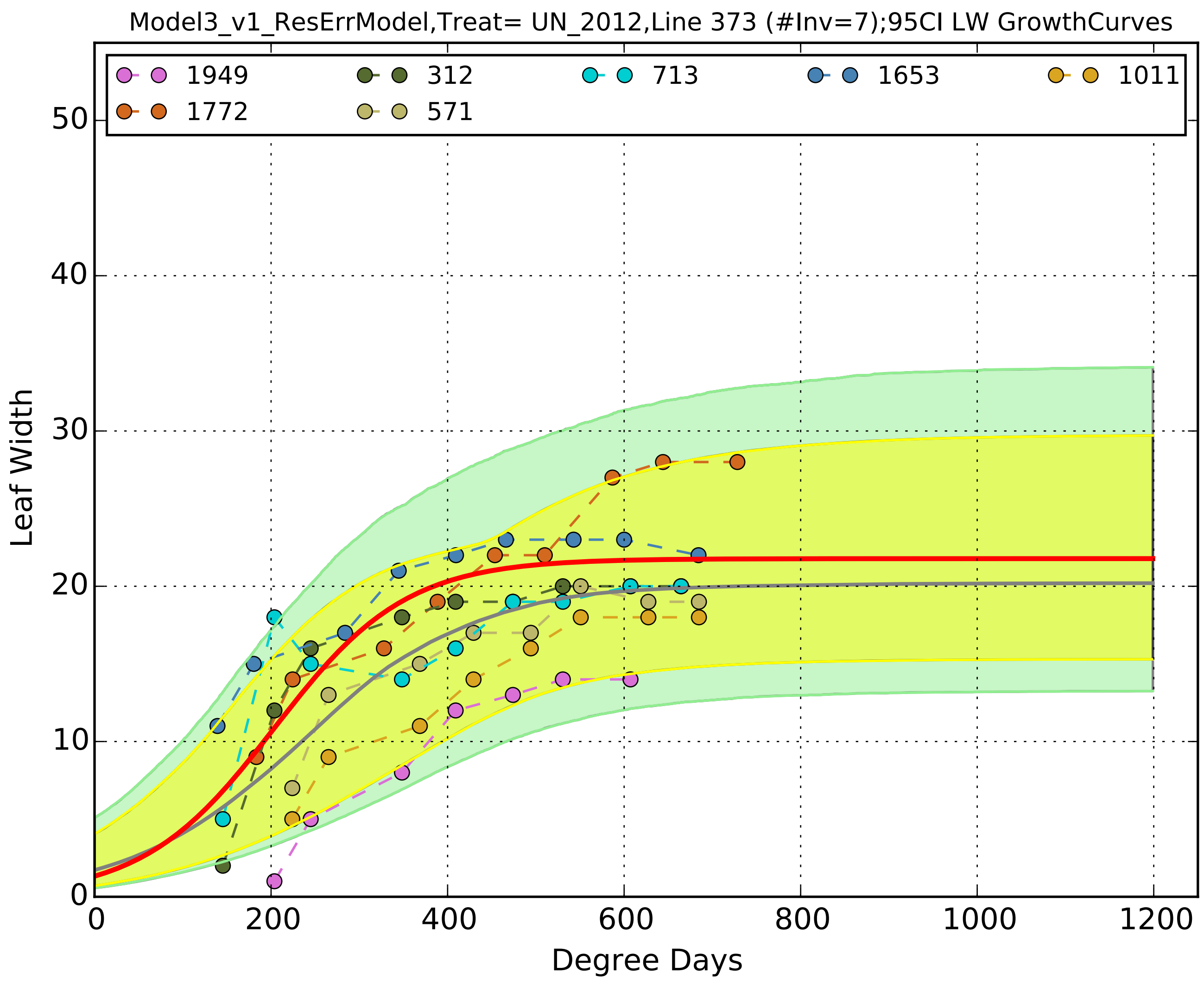
Model3_v1_ResErrModel,Treat= UN_2012,Line 373 (#Inv=7);95CI LW GrowthCurves
1949
312
713
1653
1011
1772
571
Leaf Width
Degree Days
0
10
20
30
40
50
0
200
400
600
800
1000
1200

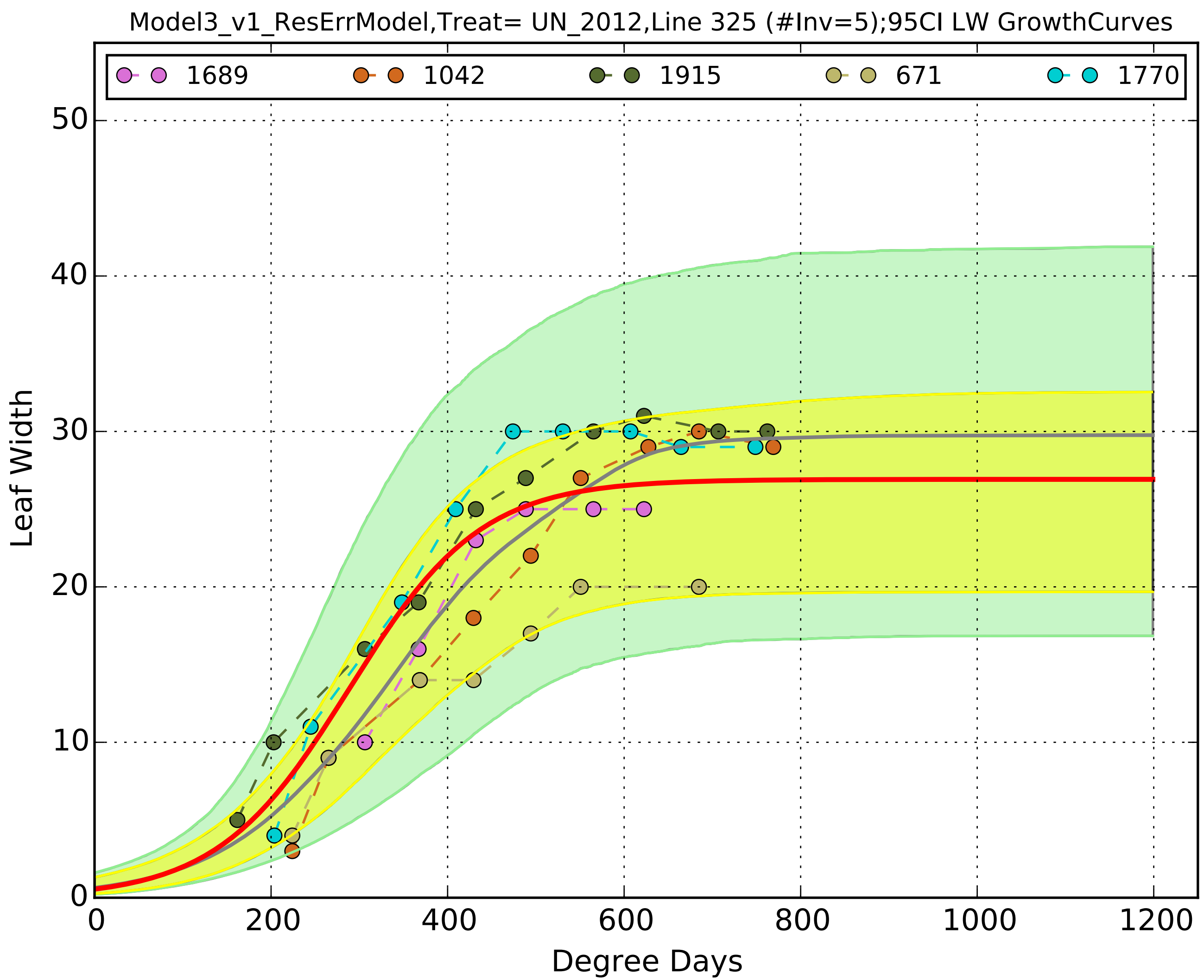

Model3_v1_ResErrModel,Treat= UN_2012,Line 325 (#Inv=5);95CI LW GrowthCurves
1689
1042
1915
671
1770
Leaf Width
Degree Days
0
10
20
30
40
50
0
200
400
600
800
1000
1200

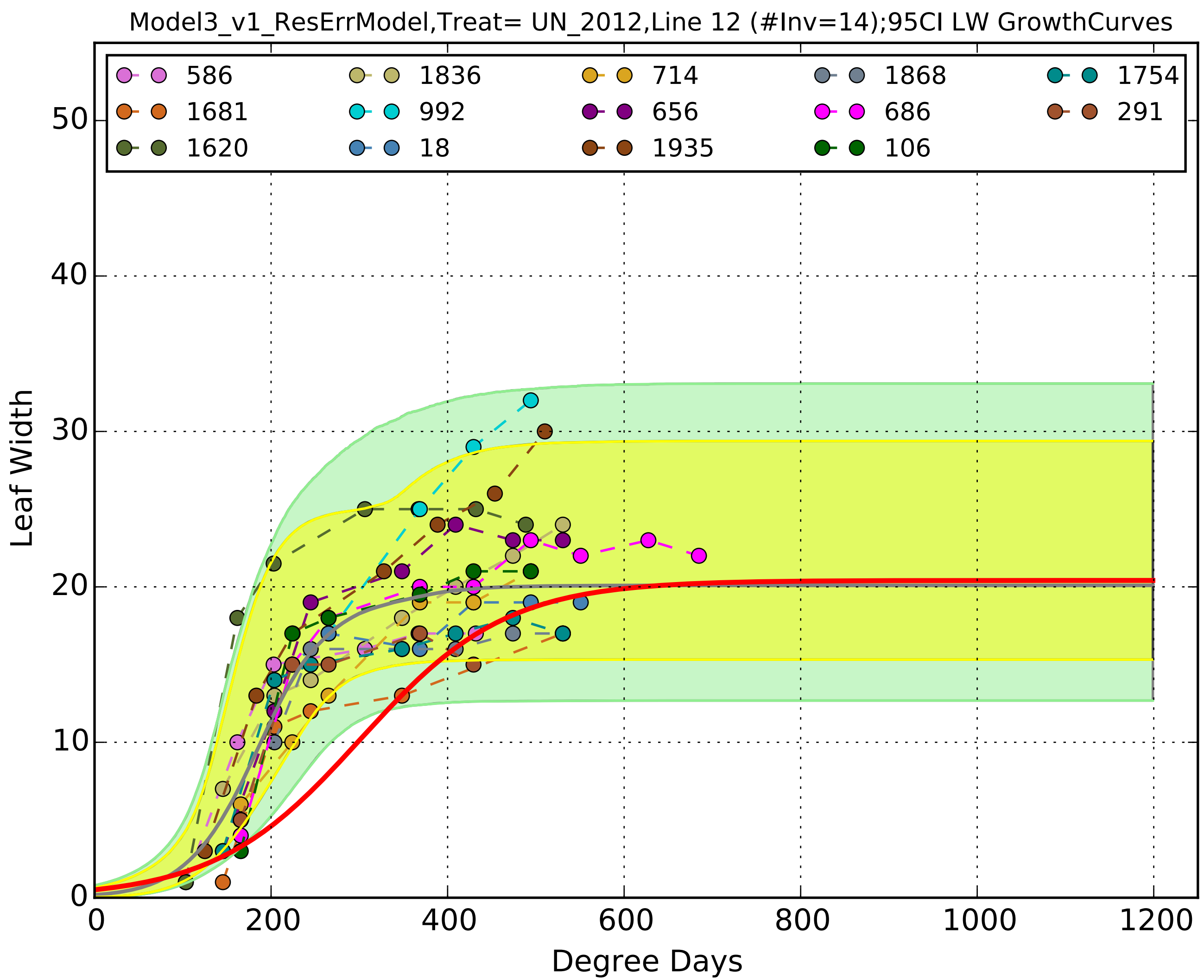

Model3_v1_ResErrModel,Treat= UN_2012,Line 12 (#Inv=14);95CI LW GrowthCurves
586
1681
1620
1836
992
18
714
656
1935
1868
686
106
1754
291
Leaf Width
Degree Days
0
10
20
30
40
50
0
200
400
600
800
1000
1200

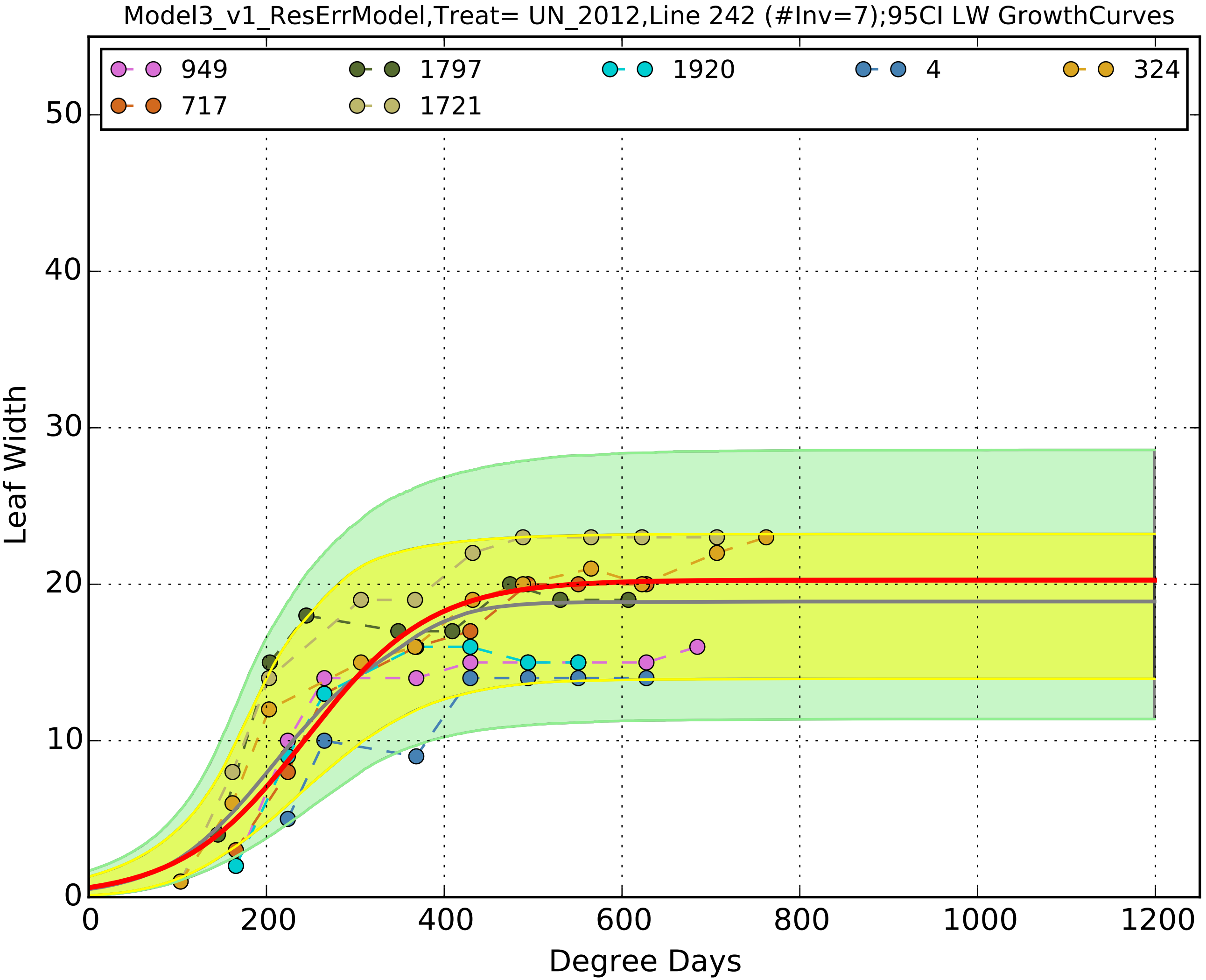
Model3_v1_ResErrModel,Treat= UN_2012,Line 242 (#Inv=7);95CI LW GrowthCurves
949
1797
1920
4
324
717
1721
Leaf Width
Degree Days
0
10
20
30
40
50
0
200
400
600
800
1000
1200

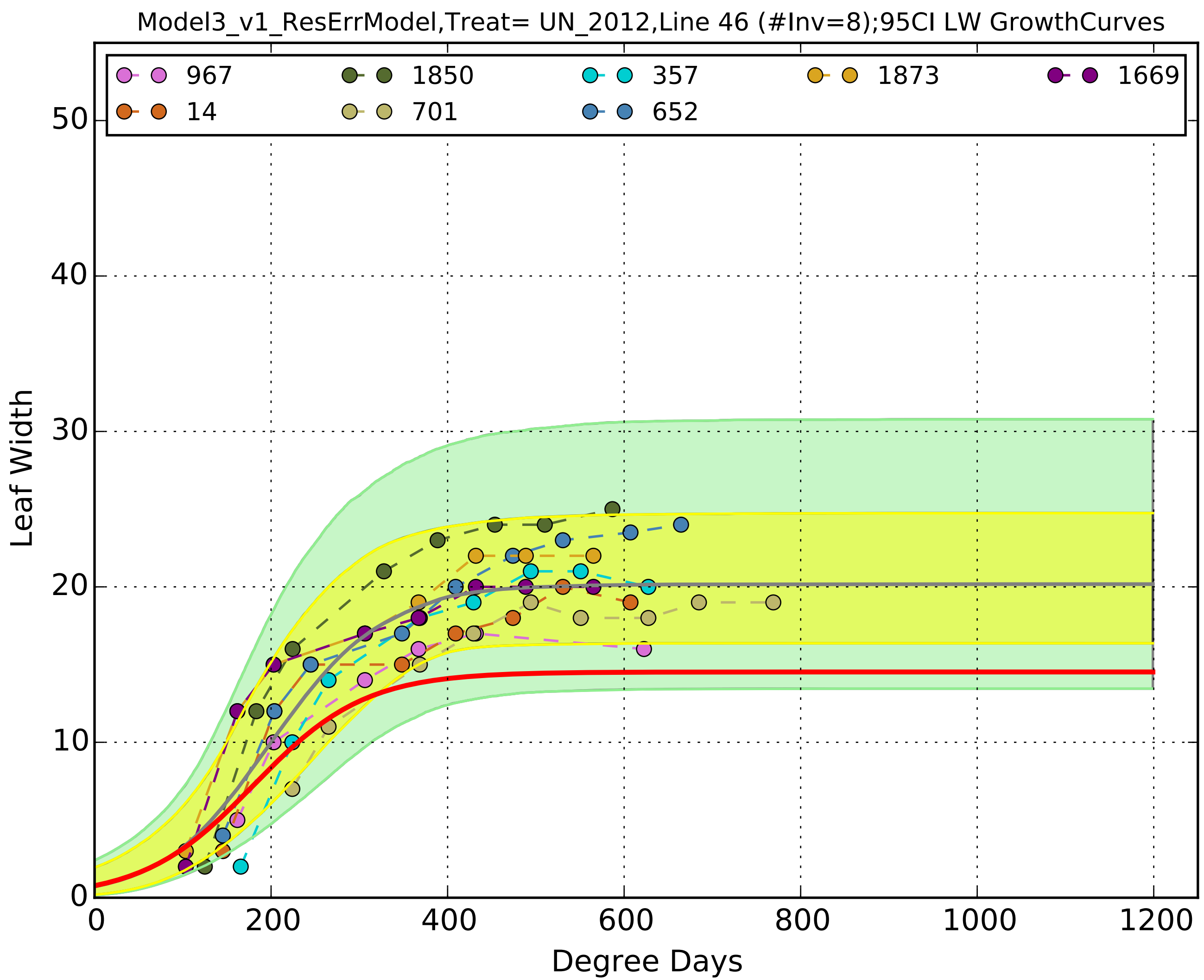
Model3_v1_ResErrModel,Treat= UN_2012,Line 46 (#Inv=8);95CI LW GrowthCurves
967
1850
357
1873
1669
14
701
652
Leaf Width
Degree Days
0
10
20
30
40
50
0
200
400
600
800
1000
1200

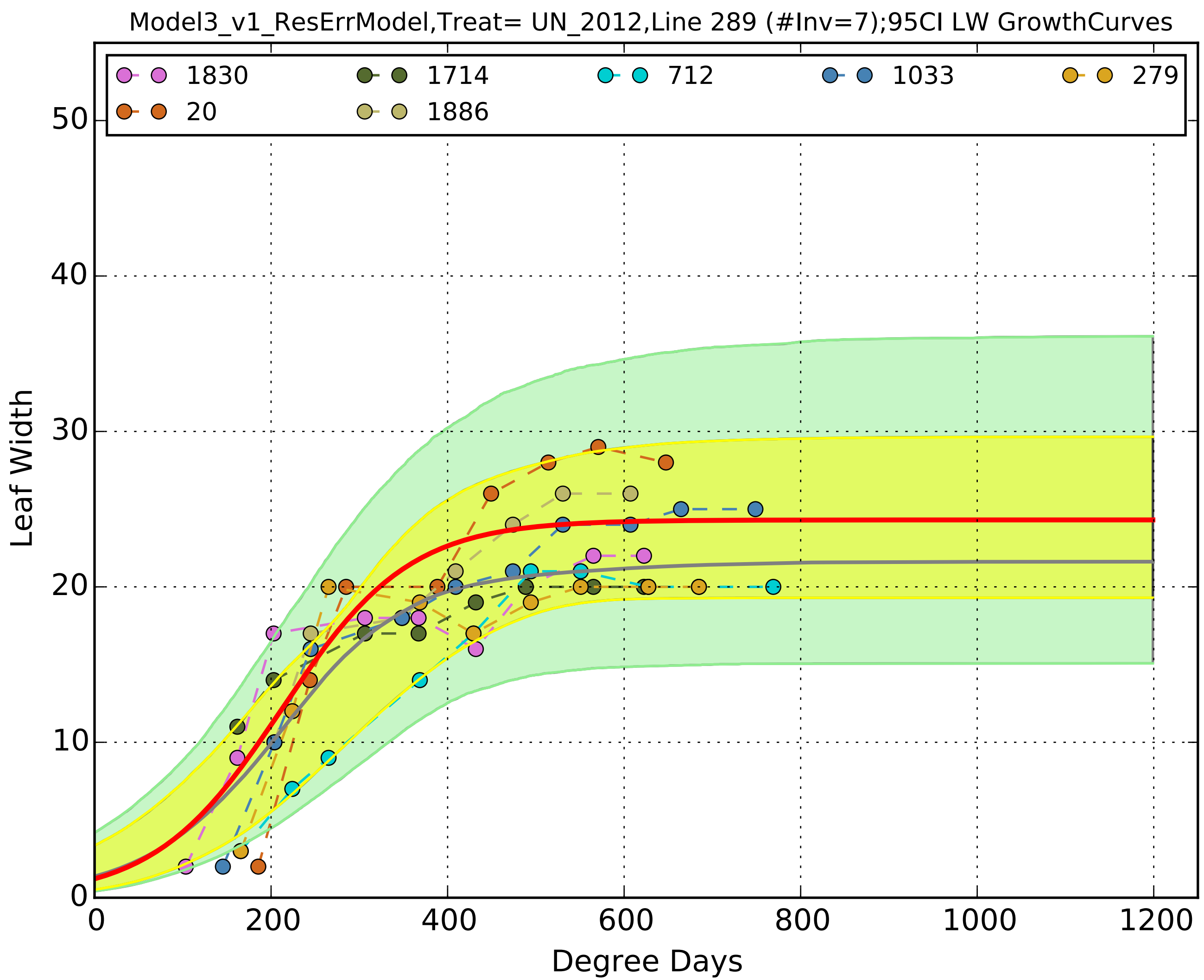

Model3_v1_ResErrModel,Treat= UN_2012,Line 289 (#Inv=7);95CI LW GrowthCurves
1830
1714
712
1033
279
20
1886
Leaf Width
Degree Days
0
10
20
30
40
50
0
200
400
600
800
1000
1200

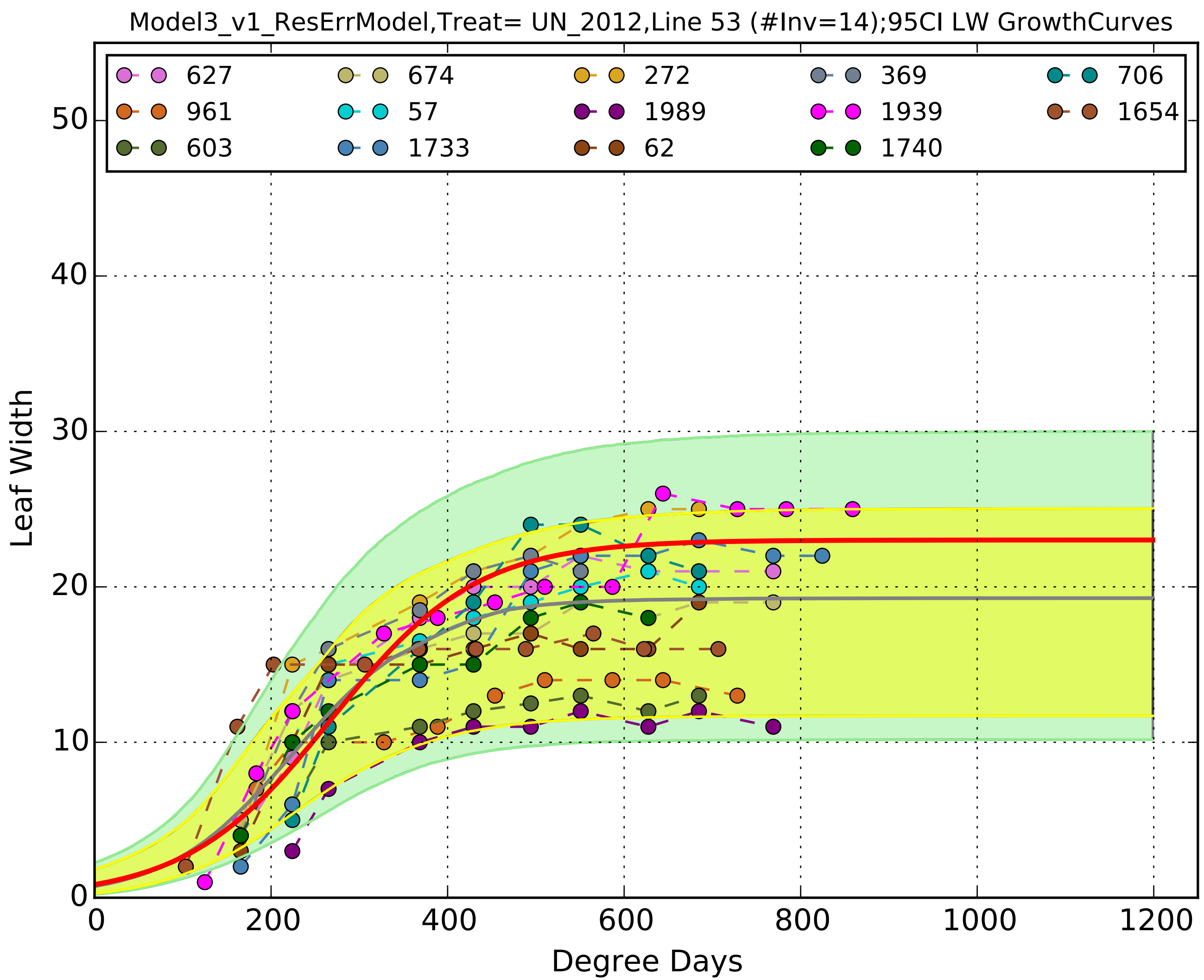

Model3_v1_ResErrModel,Treat= UN_2012,Line 53 (#Inv=14);95CI LW GrowthCurves
627
674
272
369
706
961
57
1989
1939
1654
603
1733
62
1740
Leaf Width
Degree Days
0
10
20
30
40
50
0
200
400
600
800
1000
1200

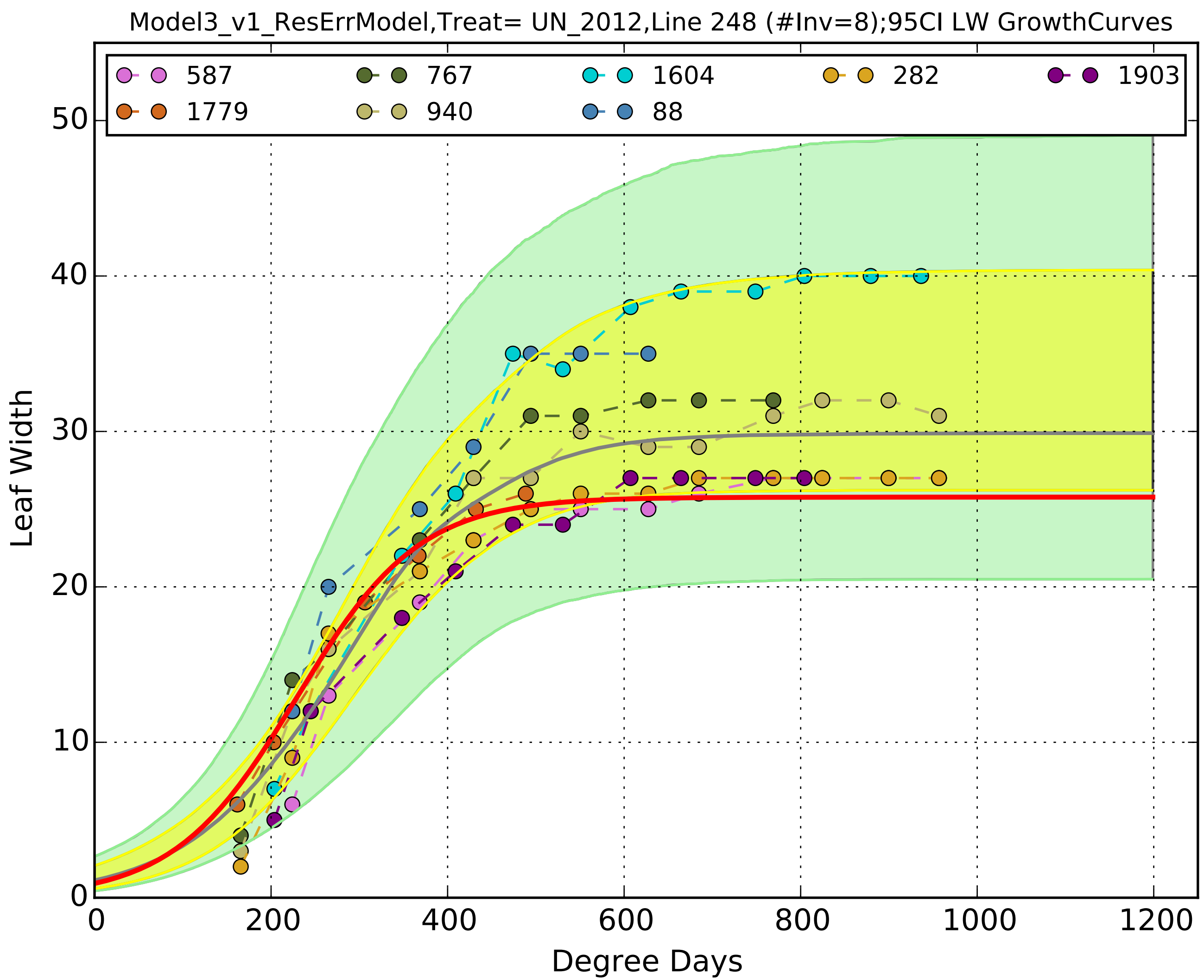
Model3_v1_ResErrModel,Treat= UN_2012,Line 248 (#Inv=8);95CI LW GrowthCurves
587
767
1604
282
1903
1779
940
88
Leaf Width
Degree Days
0
10
20
30
40
50
0
200
400
600
800
1000
1200

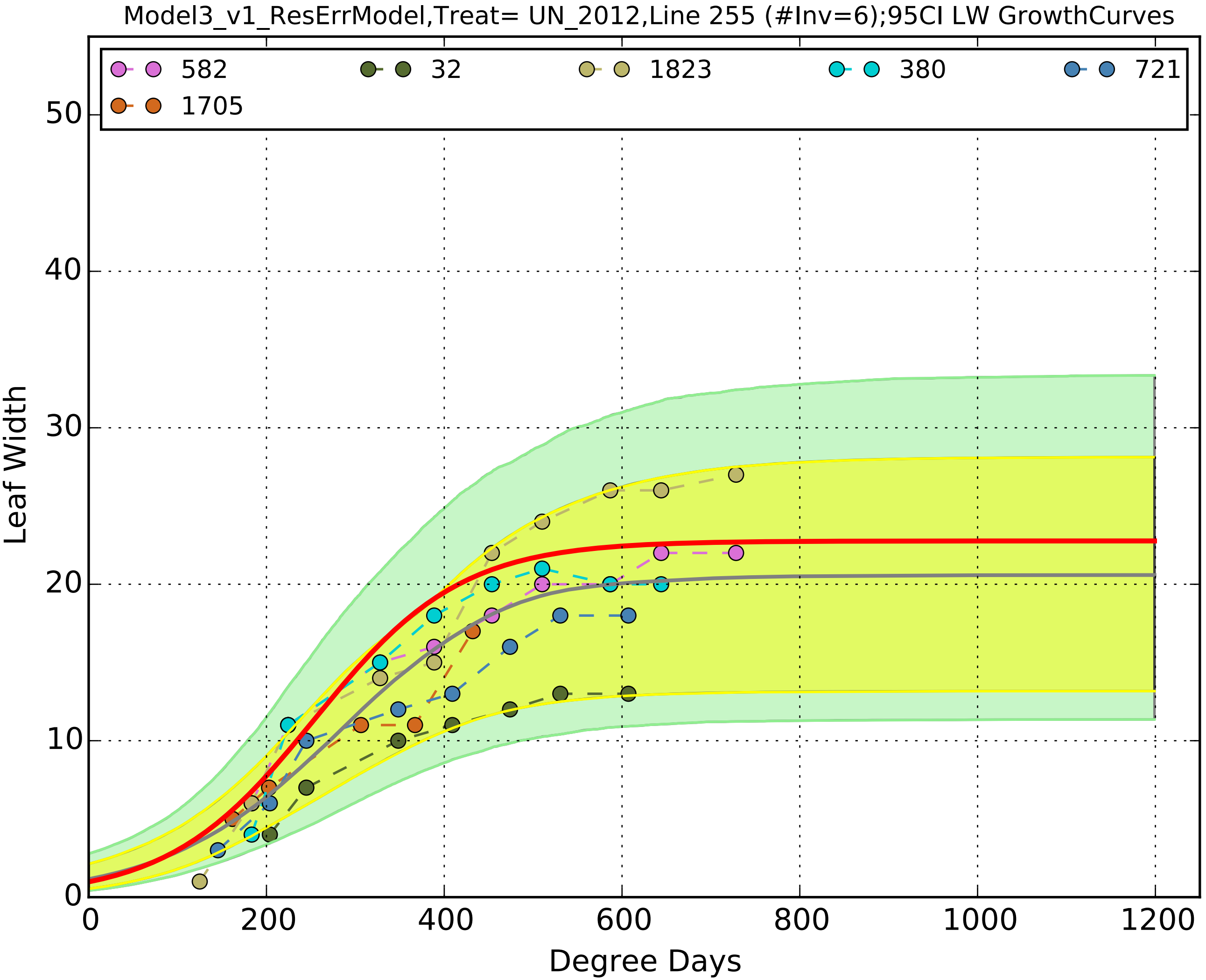

Model3_v1_ResErrModel,Treat= UN_2012,Line 255 (#Inv=6);95CI LW GrowthCurves
582
32
1823
380
721
1705
Leaf Width
Degree Days
0
10
20
30
40
50
0
200
400
600
800
1000
1200

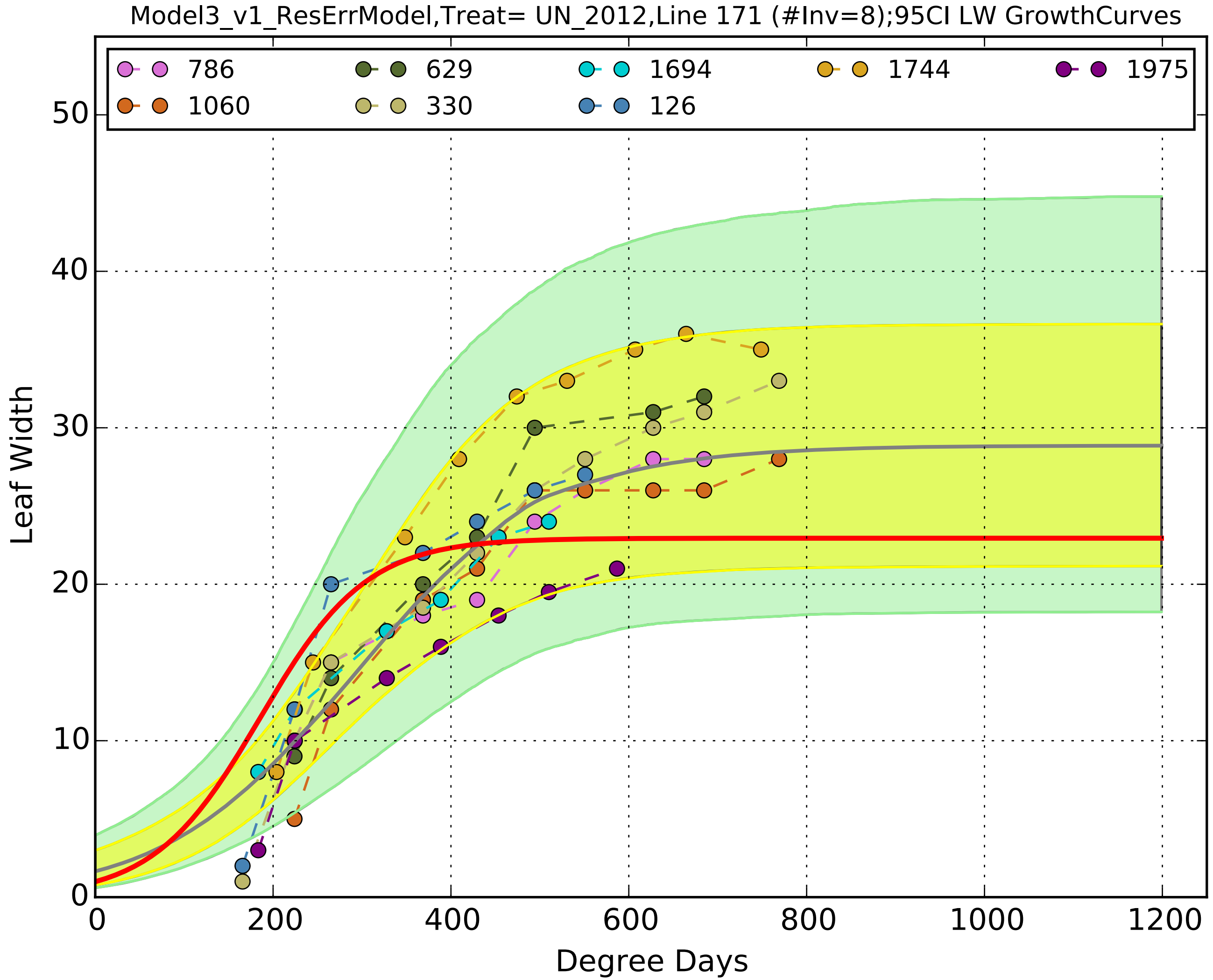
Model3_v1_ResErrModel,Treat= UN_2012,Line 171 (#Inv=8);95CI LW GrowthCurves
786
629
1694
1744
1975
1060
330
126
Leaf Width
Degree Days
0
10
20
30
40
50
0
200
400
600
800
1000
1200

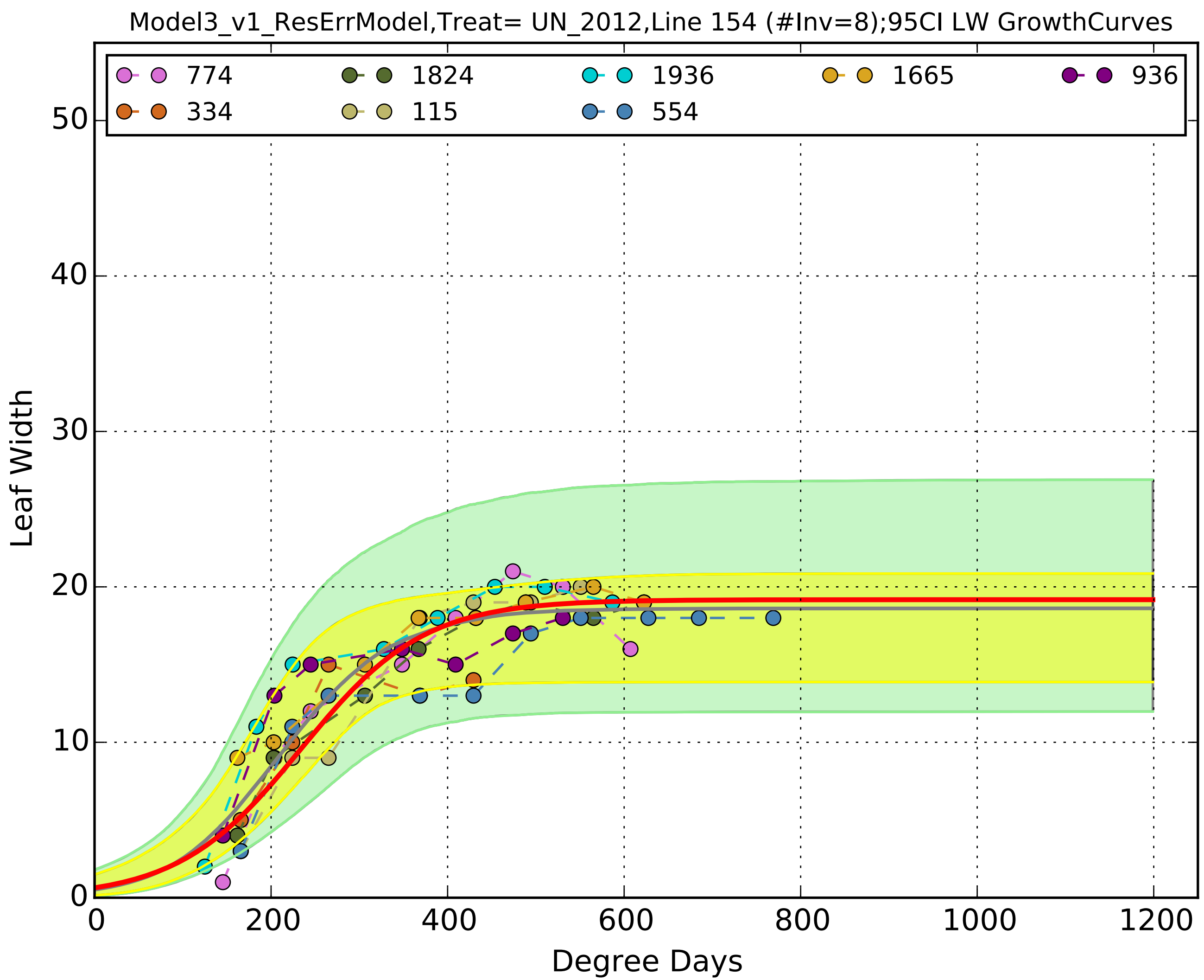
Model3_v1_ResErrModel,Treat= UN_2012,Line 154 (#Inv=8);95CI LW GrowthCurves
774
1824
1936
1665
936
334
115
554
Leaf Width
Degree Days
0
10
20
30
40
50
0
200
400
600
800
1000
1200

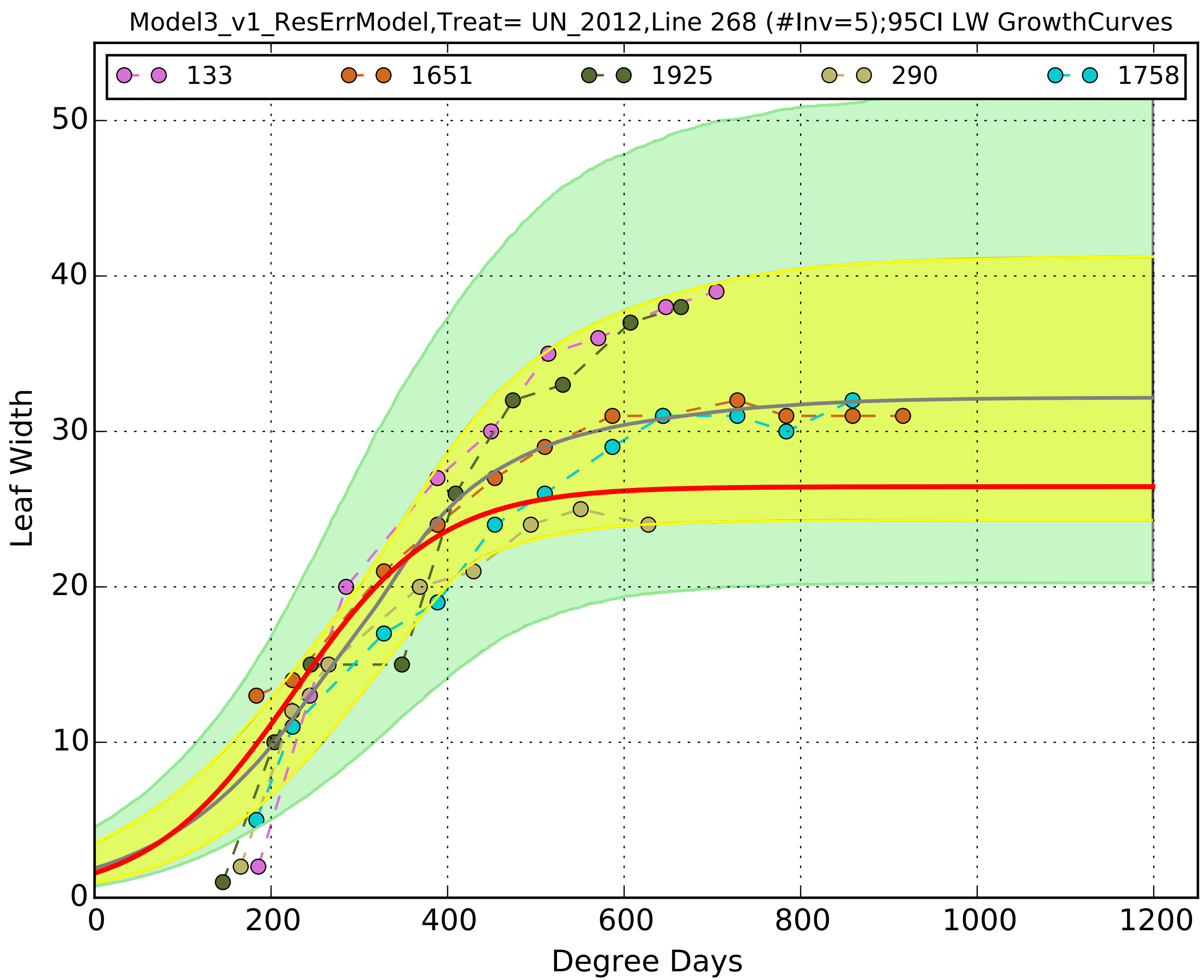
Model3_v1_ResErrModel,Treat= UN_2012,Line 268 (#Inv=5);95CI LW GrowthCurves
133
1651
1925
290
1758
Leaf Width
Degree Days
0
10
20
30
40
50
0
200
400
600
800
1000
1200

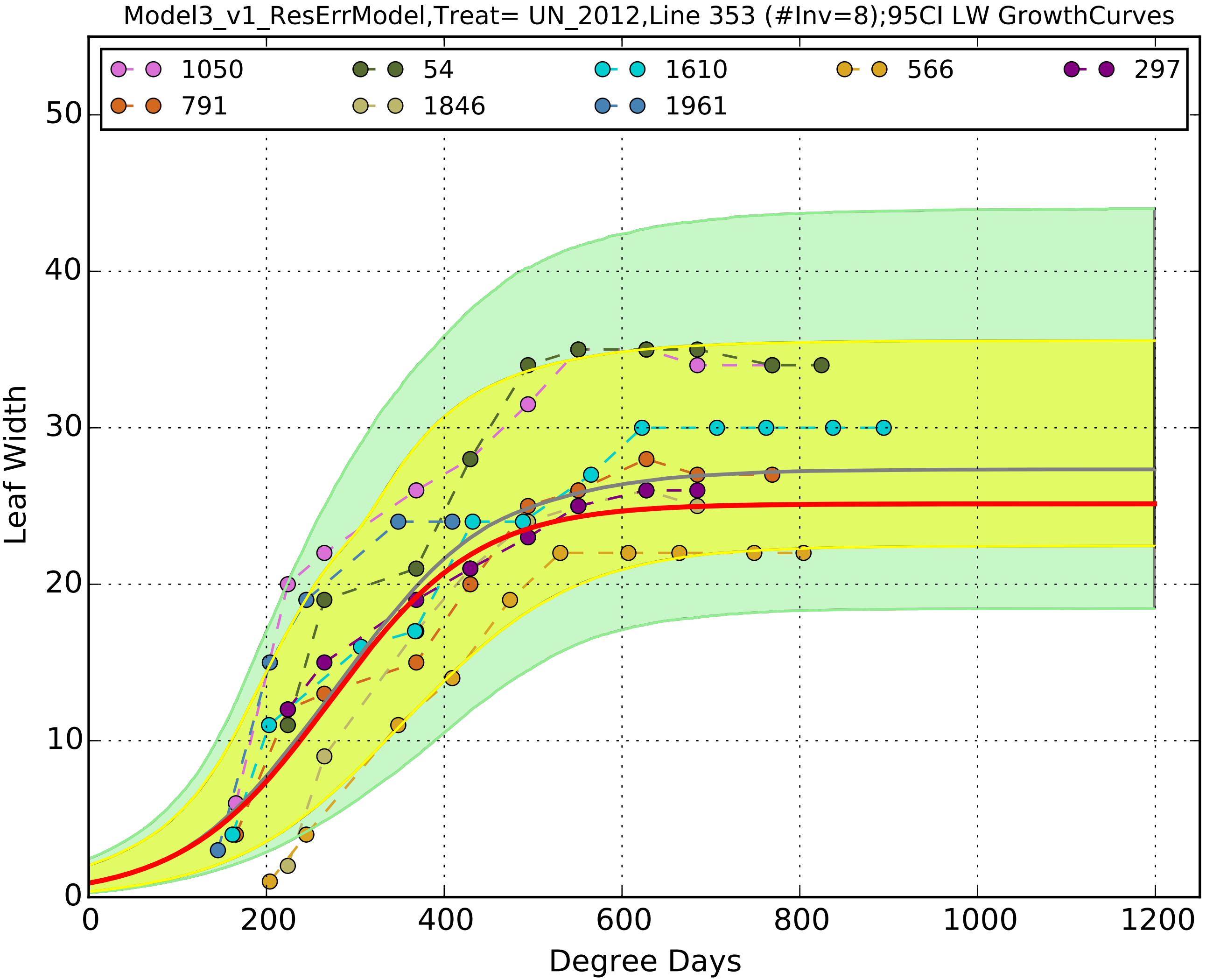

Model3_v1_ResErrModel,Treat= UN_2012,Line 353 (#Inv=8);95CI LW GrowthCurves
1050
54
1610
566
297
791
1846
1961
Leaf Width
Degree Days
0
10
20
30
40
50
0
200
400
600
800
1000
1200

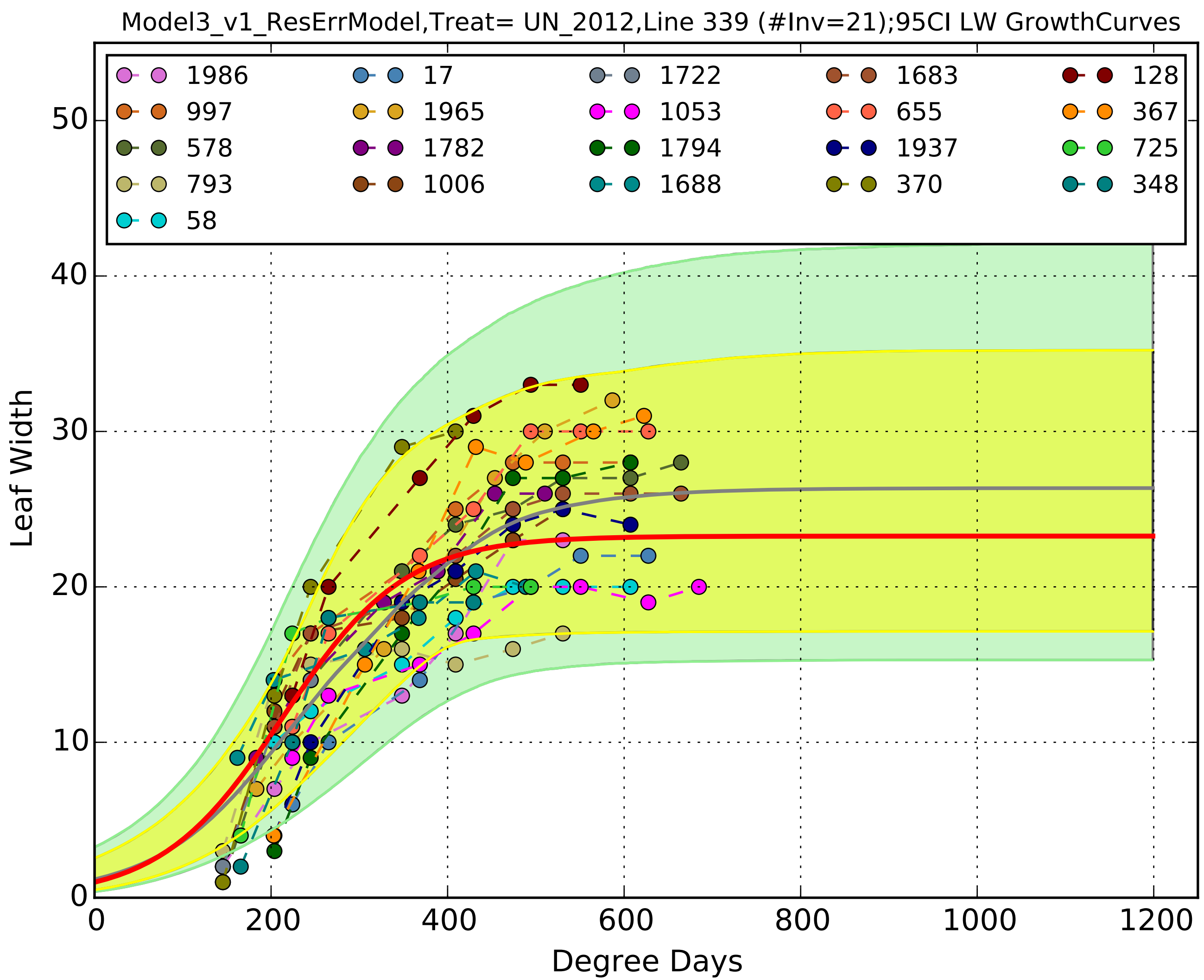

Model3_v1_ResErrModel,Treat= UN_2012,Line 339 (#Inv=21);95CI LW GrowthCurves
1986
17
1722
1683
128
997
1965
1053
655
367
578
1782
1794
1937
725
793
1006
1688
370
348
58
Leaf Width
Degree Days
0
10
20
30
40
50
0
200
400
600
800
1000
1200

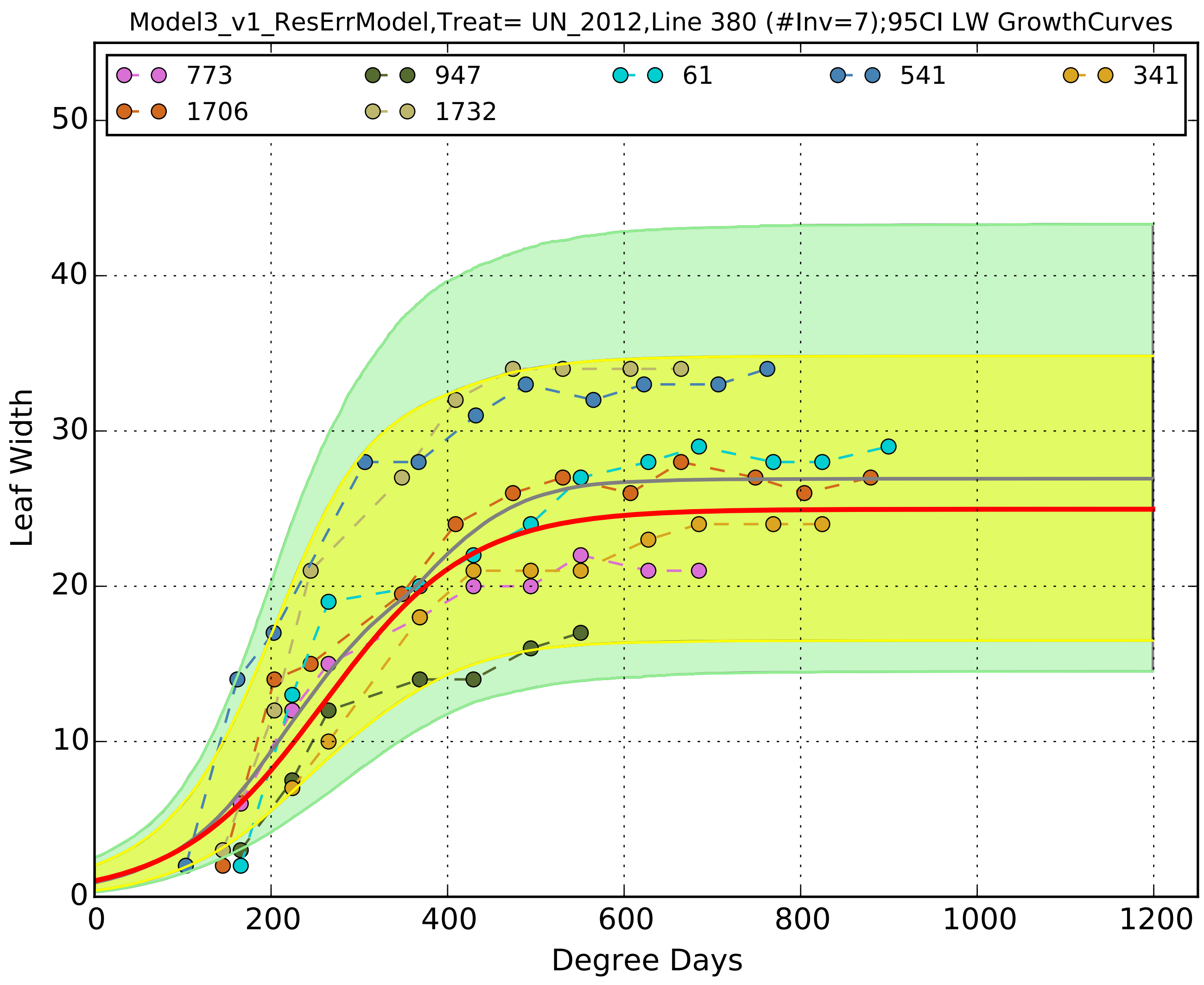
Model3_v1_ResErrModel,Treat= UN_2012,Line 380 (#Inv=7);95CI LW GrowthCurves
773
947
61
541
341
1706
1732
Leaf Width
Degree Days
0
10
20
30
40
50
0
200
400
600
800
1000
1200

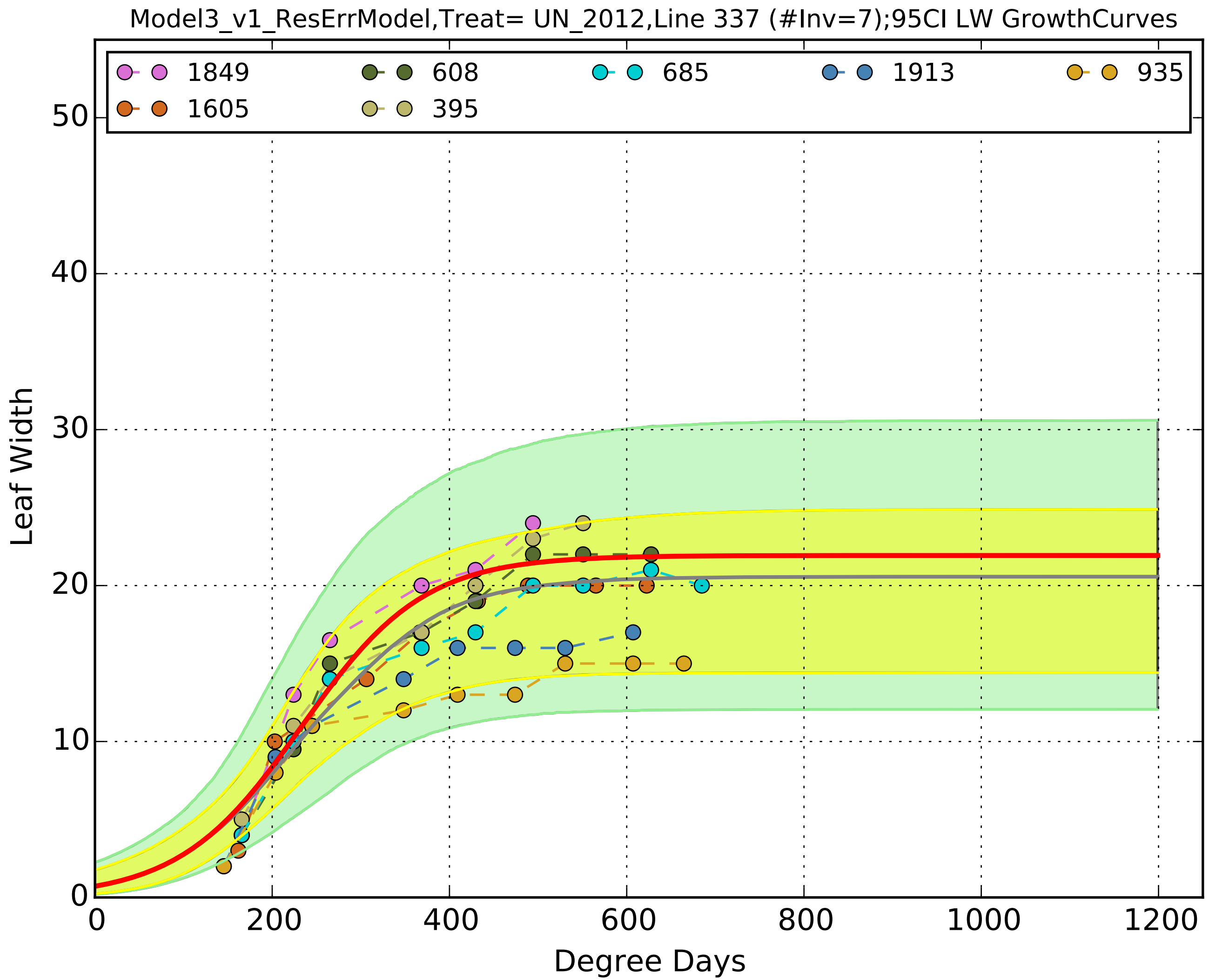

Model3_v1_ResErrModel,Treat= UN_2012,Line 337 (#Inv=7);95CI LW GrowthCurves
1849
608
685
1913
935
1605
395
Leaf Width
Degree Days
0
10
20
30
40
50
0
200
400
600
800
1000
1200

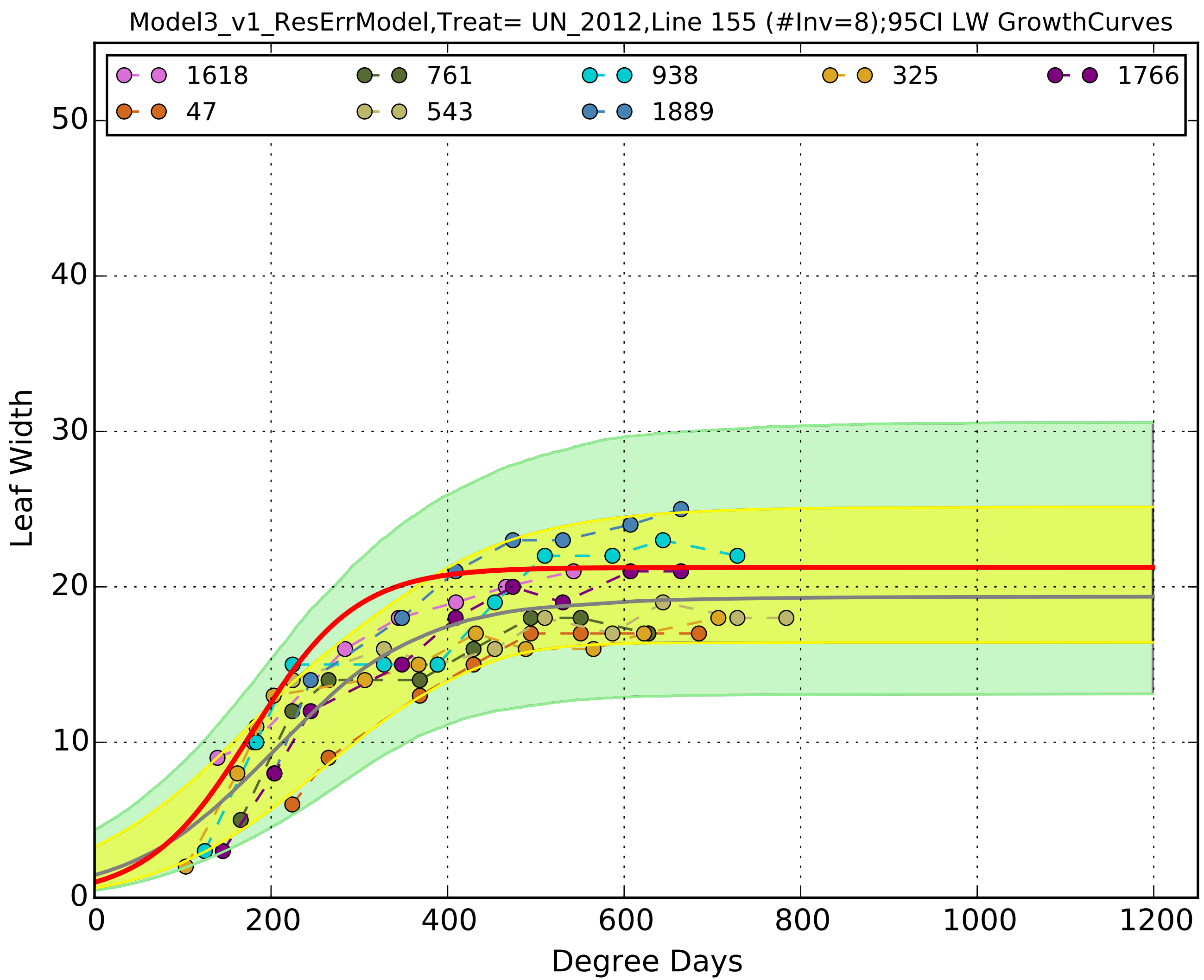

Model3_v1_ResErrModel,Treat= UN_2012,Line 155 (#Inv=8);95CI LW GrowthCurves
1618
761
938
325
1766
47
543
1889
Leaf Width
Degree Days
0
10
20
30
40
50
0
200
400
600
800
1000
1200

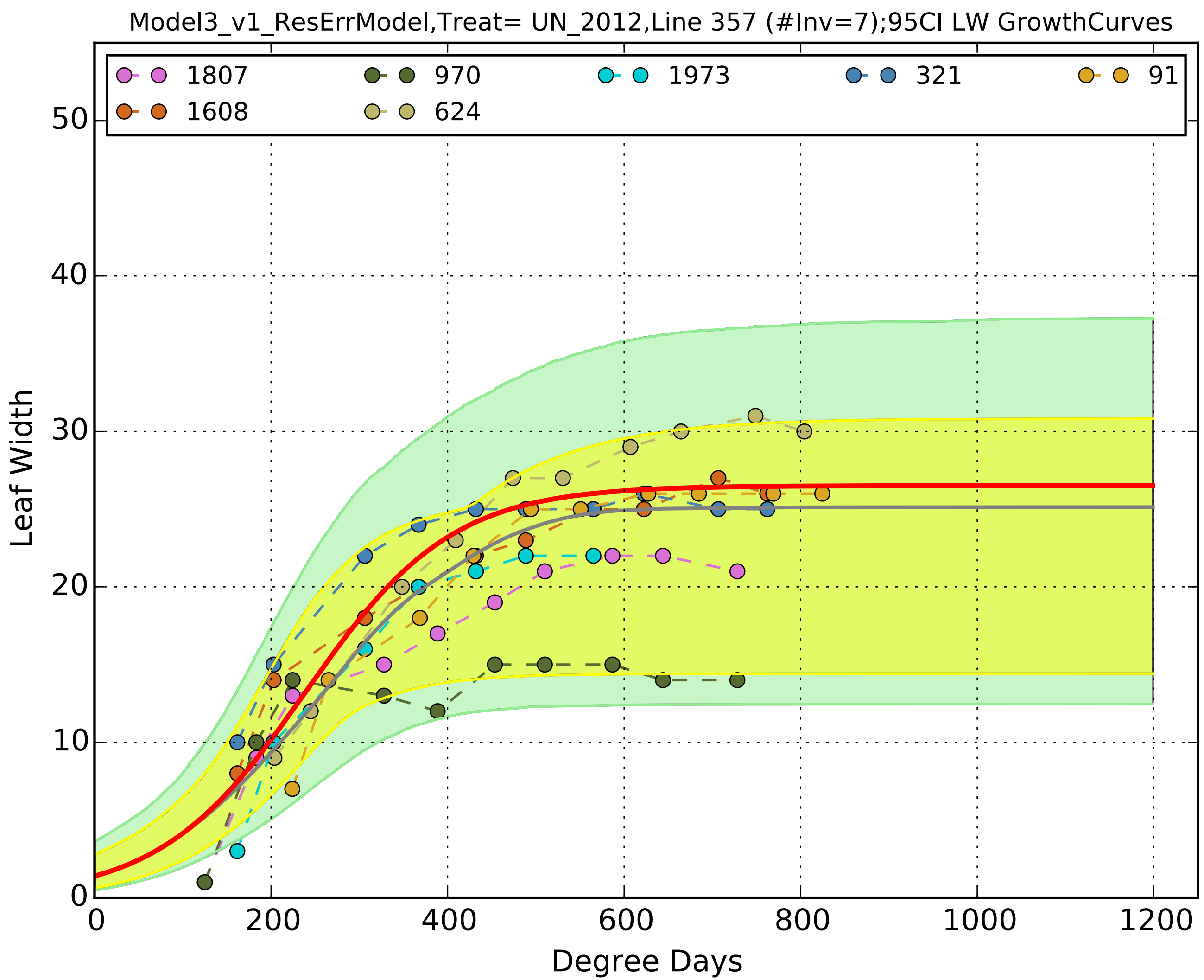

Model3_v1_ResErrModel,Treat= UN_2012,Line 357 (#Inv=7);95CI LW GrowthCurves
1807
970
1973
321
91
1608
624
Leaf Width
Degree Days
0
10
20
30
40
50
0
200
400
600
800
1000
1200

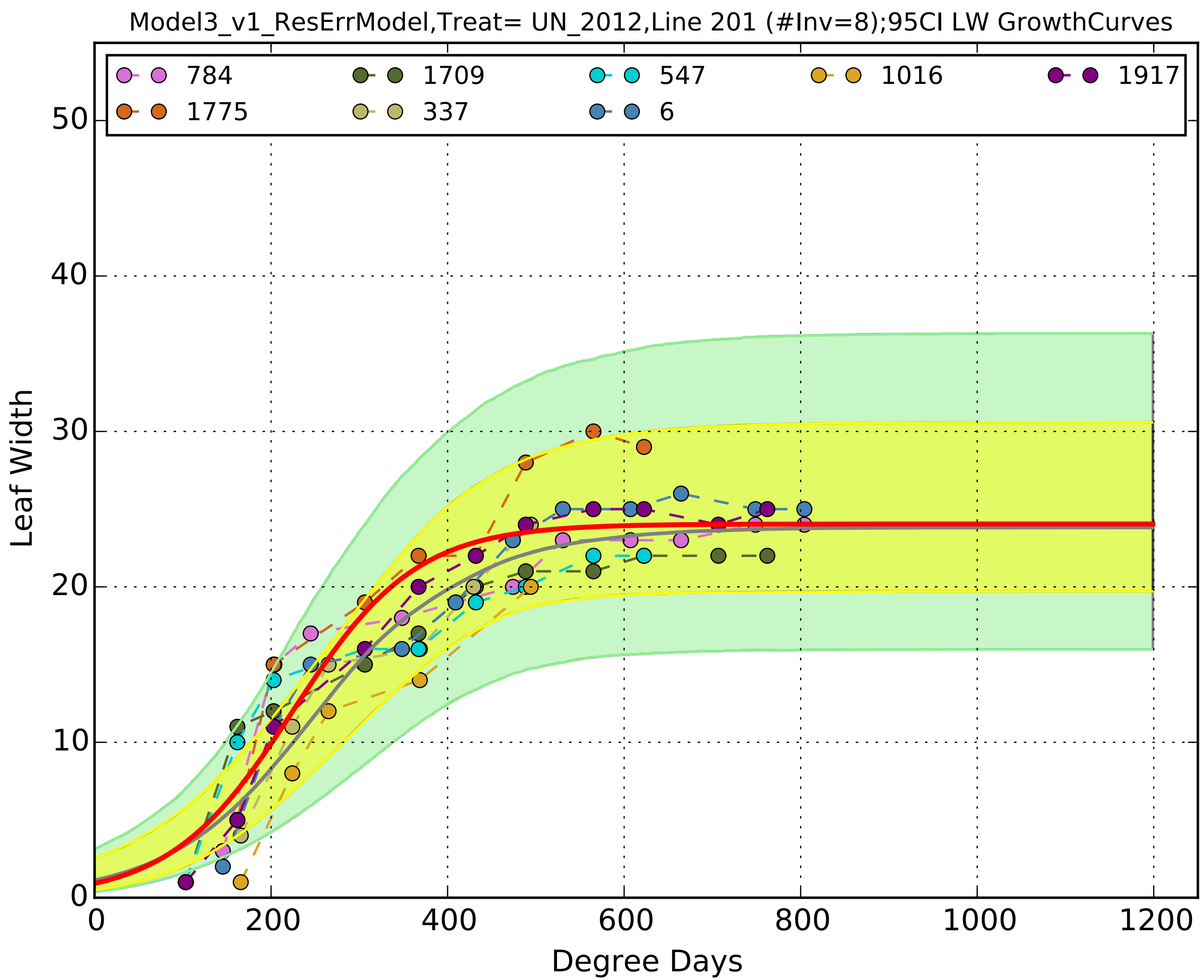

Model3_v1_ResErrModel,Treat= UN_2012,Line 201 (#Inv=8);95CI LW GrowthCurves
784
1709
547
1016
1917
1775
337
6
Leaf Width
Degree Days
0
10
20
30
40
50
0
200
400
600
800
1000
1200

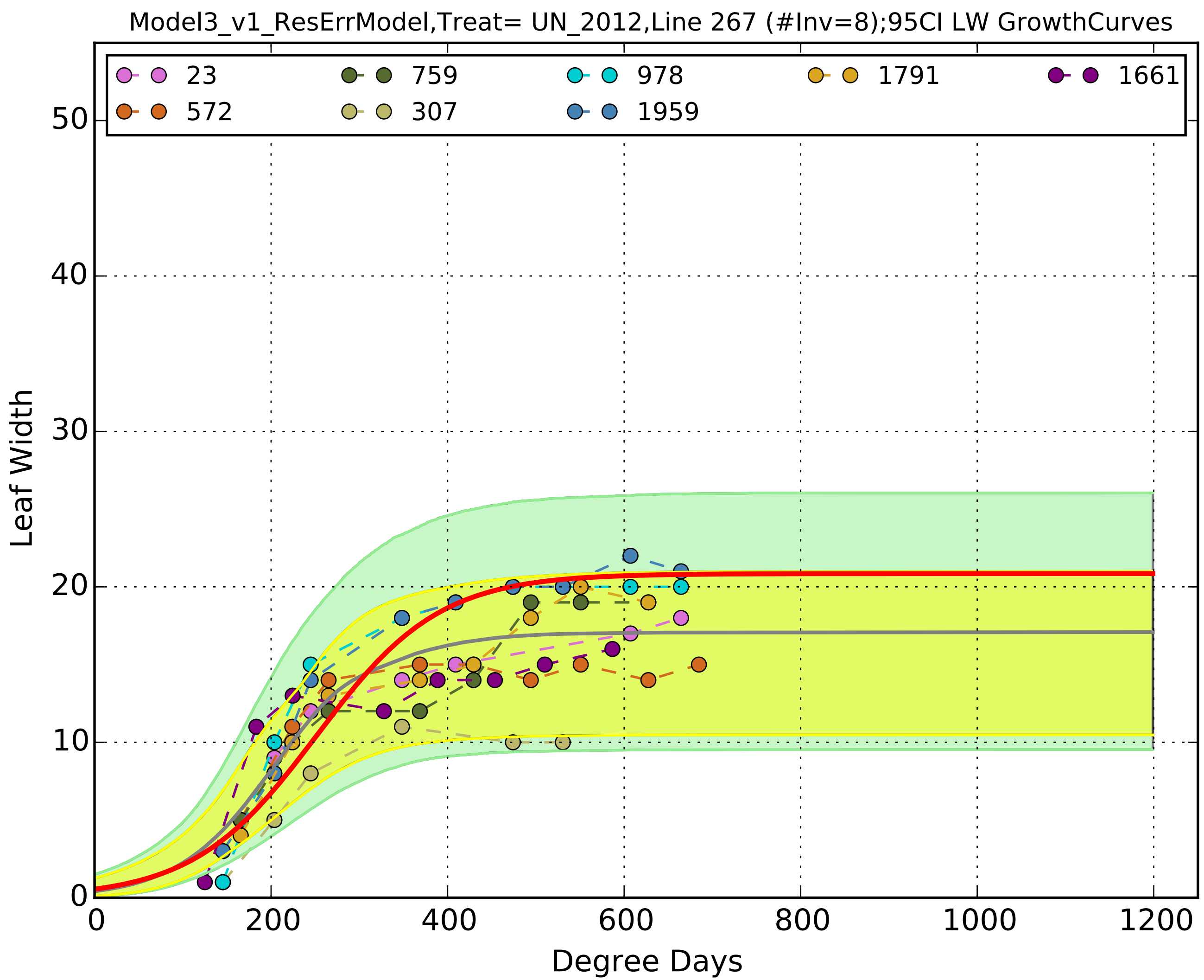

Model3_v1_ResErrModel,Treat= UN_2012,Line 267 (#Inv=8);95CI LW GrowthCurves
23
572
759
307
978
1959
1791
1661
Leaf Width
Degree Days
0
10
20
30
40
50
0
200
400
600
800
1000
1200

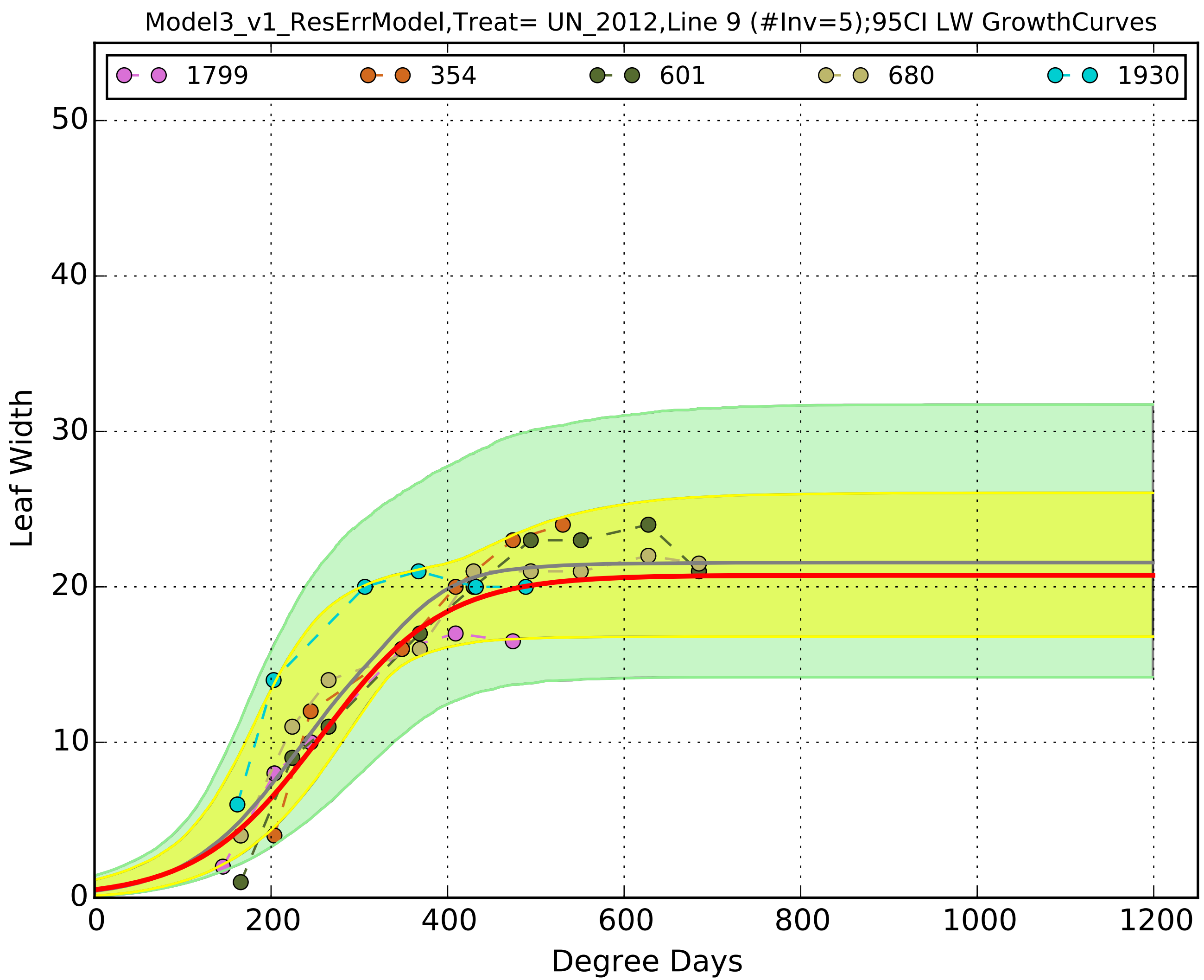

Model3_v1_ResErrModel,Treat= UN_2012,Line 9 (#Inv=5);95CI LW GrowthCurves
1799
354
601
680
1930
Leaf Width
Degree Days
0
10
20
30
40
50
0
200
400
600
800
1000
1200

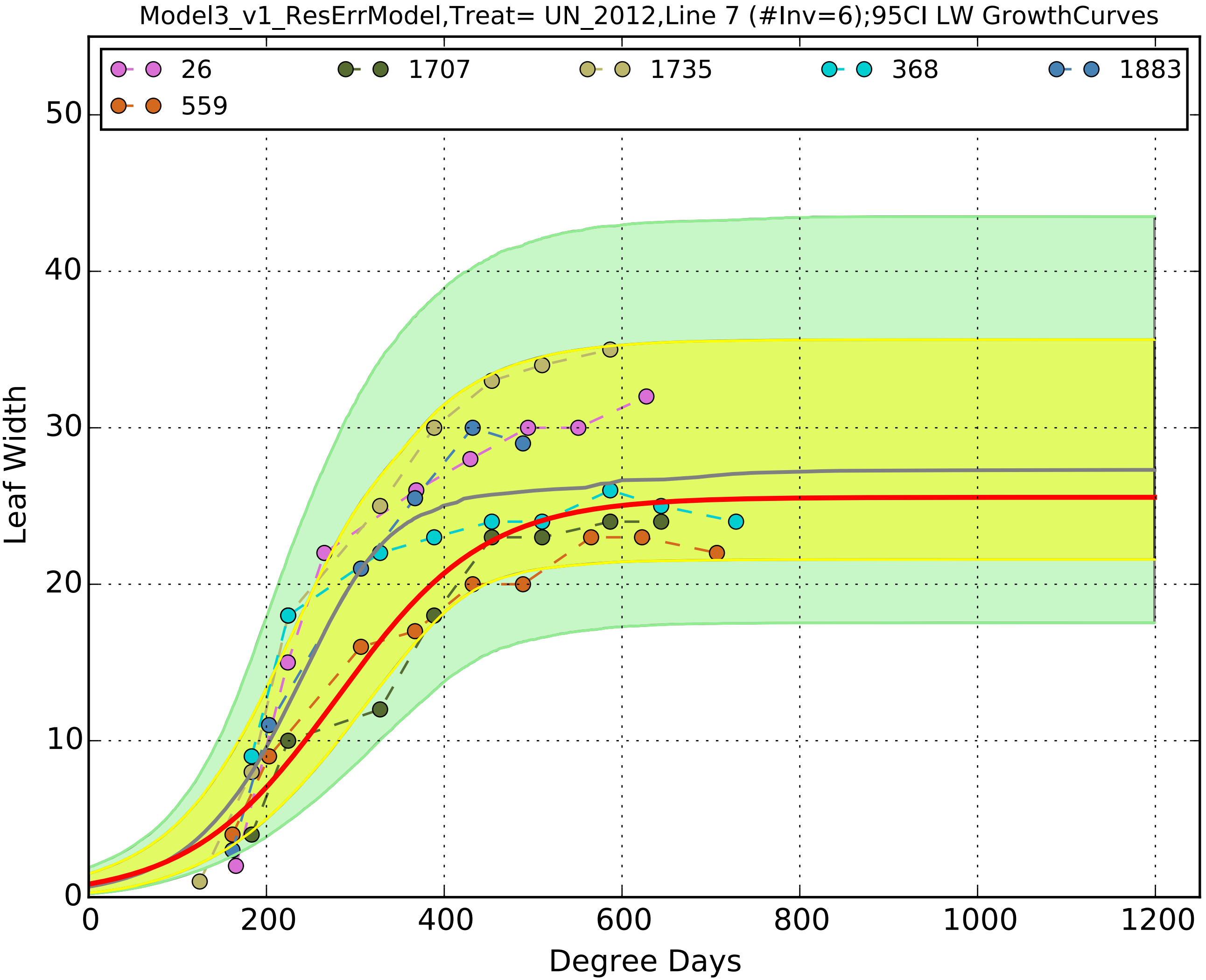

Model3_v1_ResErrModel,Treat= UN_2012,Line 7 (#Inv=6);95CI LW GrowthCurves
26
1707
1735
368
1883
559
Leaf Width
Degree Days
0
10
20
30
40
50
0
200
400
600
800
1000
1200

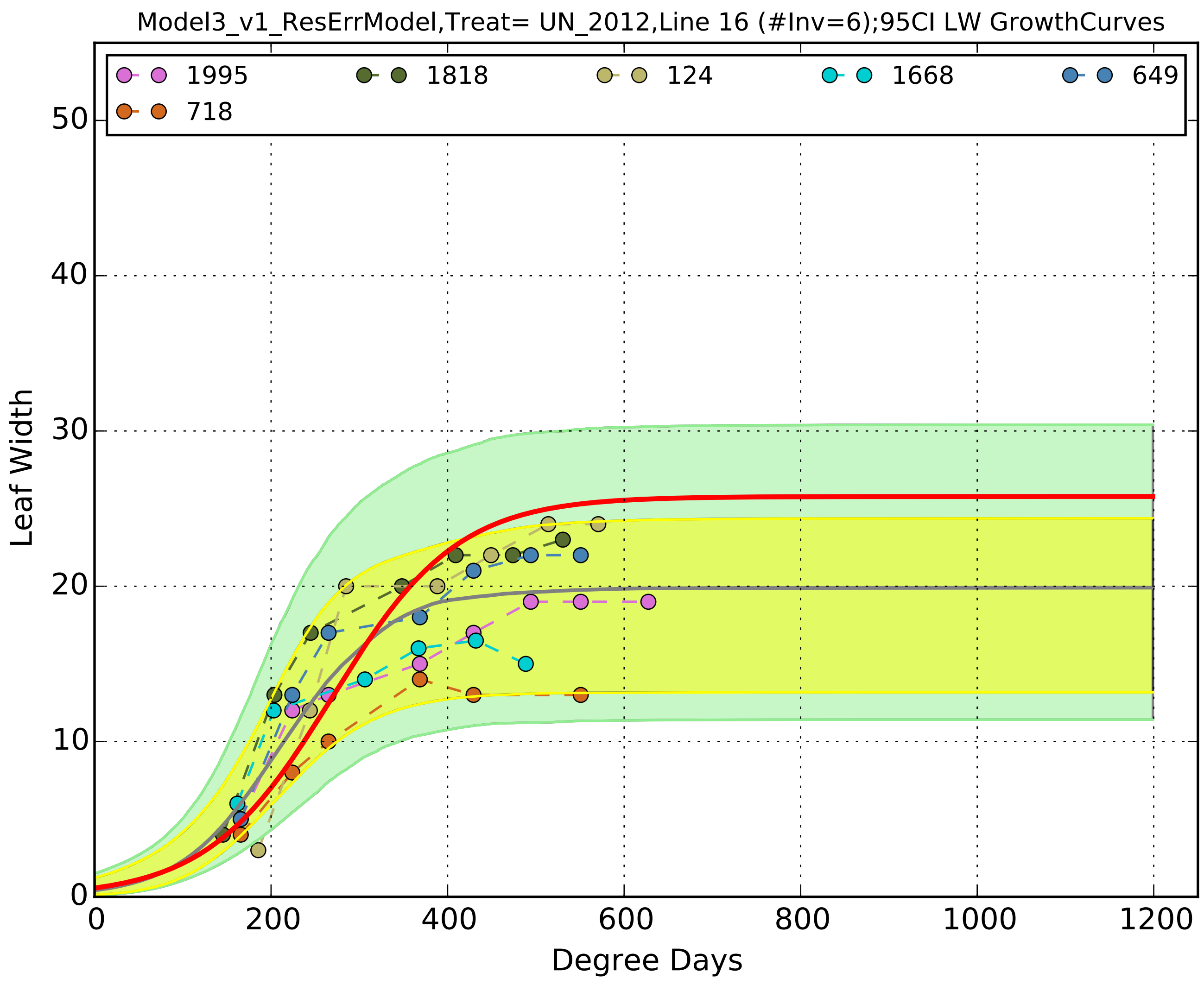

Model3_v1_ResErrModel,Treat= UN_2012,Line 16 (#Inv=6);95CI LW GrowthCurves
1995
1818
124
1668
649
718
Leaf Width
Degree Days
0
10
20
30
40
50
0
200
400
600
800
1000
1200

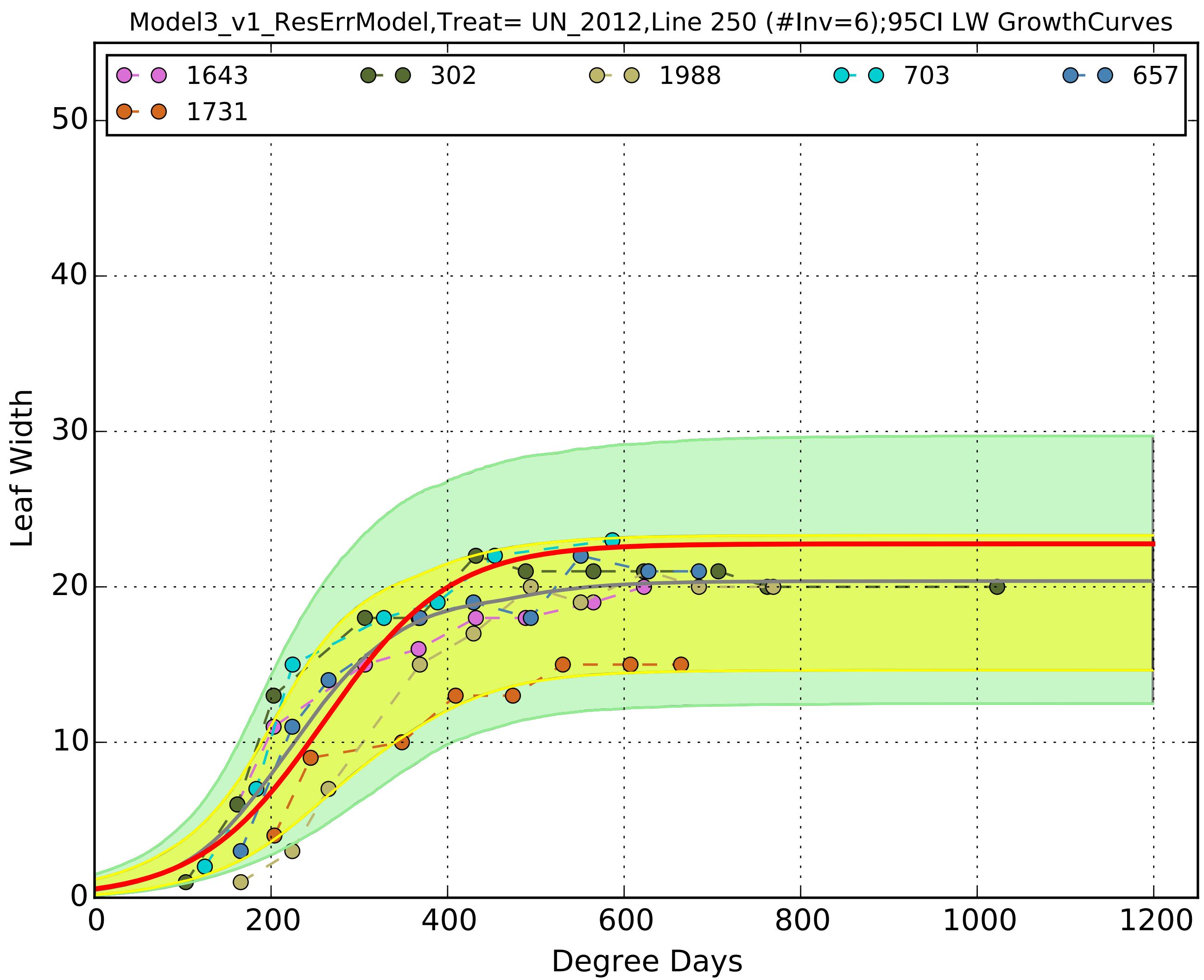

Model3_v1_ResErrModel,Treat= UN_2012,Line 250 (#Inv=6);95CI LW GrowthCurves
1643
302
1988
703
657
1731
Leaf Width
Degree Days
0
10
20
30
40
50
0
200
400
600
800
1000
1200

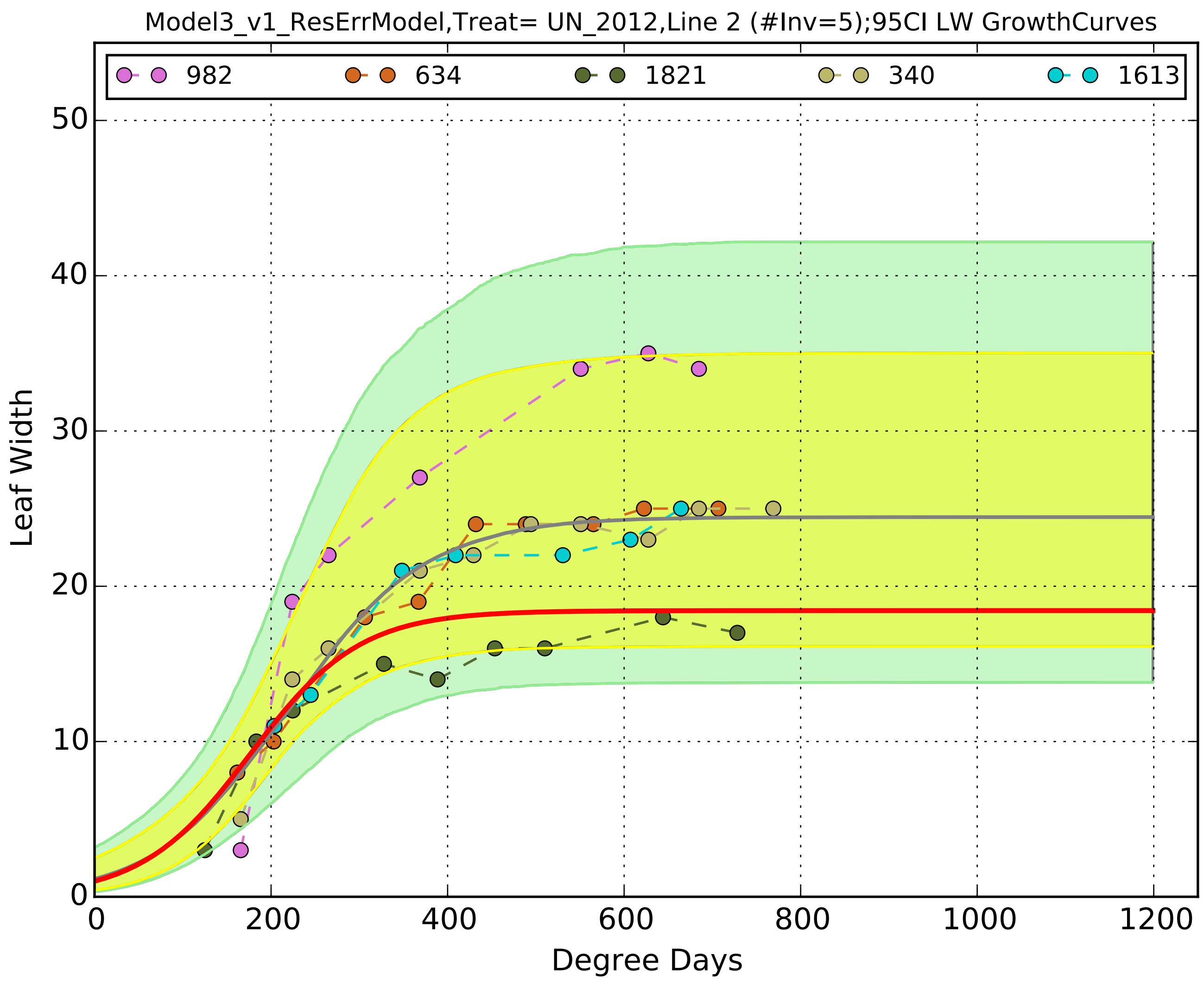

Model3_v1_ResErrModel,Treat= UN_2012,Line 2 (#Inv=5);95CI LW GrowthCurves
982
634
1821
340
1613
Leaf Width
0
10
20
30
40
50
0
200
400
600
800
1000
1200
Degree Days

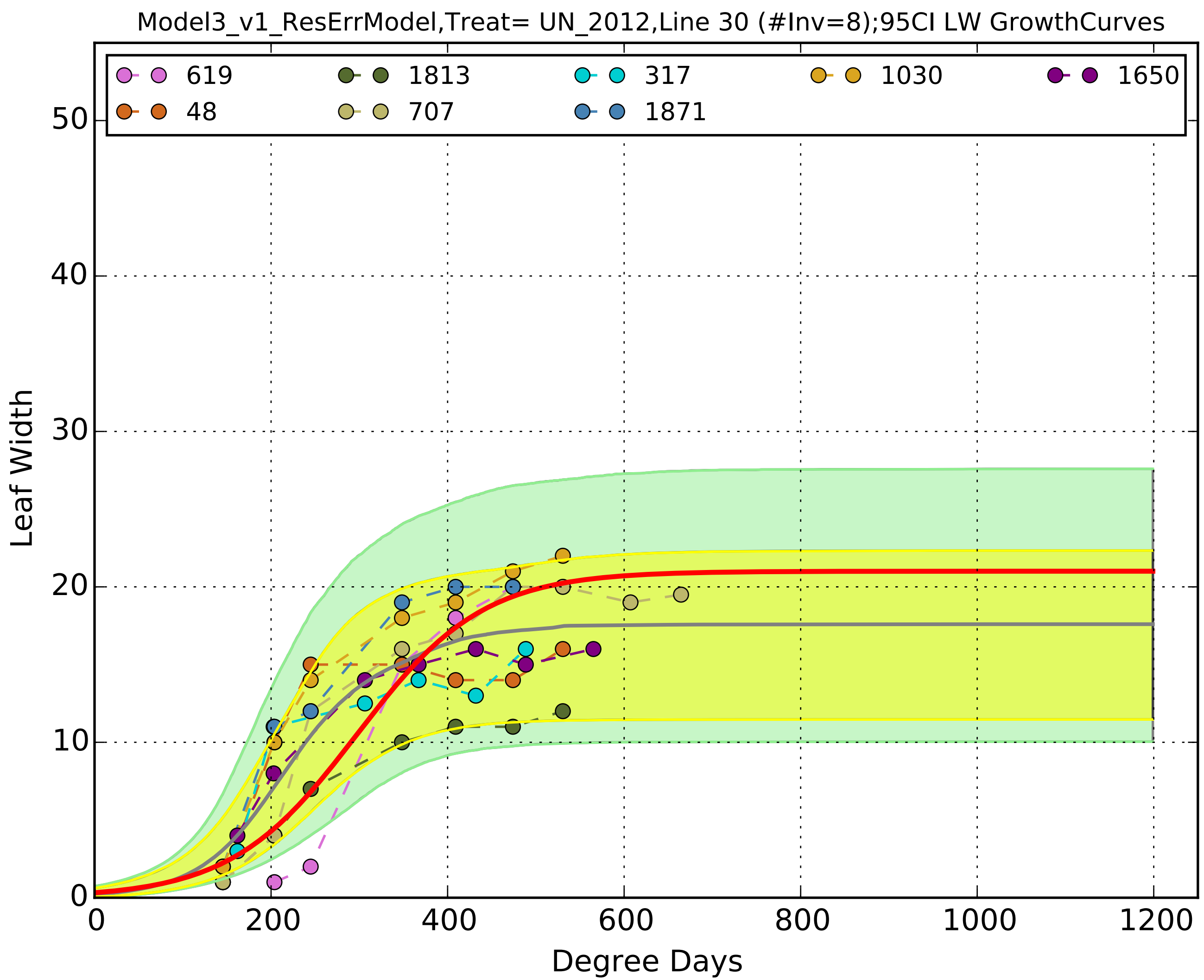
Model3_v1_ResErrModel,Treat= UN_2012,Line 30 (#Inv=8);95CI LW GrowthCurves
619
1813
317
1030
1650
48
707
1871
Leaf Width
Degree Days
0
10
20
30
40
50
0
200
400
600
800
1000
1200

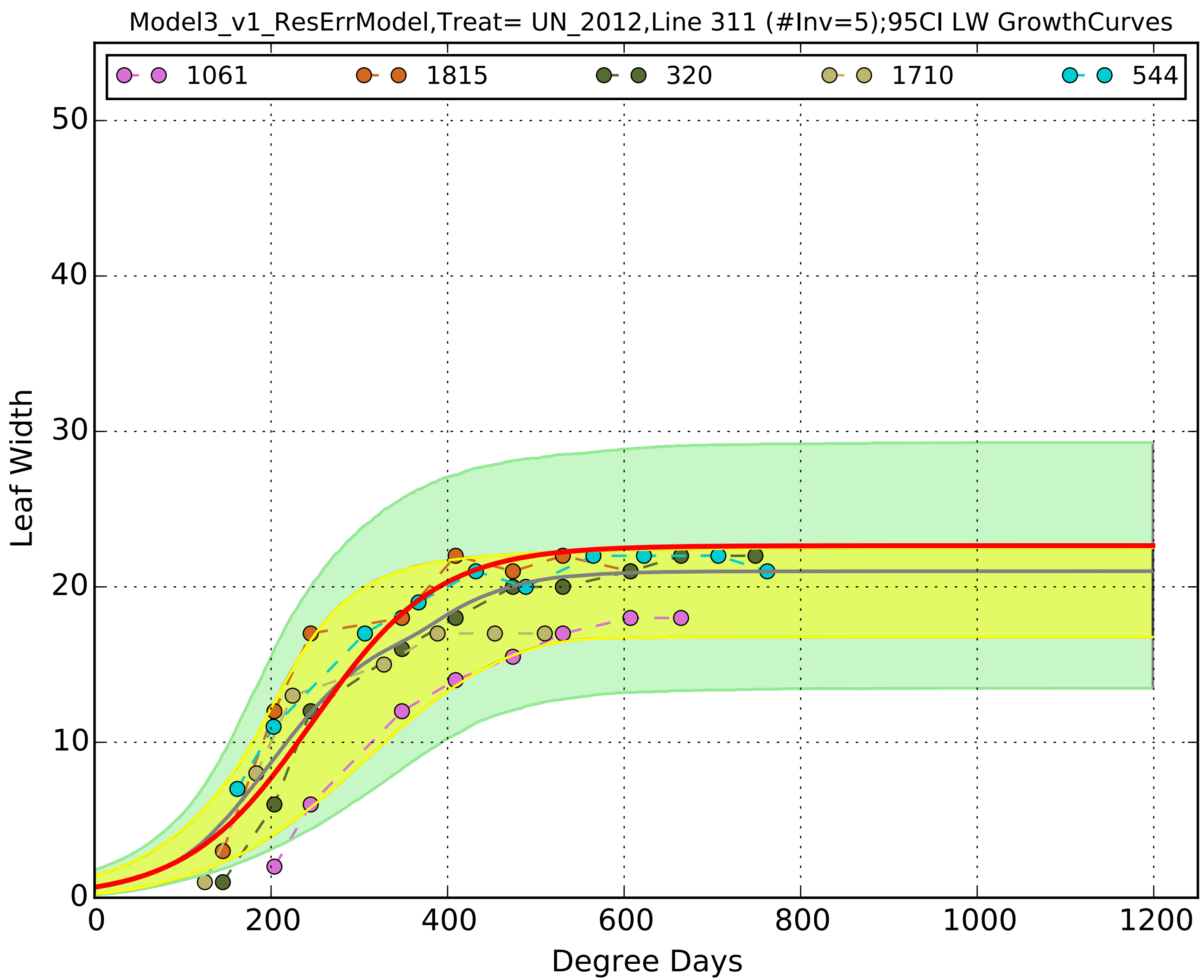

Model3_v1_ResErrModel,Treat= UN_2012,Line 311 (#Inv=5);95CI LW GrowthCurves
1061
1815
320
1710
544
Leaf Width
0
10
20
30
40
50
0
200
400
600
800
1000
1200
Degree Days

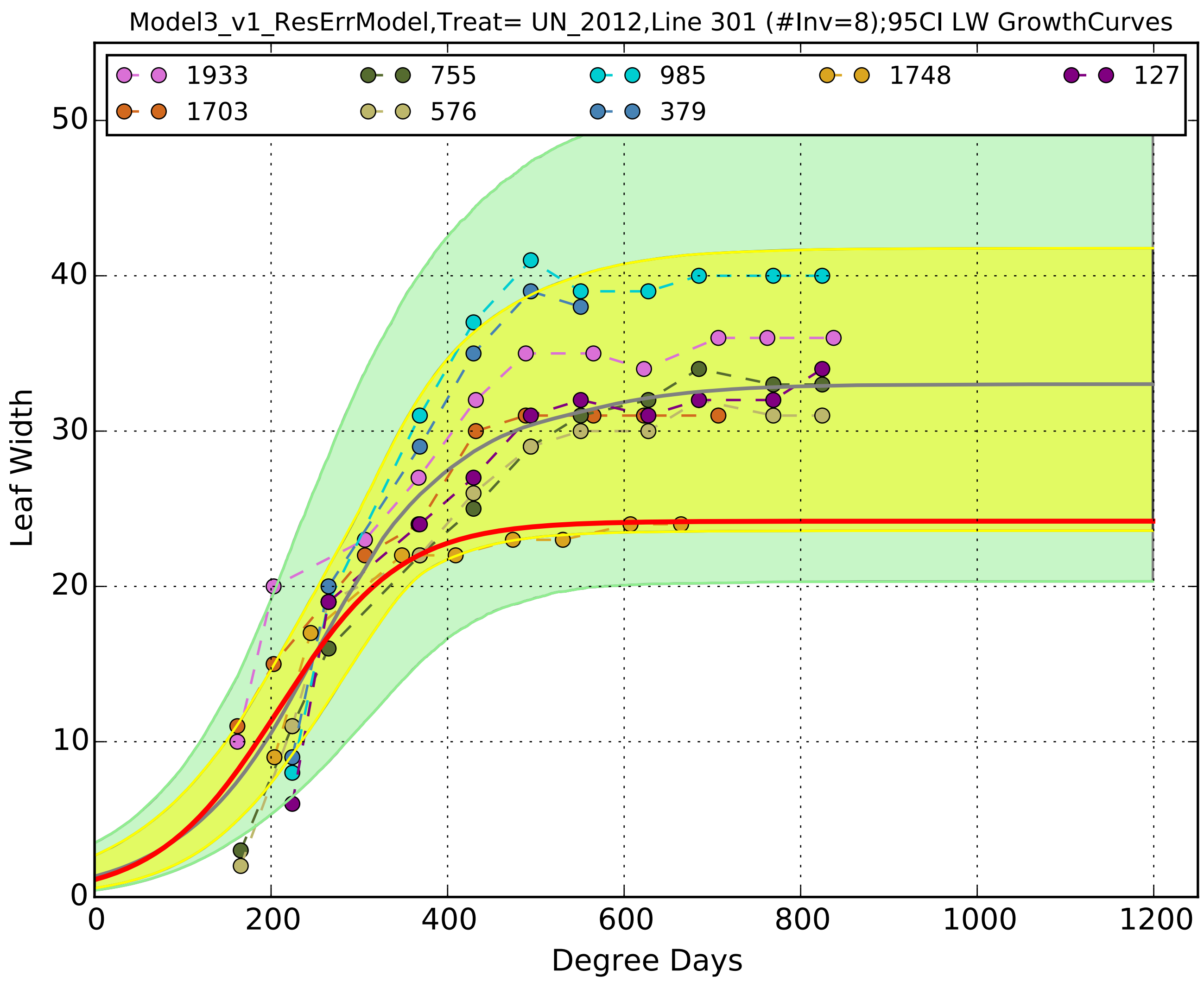
Model3_v1_ResErrModel,Treat= UN_2012,Line 301 (#Inv=8);95CI LW GrowthCurves
1933
755
985
1748
127
1703
576
379
Leaf Width
Degree Days
0
10
20
30
40
50
0
200
400
600
800
1000
1200

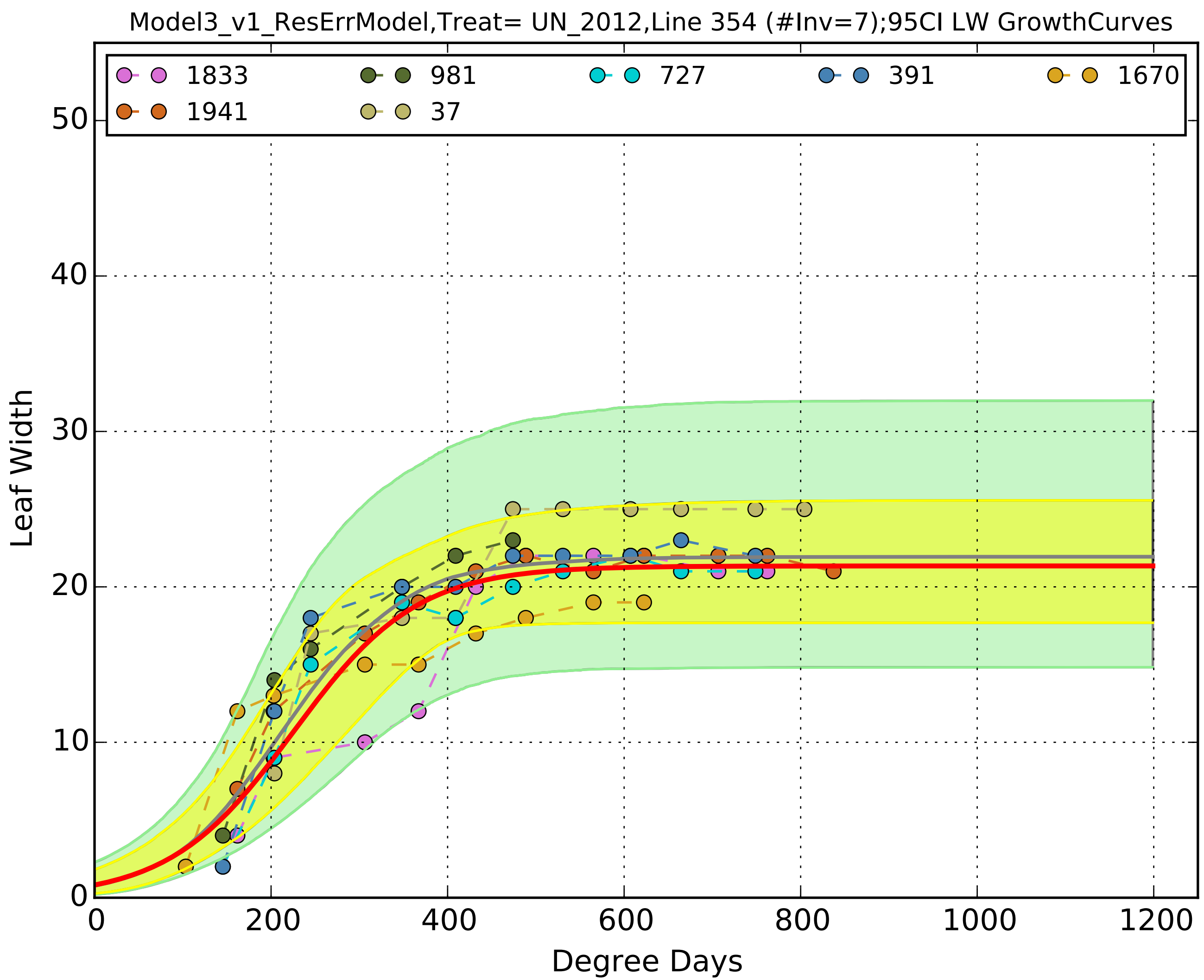
Model3_v1_ResErrModel,Treat= UN_2012,Line 354 (#Inv=7);95CI LW GrowthCurves
1833
981
727
391
1670
1941
37
Leaf Width
Degree Days
0
10
20
30
40
50
0
200
400
600
800
1000
1200

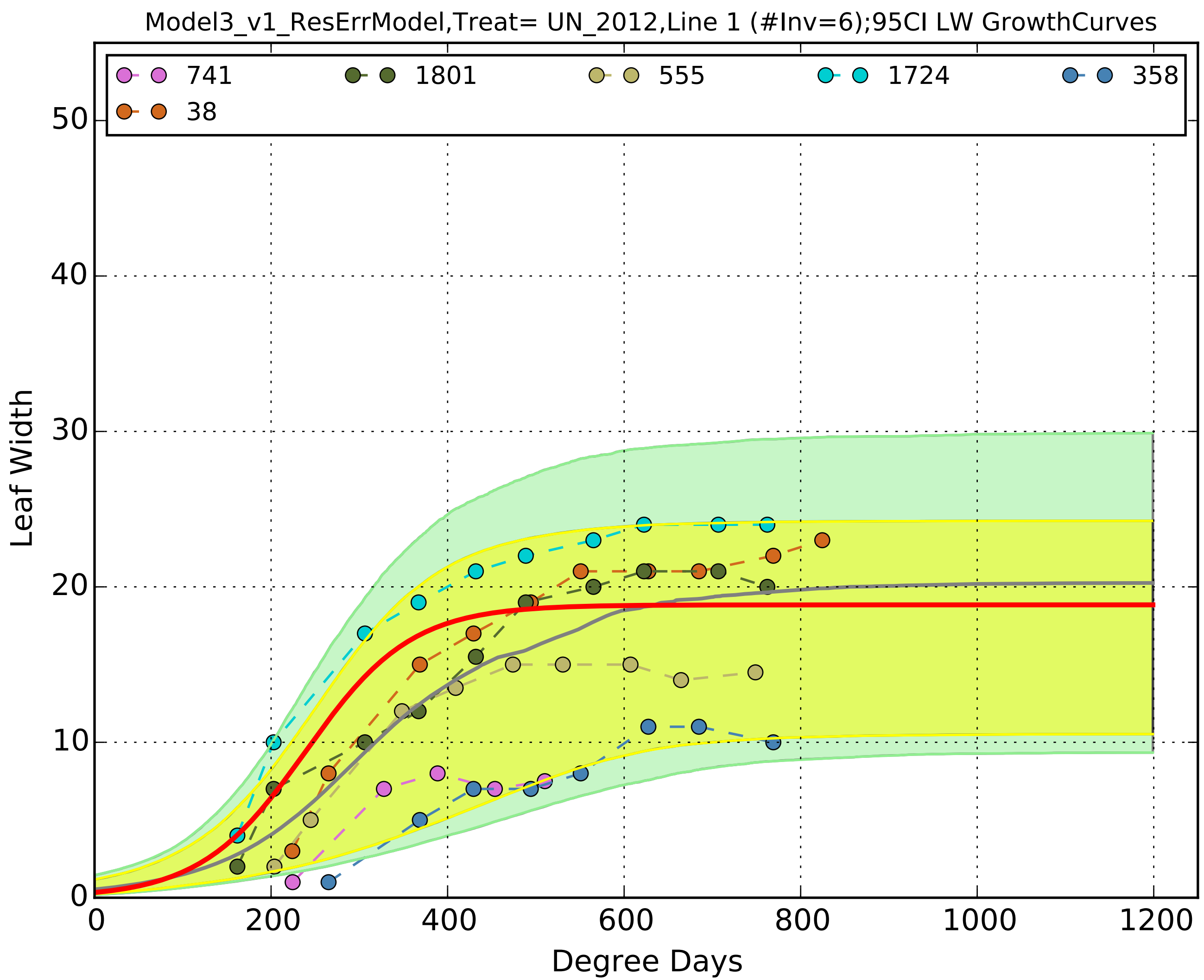

Model3_v1_ResErrModel,Treat= UN_2012,Line 1 (#Inv=6);95CI LW GrowthCurves
741
1801
555
1724
358
38
Leaf Width
Degree Days
0
10
20
30
40
50
0
200
400
600
800
1000
1200

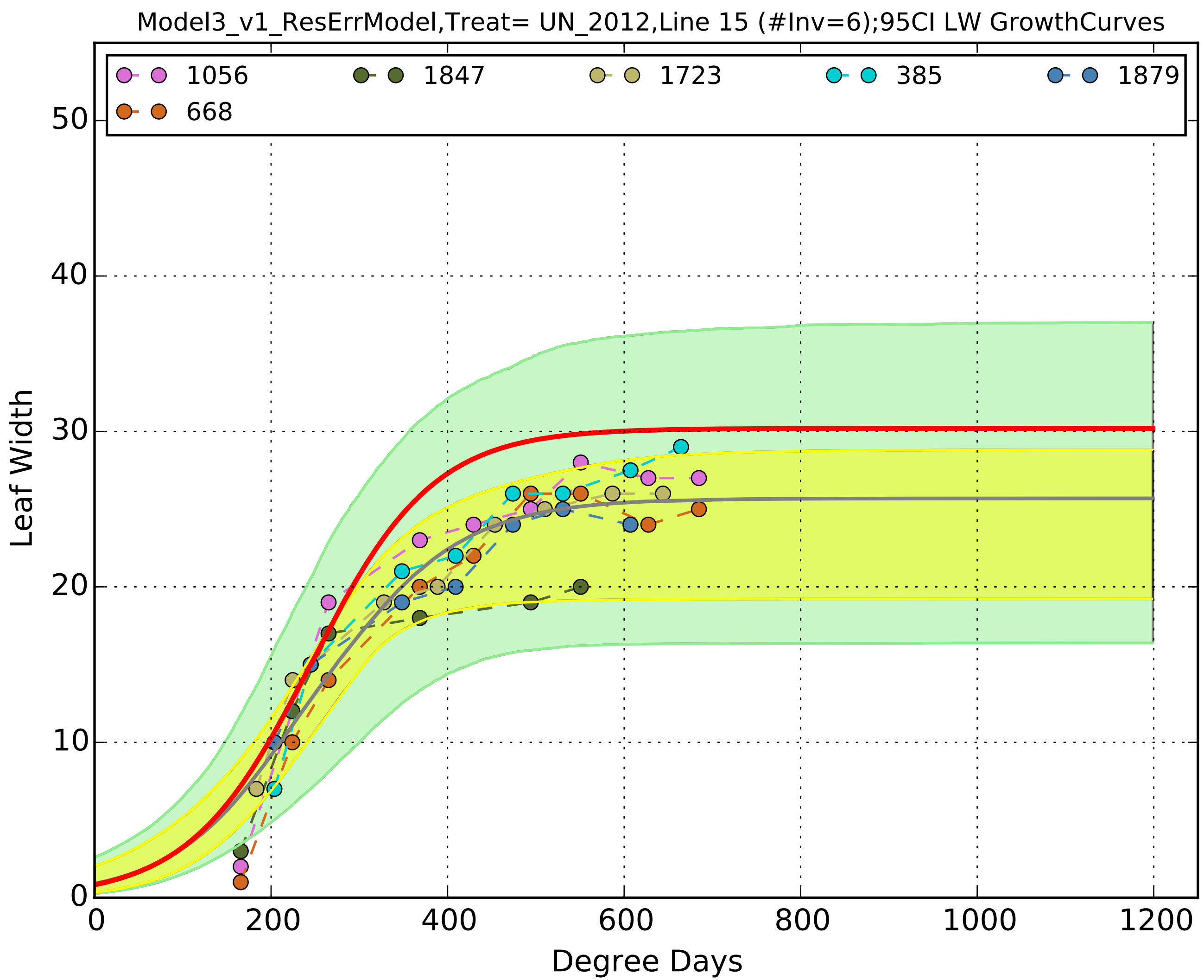
Model3_v1_ResErrModel,Treat= UN_2012,Line 15 (#Inv=6);95CI LW GrowthCurves
1056
1847
1723
385
1879
668
Leaf Width
Degree Days
0
10
20
30
40
50
0
200
400
600
800
1000
1200

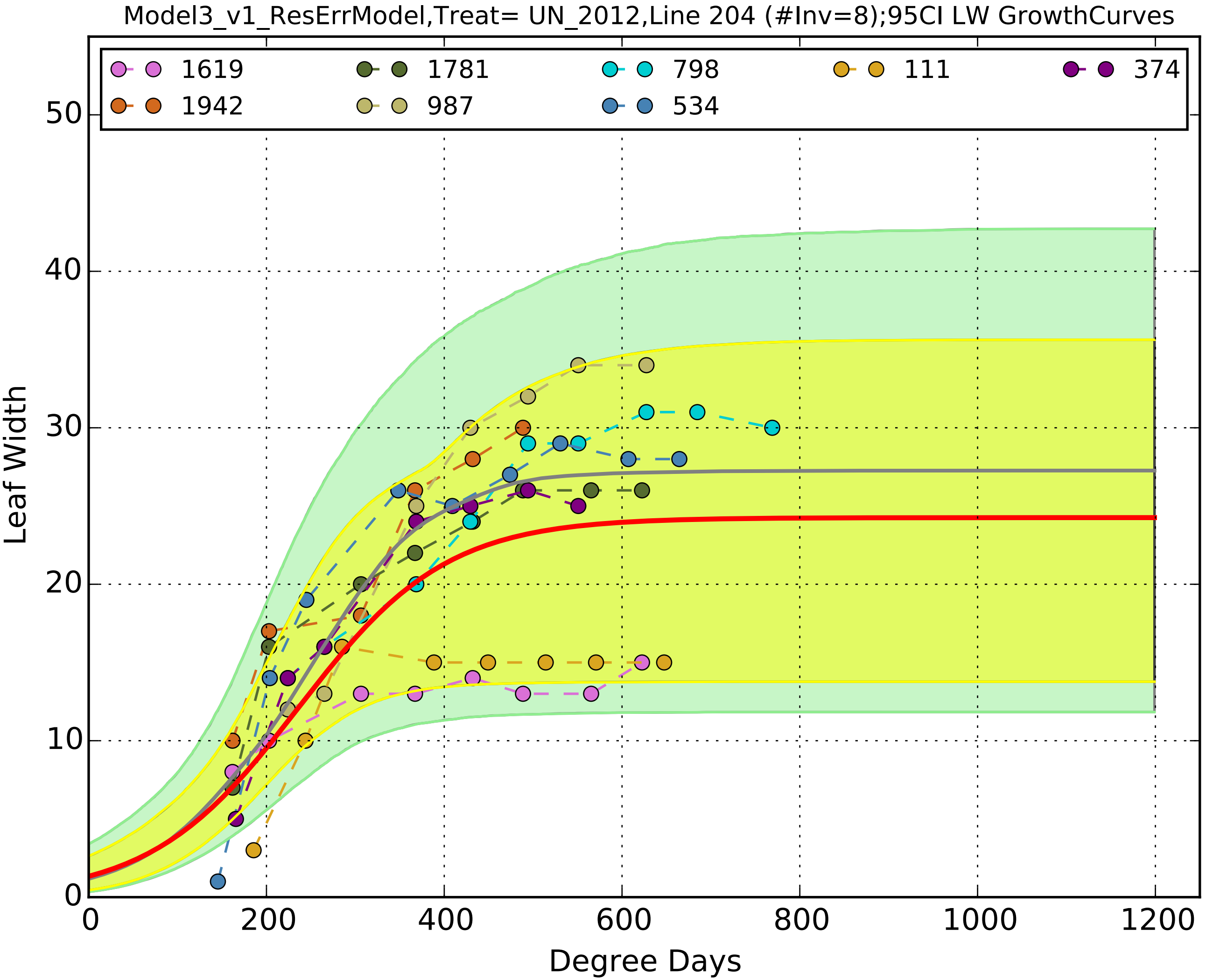

Model3_v1_ResErrModel,Treat= UN_2012,Line 204 (#Inv=8);95CI LW GrowthCurves
1619
1781
798
111
374
1942
987
534
Leaf Width
Degree Days
0
10
20
30
40
50
0
200
400
600
800
1000
1200

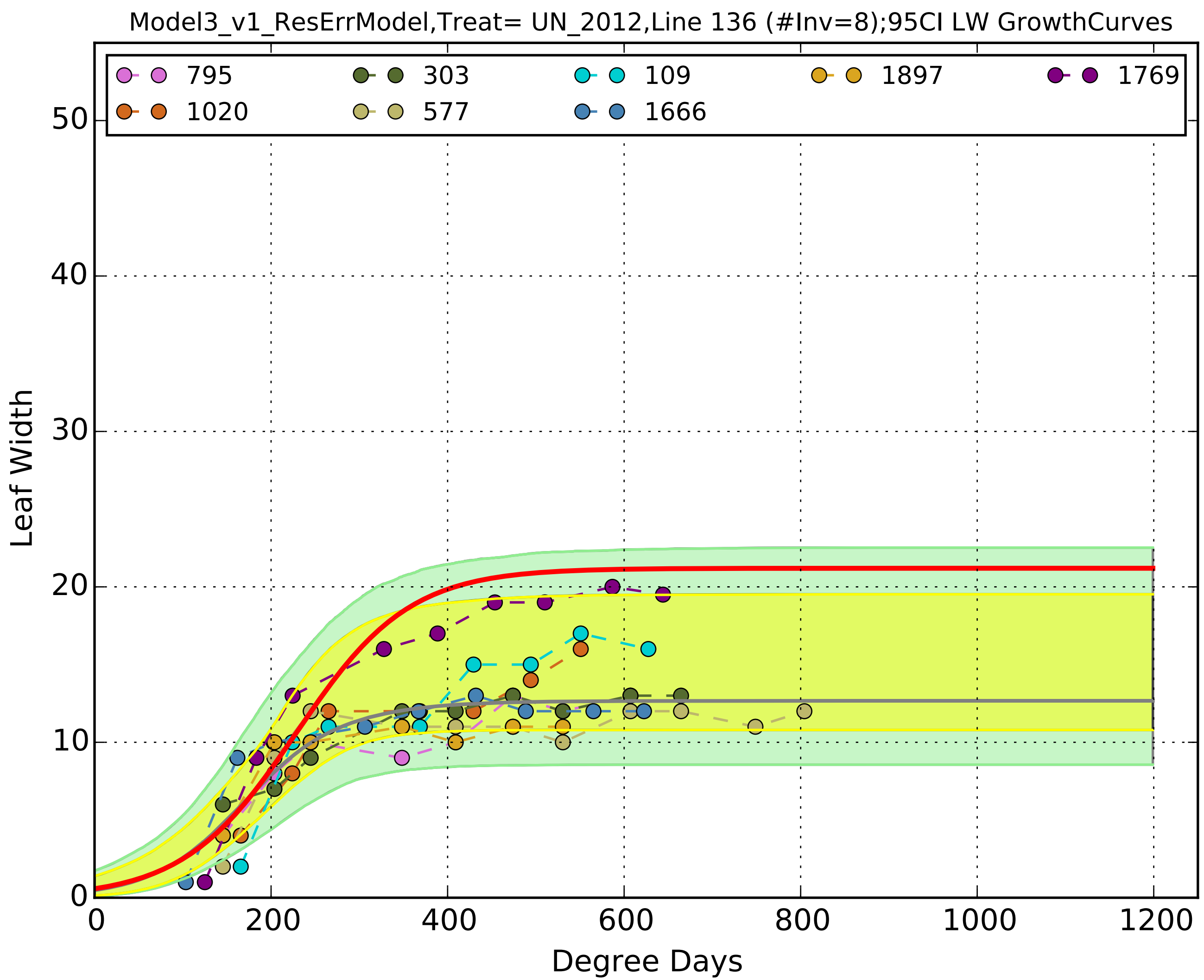

Model3_v1_ResErrModel,Treat= UN_2012,Line 136 (#Inv=8);95CI LW GrowthCurves
795
1020
303
577
109
1666
1897
1769
Leaf Width
Degree Days
0
10
20
30
40
50
0
200
400
600
800
1000
1200

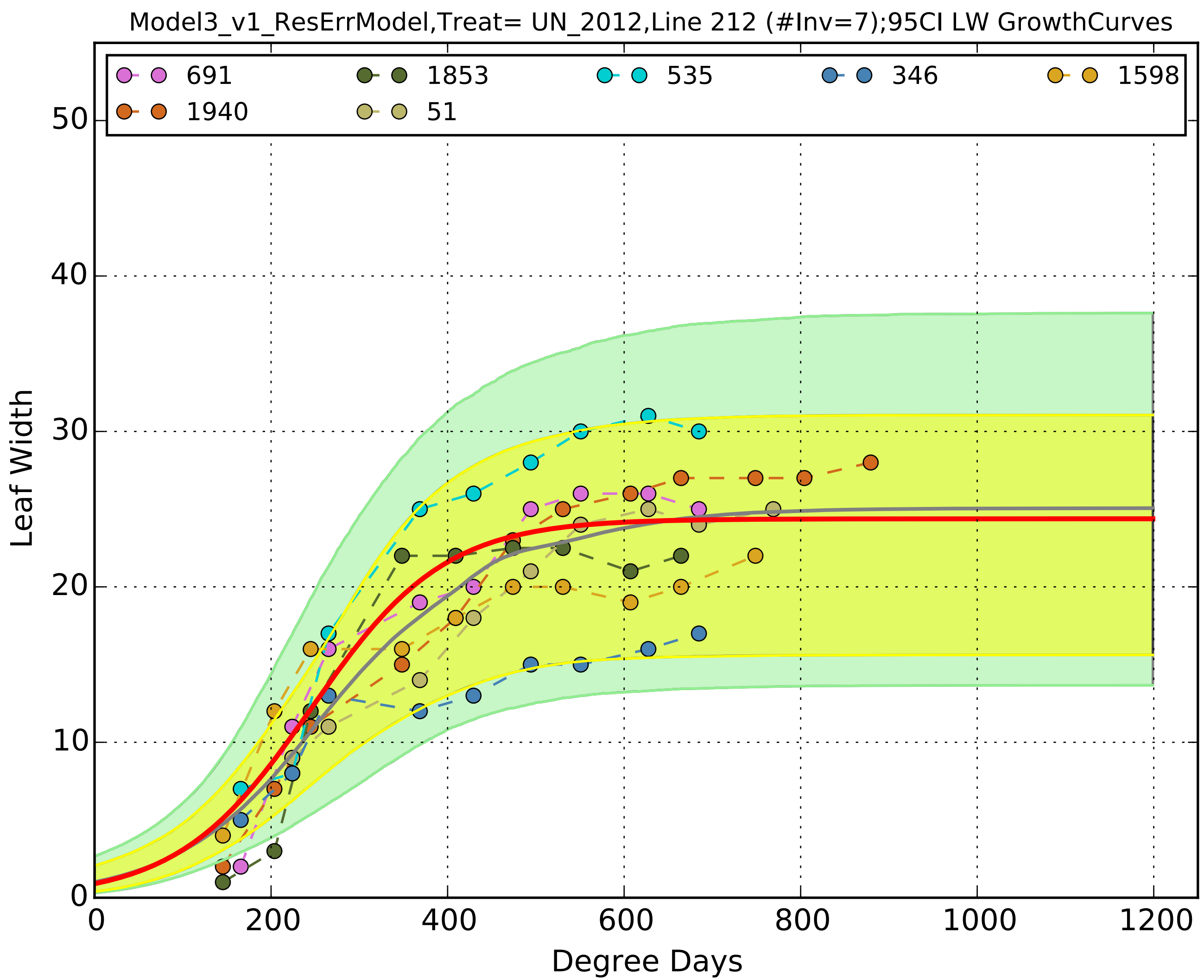

Model3_v1_ResErrModel,Treat= UN_2012,Line 212 (#Inv=7);95CI LW GrowthCurves
691
1853
535
346
1598
1940
51
Leaf Width
Degree Days
0
10
20
30
40
50
0
200
400
600
800
1000
1200

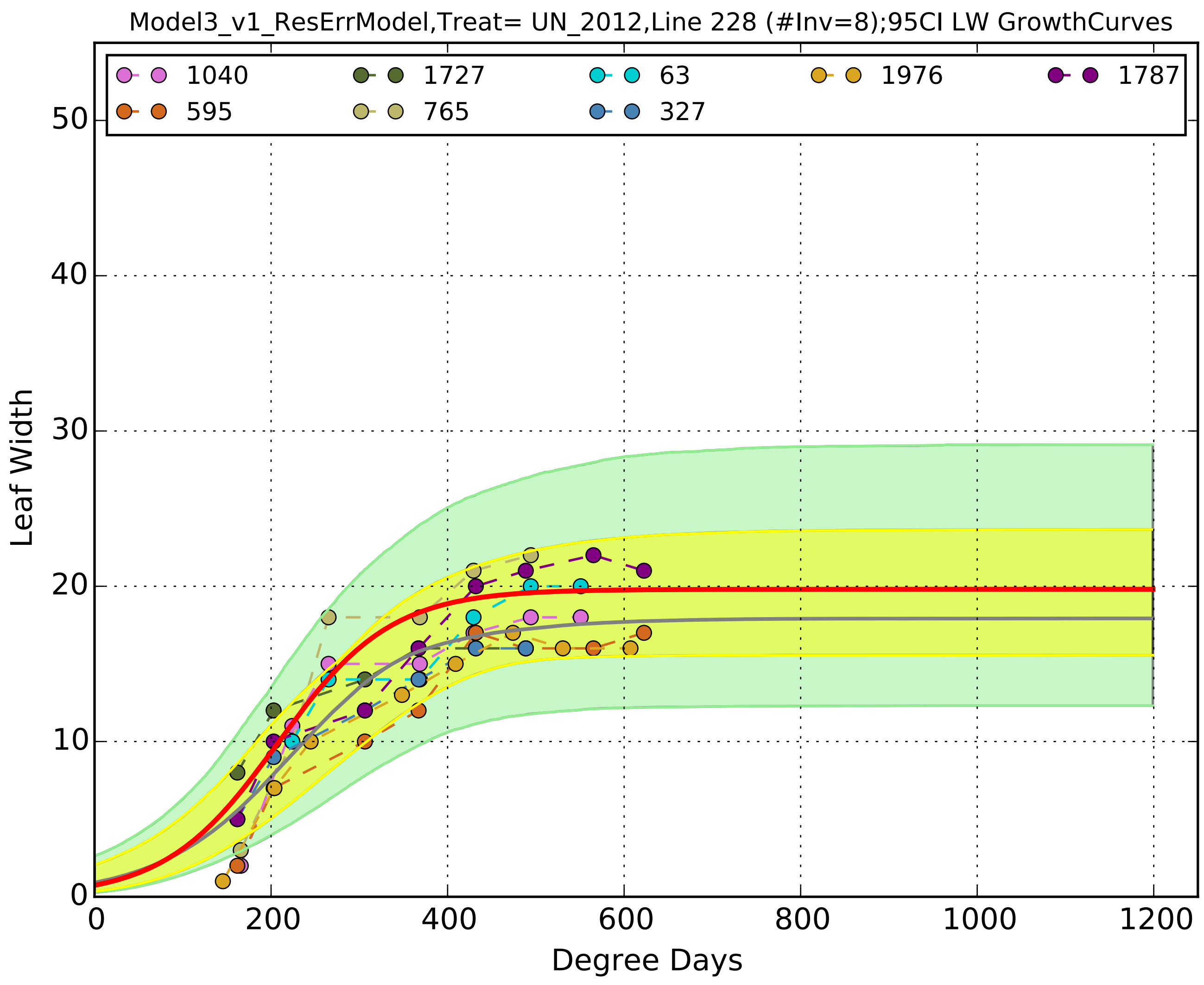
Model3_v1_ResErrModel,Treat= UN_2012,Line 228 (#Inv=8);95CI LW GrowthCurves
1040
595
1727
765
63
327
1976
1787
Leaf Width
Degree Days
0
10
20
30
40
50
0
200
400
600
800
1000
1200

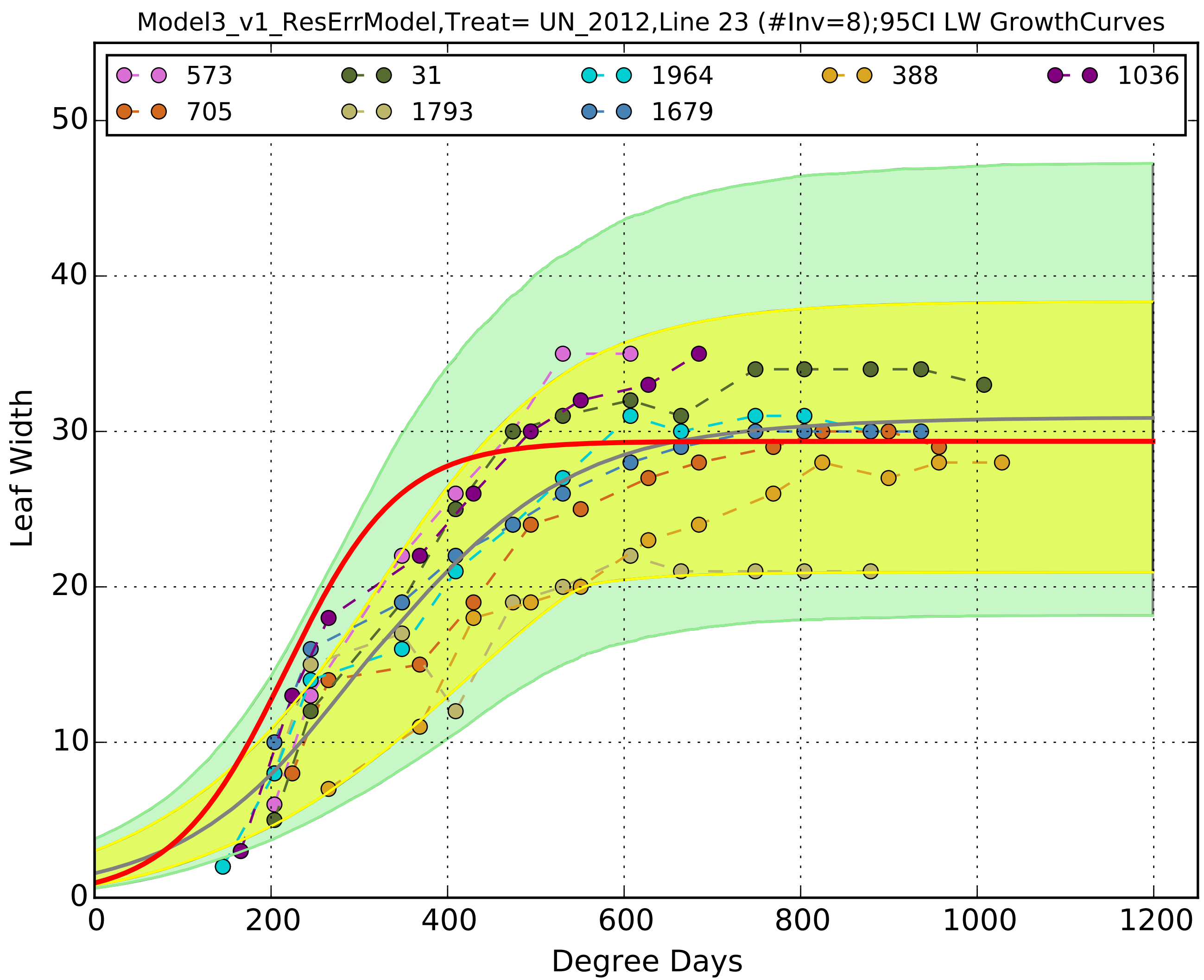
Model3_v1_ResErrModel,Treat= UN_2012,Line 23 (#Inv=8);95CI LW GrowthCurves
573
705
31
1793
1964
1679
388
1036
Leaf Width
Degree Days
0
10
20
30
40
50
0
200
400
600
800
1000
1200

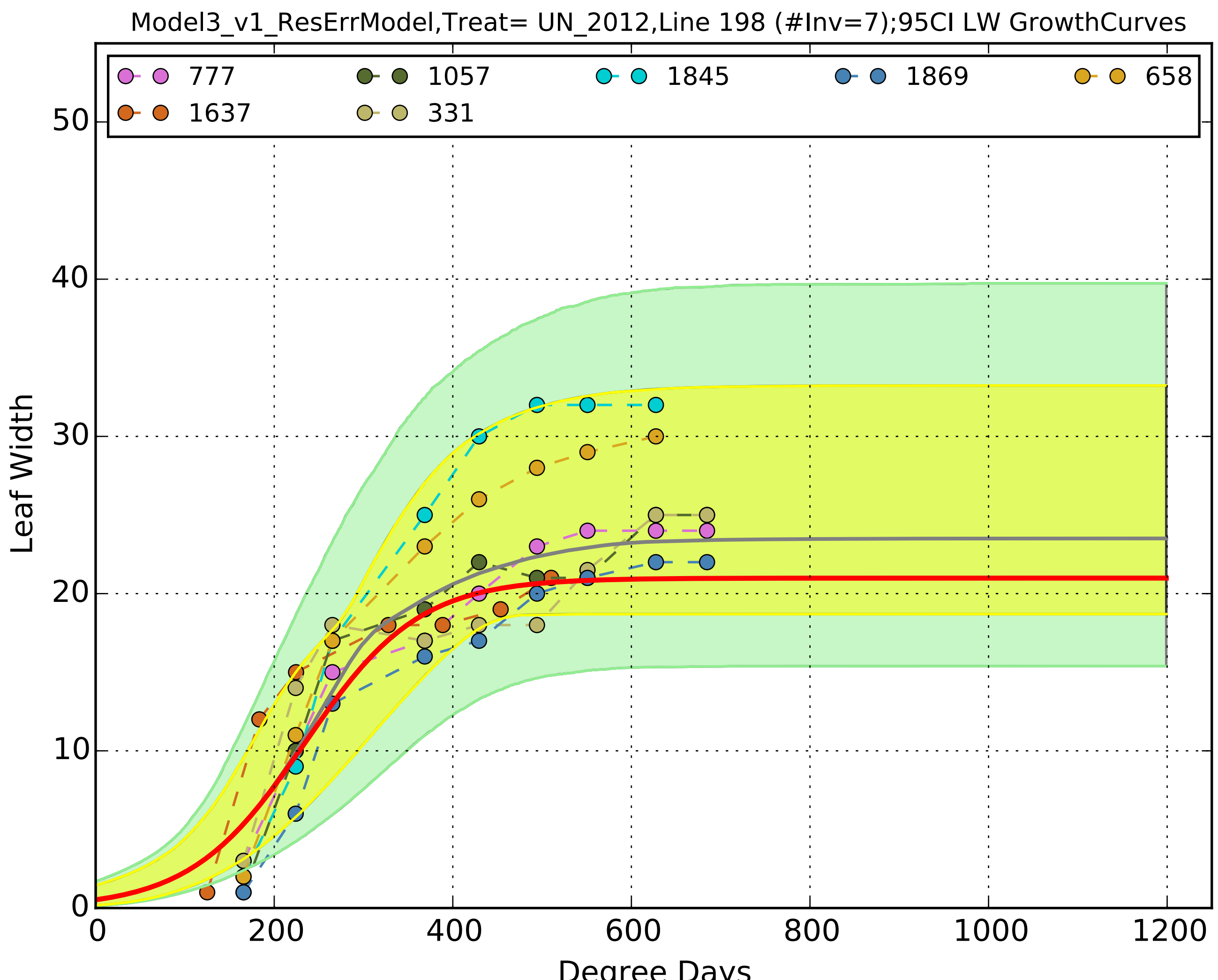

Model3_v1_ResErrModel,Treat= UN_2012,Line 198 (#Inv=7);95CI LW GrowthCurves
777
1057
1845
1869
658
1637
331
Leaf Width
Degree Days
0
10
20
30
40
50
0
200
400
600
800
1000
1200

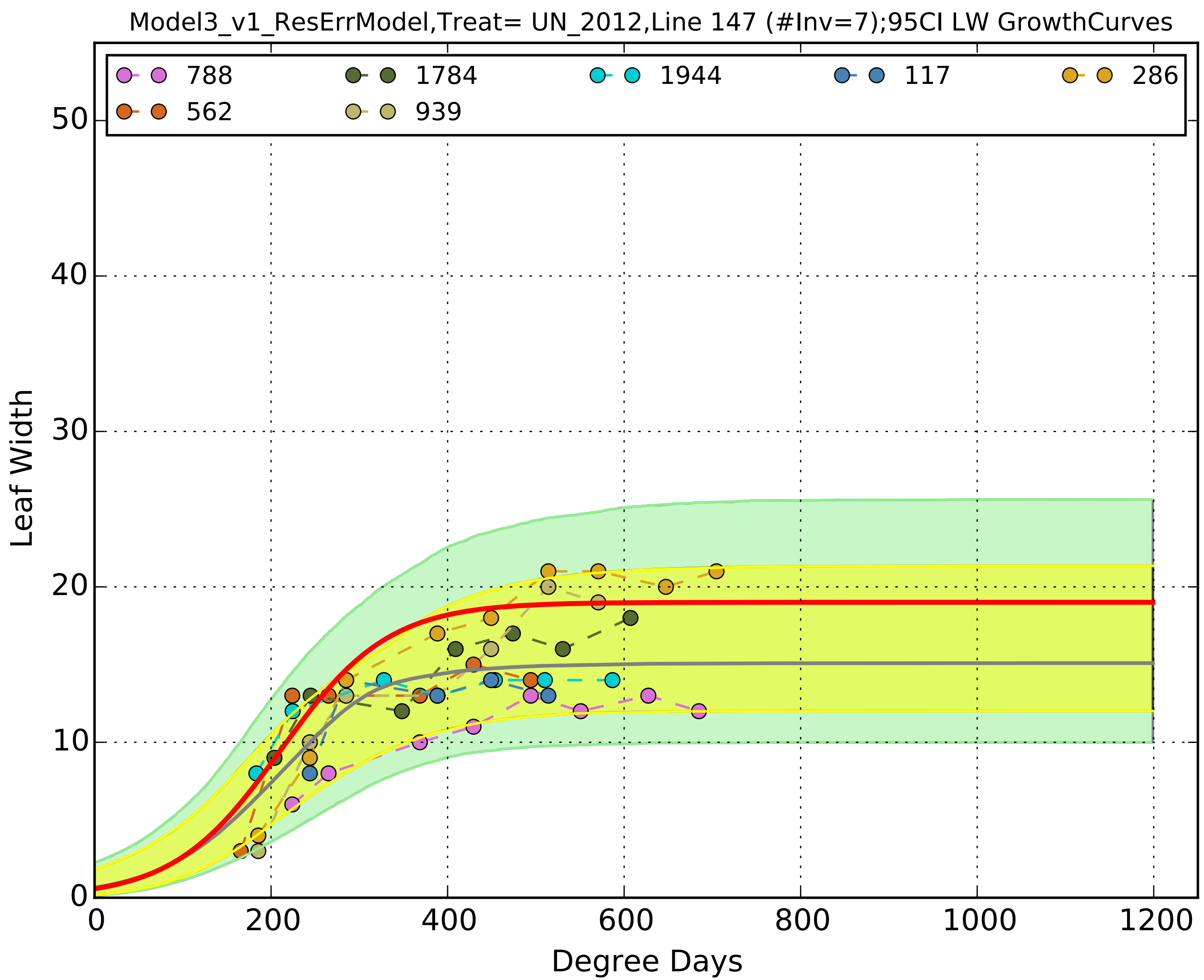

Model3_v1_ResErrModel,Treat= UN_2012,Line 147 (#Inv=7);95CI LW GrowthCurves
788
1784
1944
117
286
562
939
Leaf Width
Degree Days
0
10
20
30
40
50
0
200
400
600
800
1000
1200

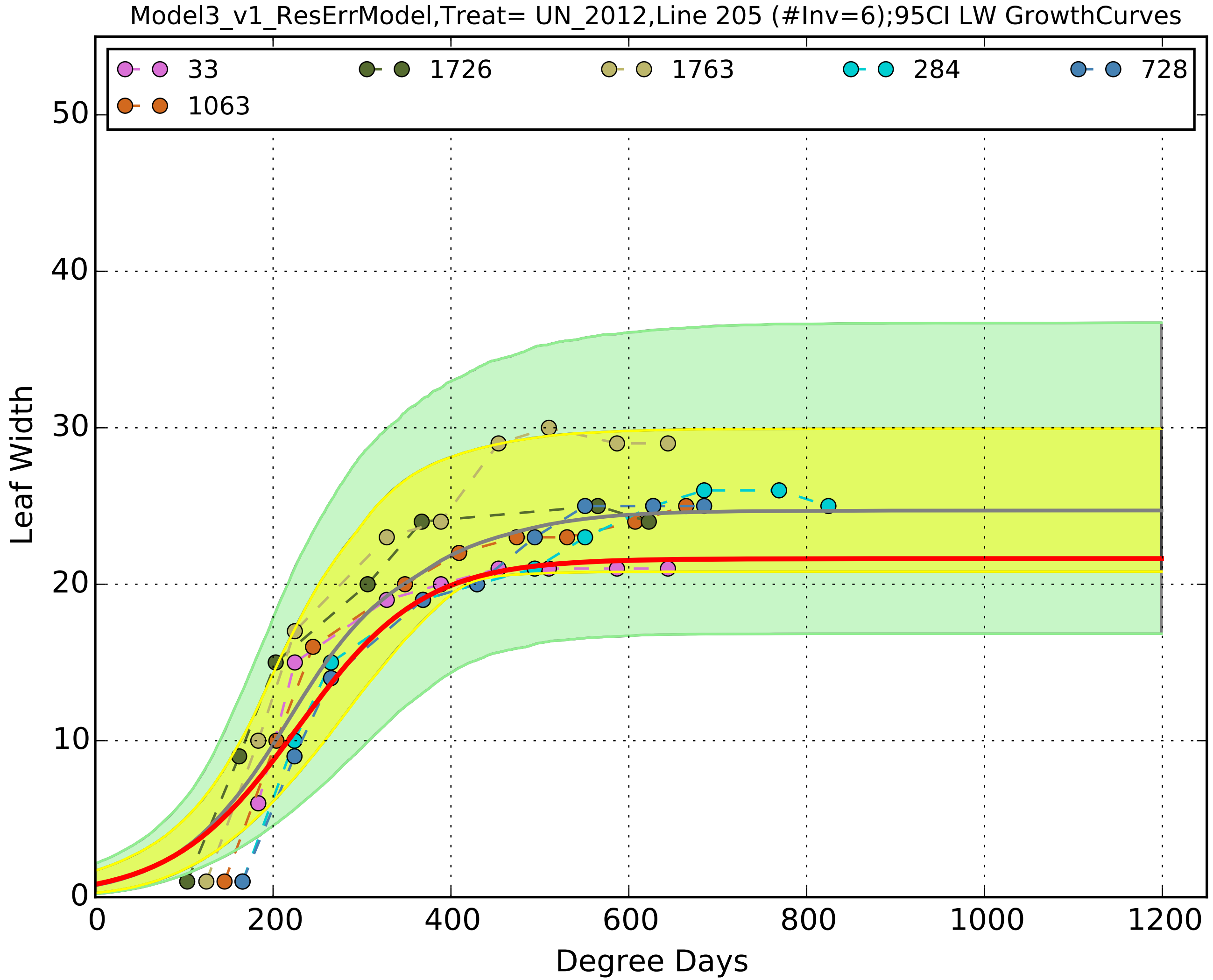

Model3_v1_ResErrModel,Treat= UN_2012,Line 205 (#Inv=6);95CI LW GrowthCurves
33
1726
1763
284
728
1063
Leaf Width
Degree Days
0
10
20
30
40
50
0
200
400
600
800
1000
1200

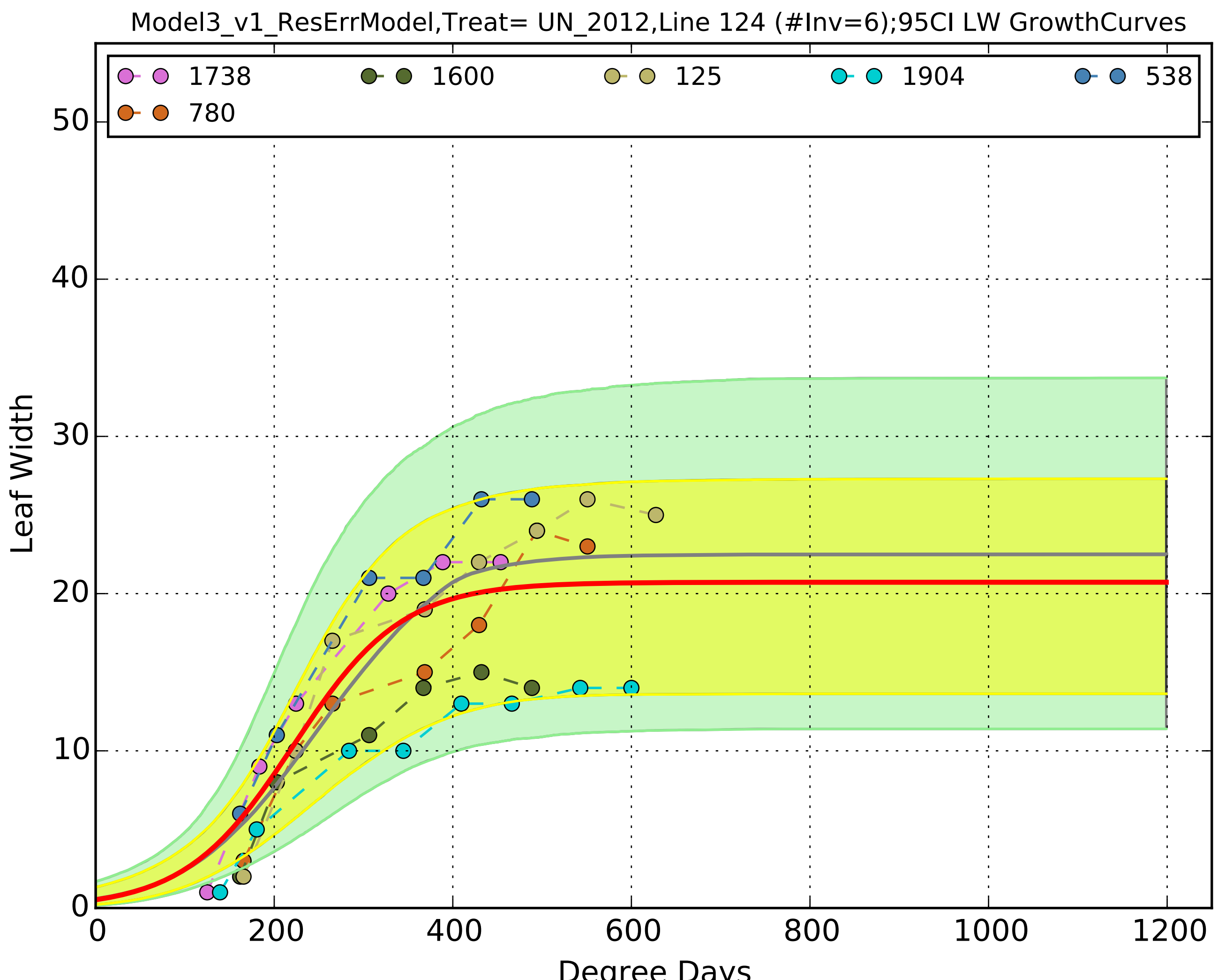

Model3_v1_ResErrModel,Treat= UN_2012,Line 124 (#Inv=6);95CI LW GrowthCurves
1738
1600
125
1904
538
780
Leaf Width
Degree Days
0
10
20
30
40
50
0
200
400
600
800
1000
1200

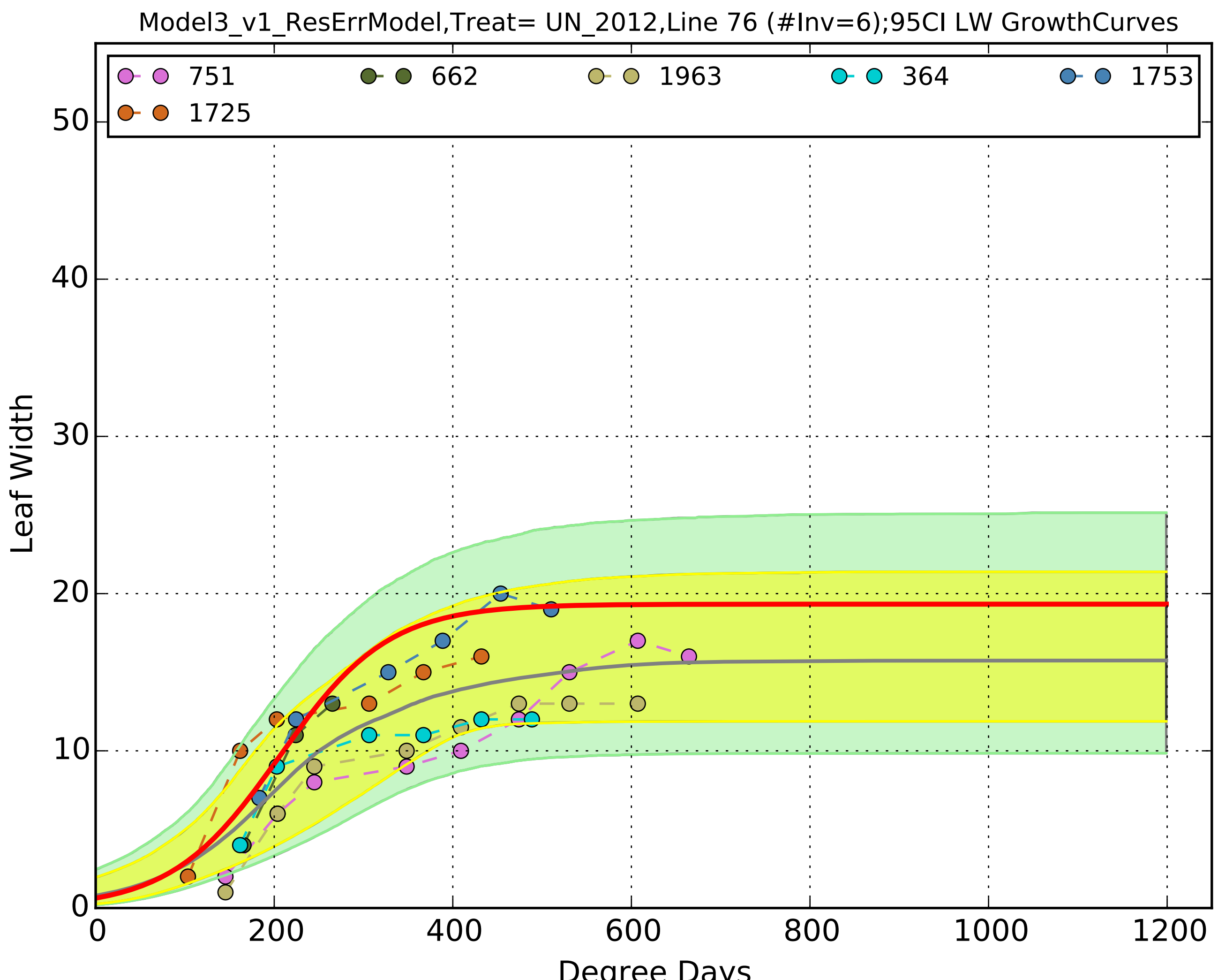

Model3_v1_ResErrModel,Treat= UN_2012,Line 76 (#Inv=6);95CI LW GrowthCurves
751
662
1963
364
1753
1725
Leaf Width
0
10
20
30
40
50
0
200
400
600
800
1000
1200
Degree Days

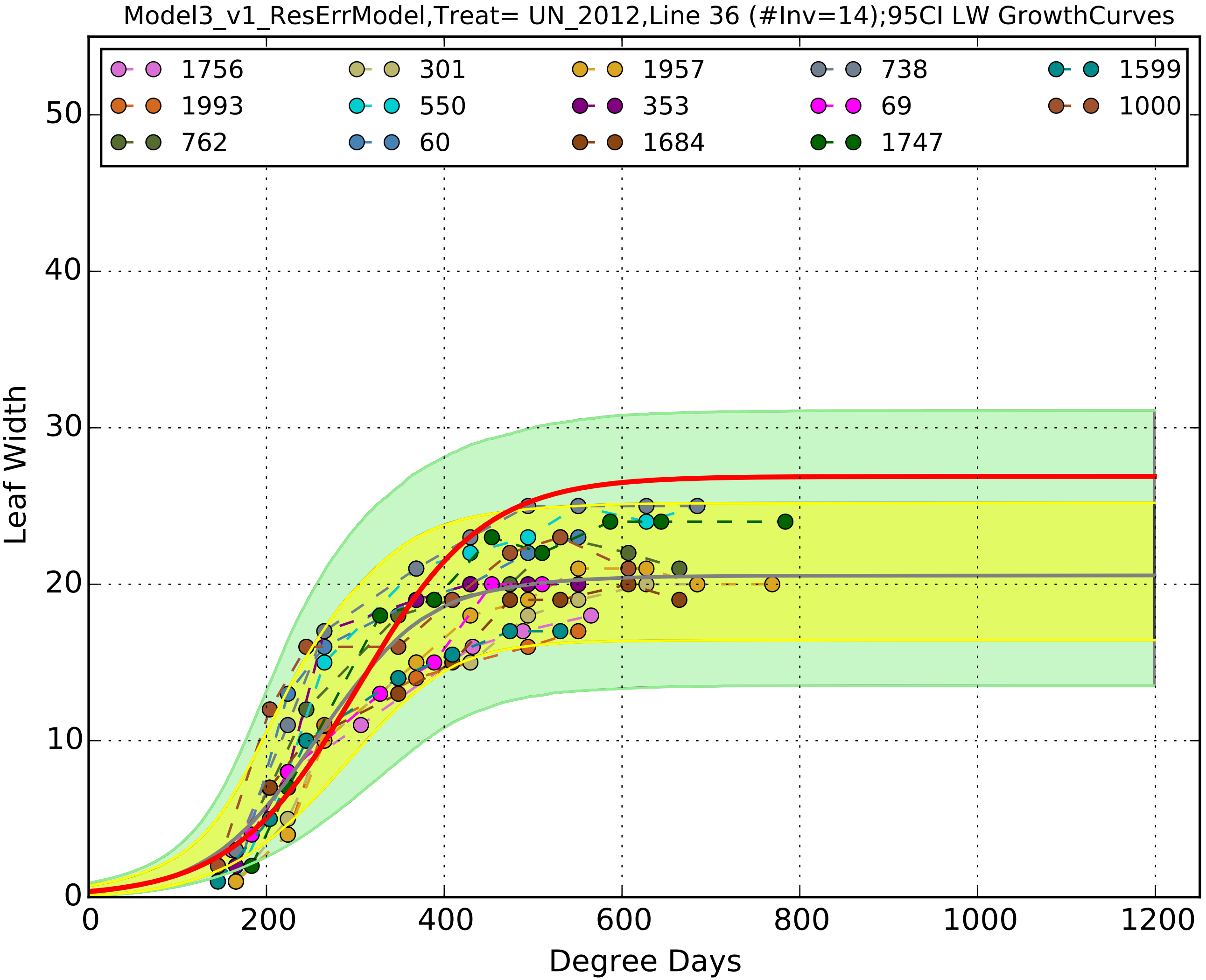

Model3_v1_ResErrModel,Treat= UN_2012,Line 36 (#Inv=14);95CI LW GrowthCurves
1756
301
1957
738
1599
1993
550
353
69
1000
762
60
1684
1747
Leaf Width
Degree Days
0
10
20
30
40
50
0
200
400
600
800
1000
1200

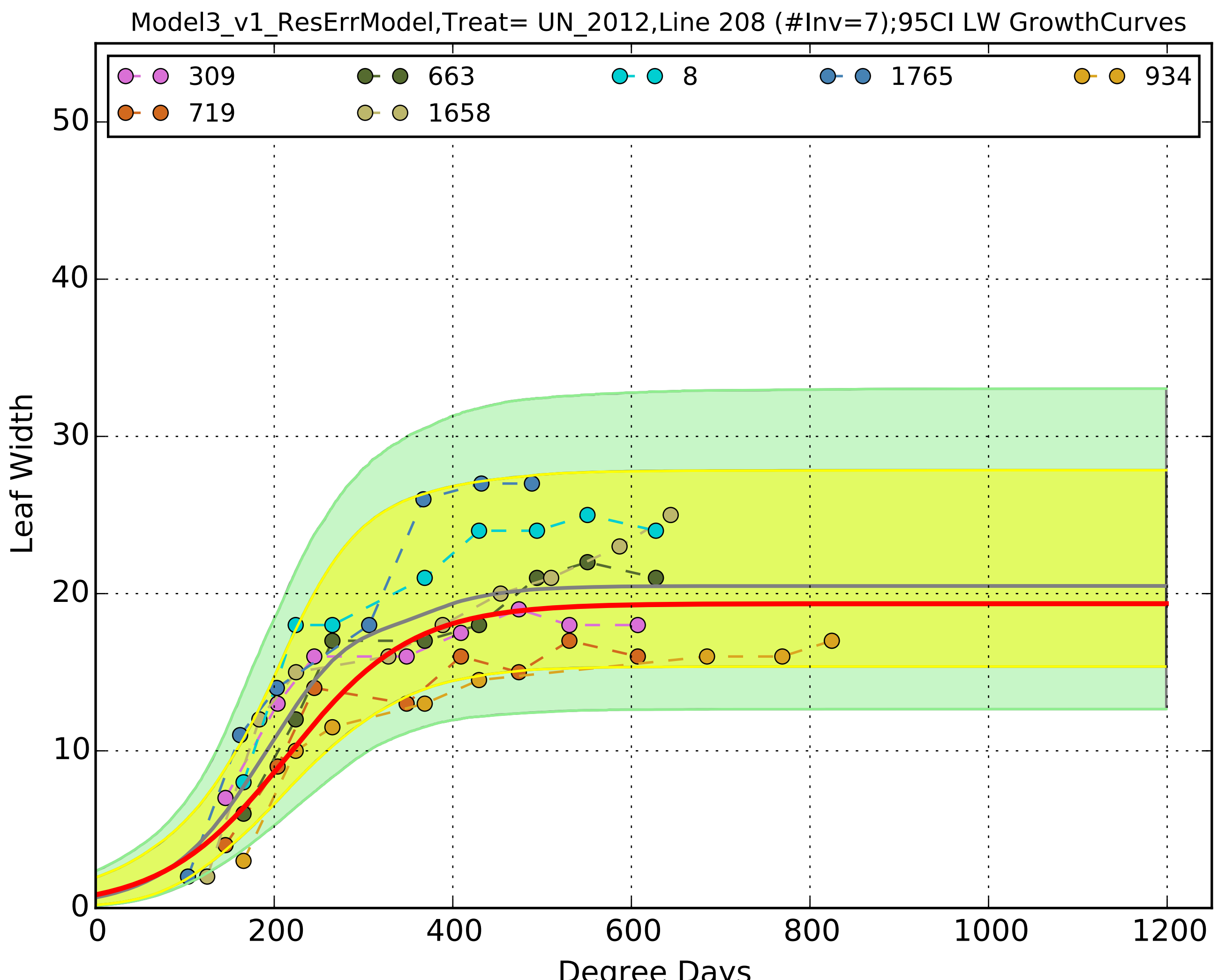

Model3_v1_ResErrModel,Treat= UN_2012,Line 208 (#Inv=7);95CI LW GrowthCurves
309
663
8
1765
934
719
1658
Leaf Width
Degree Days
0
10
20
30
40
50
0
200
400
600
800
1000
1200

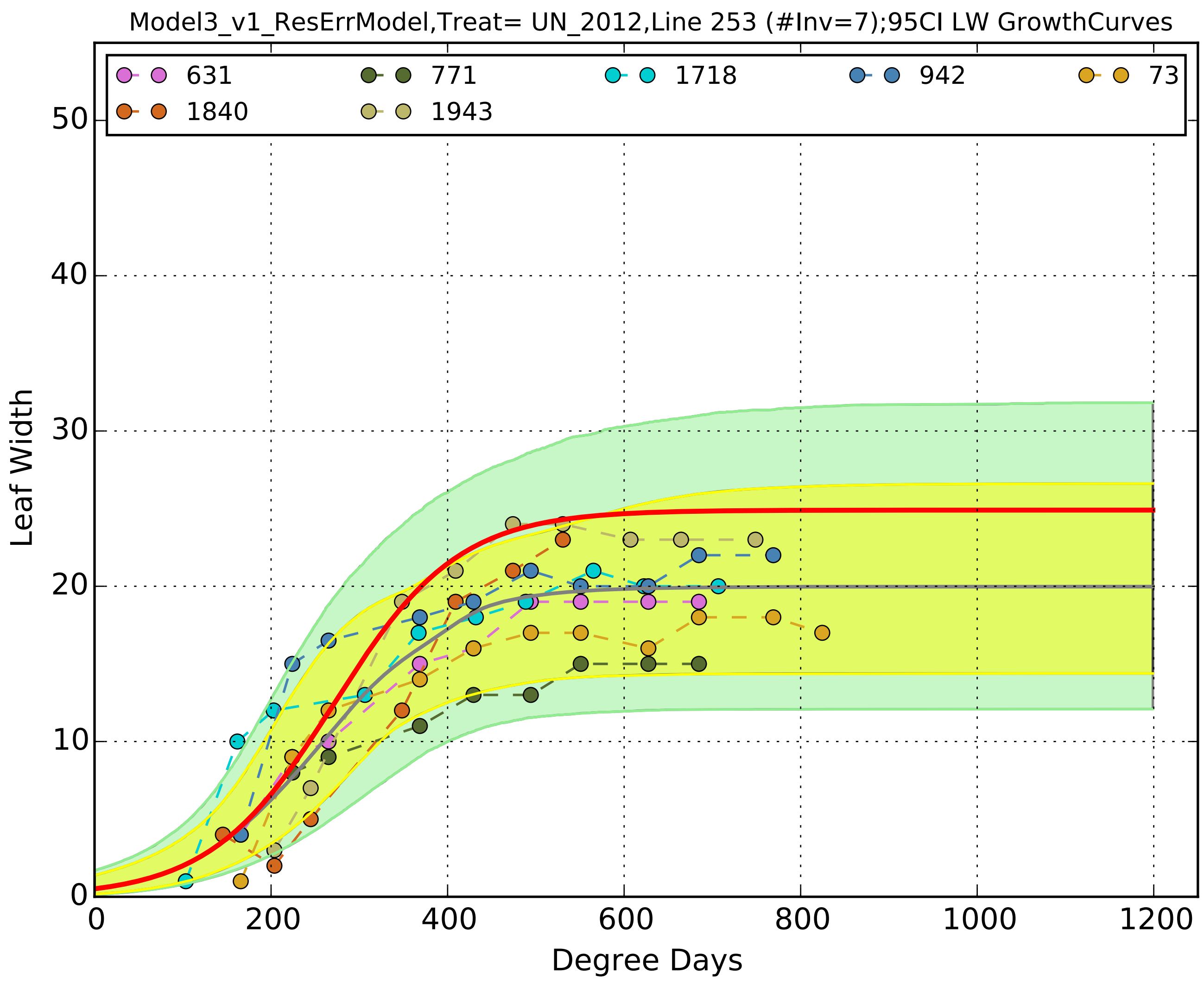

Model3_v1_ResErrModel,Treat= UN_2012,Line 253 (#Inv=7);95CI LW GrowthCurves
631
771
1718
942
73
1840
1943
Leaf Width
Degree Days
0
10
20
30
40
50
0
200
400
600
800
1000
1200

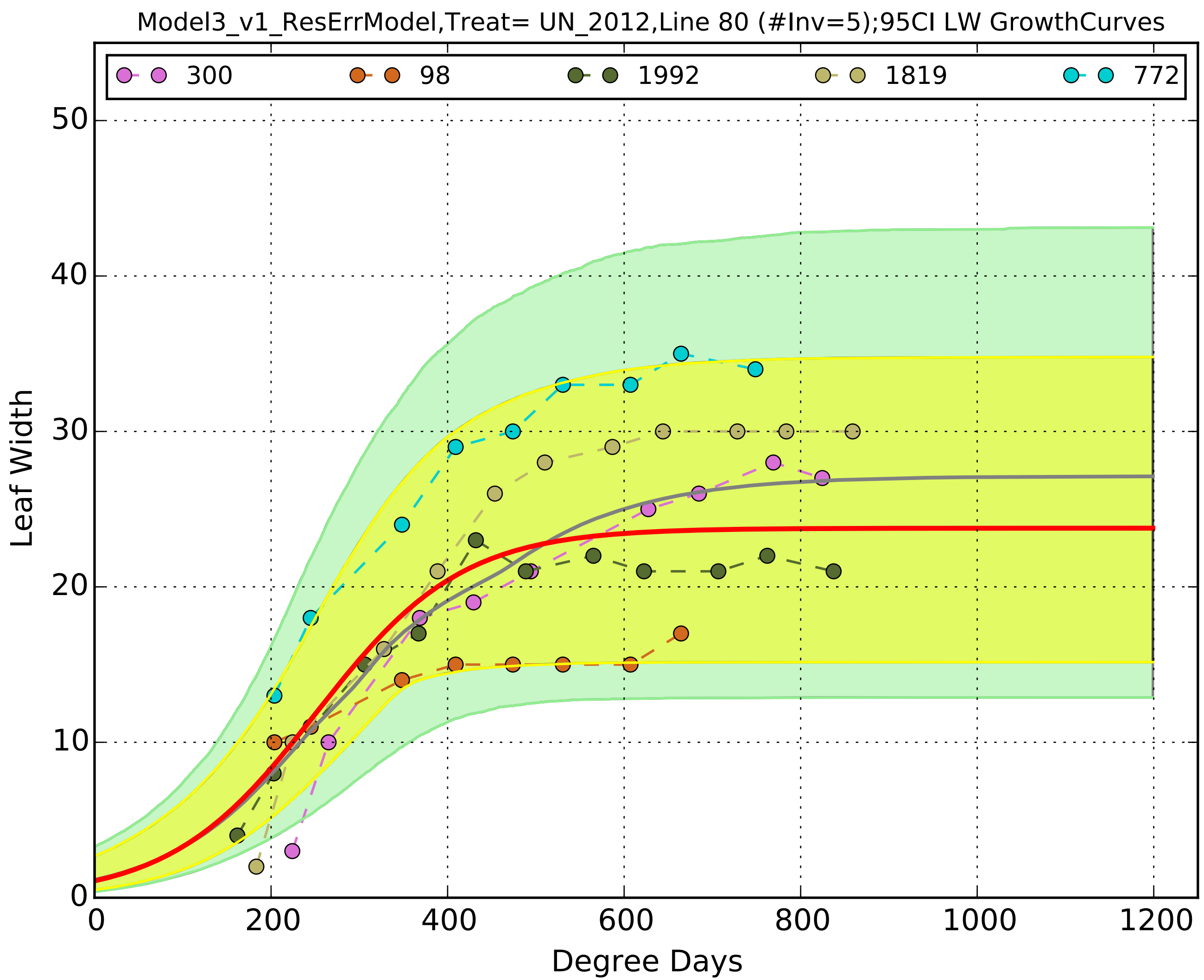

Model3_v1_ResErrModel,Treat= UN_2012,Line 80 (#Inv=5);95CI LW GrowthCurves
300
98
1992
1819
772
Leaf Width
Degree Days
0
10
20
30
40
50
0
200
400
600
800
1000
1200

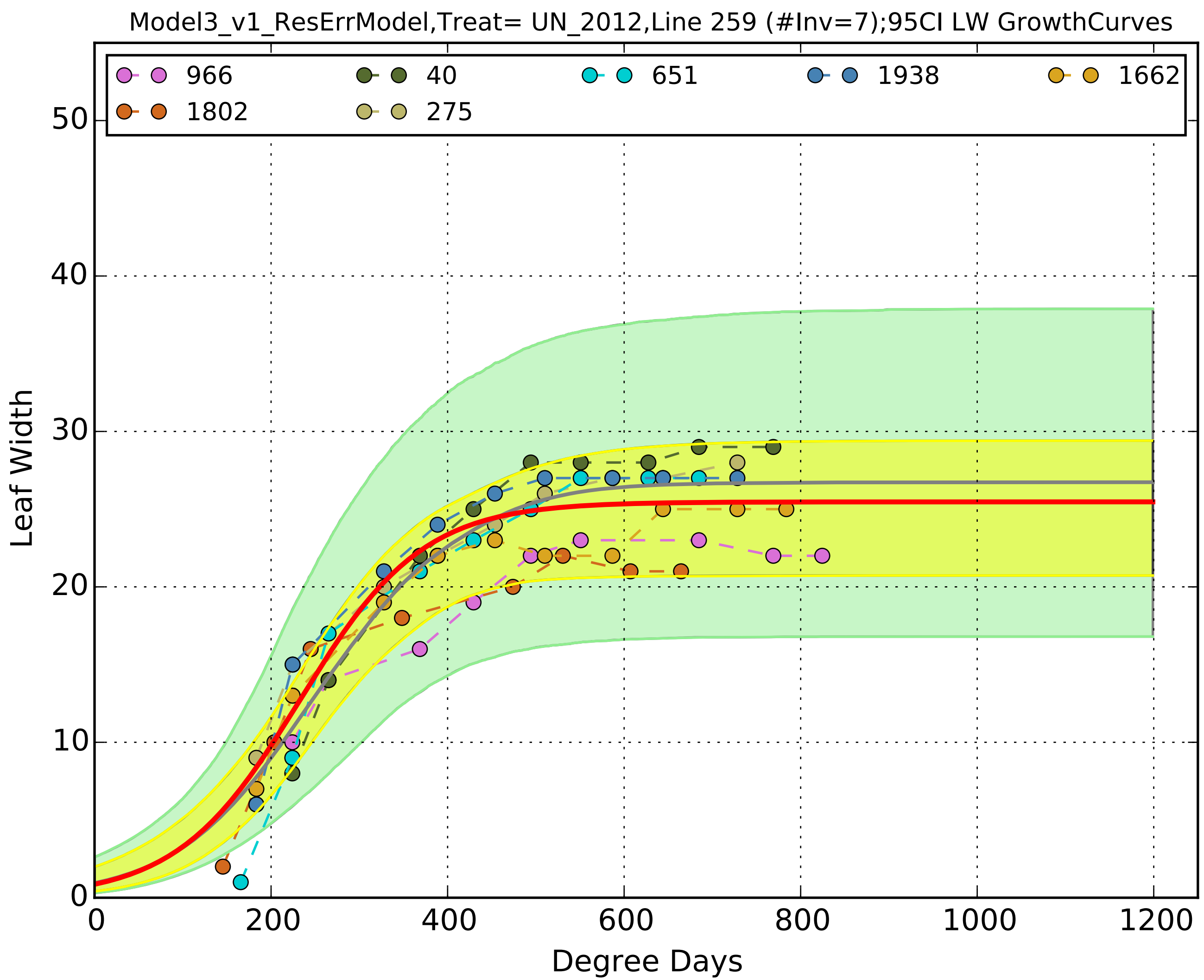

Model3_v1_ResErrModel,Treat= UN_2012,Line 259 (#Inv=7);95CI LW GrowthCurves
966
40
651
1938
1662
1802
275
Leaf Width
0
10
20
30
40
50
0
200
400
600
800
1000
1200
Degree Days

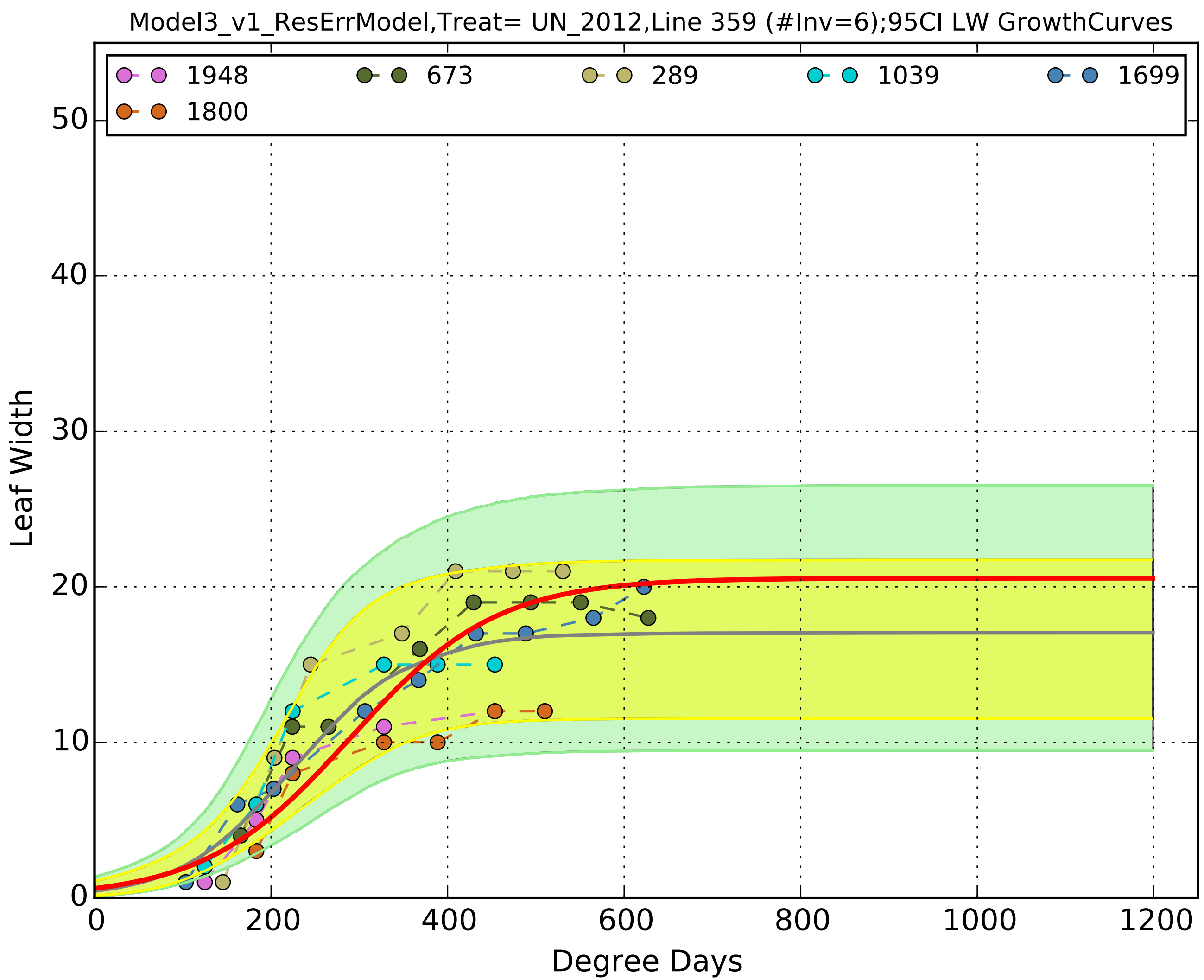

Model3_v1_ResErrModel,Treat= UN_2012,Line 359 (#Inv=6);95CI LW GrowthCurves
1948
673
289
1039
1699
1800
Leaf Width
Degree Days
0
10
20
30
40
50
0
200
400
600
800
1000
1200

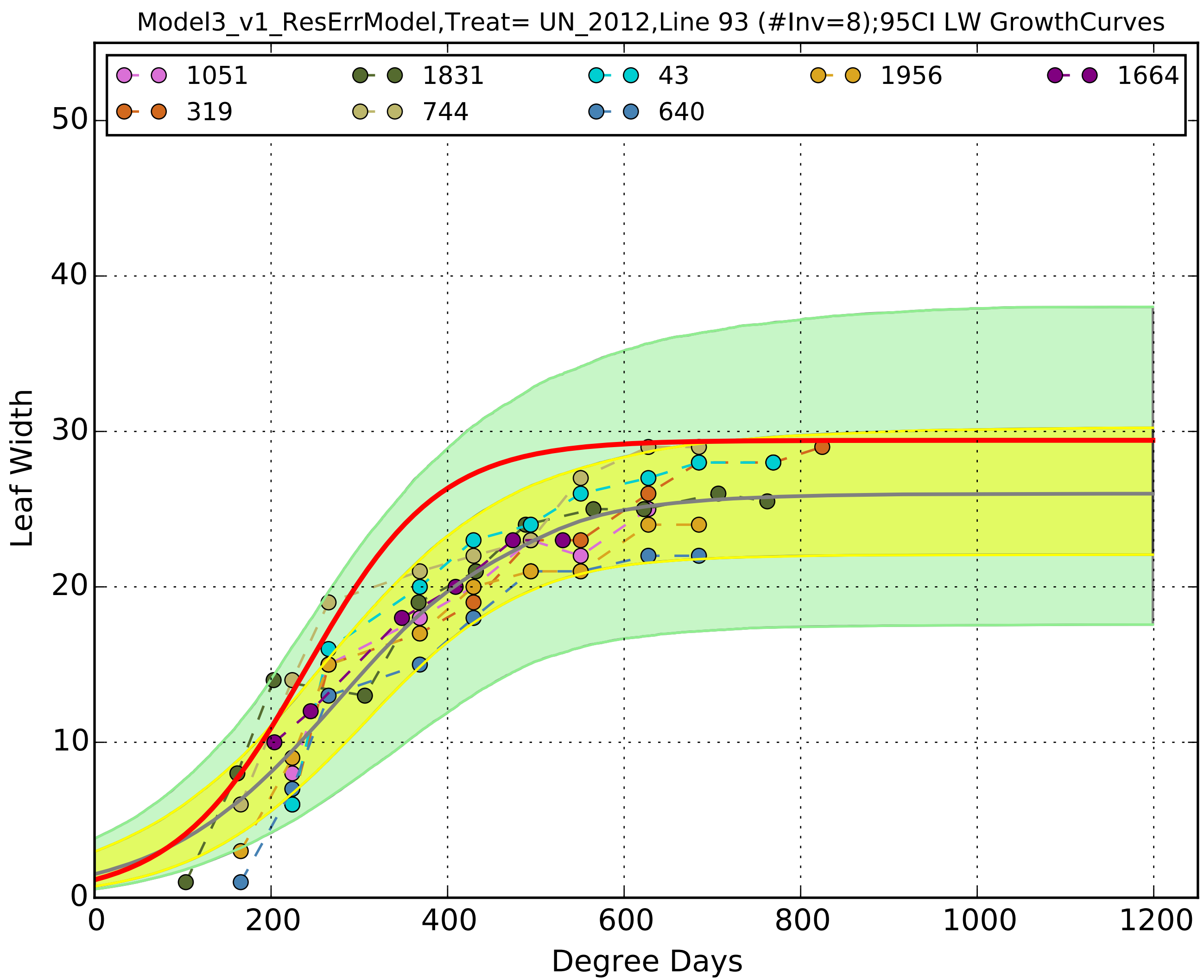
Model3_v1_ResErrModel,Treat= UN_2012,Line 93 (#Inv=8);95CI LW GrowthCurves
1051
1831
43
1956
1664
319
744
640
Leaf Width
Degree Days
0
10
20
30
40
50
0
200
400
600
800
1000
1200

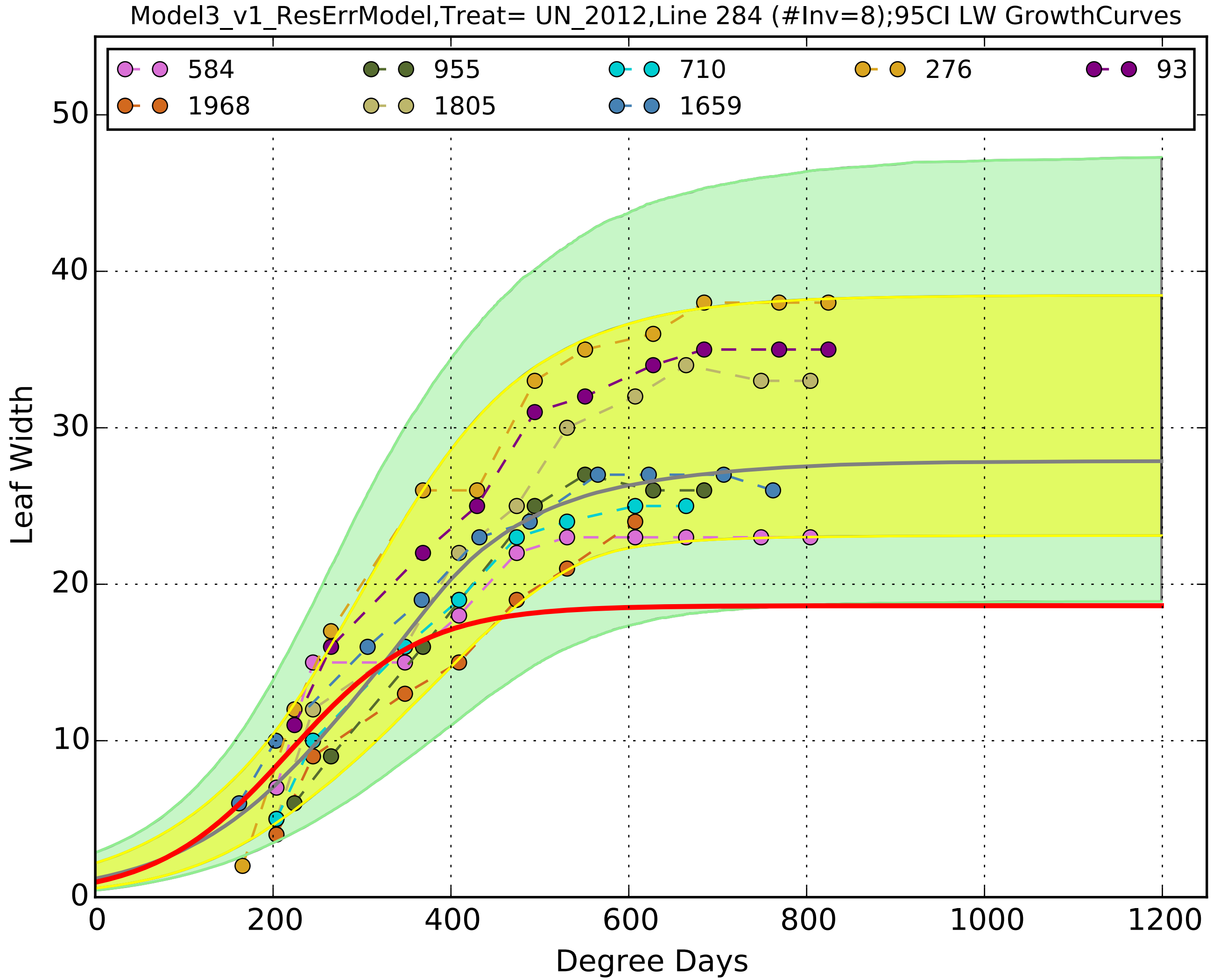
Model3_v1_ResErrModel,Treat= UN_2012,Line 284 (#Inv=8);95CI LW GrowthCurves
584
955
710
276
93
1968
1805
1659
Leaf Width
Degree Days
0
10
20
30
40
50
0
200
400
600
800
1000
1200

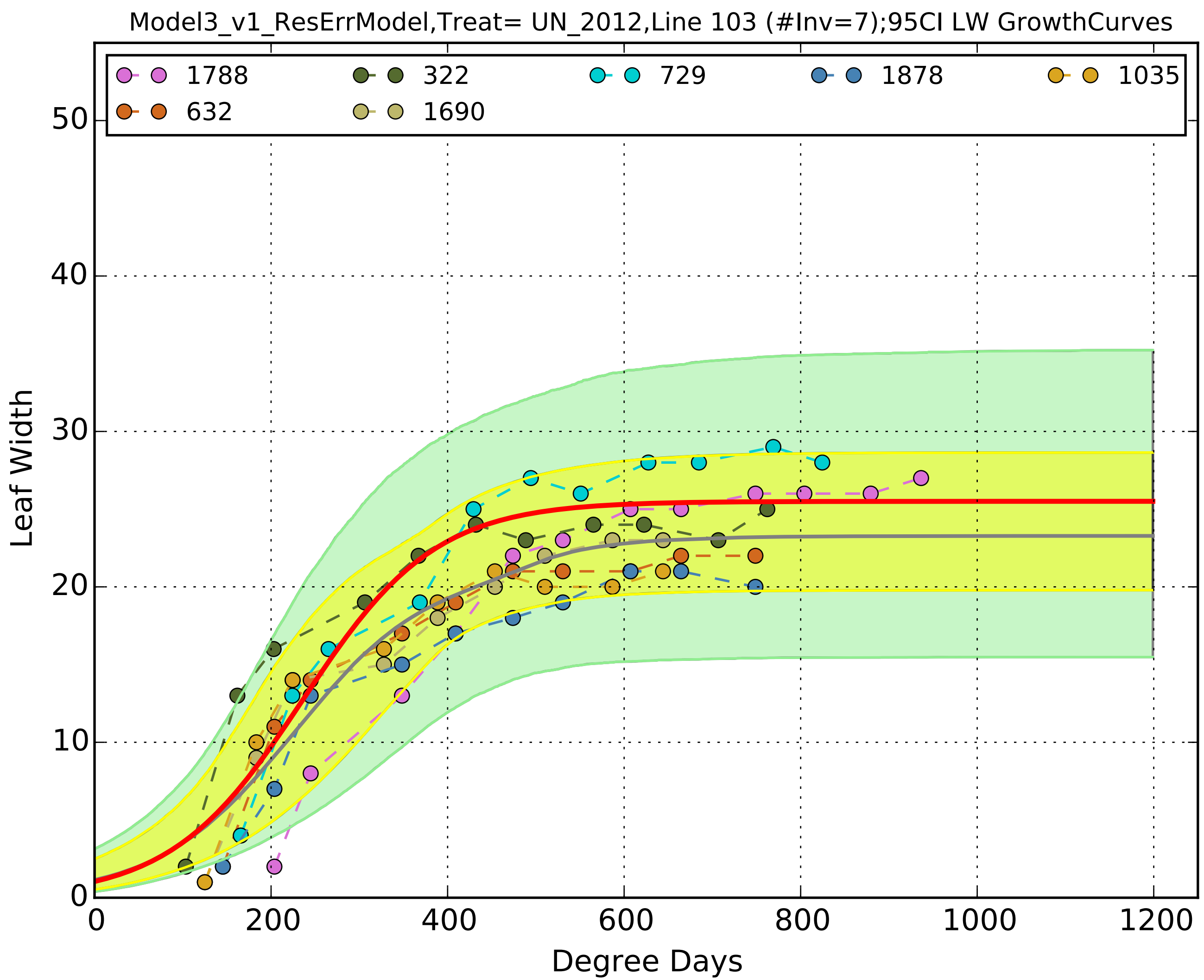

Model3_v1_ResErrModel,Treat= UN_2012,Line 103 (#Inv=7);95CI LW GrowthCurves
1788
322
729
1878
1035
632
1690
Leaf Width
Degree Days
0
10
20
30
40
50
0
200
400
600
800
1000
1200

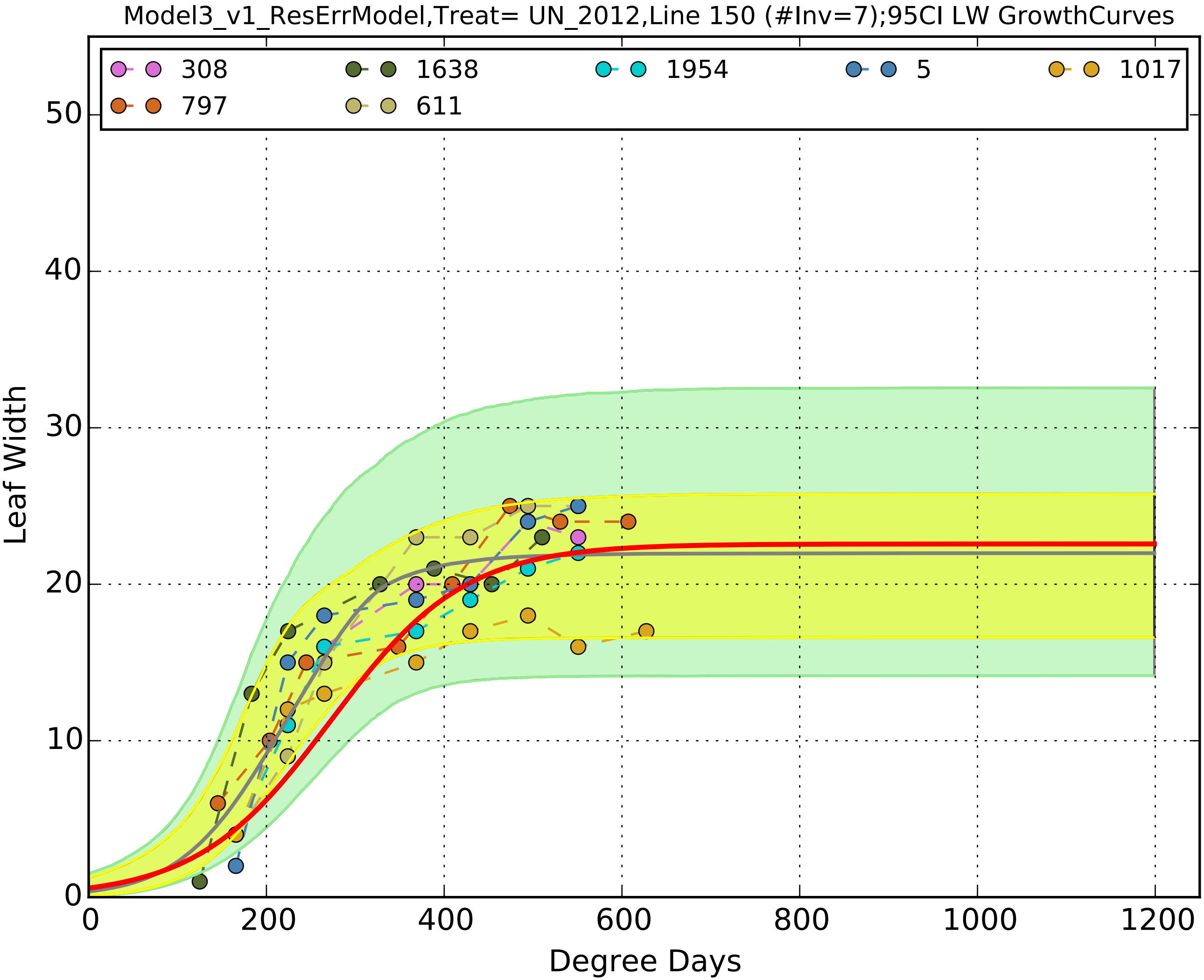
Model3_v1_ResErrModel,Treat= UN_2012,Line 150 (#Inv=7);95CI LW GrowthCurves
308
1638
1954
5
1017
797
611
Leaf Width
Degree Days
0
10
20
30
40
50
0
200
400
600
800
1000
1200

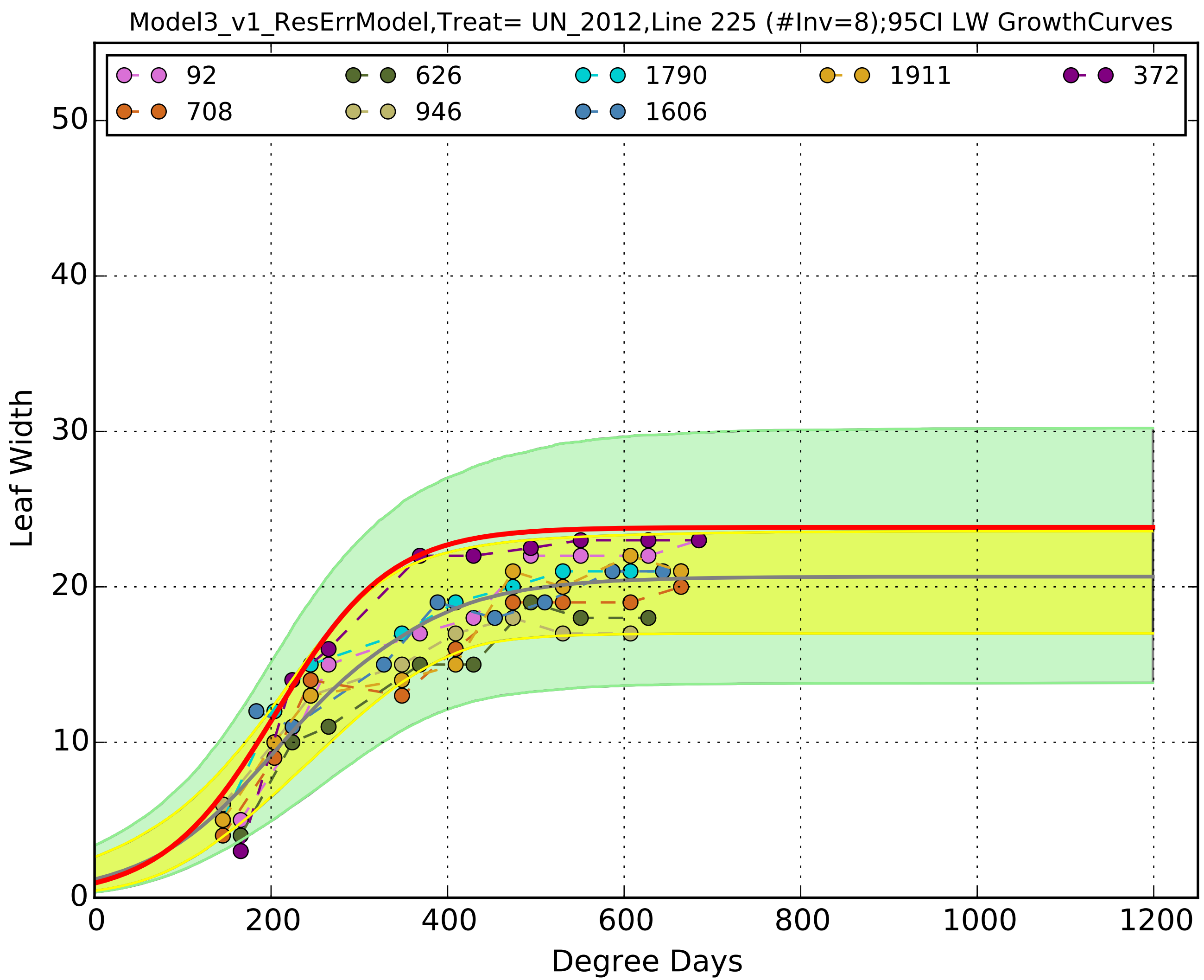

Model3_v1_ResErrModel,Treat= UN_2012,Line 225 (#Inv=8);95CI LW GrowthCurves
92
626
1790
1911
372
708
946
1606
Leaf Width
Degree Days
0
10
20
30
40
50
0
200
400
600
800
1000
1200

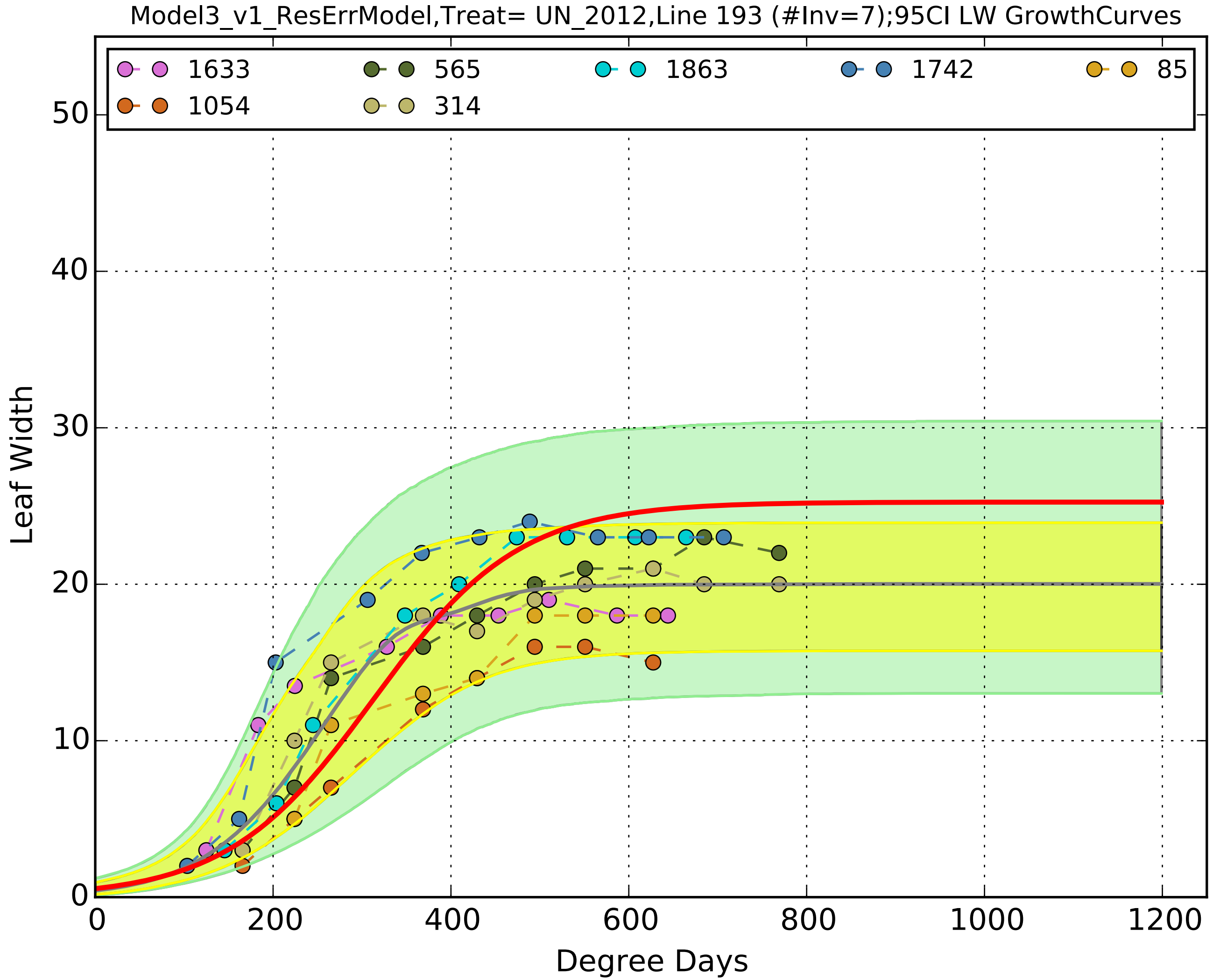

Model3_v1_ResErrModel,Treat= UN_2012,Line 193 (#Inv=7);95CI LW GrowthCurves
1633
565
1863
1742
85
1054
314
Leaf Width
Degree Days
0
10
20
30
40
50
0
200
400
600
800
1000
1200

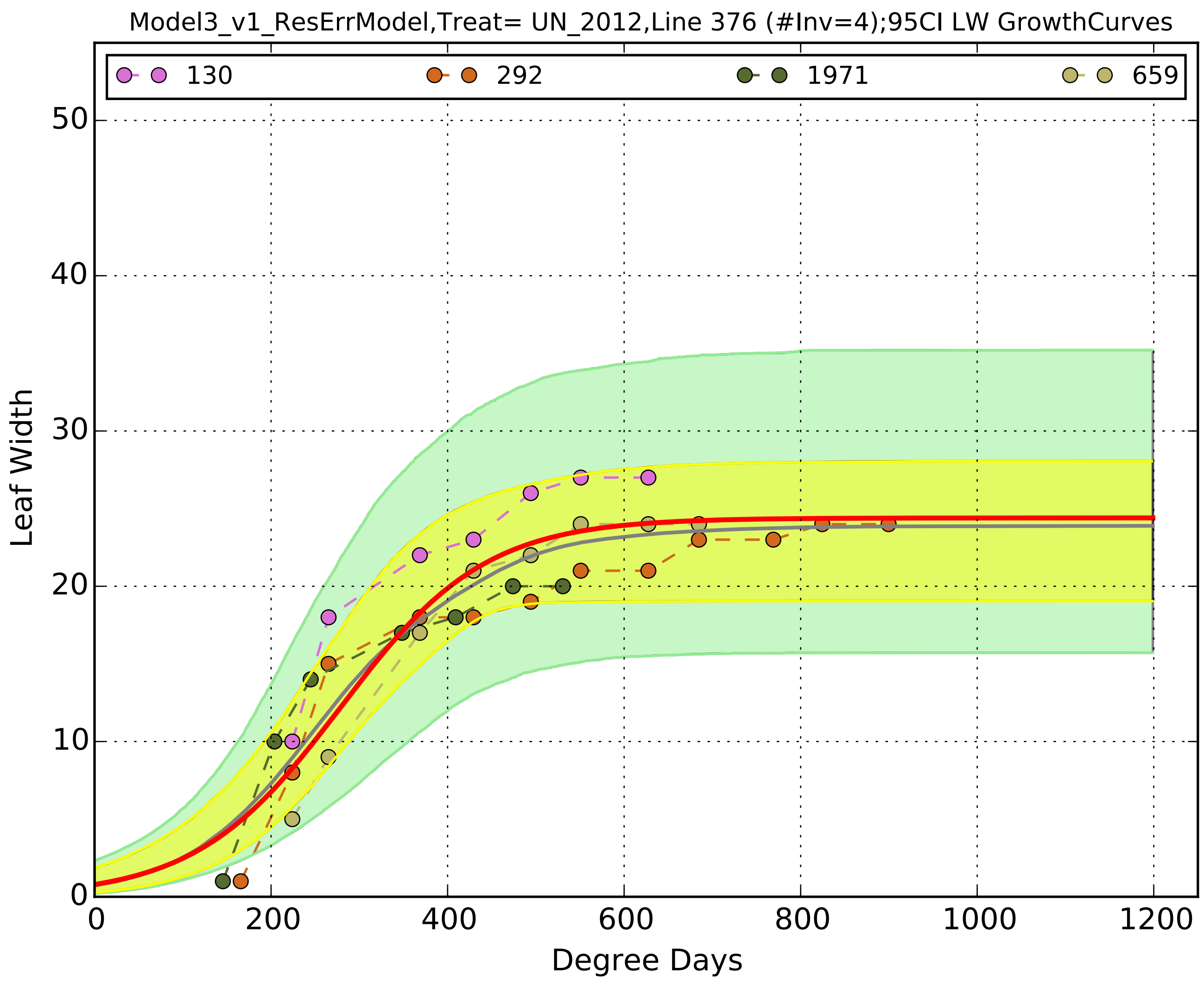
Model3_v1_ResErrModel,Treat= UN_2012,Line 376 (#Inv=4);95CI LW GrowthCurves
130
292
1971
659
Leaf Width
Degree Days
0
10
20
30
40
50
0
200
400
600
800
1000
1200

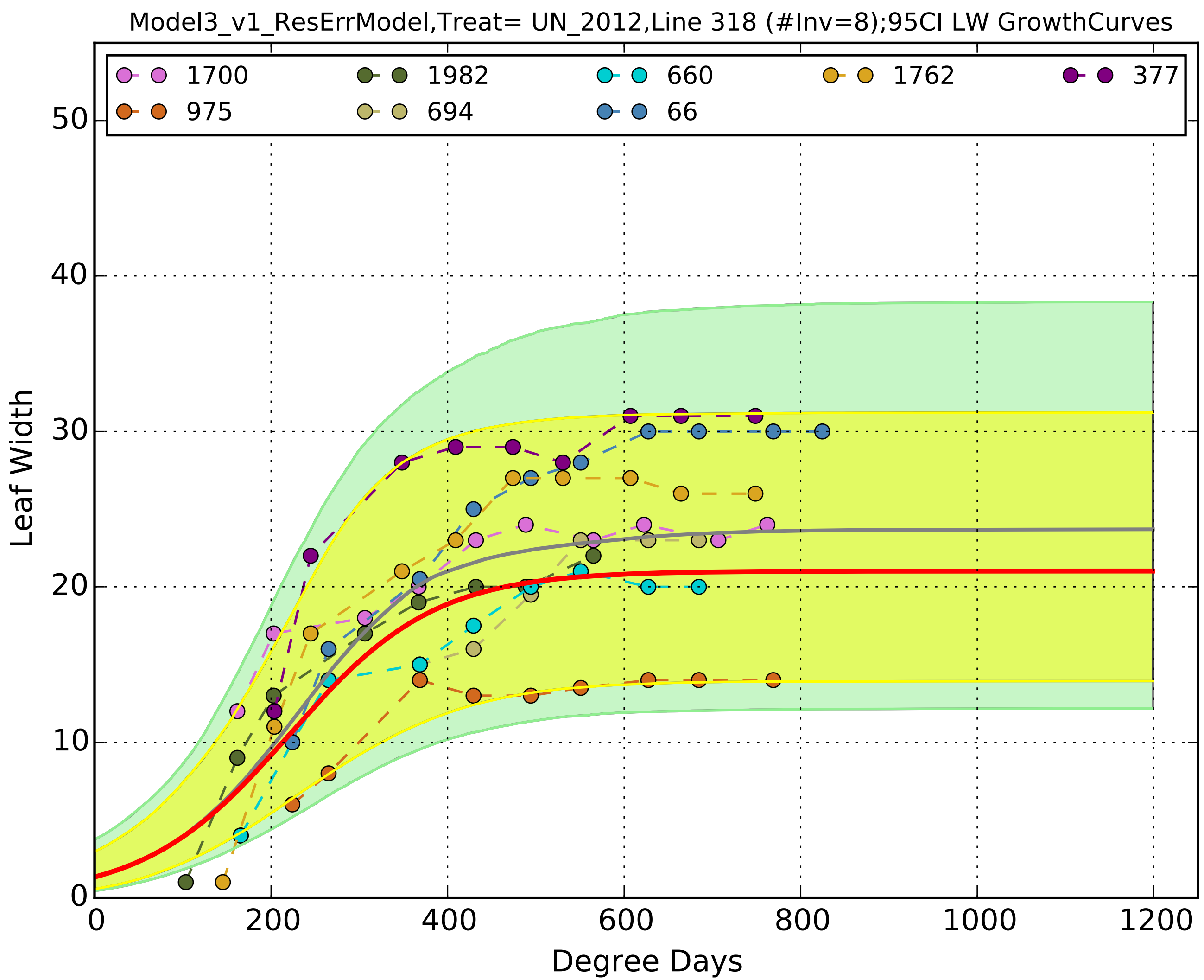

Model3_v1_ResErrModel,Treat= UN_2012,Line 318 (#Inv=8);95CI LW GrowthCurves
1700
1982
660
1762
377
975
694
66
Leaf Width
0
10
20
30
40
50
Degree Days
0
200
400
600
800
1000
1200

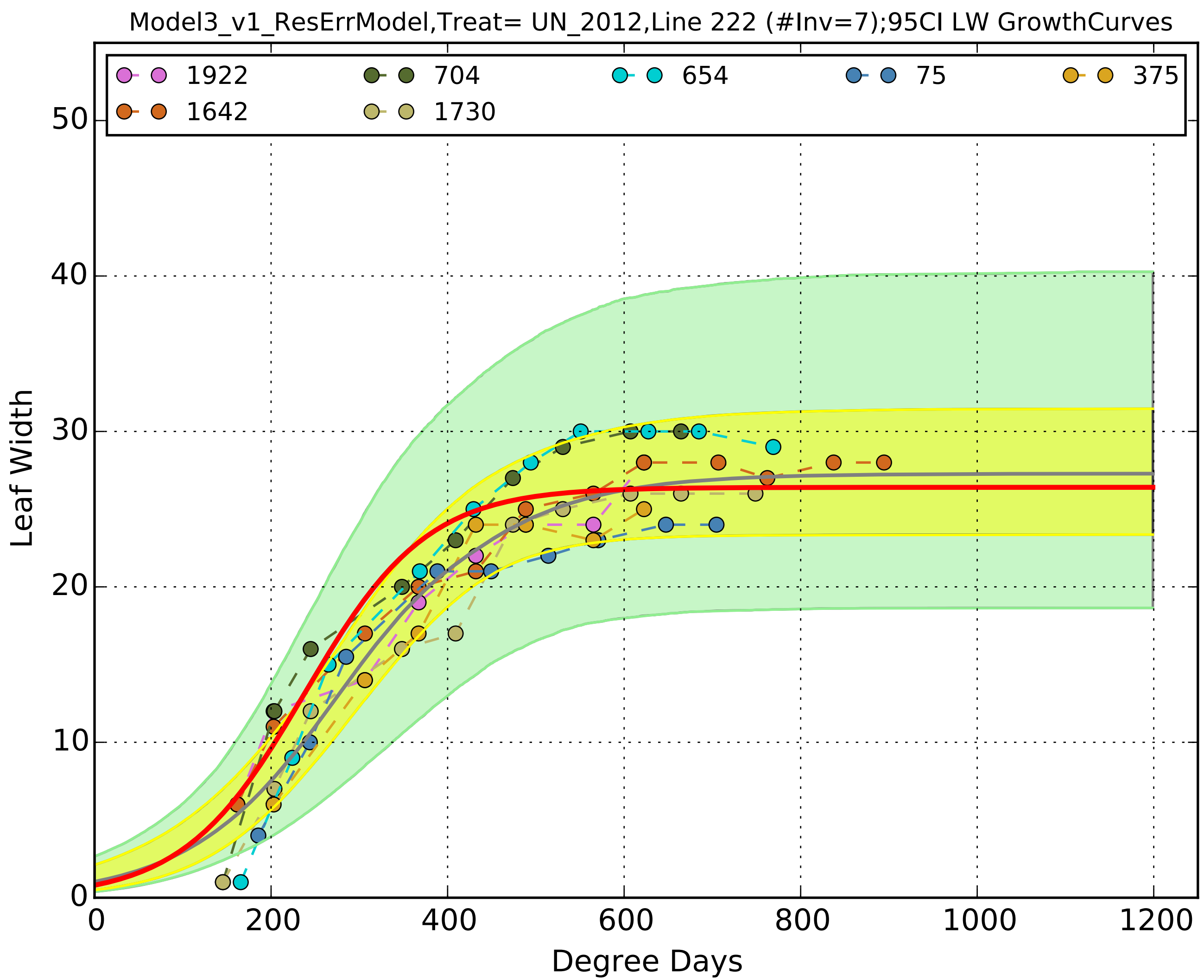
Model3_v1_ResErrModel,Treat= UN_2012,Line 222 (#Inv=7);95CI LW GrowthCurves
1922
704
654
75
375
1642
1730
Leaf Width
Degree Days
0
10
20
30
40
50
0
200
400
600
800
1000
1200

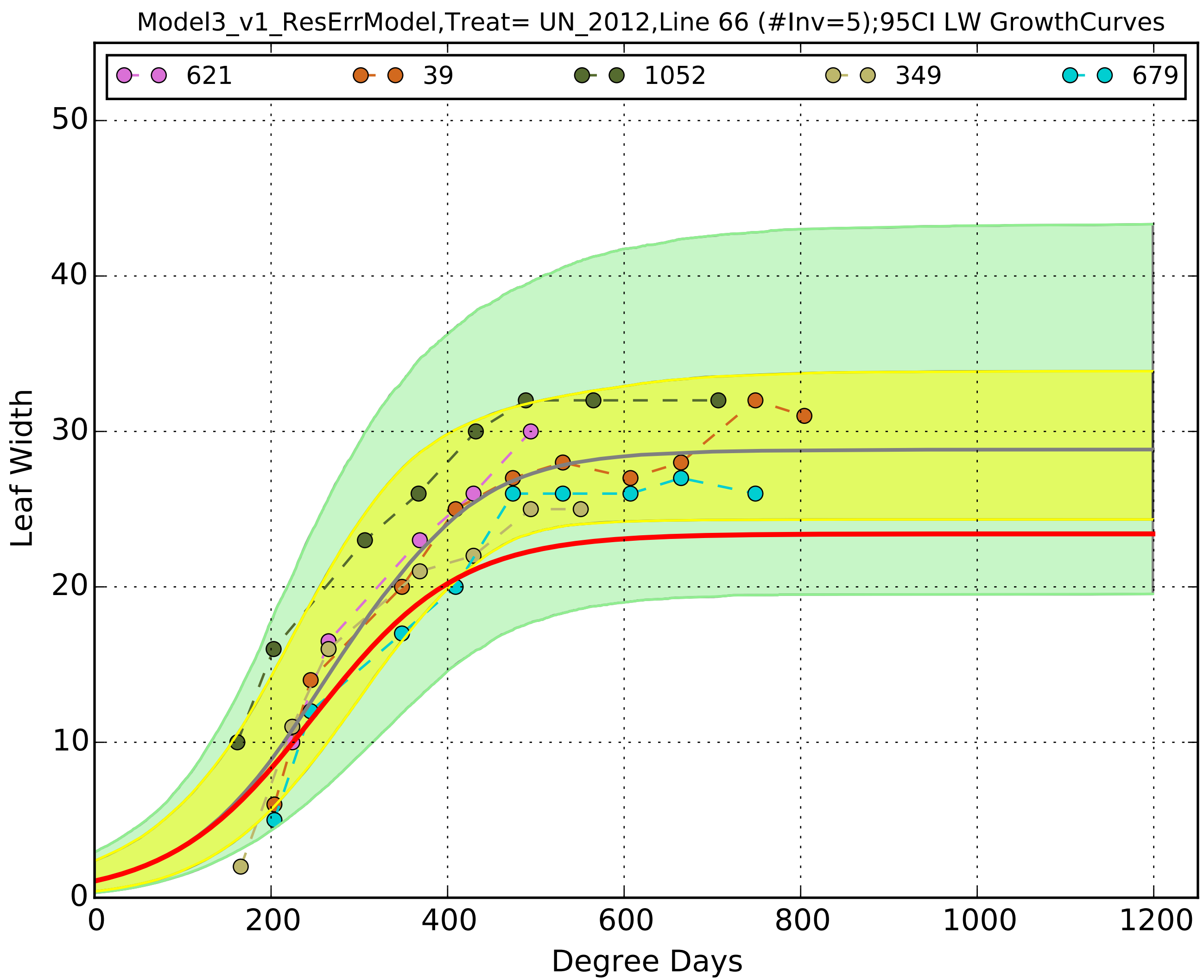

Model3_v1_ResErrModel,Treat= UN_2012,Line 66 (#Inv=5);95CI LW GrowthCurves
621
39
1052
349
679
Leaf Width
Degree Days
0
10
20
30
40
50
0
200
400
600
800
1000
1200

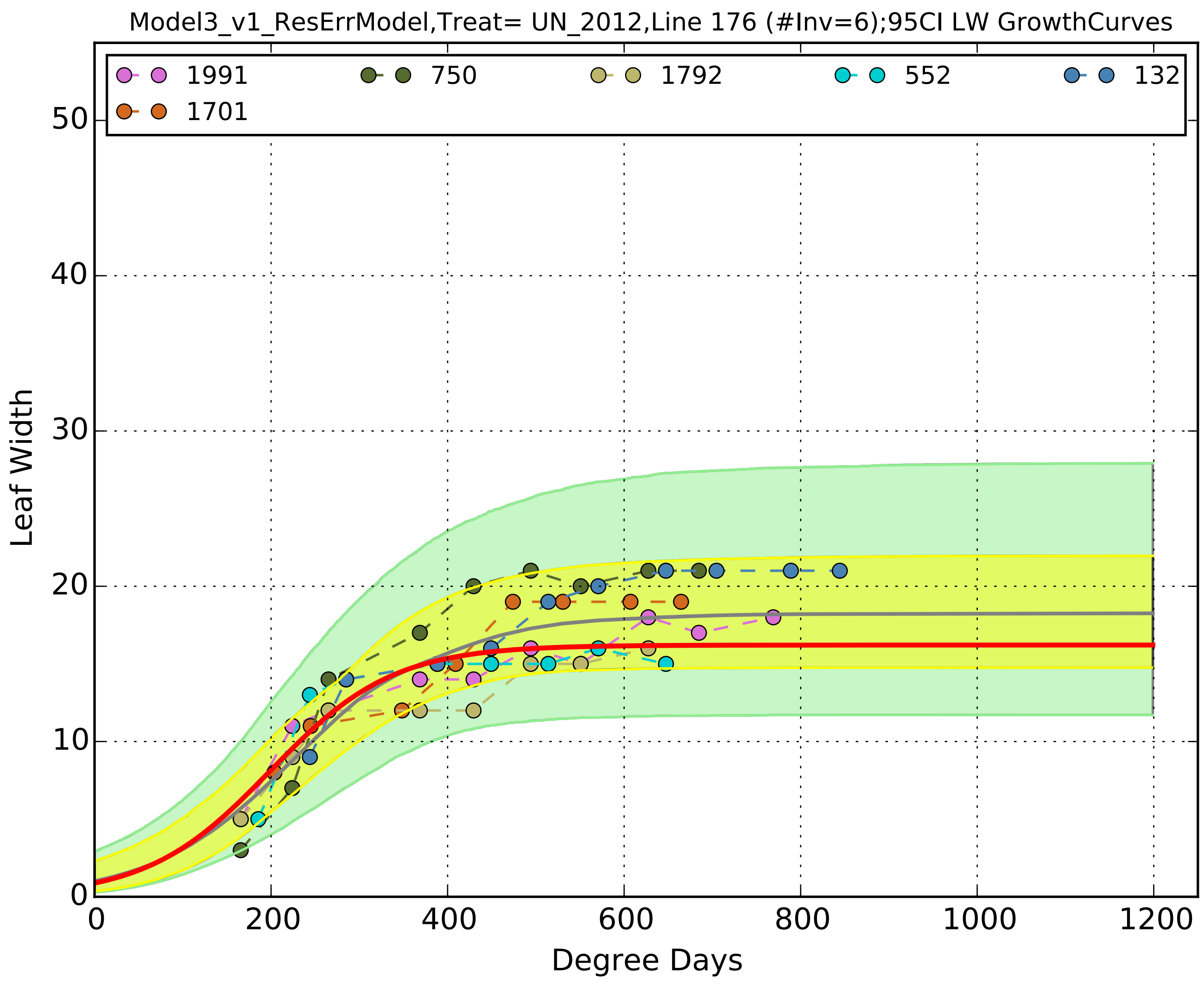
Model3_v1_ResErrModel,Treat= UN_2012,Line 176 (#Inv=6);95CI LW GrowthCurves
1991
750
1792
552
132
1701
Leaf Width
Degree Days
0
10
20
30
40
50
0
200
400
600
800
1000
1200

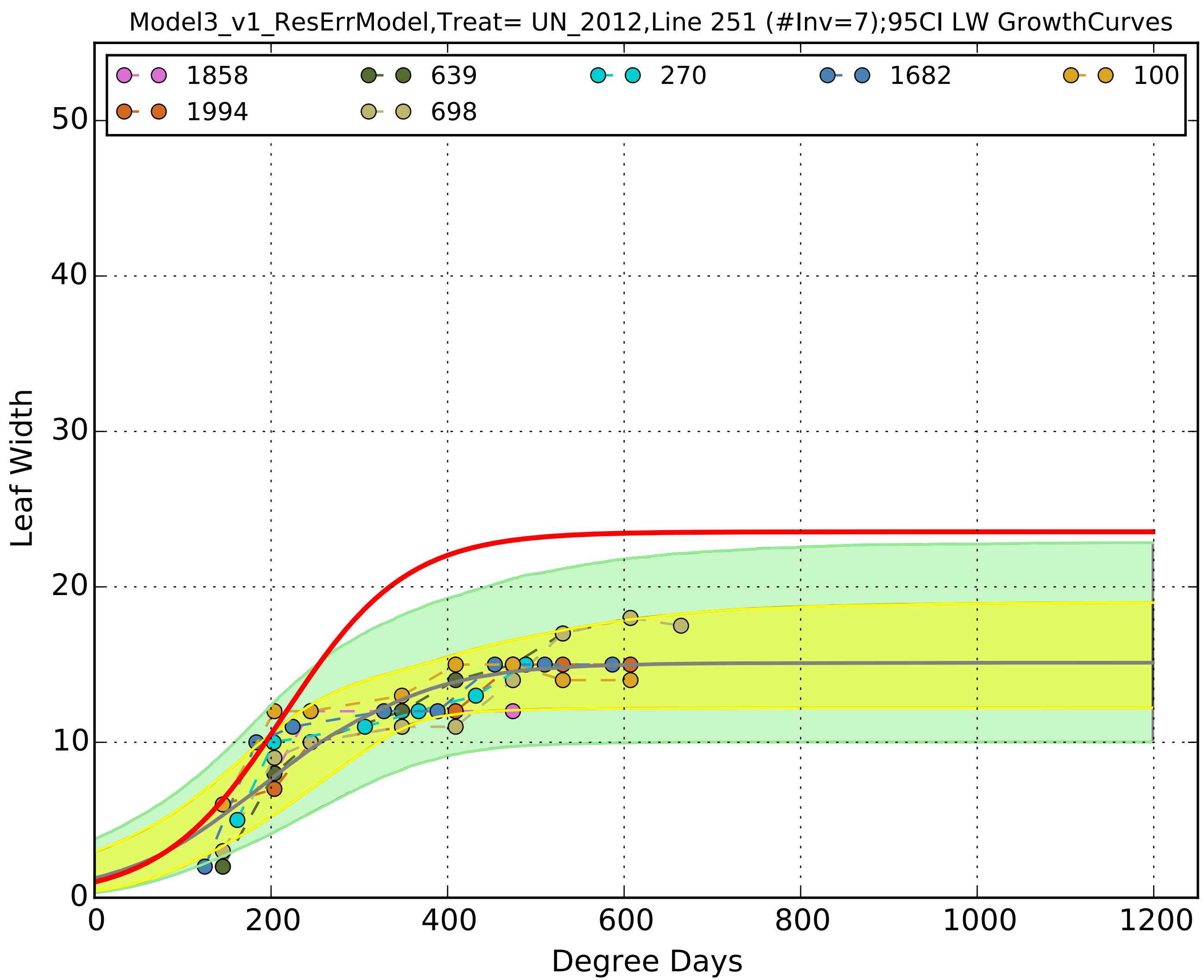

Model3_v1_ResErrModel,Treat= UN_2012,Line 251 (#Inv=7);95CI LW GrowthCurves
1858
639
270
1682
100
1994
698
Leaf Width
Degree Days
0
10
20
30
40
50
0
200
400
600
800
1000
1200

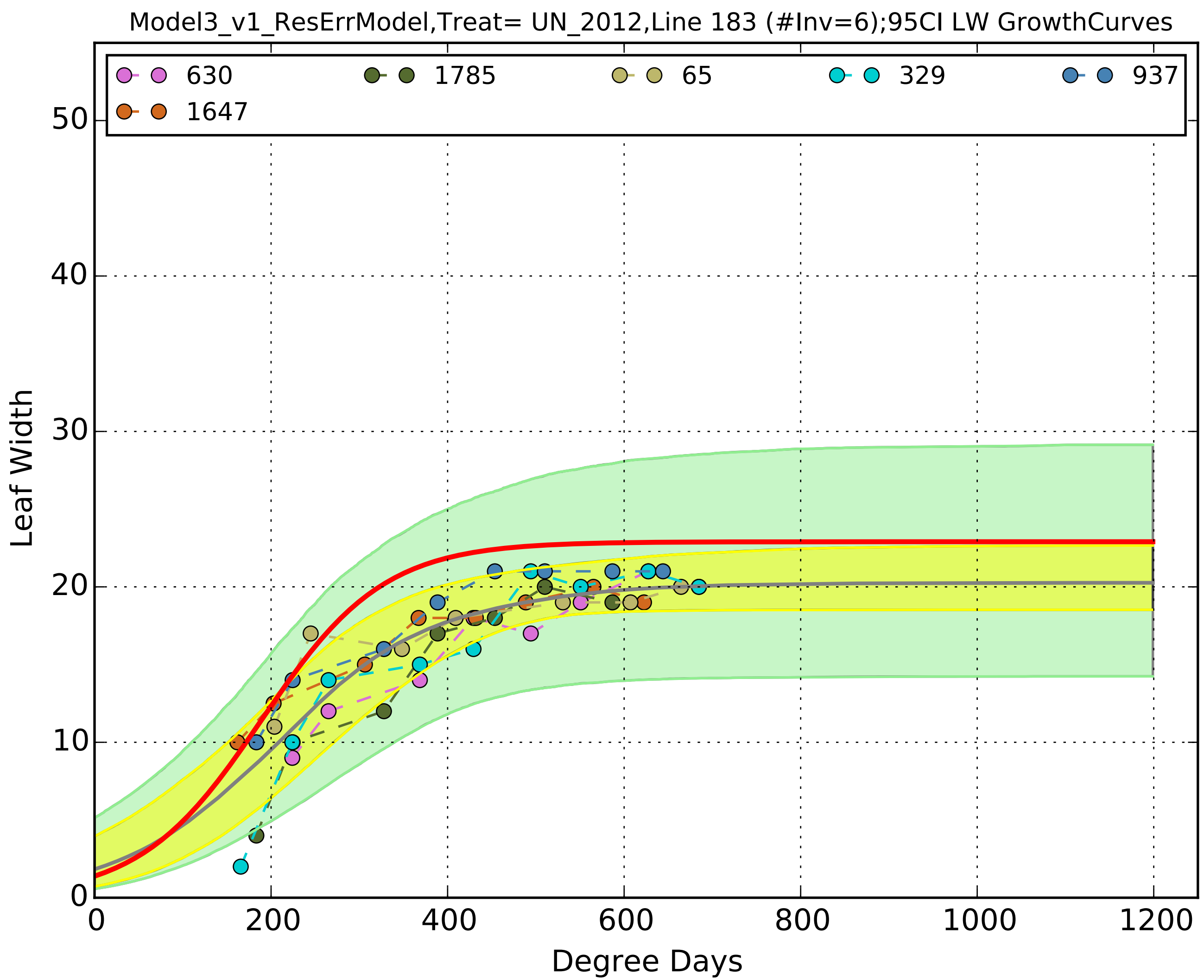

Model3_v1_ResErrModel,Treat= UN_2012,Line 183 (#Inv=6);95CI LW GrowthCurves
630
1785
65
329
937
1647
Leaf Width
Degree Days
0
10
20
30
40
50
0
200
400
600
800
1000
1200

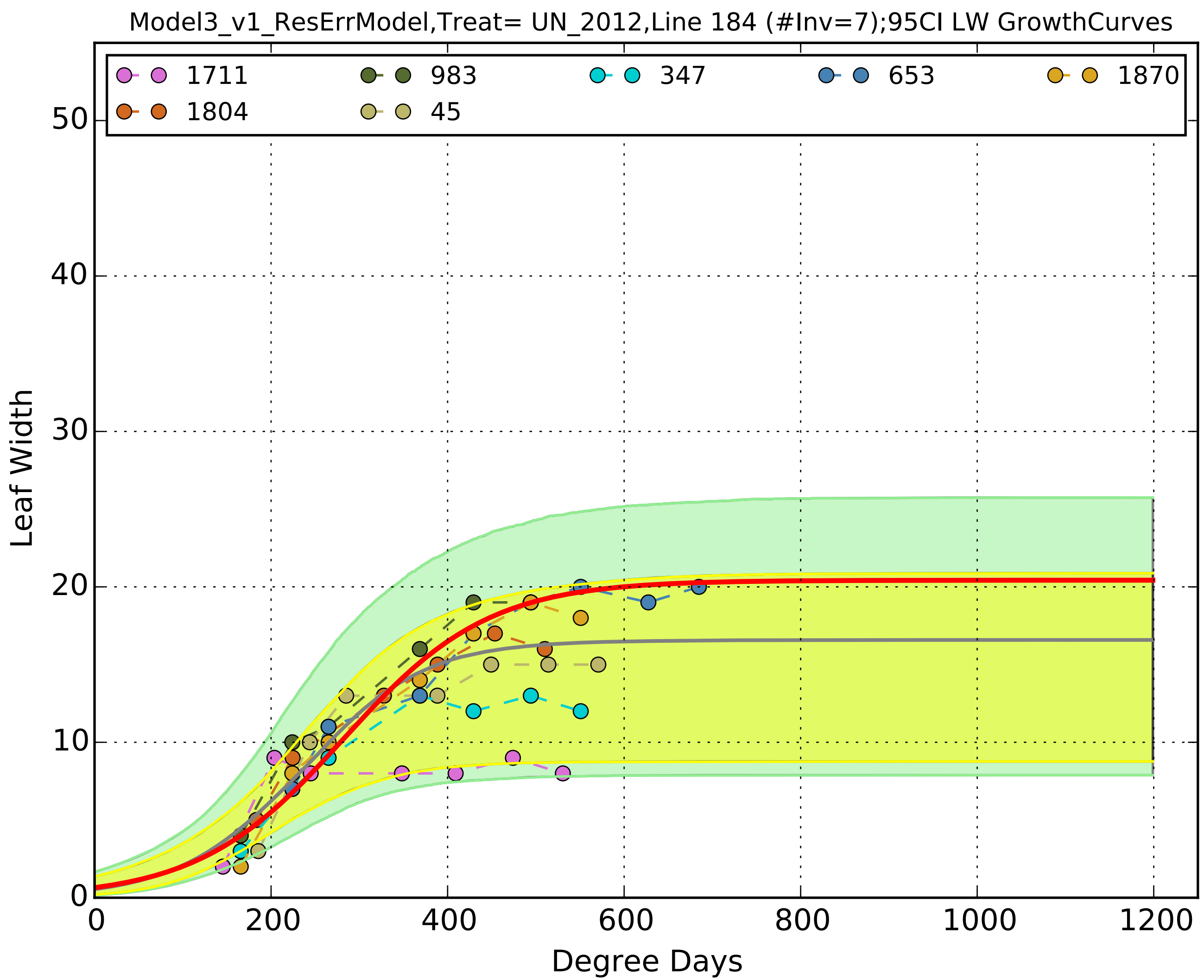
Model3_v1_ResErrModel,Treat= UN_2012,Line 184 (#Inv=7);95CI LW GrowthCurves
1711
983
347
653
1870
1804
45
Leaf Width
Degree Days
0
10
20
30
40
50
0
200
400
600
800
1000
1200

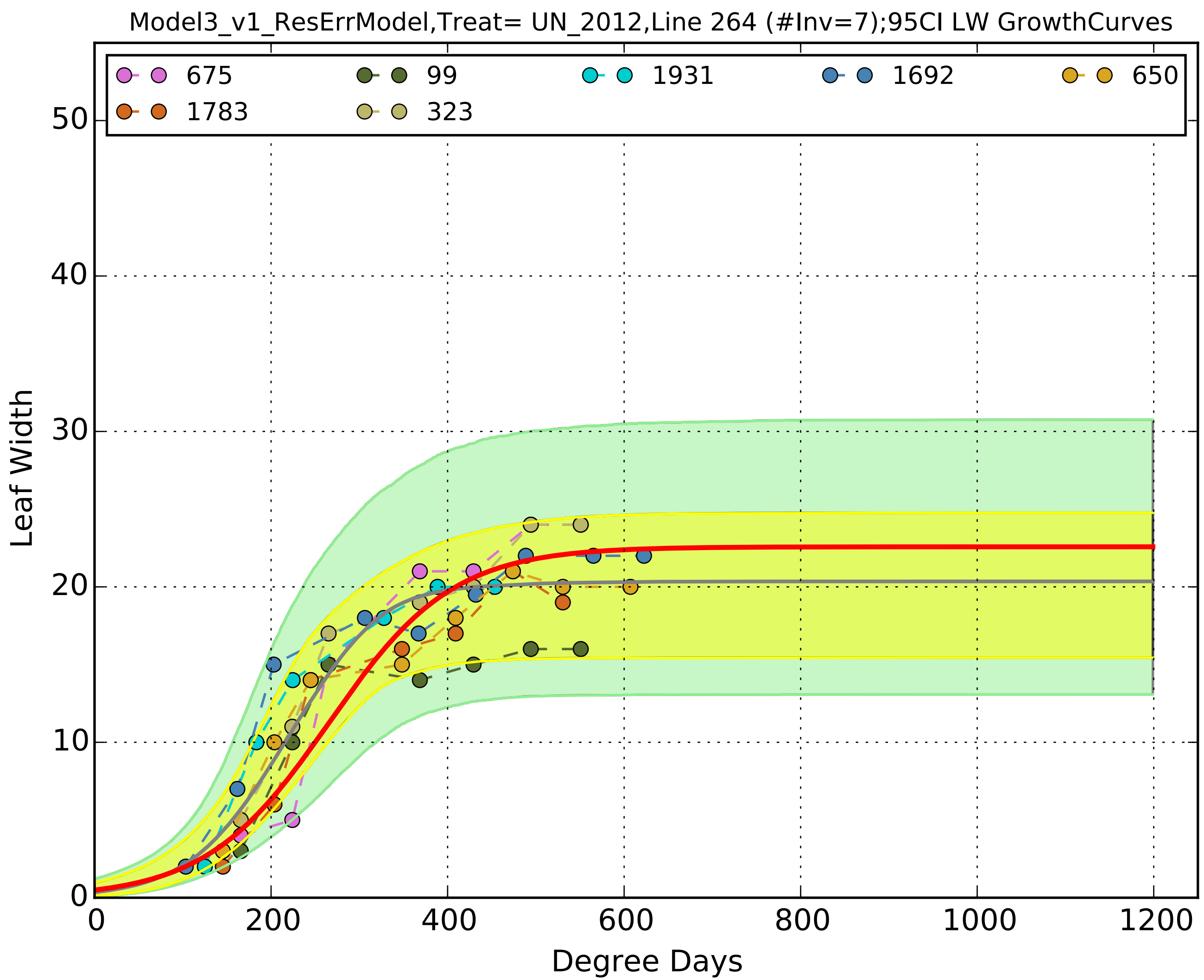

Model3_v1_ResErrModel,Treat= UN_2012,Line 264 (#Inv=7);95CI LW GrowthCurves
675
99
1931
1692
650
1783
323
Leaf Width
Degree Days
0
10
20
30
40
50
0
200
400
600
800
1000
1200

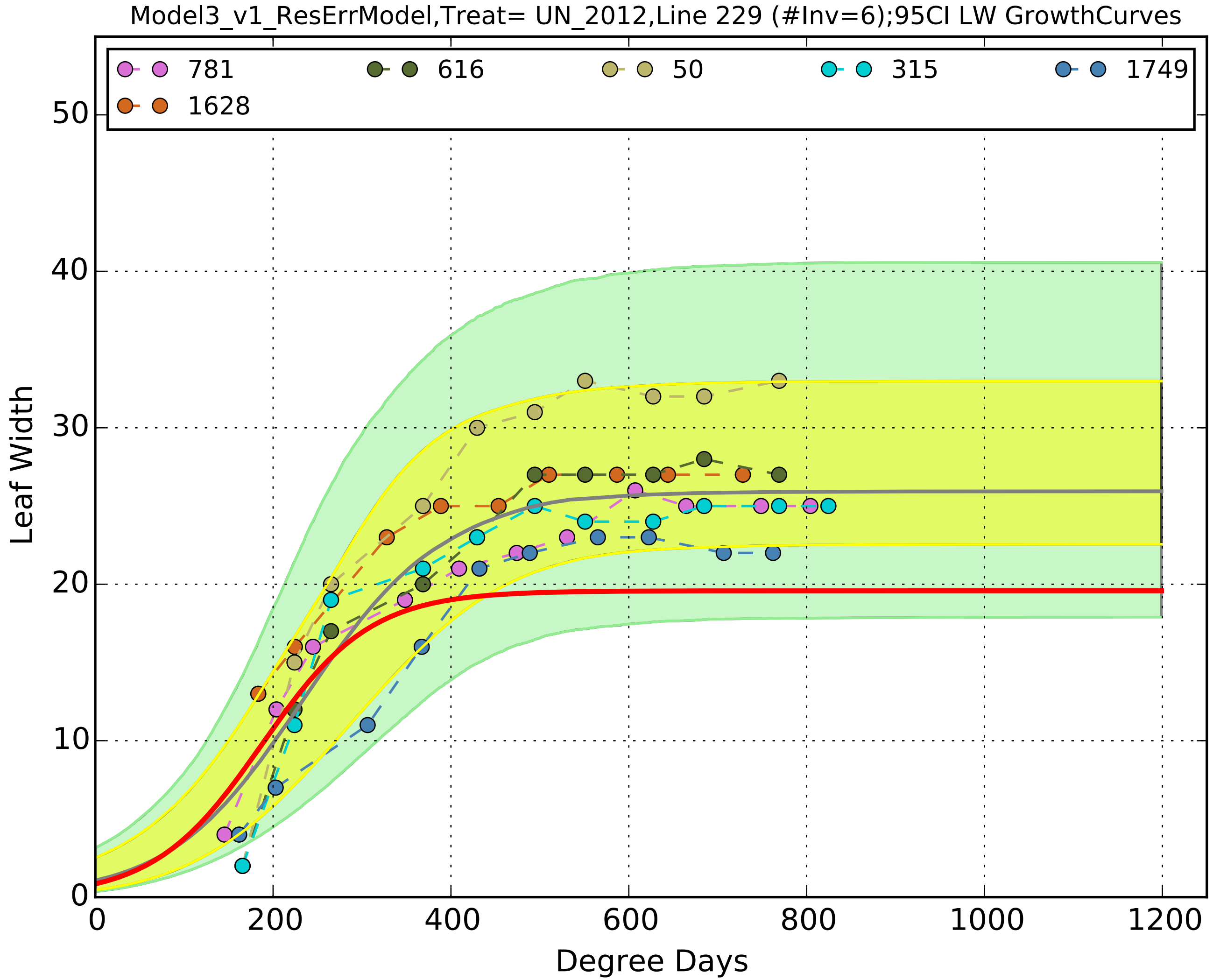
Model3_v1_ResErrModel,Treat= UN_2012,Line 229 (#Inv=6);95CI LW GrowthCurves
781
616
50
315
1749
1628
Leaf Width
Degree Days
0
10
20
30
40
50
0
200
400
600
800
1000
1200

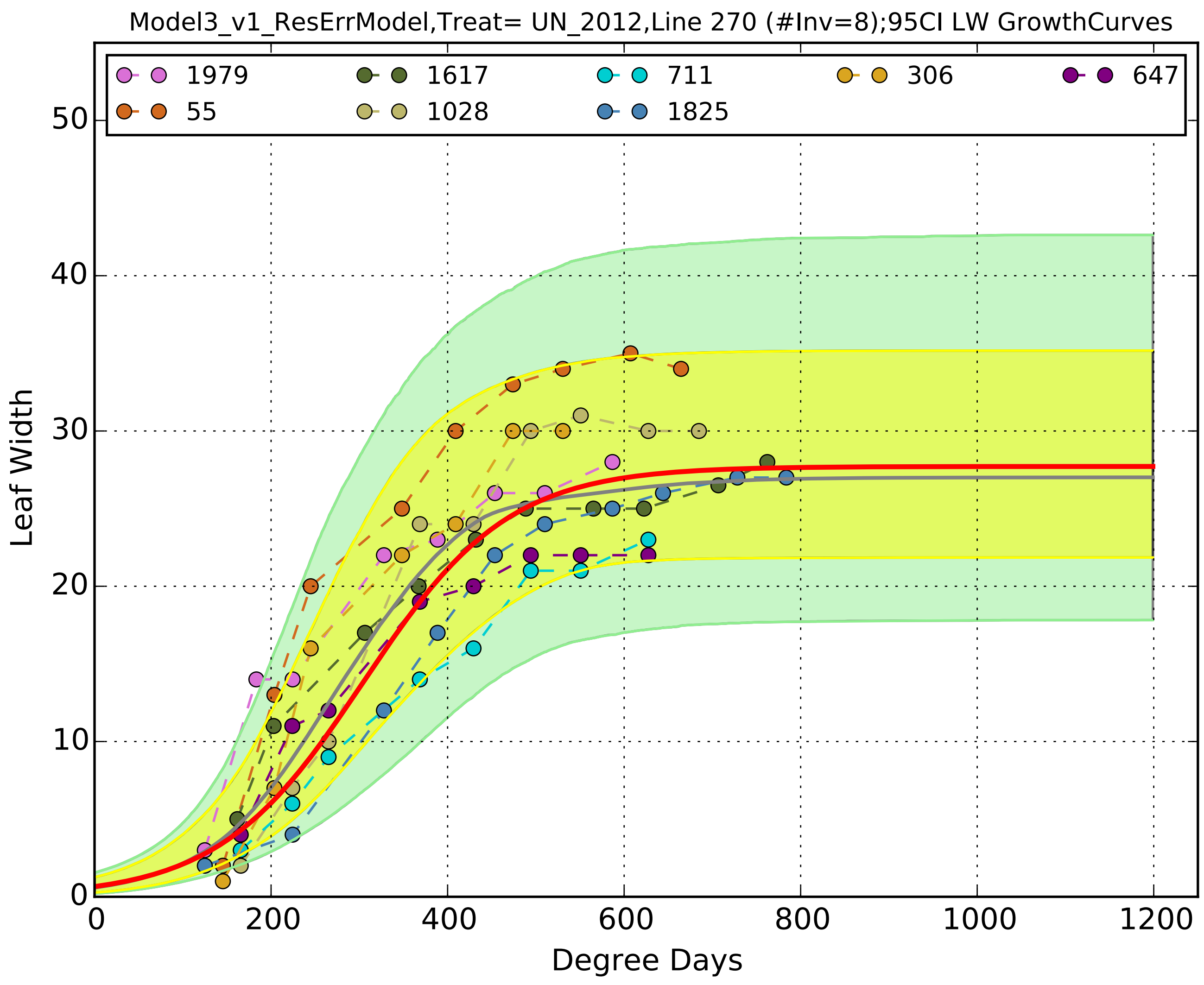
Model3_v1_ResErrModel,Treat= UN_2012,Line 270 (#Inv=8);95CI LW GrowthCurves
1979
1617
711
306
647
55
1028
1825
Leaf Width
Degree Days
0
10
20
30
40
50
0
200
400
600
800
1000
1200

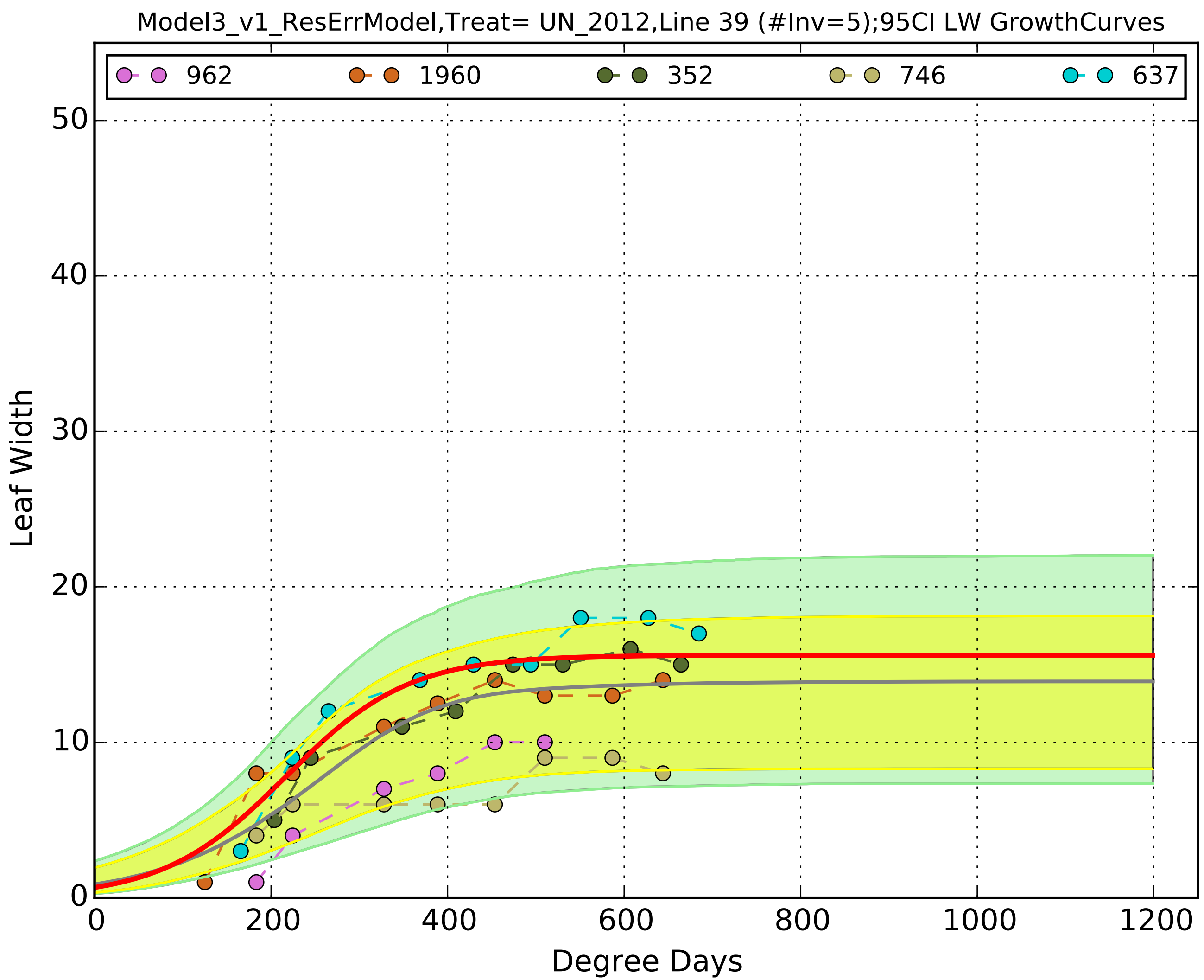

Model3_v1_ResErrModel,Treat= UN_2012,Line 39 (#Inv=5);95CI LW GrowthCurves
962
1960
352
746
637
Leaf Width
Degree Days
0
10
20
30
40
50
0
200
400
600
800
1000
1200

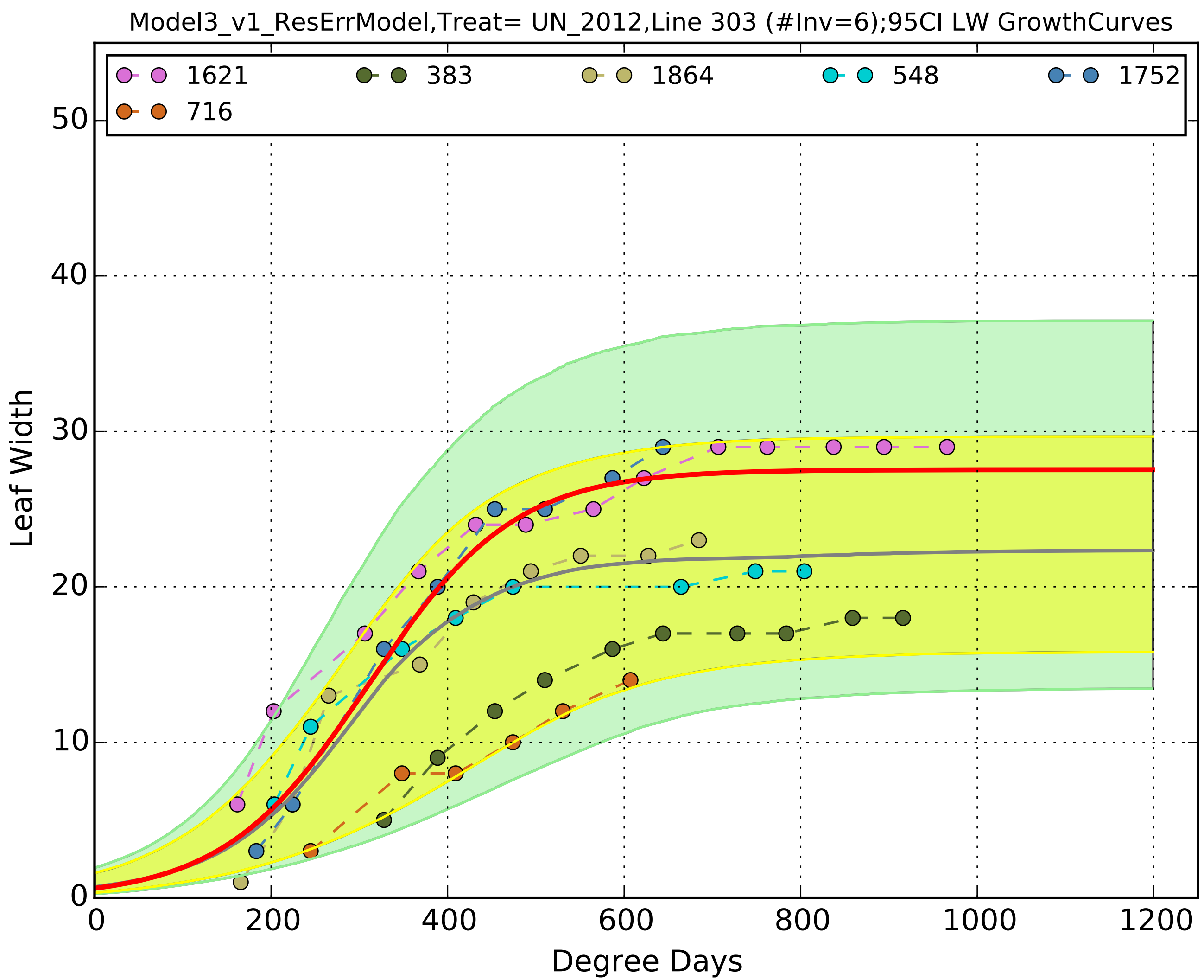

Model3_v1_ResErrModel,Treat= UN_2012,Line 303 (#Inv=6);95CI LW GrowthCurves
1621
383
1864
548
1752
716
Leaf Width
Degree Days
0
10
20
30
40
50
0
200
400
600
800
1000
1200

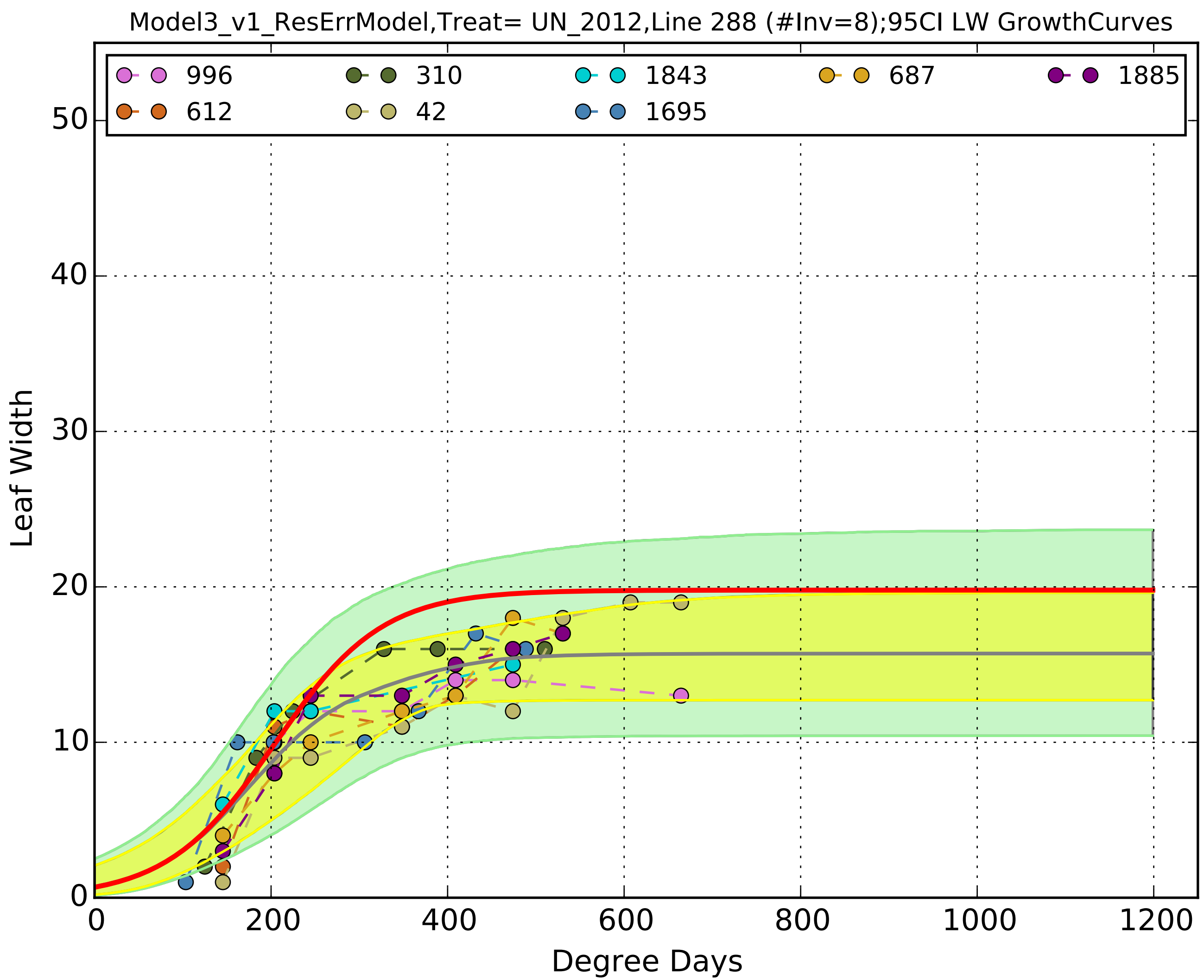

Model3_v1_ResErrModel,Treat= UN_2012,Line 288 (#Inv=8);95CI LW GrowthCurves
996
310
1843
687
1885
612
42
1695
Leaf Width
Degree Days
0
10
20
30
40
50
0
200
400
600
800
1000
1200

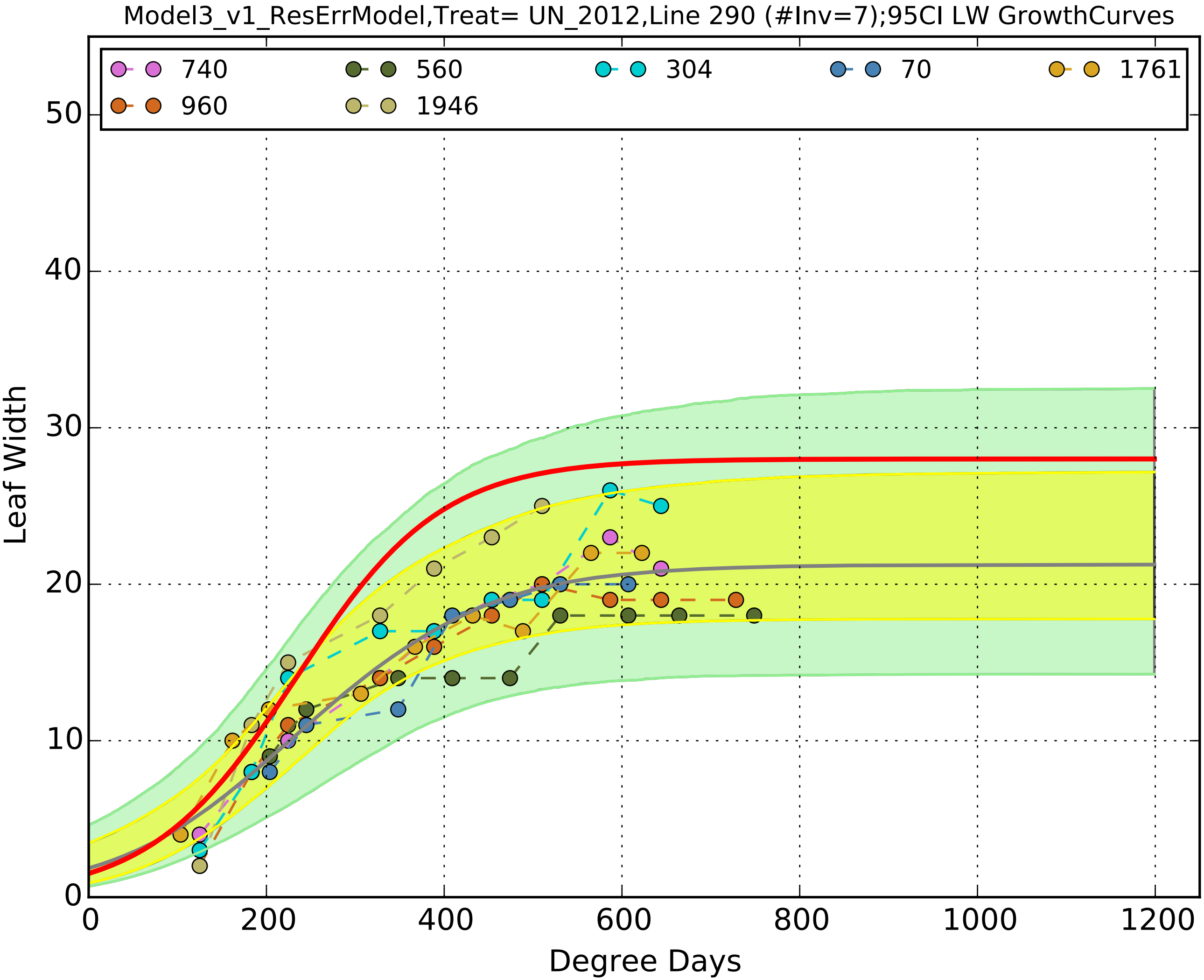

Model3_v1_ResErrModel,Treat= UN_2012,Line 290 (#Inv=7);95CI LW GrowthCurves
740
560
304
70
1761
960
1946
Leaf Width
Degree Days
0
10
20
30
40
50
0
200
400
600
800
1000
1200

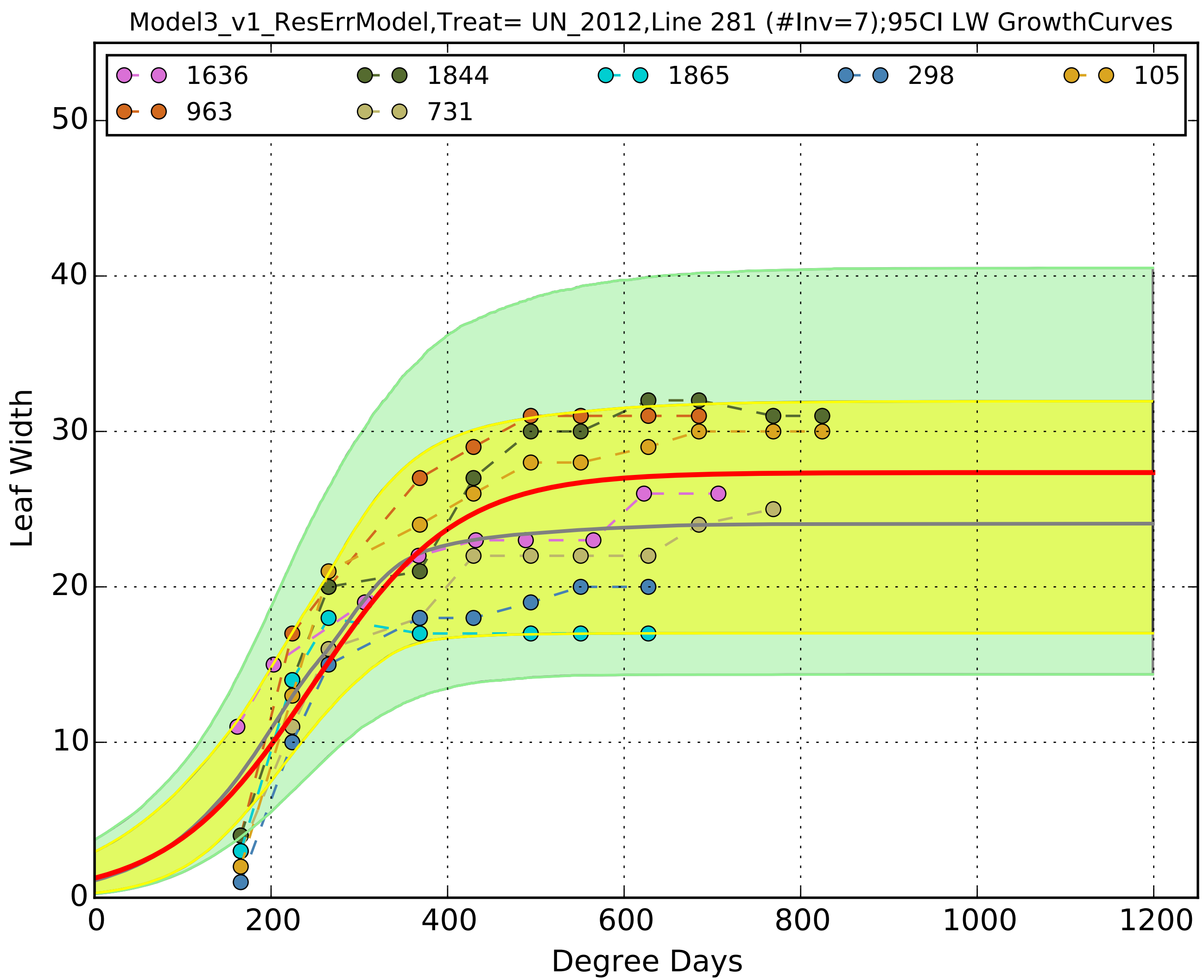

Model3_v1_ResErrModel,Treat= UN_2012,Line 281 (#Inv=7);95CI LW GrowthCurves
1636
1844
1865
298
105
963
731
Leaf Width
Degree Days
0
10
20
30
40
50
0
200
400
600
800
1000
1200

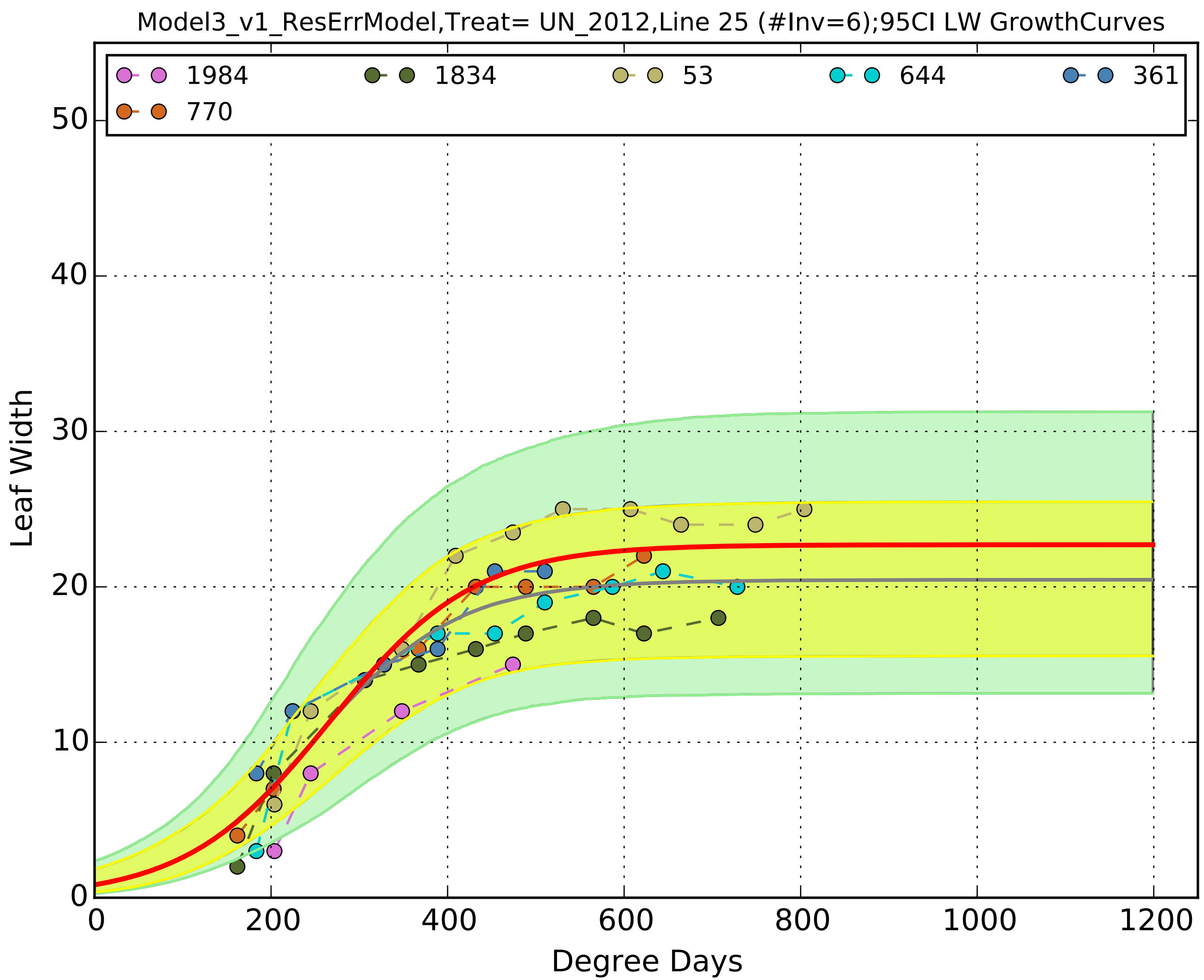

Model3_v1_ResErrModel,Treat= UN_2012,Line 25 (#Inv=6);95CI LW GrowthCurves
1984
1834
53
644
361
770
Leaf Width
Degree Days
0
10
20
30
40
50
0
200
400
600
800
1000
1200

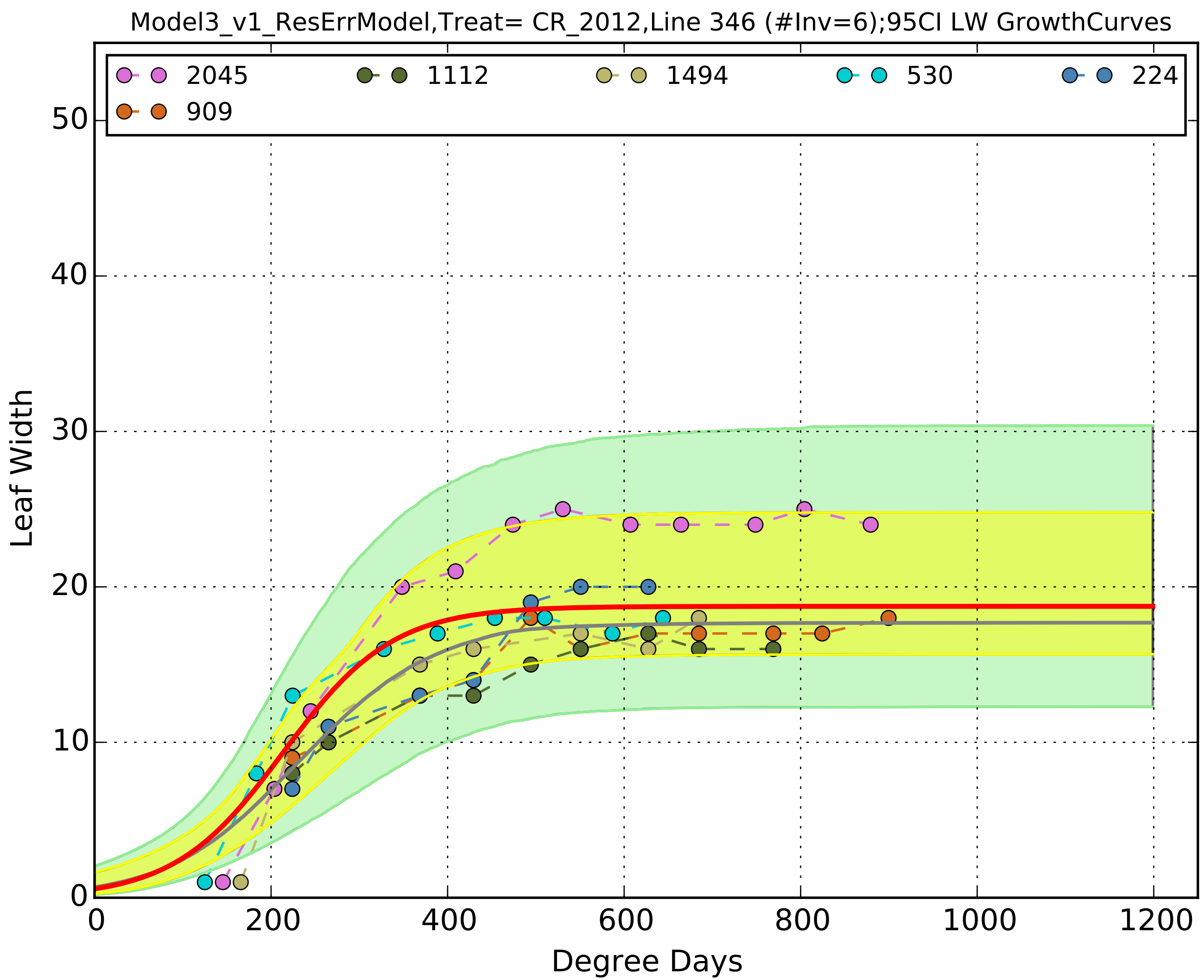

Model3_v1_ResErrModel,Treat= CR_2012,Line 346 (#Inv=6);95CI LW GrowthCurves
2045
1112
1494
530
224
909
Leaf Width
Degree Days
0
10
20
30
40
50
0
200
400
600
800
1000
1200

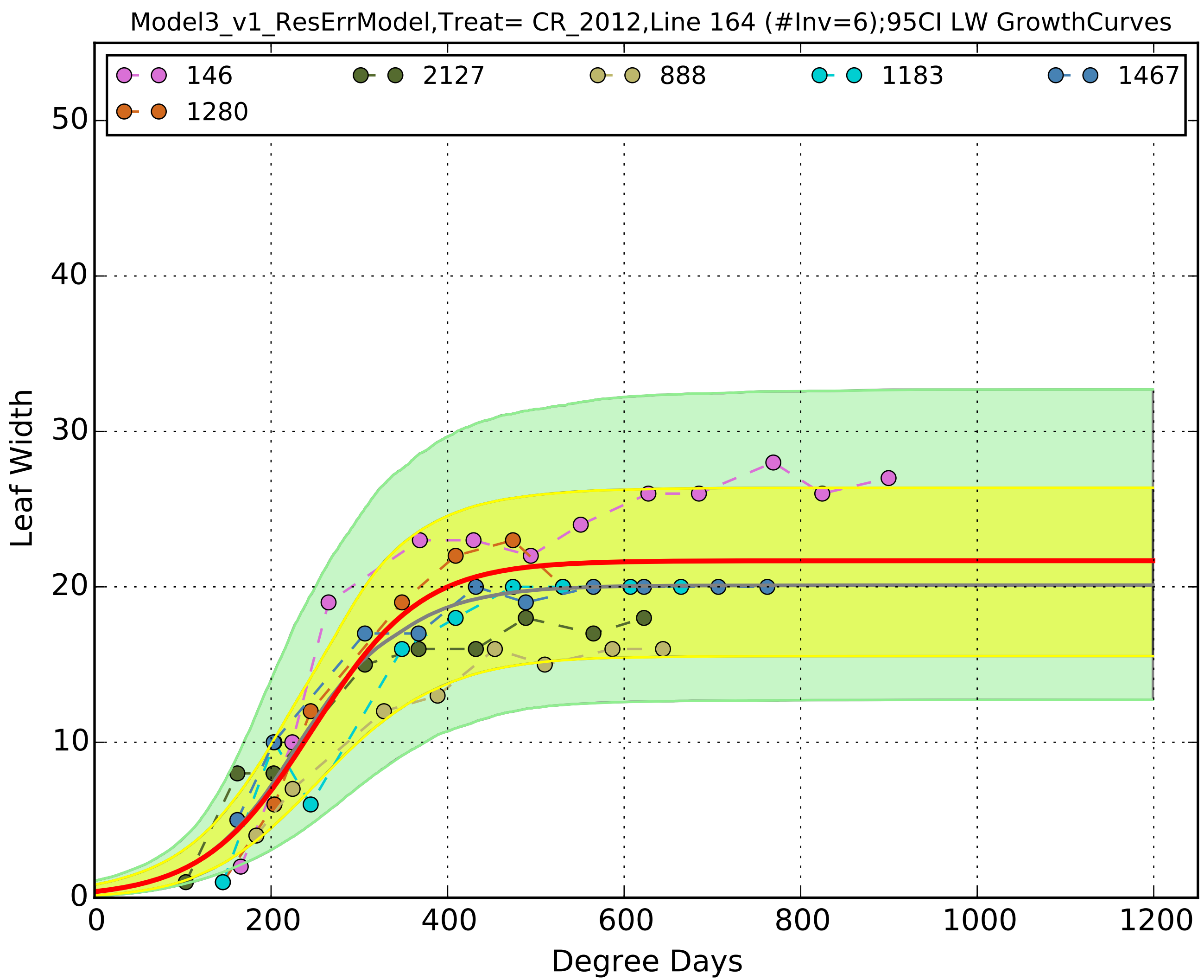

Model3_v1_ResErrModel,Treat= CR_2012,Line 164 (#Inv=6);95CI LW GrowthCurves
146
2127
888
1183
1467
1280
0
10
20
30
40
50
Leaf Width
0
200
400
600
800
1000
1200
Degree Days

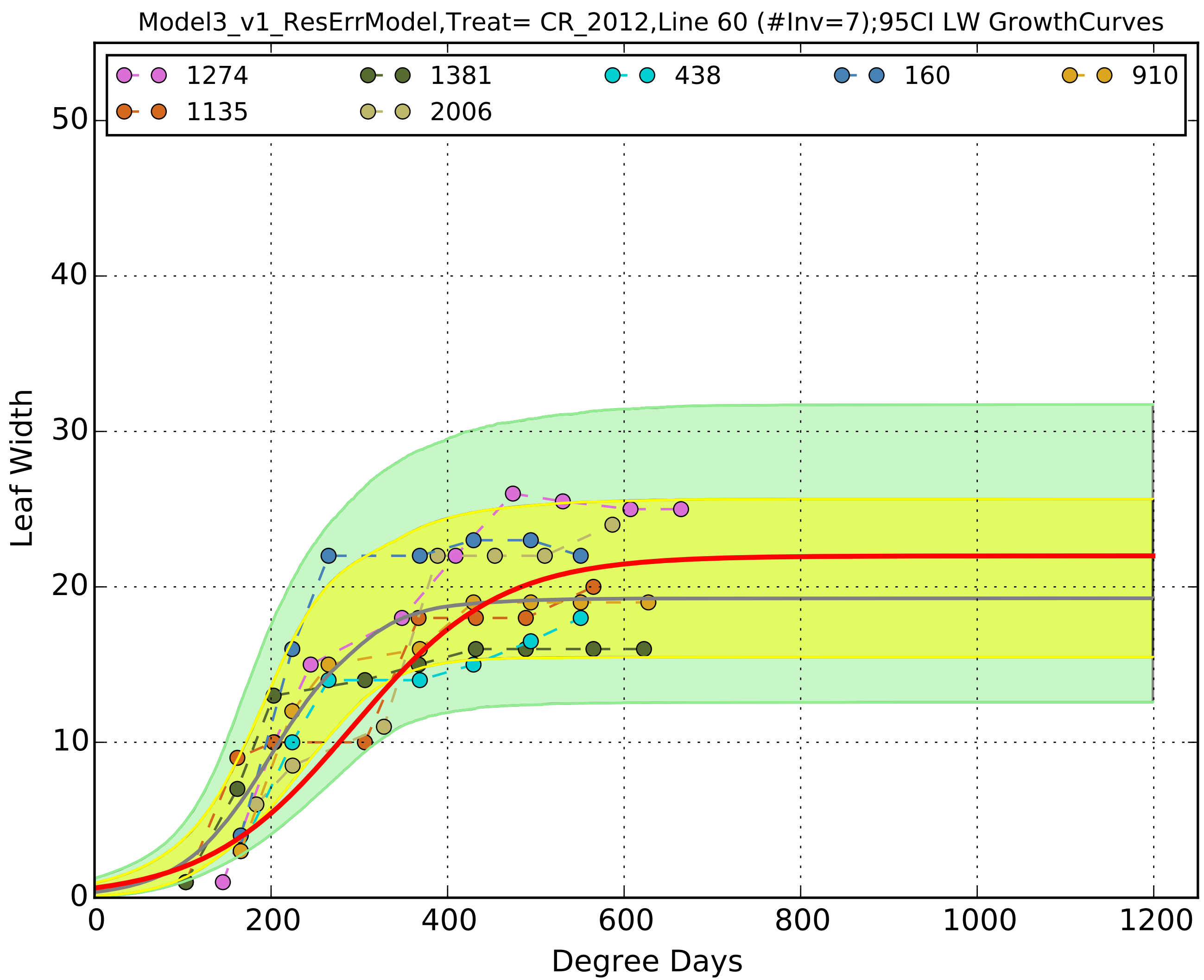
Model3_v1_ResErrModel,Treat= CR_2012,Line 60 (#Inv=7);95CI LW GrowthCurves
1274
1381
438
160
910
1135
2006
Leaf Width
Degree Days
0
10
20
30
40
50
0
200
400
600
800
1000
1200

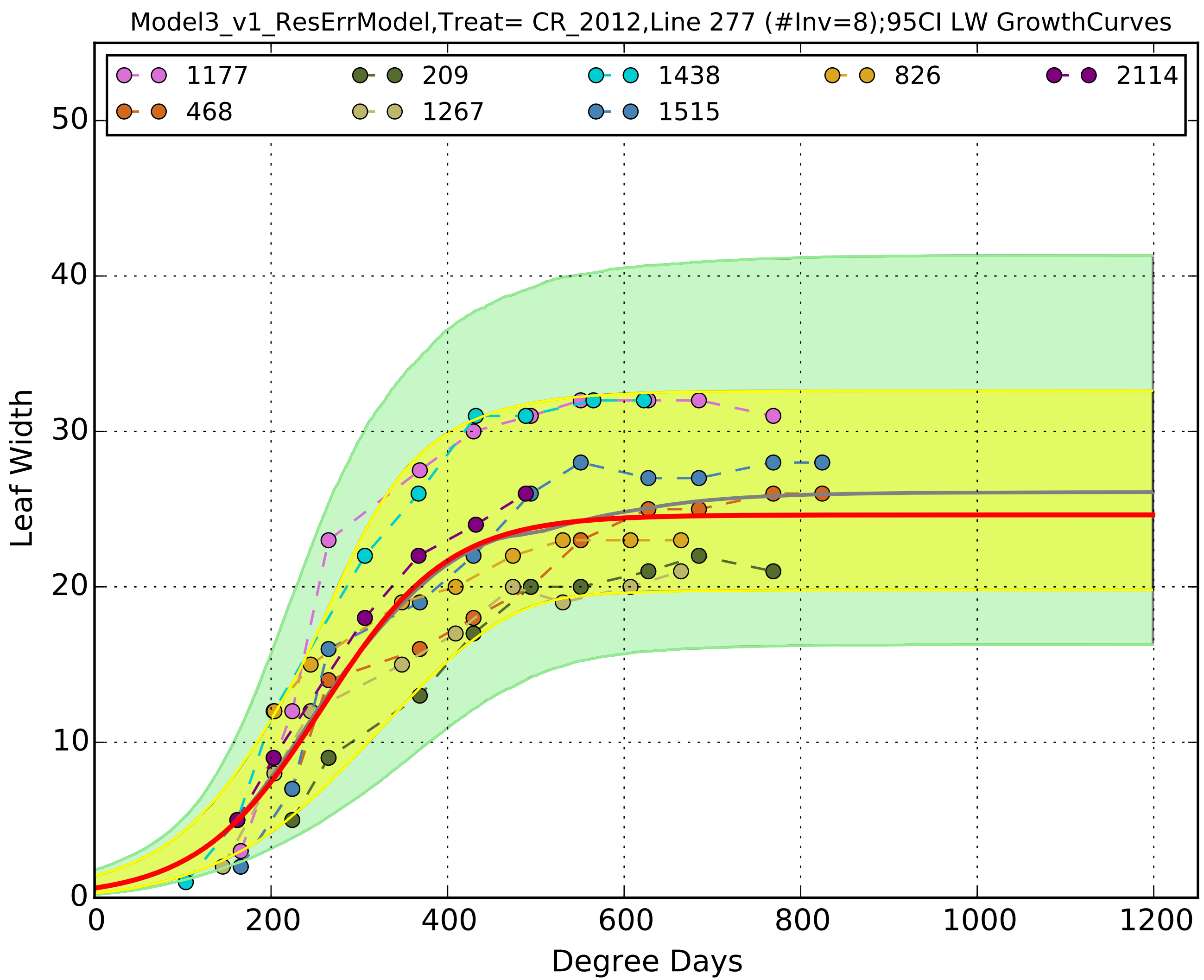
Model3_v1_ResErrModel,Treat= CR_2012,Line 277 (#Inv=8);95CI LW GrowthCurves
1177
209
1438
826
2114
468
1267
1515
Leaf Width
Degree Days
0
10
20
30
40
50
0
200
400
600
800
1000
1200

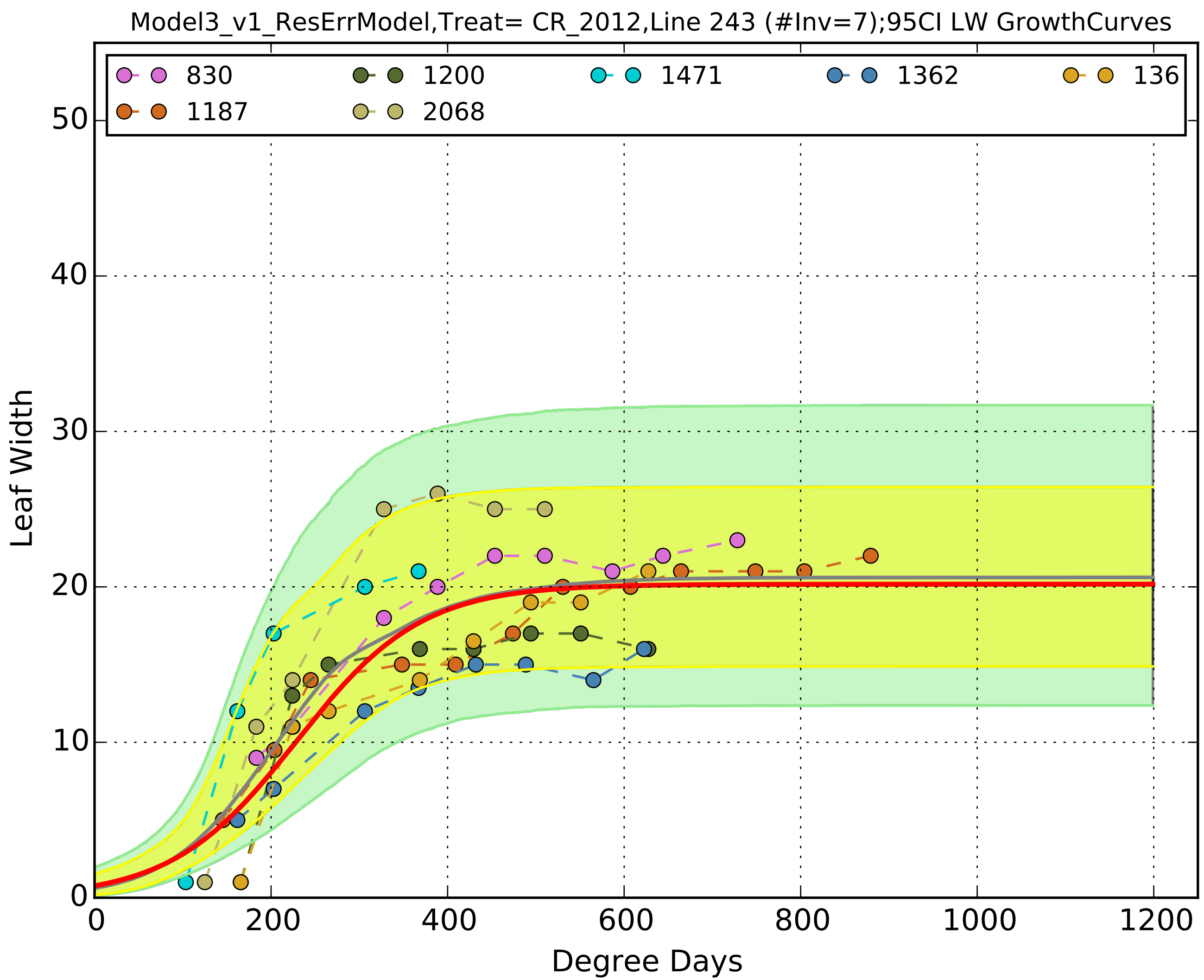

Model3_v1_ResErrModel,Treat= CR_2012,Line 243 (#Inv=7);95CI LW GrowthCurves
830
1200
1471
1362
136
1187
2068
Leaf Width
0
10
20
30
40
50
0
200
400
600
800
1000
1200
Degree Days

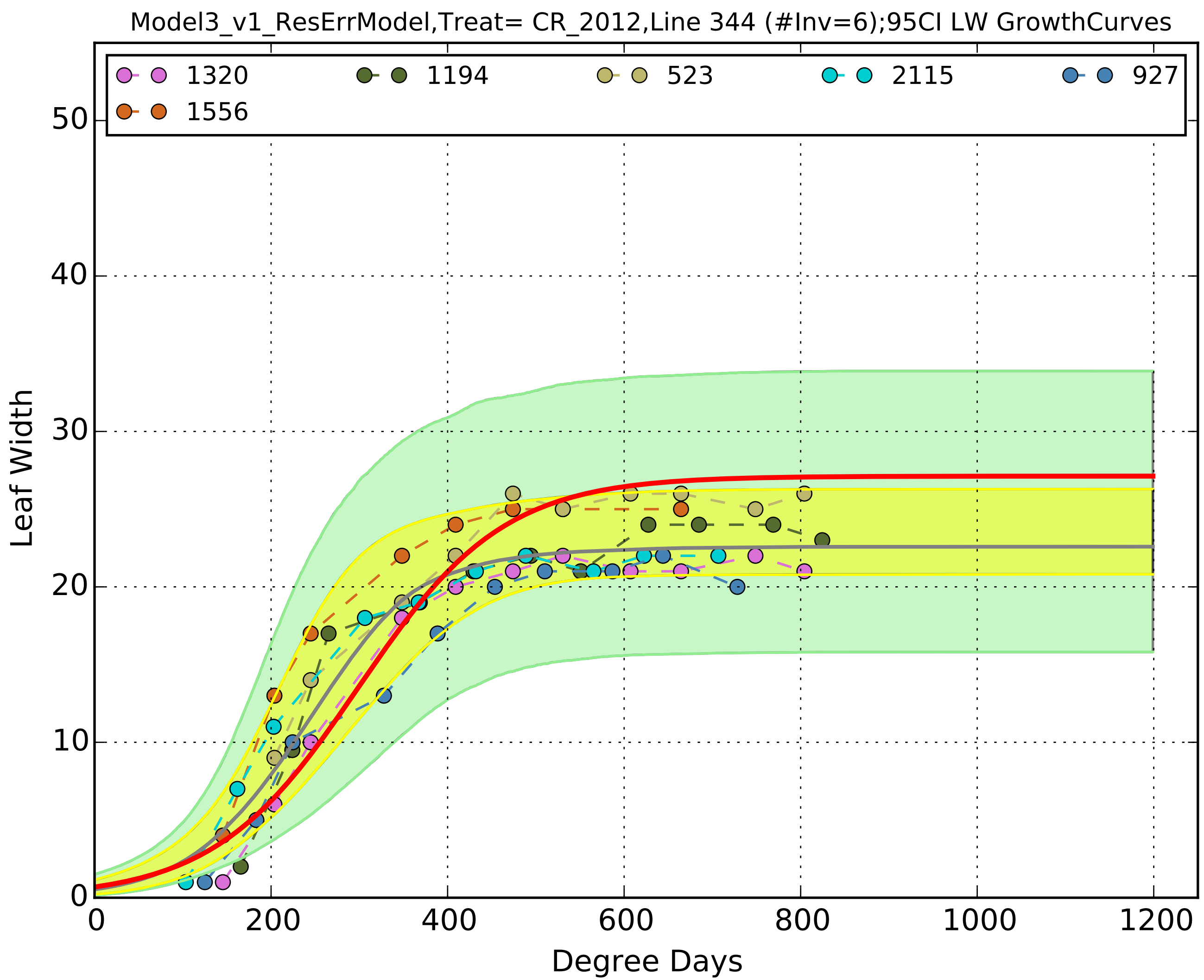

Model3_v1_ResErrModel,Treat= CR_2012,Line 344 (#Inv=6);95CI LW GrowthCurves
1320
1194
523
2115
927
1556
Leaf Width
Degree Days
0
10
20
30
40
50
0
200
400
600
800
1000
1200

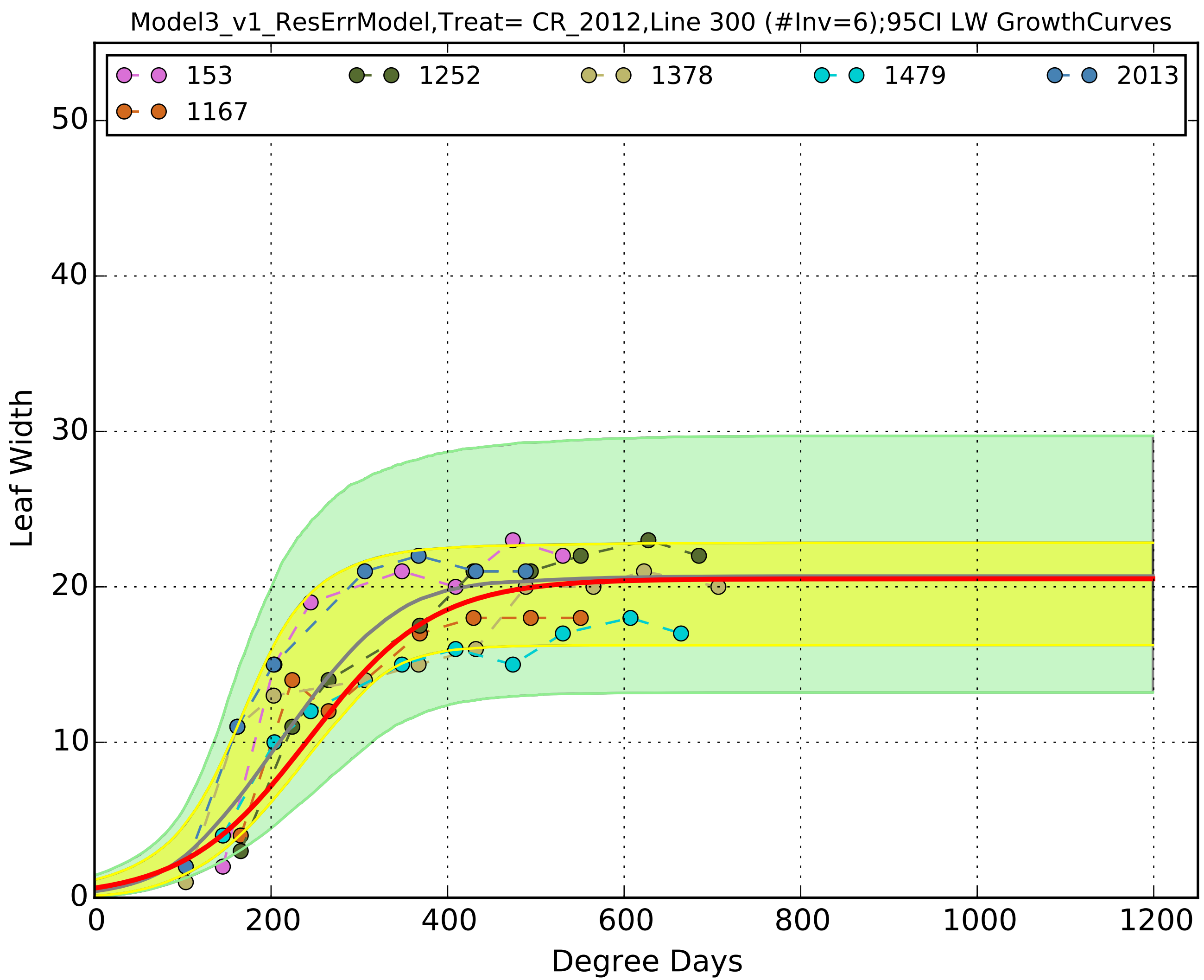
Model3_v1_ResErrModel,Treat= CR_2012,Line 300 (#Inv=6);95CI LW GrowthCurves
153
1252
1378
1479
2013
1167
Leaf Width
0
10
20
30
40
50
0
200
400
600
800
1000
1200
Degree Days

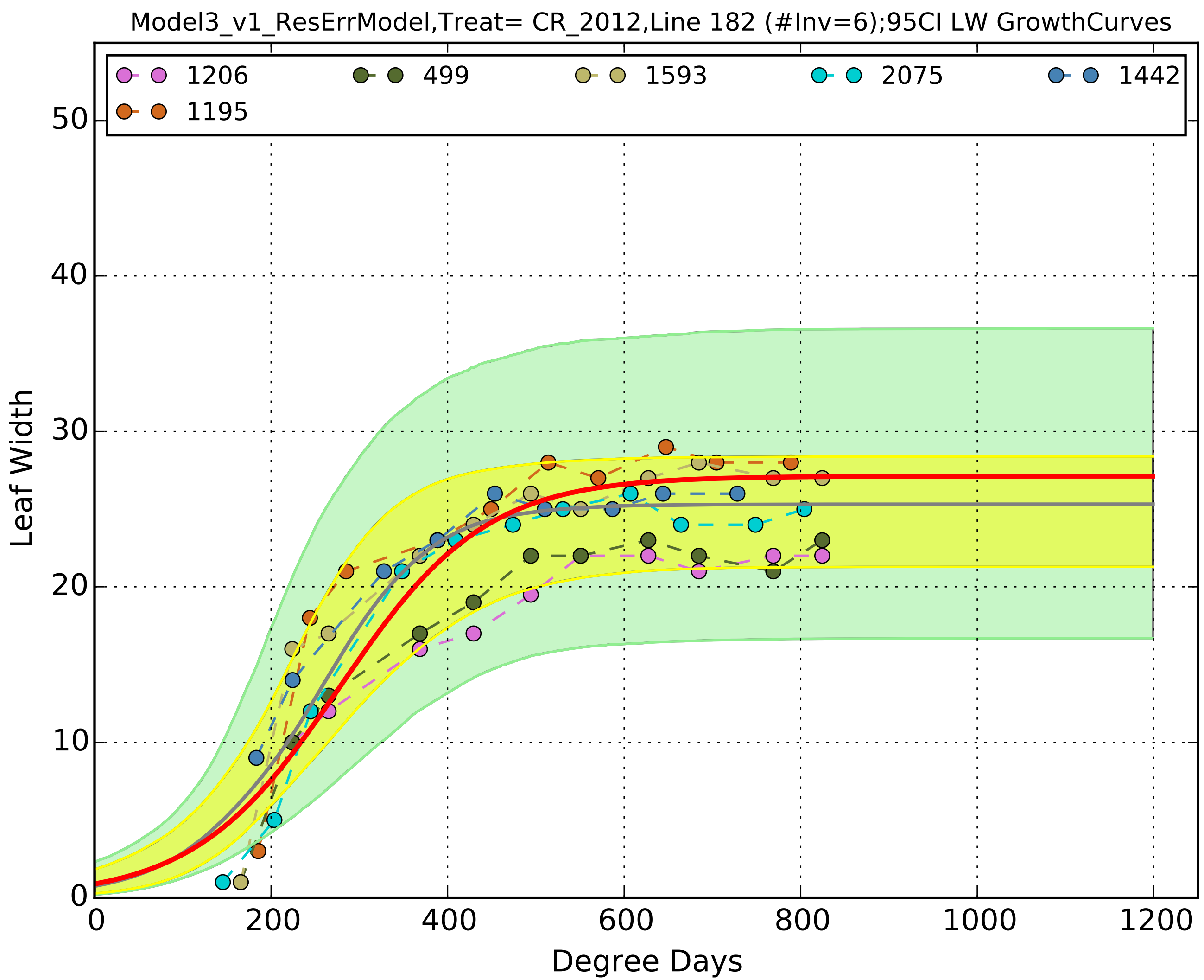
Model3_v1_ResErrModel,Treat= CR_2012,Line 182 (#Inv=6);95CI LW GrowthCurves
1206
499
1593
2075
1442
1195
Leaf Width
Degree Days
0
10
20
30
40
50
0
200
400
600
800
1000
1200

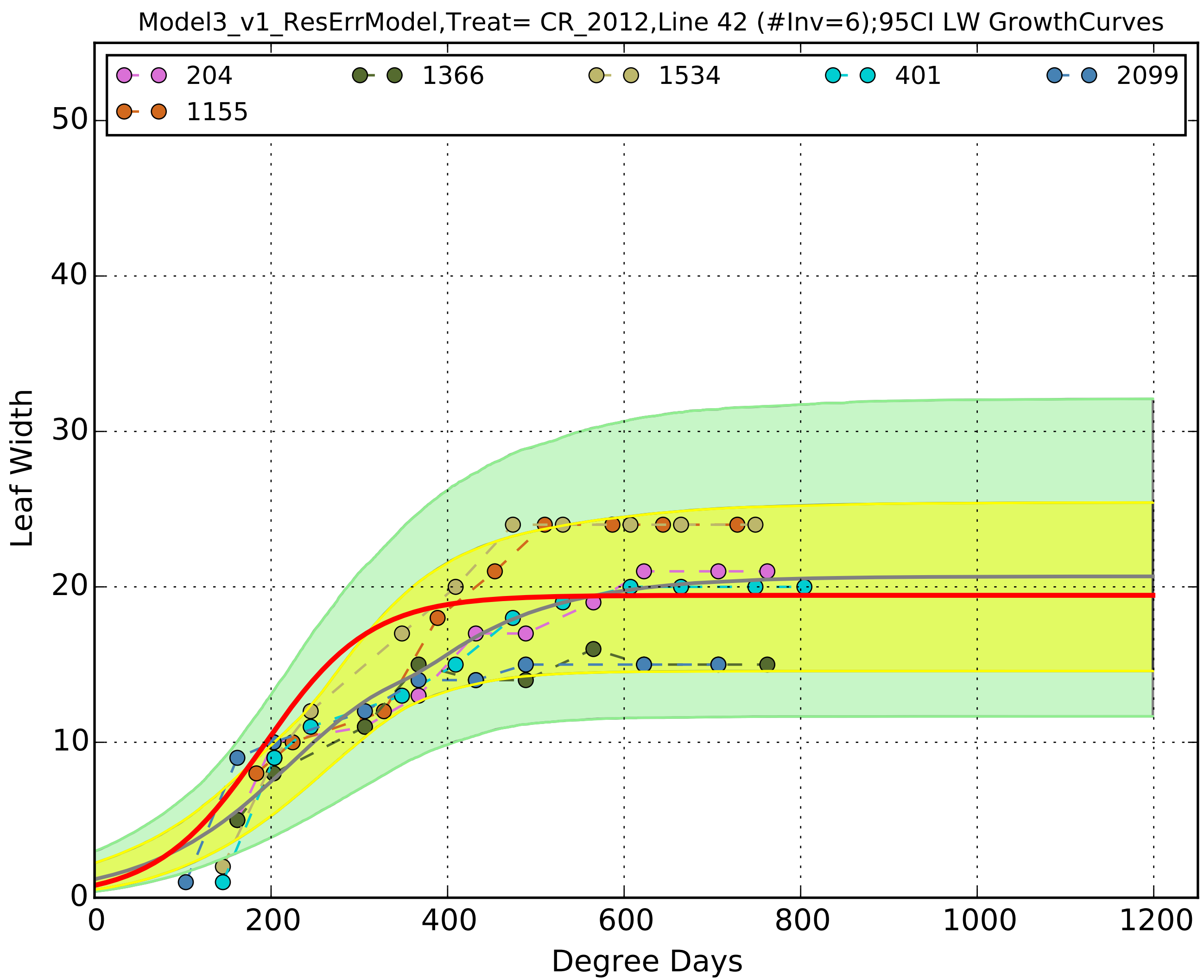
Model3_v1_ResErrModel,Treat= CR_2012,Line 42 (#Inv=6);95CI LW GrowthCurves
204
1366
1534
401
2099
1155
Leaf Width
Degree Days
0
10
20
30
40
50
0
200
400
600
800
1000
1200

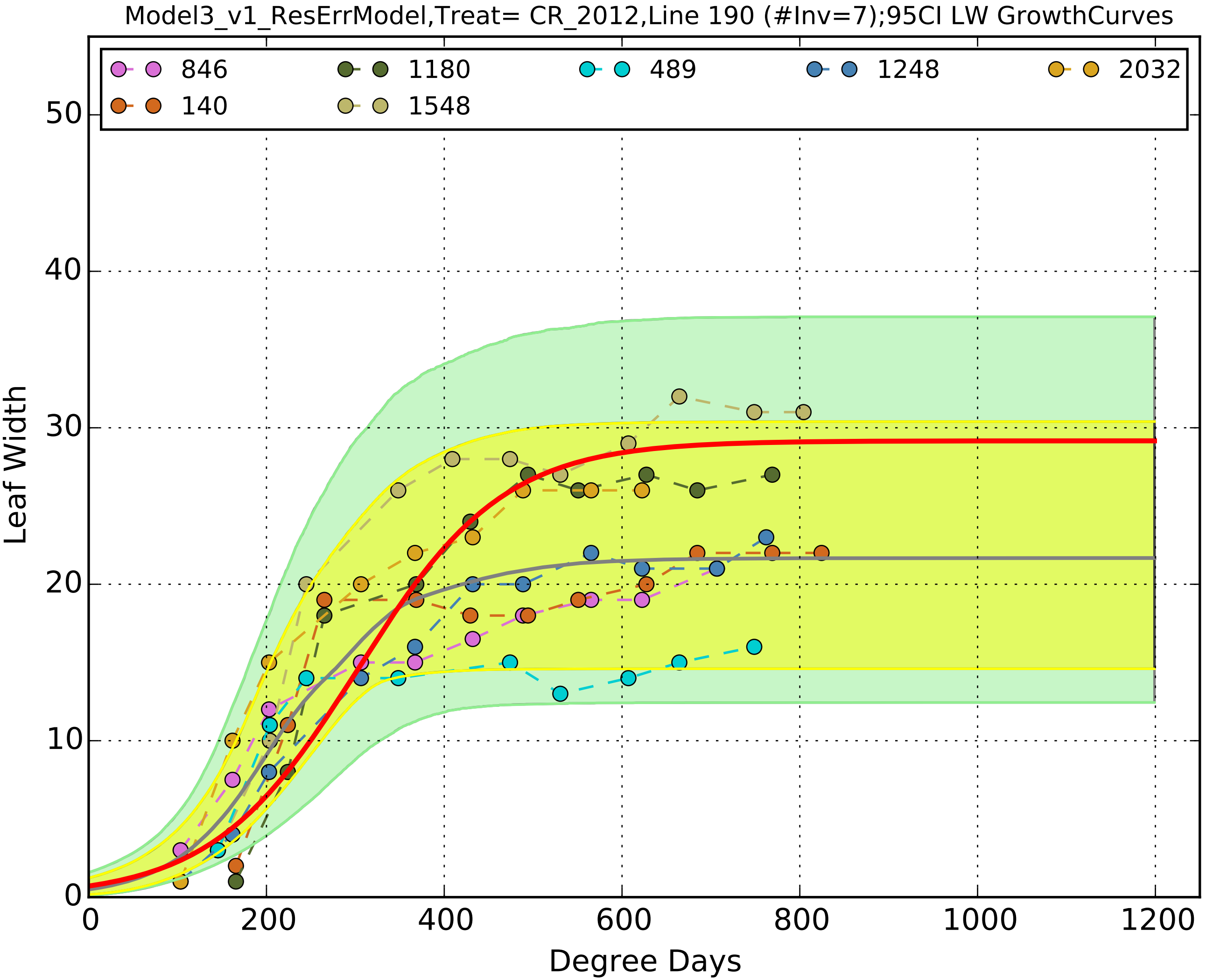

Model3_v1_ResErrModel,Treat= CR_2012,Line 190 (#Inv=7);95CI LW GrowthCurves
846
1180
489
1248
2032
140
1548
Leaf Width
Degree Days
0
10
20
30
40
50
0
200
400
600
800
1000
1200

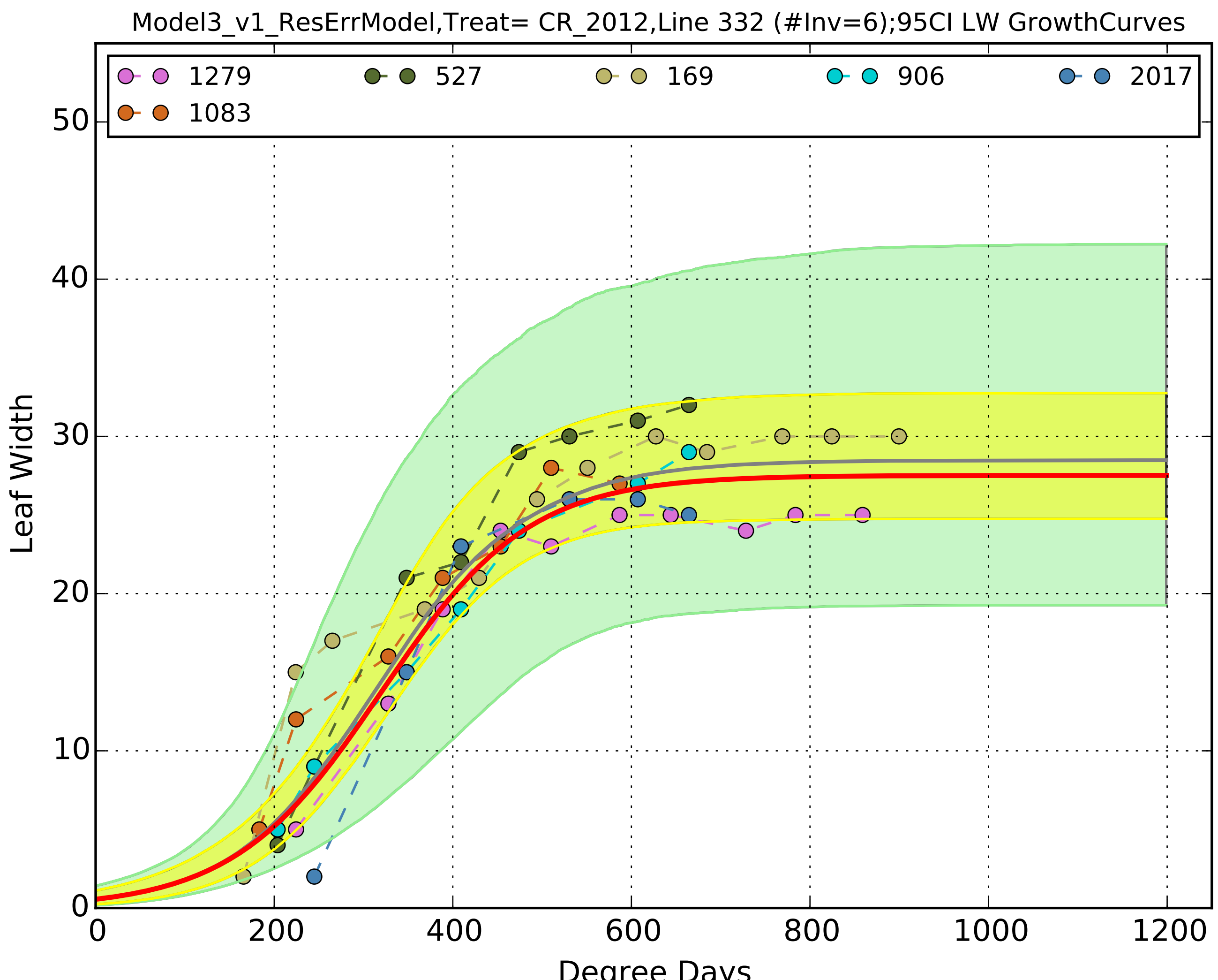
Model3_v1_ResErrModel,Treat= CR_2012,Line 332 (#Inv=6);95CI LW GrowthCurves
1279
527
169
906
2017
1083
Leaf Width
Degree Days
0
10
20
30
40
50
0
200
400
600
800
1000
1200

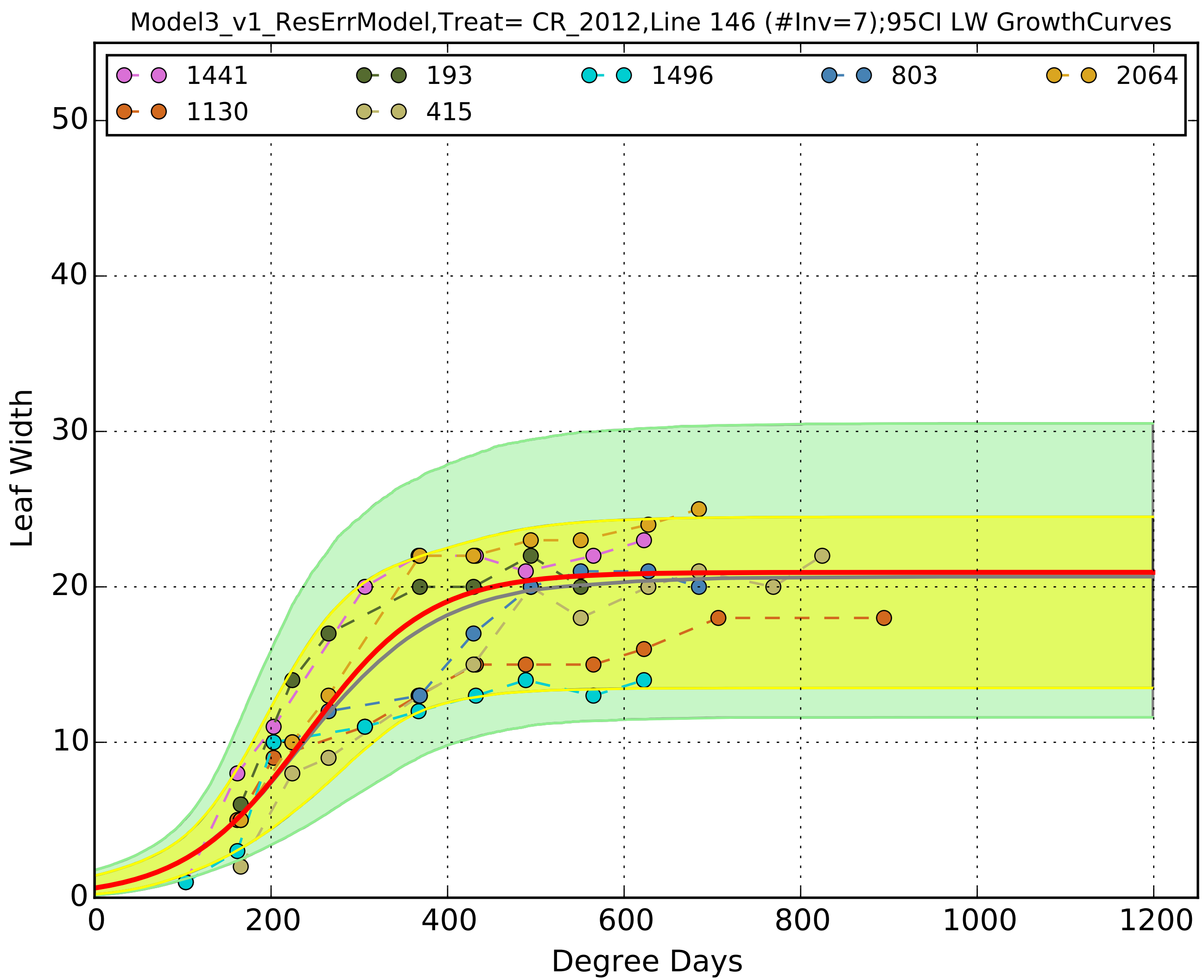

Model3_v1_ResErrModel,Treat= CR_2012,Line 146 (#Inv=7);95CI LW GrowthCurves
1441
193
1496
803
2064
1130
415
Leaf Width
Degree Days
0
10
20
30
40
50
200
400
600
800
1000
1200

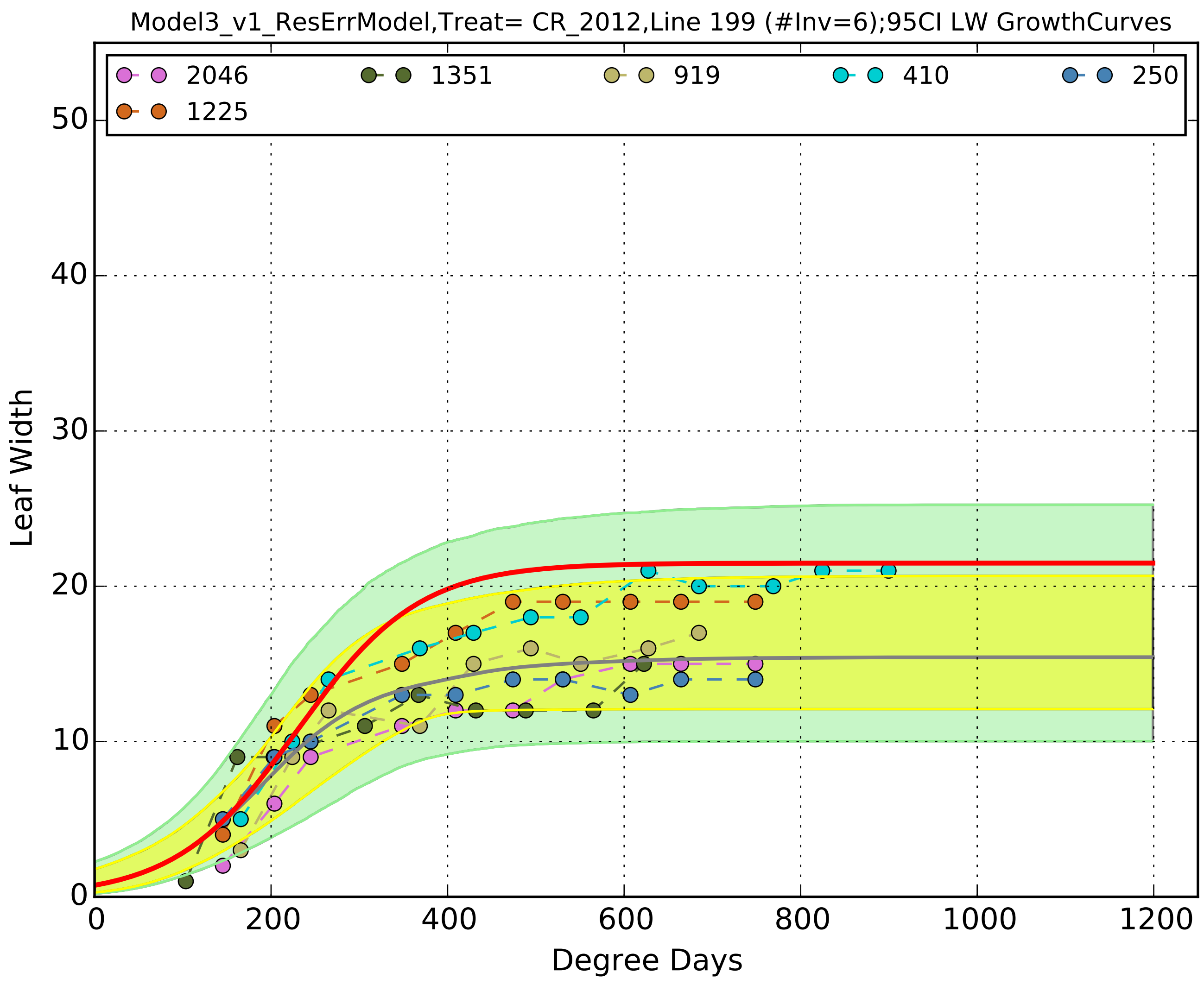

Model3_v1_ResErrModel,Treat= CR_2012,Line 199 (#Inv=6);95CI LW GrowthCurves
2046
1351
919
410
250
1225
Leaf Width
Degree Days
0
10
20
30
40
50
0
200
400
600
800
1000
1200

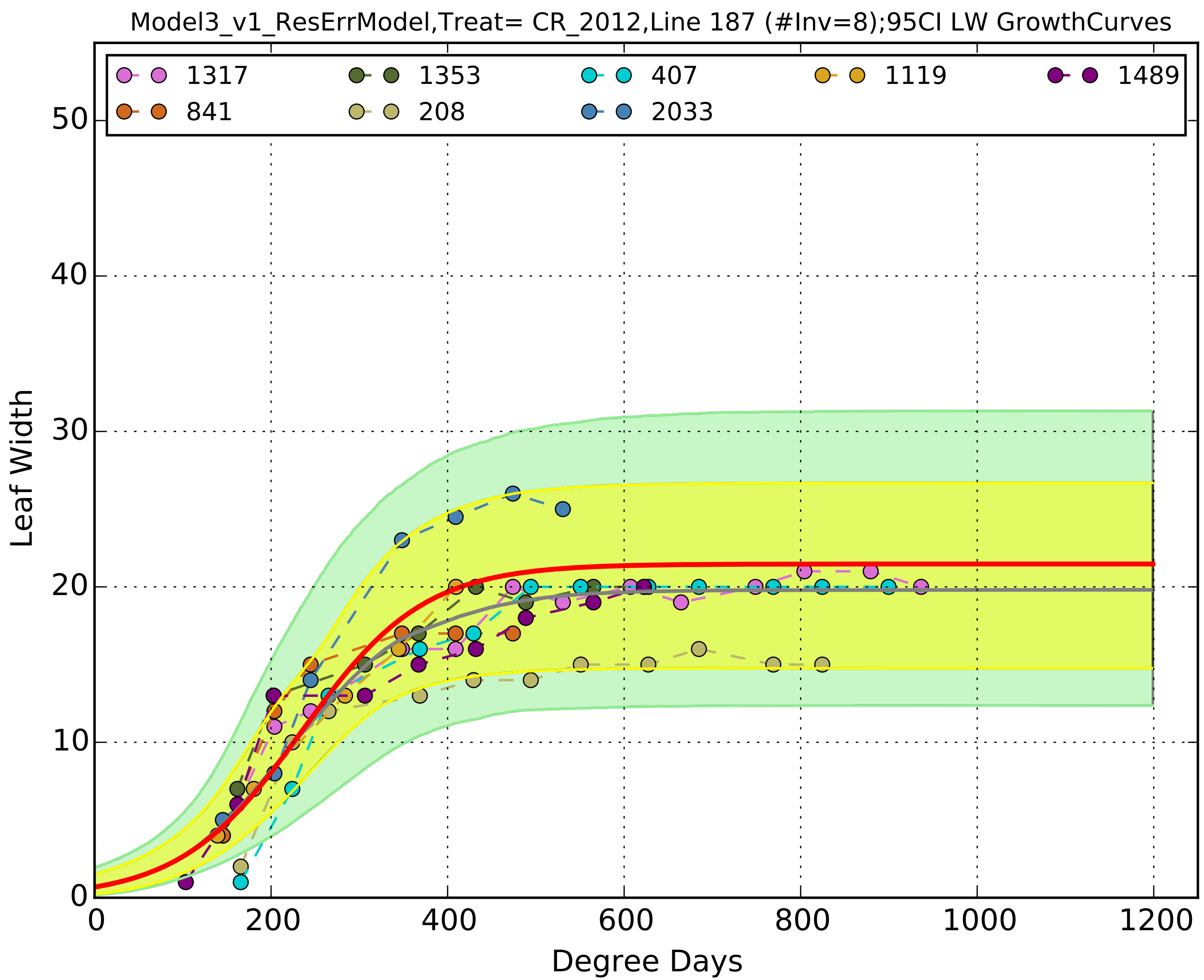
Model3_v1_ResErrModel,Treat= CR_2012,Line 187 (#Inv=8);95CI LW GrowthCurves
1317
1353
407
1119
1489
841
208
2033
Leaf Width
Degree Days
0
10
20
30
40
50
0
200
400
600
800
1000
1200

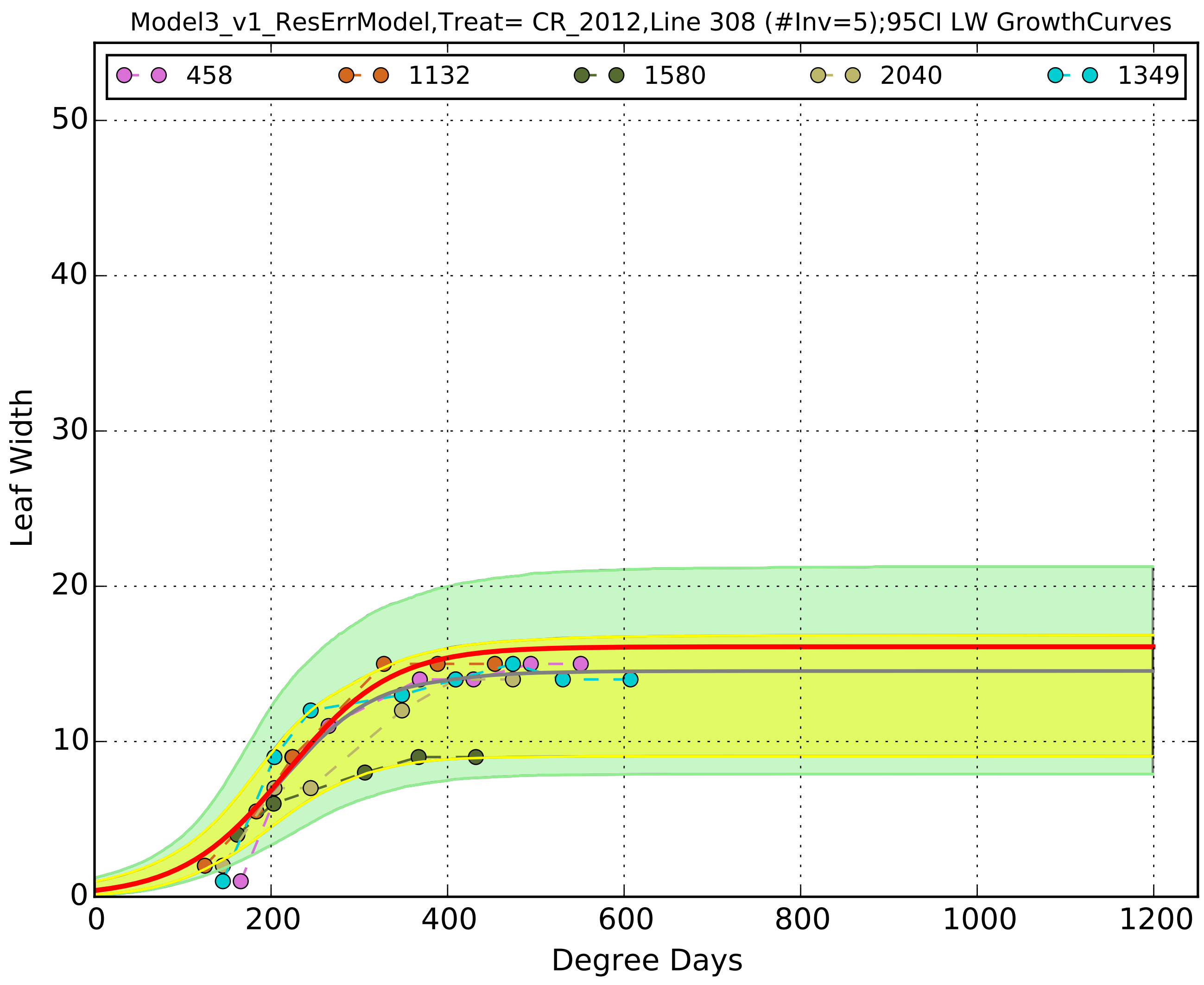

Model3_v1_ResErrModel,Treat= CR_2012,Line 308 (#Inv=5);95CI LW GrowthCurves
458
1132
1580
2040
1349
Leaf Width
Degree Days
0
10
20
30
40
50
0
200
400
600
800
1000
1200

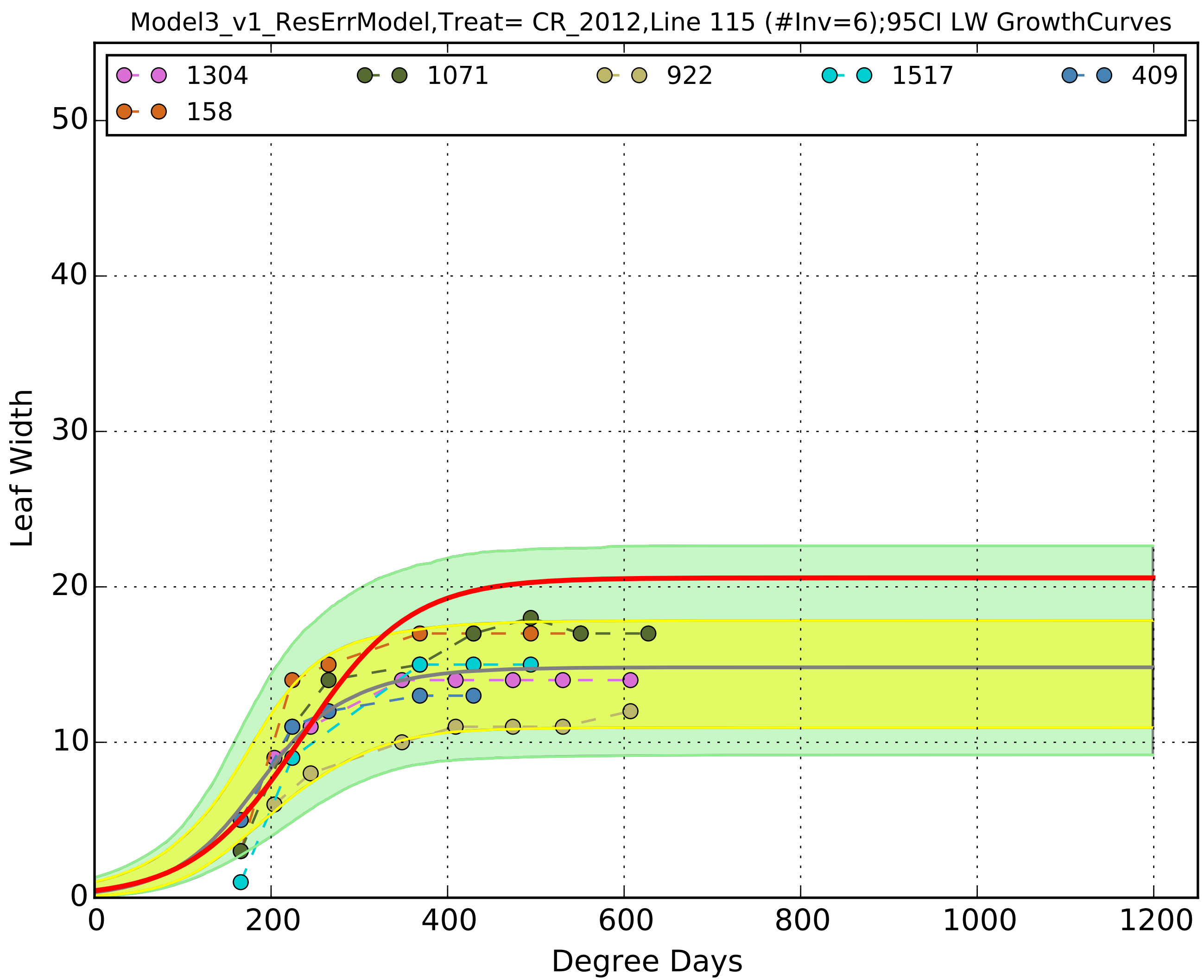
Model3_v1_ResErrModel,Treat= CR_2012,Line 115 (#Inv=6);95CI LW GrowthCurves
1304
1071
922
1517
409
158
Leaf Width
Degree Days
0
10
20
30
40
50
0
200
400
600
800
1000
1200

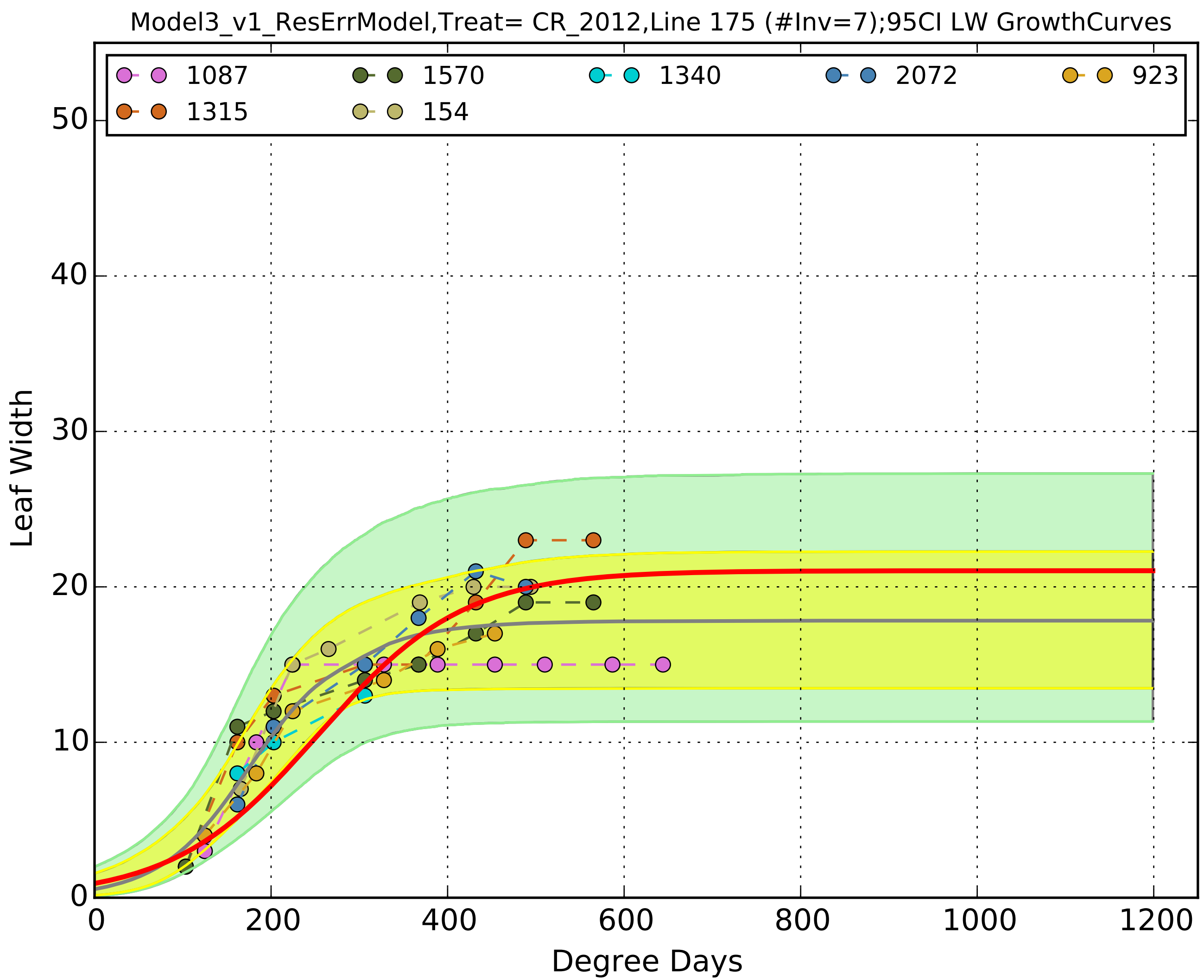

Model3_v1_ResErrModel,Treat= CR_2012,Line 175 (#Inv=7);95CI LW GrowthCurves
1087
1570
1340
2072
923
1315
154
Leaf Width
Degree Days
0
10
20
30
40
50
0
200
400
600
800
1000
1200

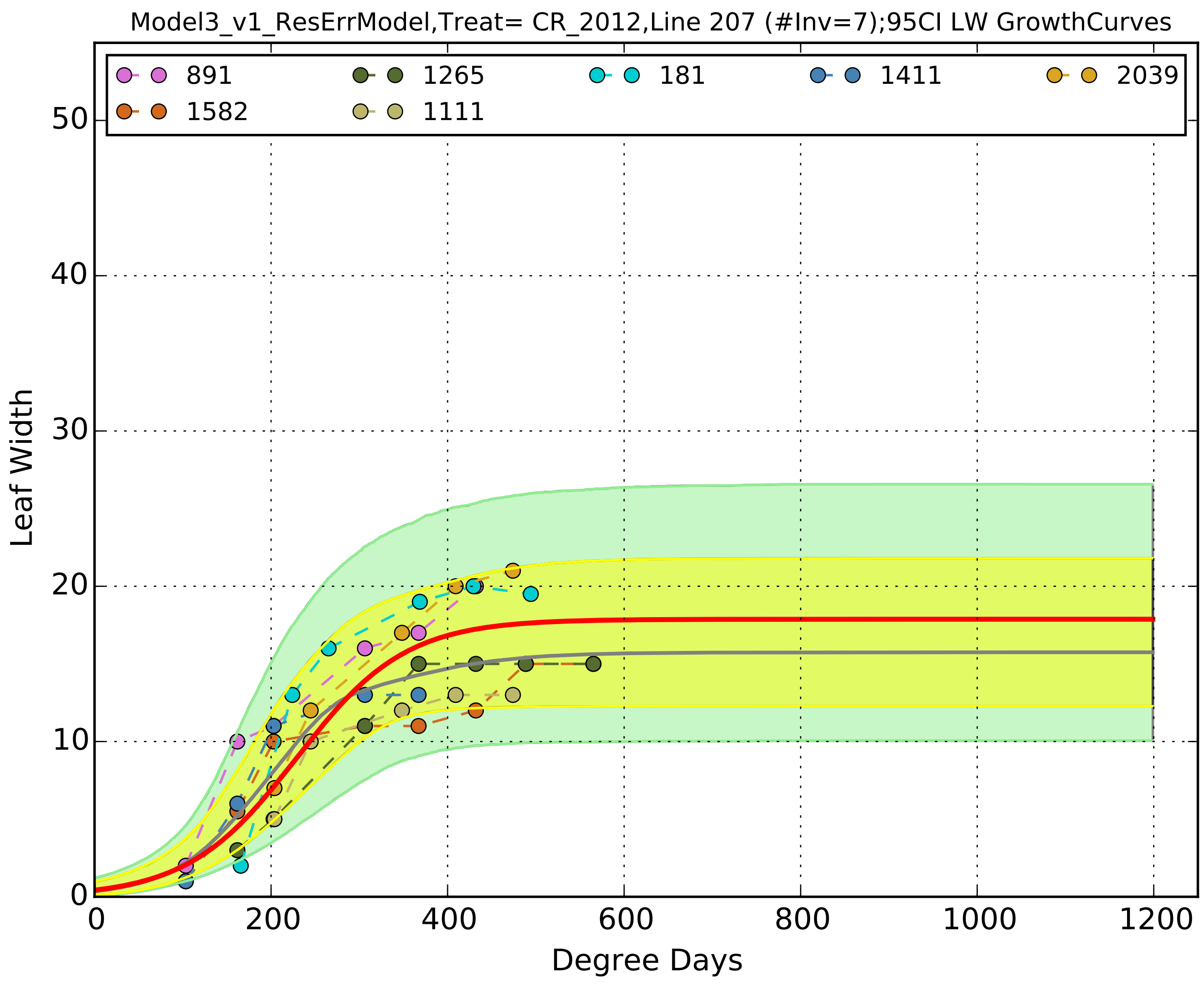

Model3_v1_ResErrModel,Treat= CR_2012,Line 207 (#Inv=7);95CI LW GrowthCurves
891
1265
181
1411
2039
1582
1111
Leaf Width
Degree Days
0
10
20
30
40
50
0
200
400
600
800
1000
1200

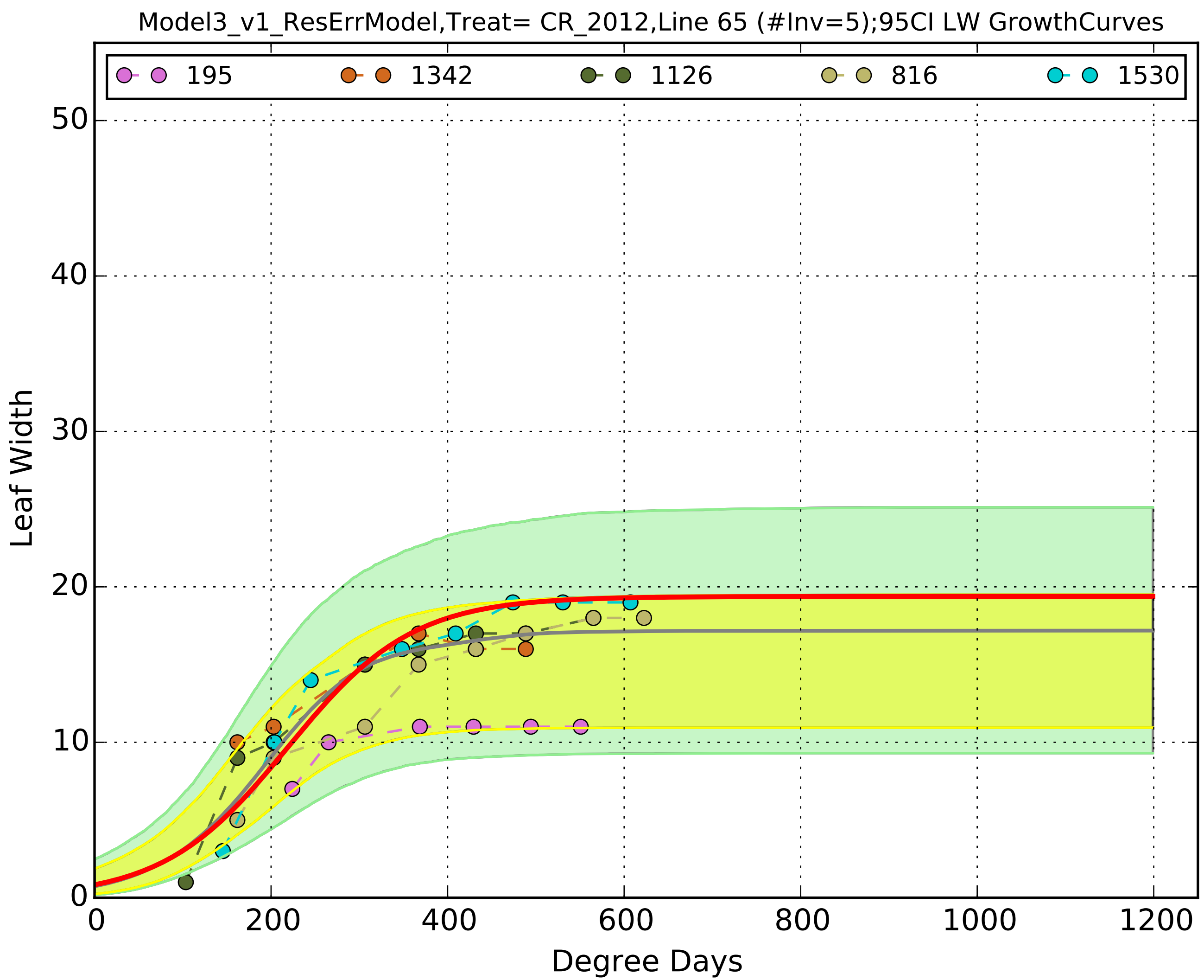

Model3_v1_ResErrModel,Treat= CR_2012,Line 65 (#Inv=5);95CI LW GrowthCurves
195
1342
1126
816
1530
Leaf Width
Degree Days
0
10
20
30
40
50
0
200
400
600
800
1000
1200

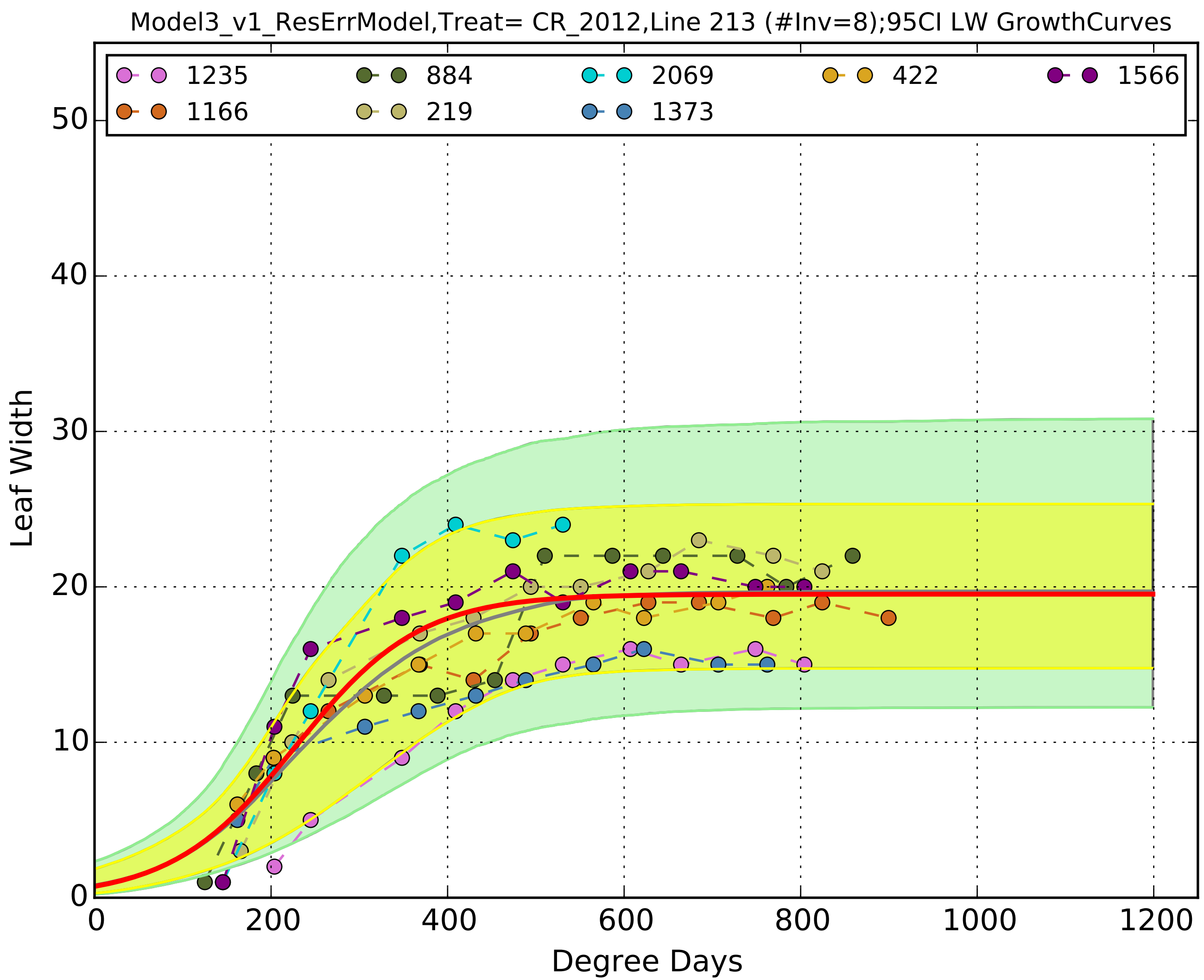

Model3_v1_ResErrModel,Treat= CR_2012,Line 213 (#Inv=8);95CI LW GrowthCurves
1235
884
2069
422
1566
1166
219
1373
Leaf Width
Degree Days
0
10
20
30
40
50
0
200
400
600
800
1000
1200

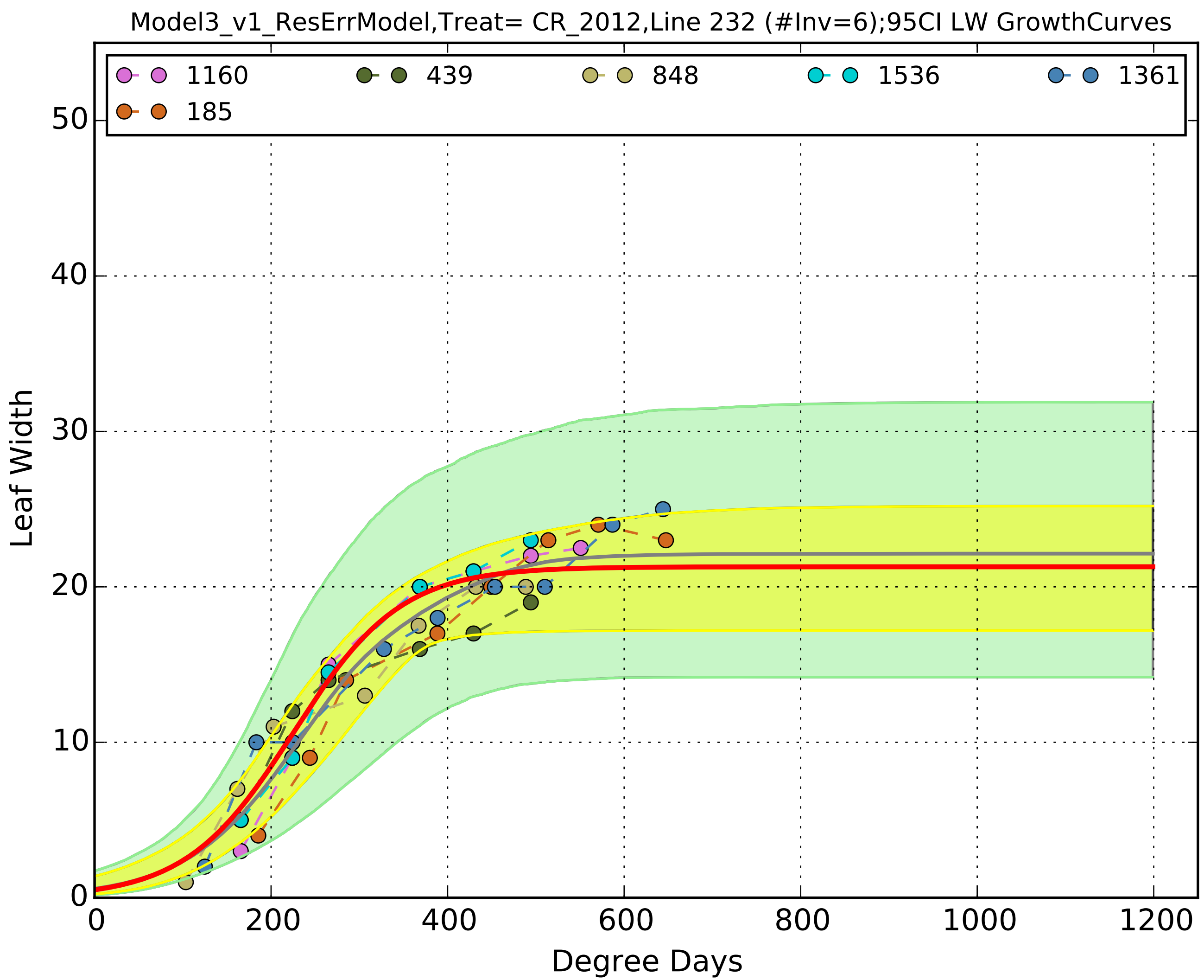

Model3_v1_ResErrModel,Treat= CR_2012,Line 232 (#Inv=6);95CI LW GrowthCurves
1160
439
848
1536
1361
185
Leaf Width
Degree Days
0
10
20
30
40
50
0
200
400
600
800
1000
1200

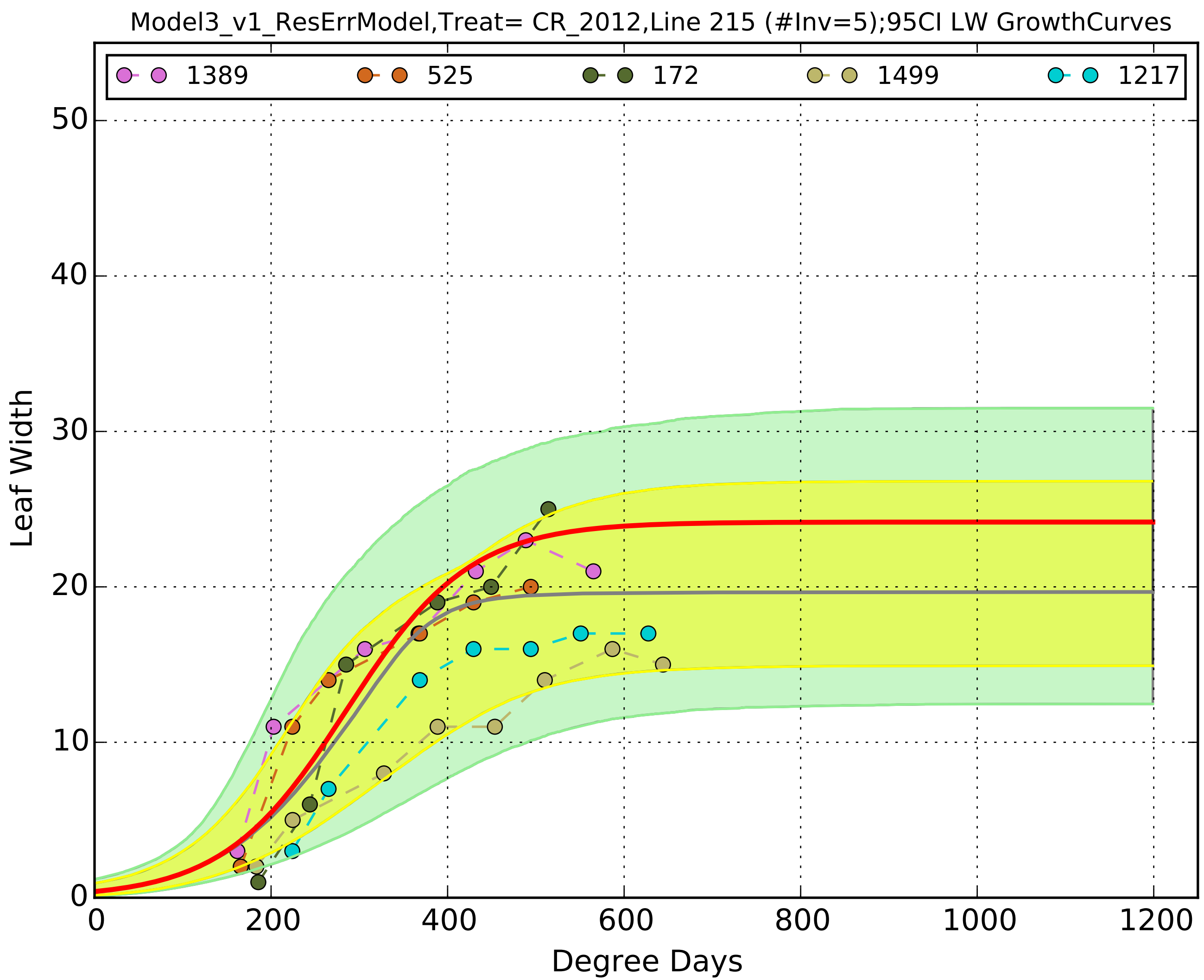

Model3_v1_ResErrModel,Treat= CR_2012,Line 215 (#Inv=5);95CI LW GrowthCurves
1389
525
172
1499
1217
Leaf Width
Degree Days
0
10
20
30
40
50
0
200
400
600
800
1000
1200

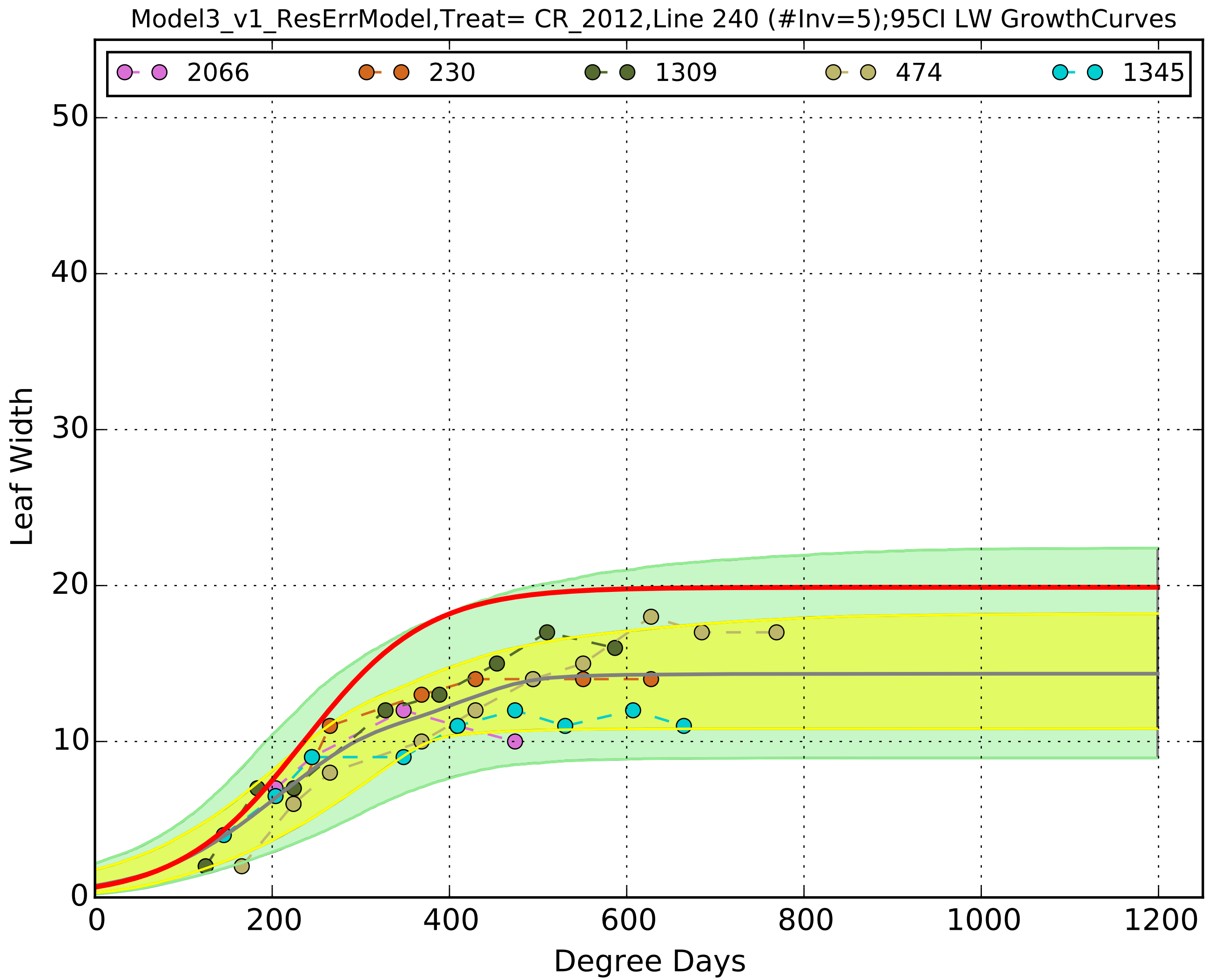

Model3_v1_ResErrModel,Treat= CR_2012,Line 240 (#Inv=5);95CI LW GrowthCurves
2066
230
1309
474
1345
Leaf Width
Degree Days
0
10
20
30
40
50
0
200
400
600
800
1000
1200

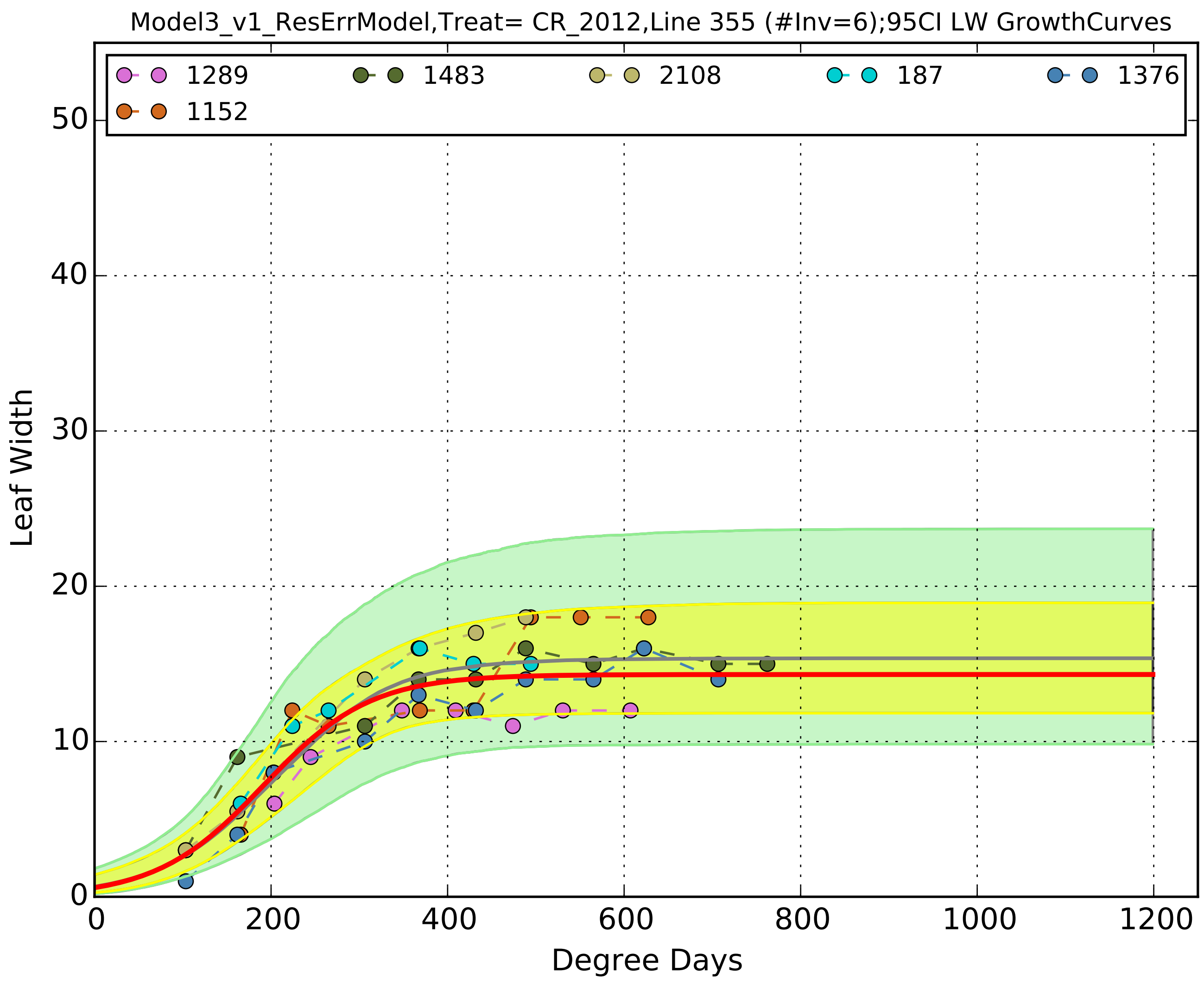

Model3_v1_ResErrModel,Treat= CR_2012,Line 355 (#Inv=6);95CI LW GrowthCurves
1289
1483
2108
187
1376
1152
Leaf Width
Degree Days
0
10
20
30
40
50
0
200
400
600
800
1000
1200

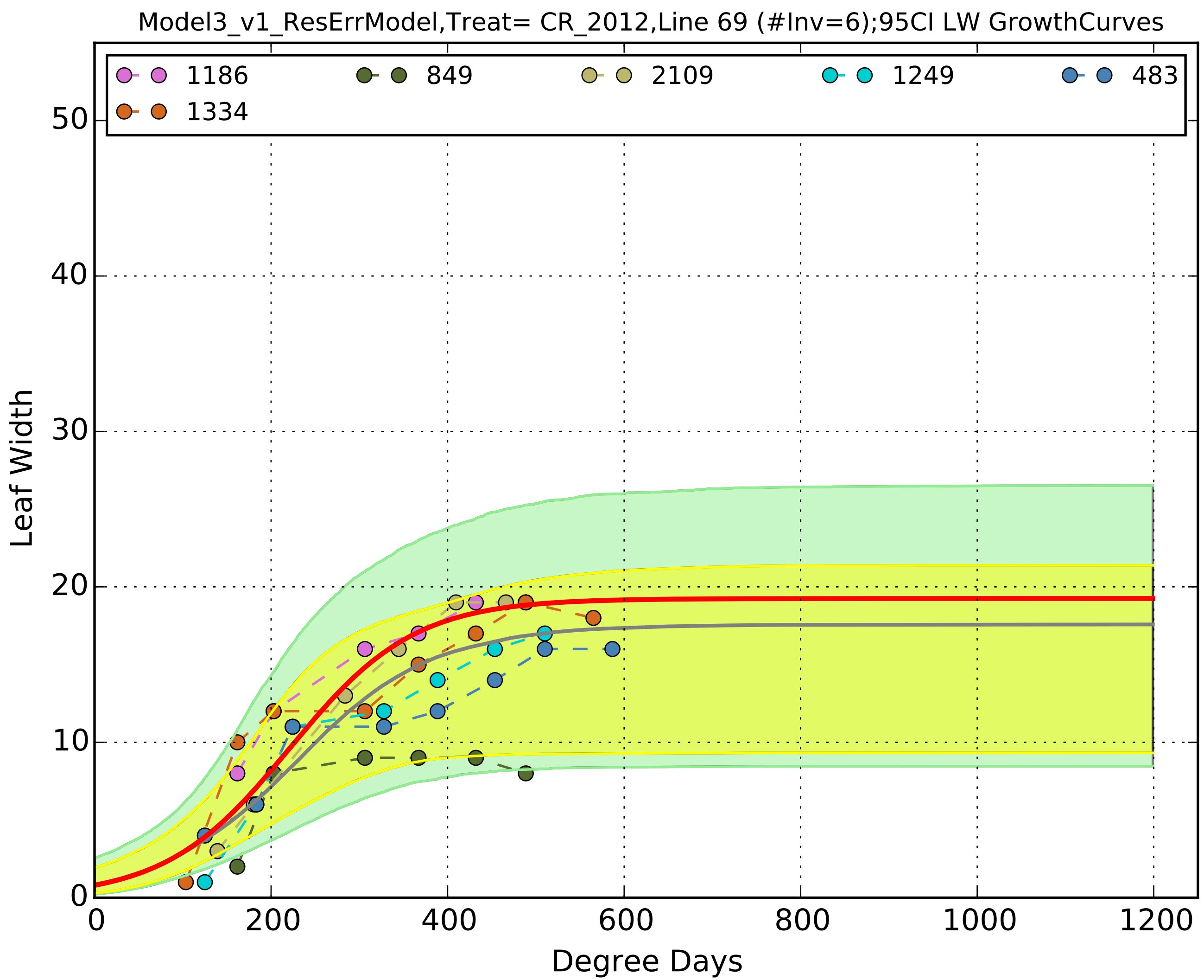
Model3_v1_ResErrModel,Treat= CR_2012,Line 69 (#Inv=6);95CI LW GrowthCurves
1186
849
2109
1249
483
1334
0
10
20
30
40
50
Leaf Width
0
200
400
600
800
1000
1200
Degree Days

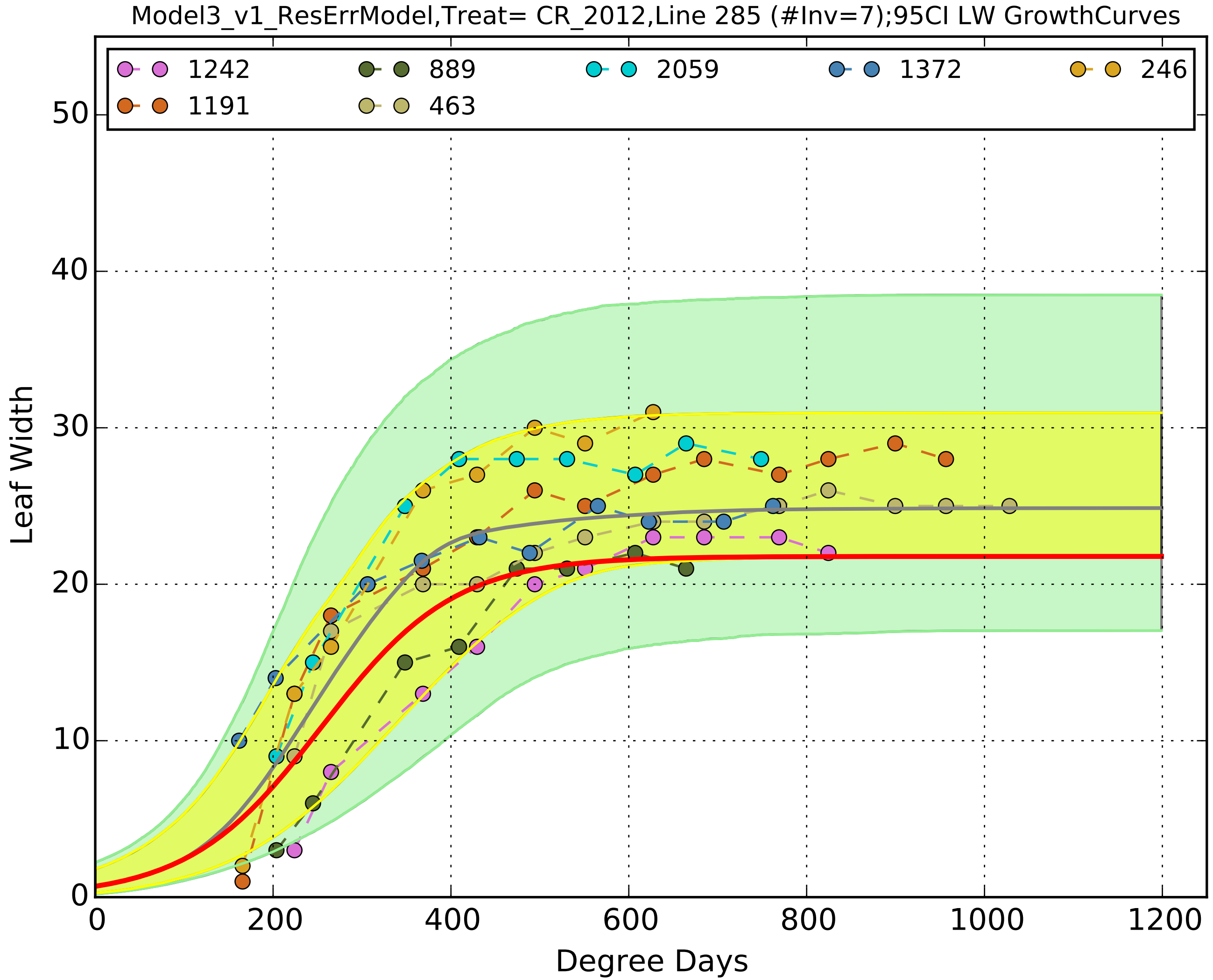

Model3_v1_ResErrModel,Treat= CR_2012,Line 285 (#Inv=7);95CI LW GrowthCurves
1242
889
2059
1372
246
1191
463
Leaf Width
Degree Days
0
10
20
30
40
50
0
200
400
600
800
1000
1200

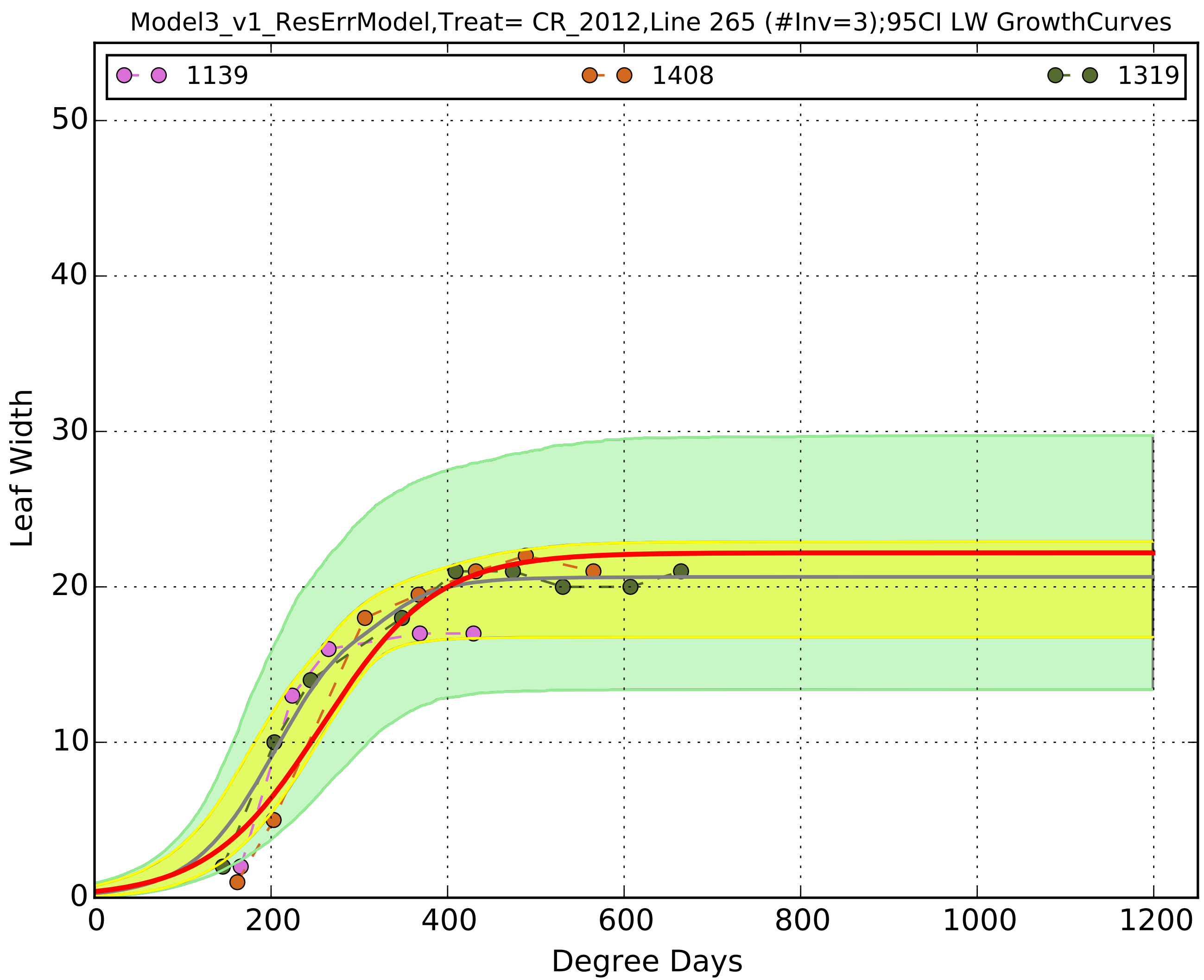

Model3_v1_ResErrModel,Treat= CR_2012,Line 265 (#Inv=3);95CI LW GrowthCurves
1139
1408
1319
Leaf Width
Degree Days
0
10
20
30
40
50
0
200
400
600
800
1000
1200

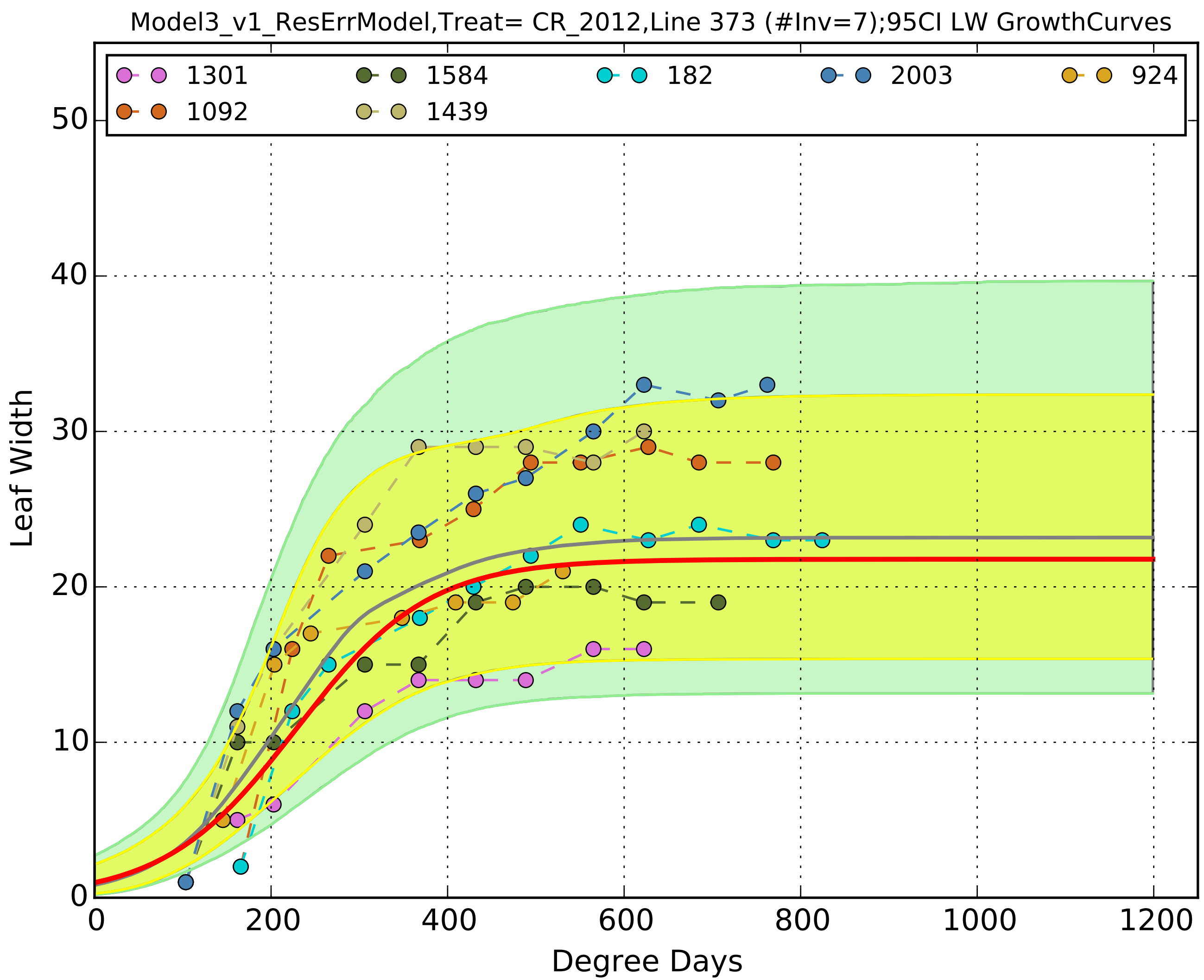
Model3_v1_ResErrModel,Treat= CR_2012,Line 373 (#Inv=7);95CI LW GrowthCurves
1301
1584
182
2003
924
1092
1439
Leaf Width
Degree Days
0
10
20
30
40
50
0
200
400
600
800
1000
1200

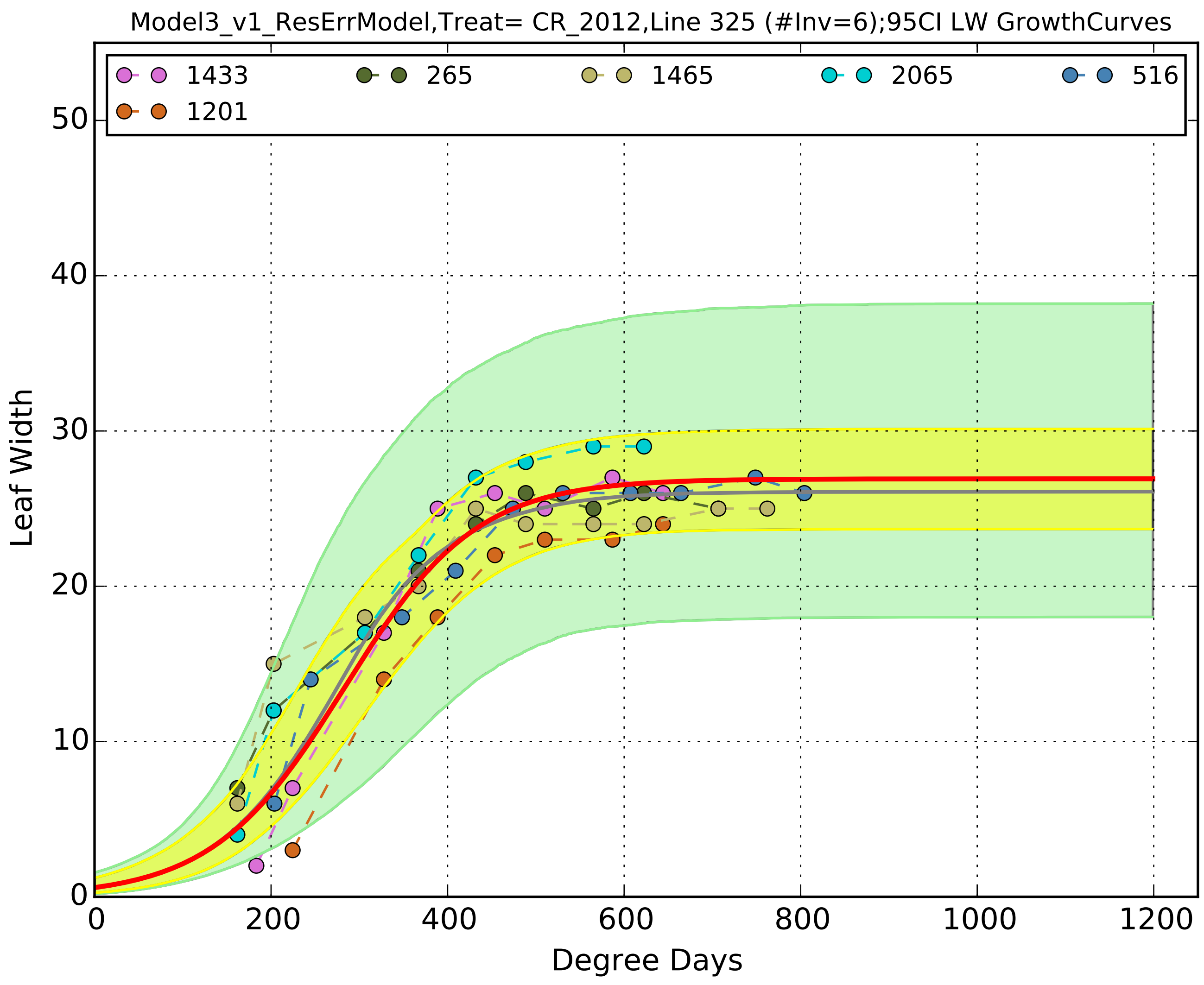

Model3_v1_ResErrModel,Treat= CR_2012,Line 325 (#Inv=6);95CI LW GrowthCurves
1433
265
1465
2065
516
1201
Leaf Width
Degree Days
0
10
20
30
40
50
0
200
400
600
800
1000
1200

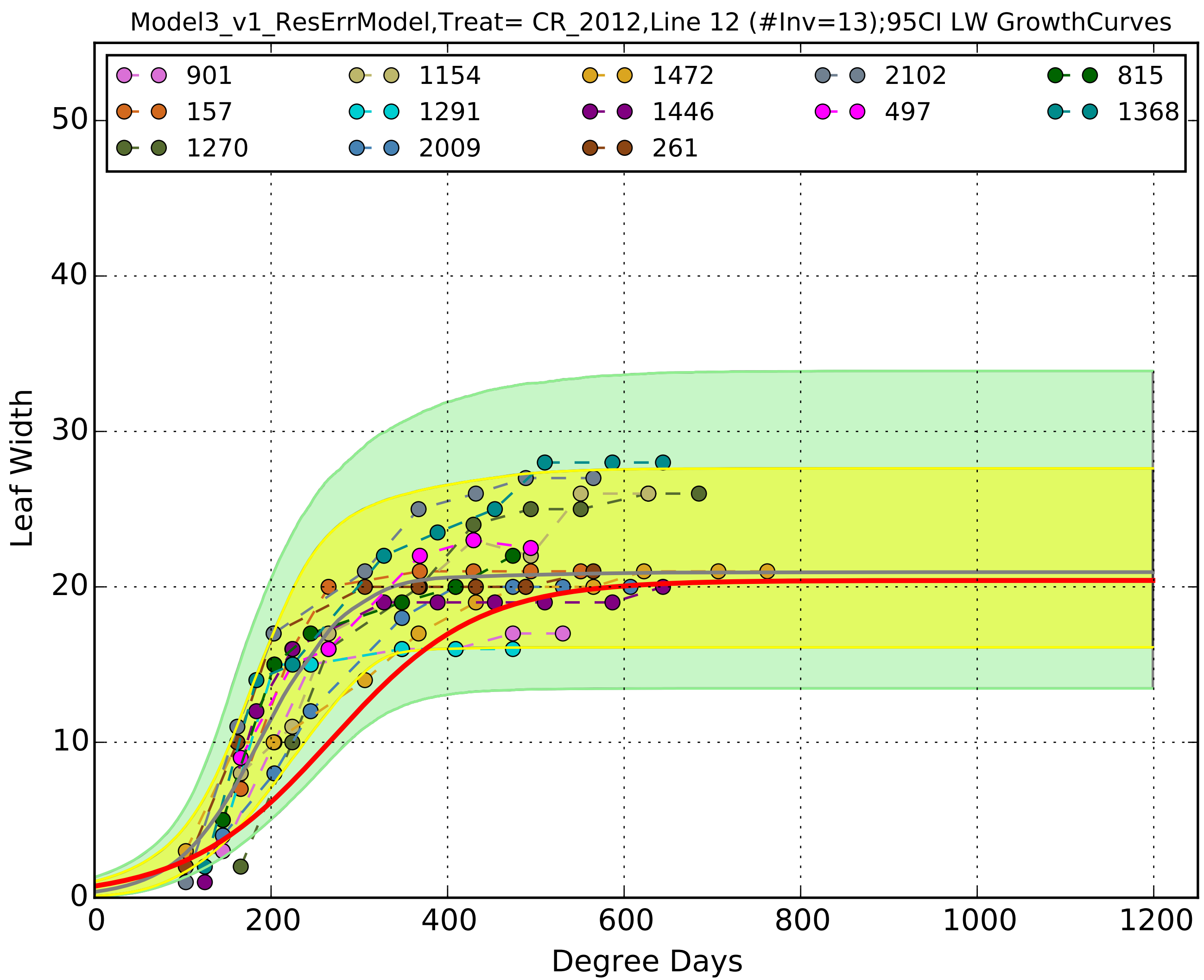

Model3_v1_ResErrModel,Treat= CR_2012,Line 12 (#Inv=13);95CI LW GrowthCurves
901
157
1270
1154
1291
2009
1472
1446
261
2102
497
815
1368
Leaf Width
Degree Days
0
10
20
30
40
50
200
400
600
800
1000
1200

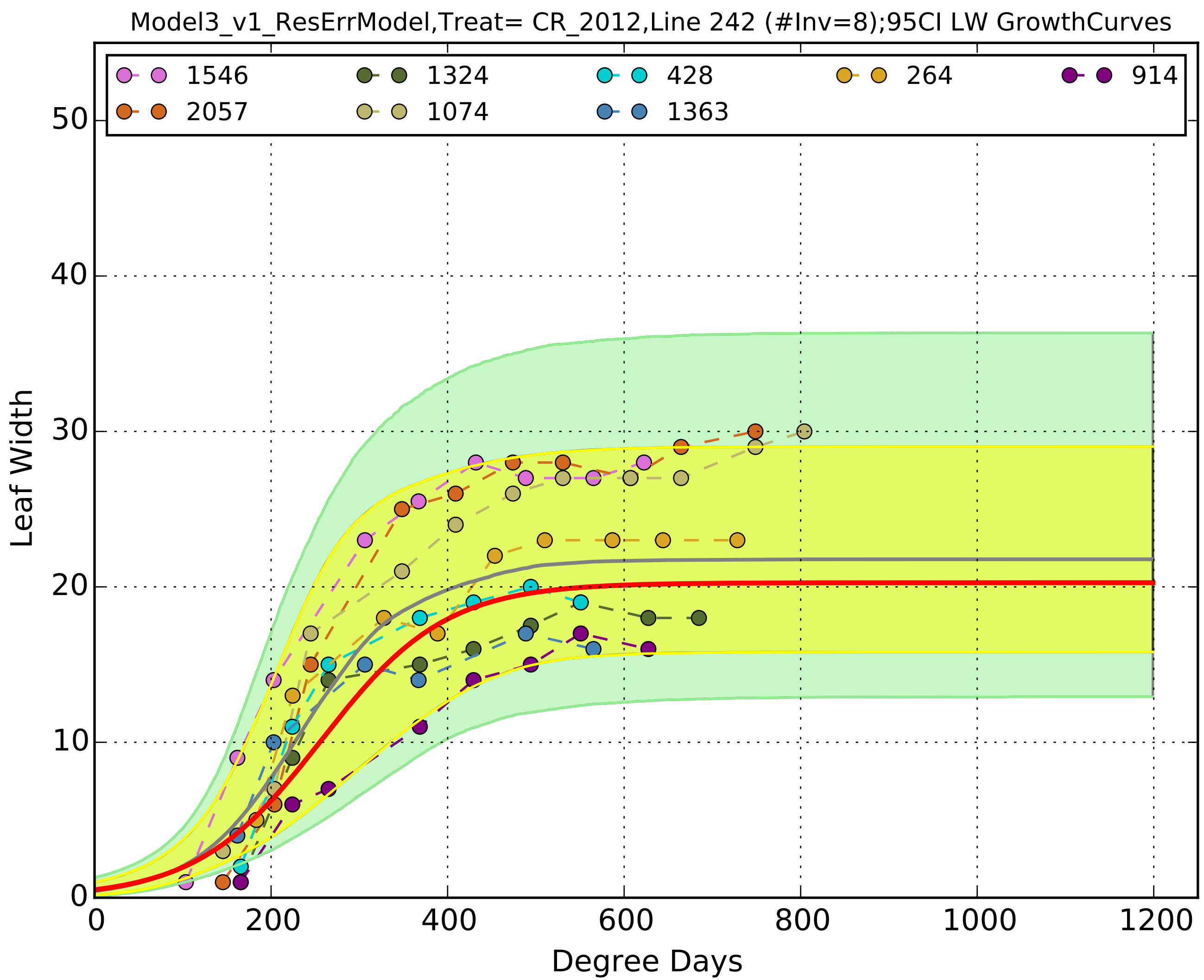
Model3_v1_ResErrModel,Treat= CR_2012,Line 242 (#Inv=8);95CI LW GrowthCurves
1546
2057
1324
1074
428
1363
264
914
Leaf Width
Degree Days
0
10
20
30
40
50
0
200
400
600
800
1000
1200

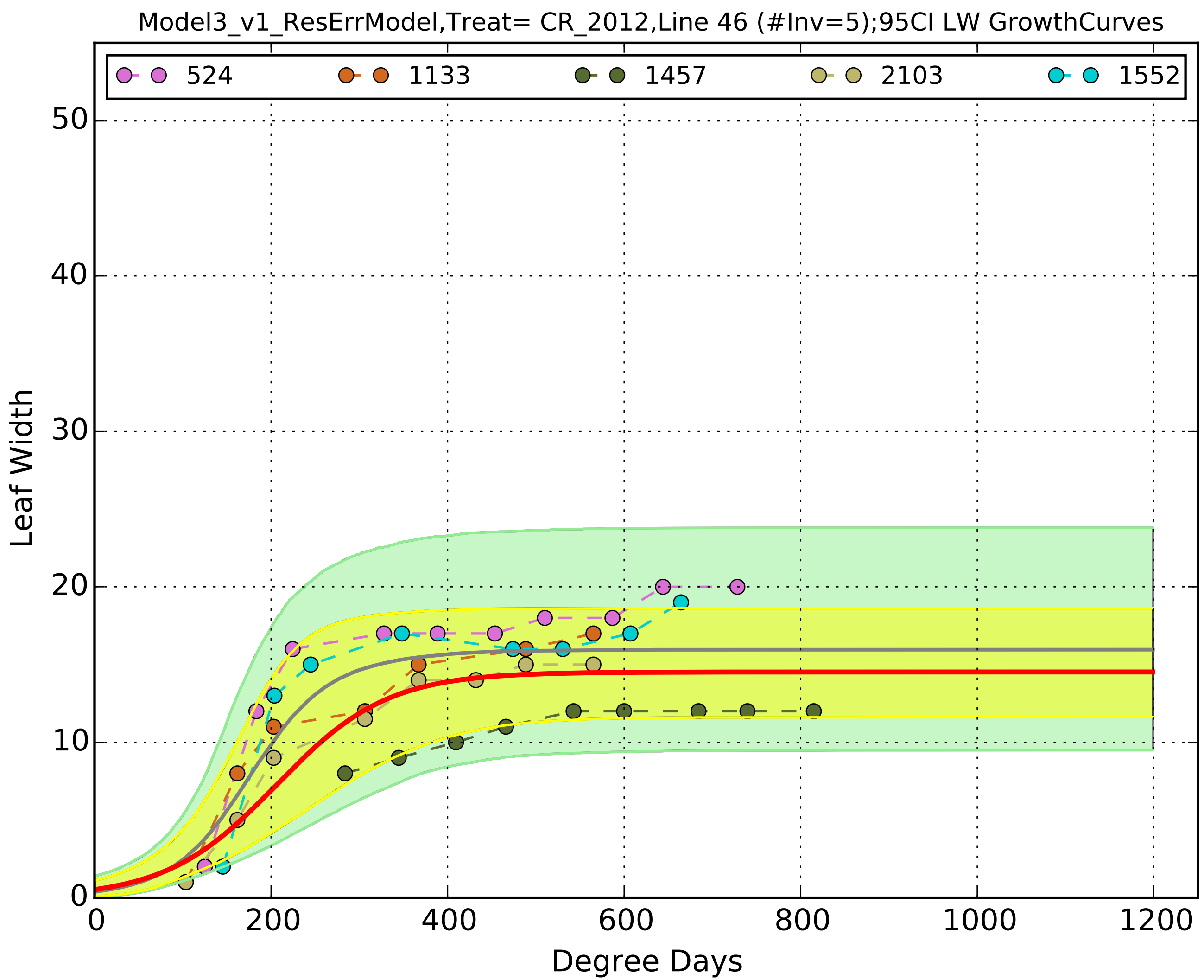

Model3_v1_ResErrModel,Treat= CR_2012,Line 46 (#Inv=5);95CI LW GrowthCurves
524
1133
1457
2103
1552
Leaf Width
Degree Days
0
10
20
30
40
50
0
200
400
600
800
1000
1200

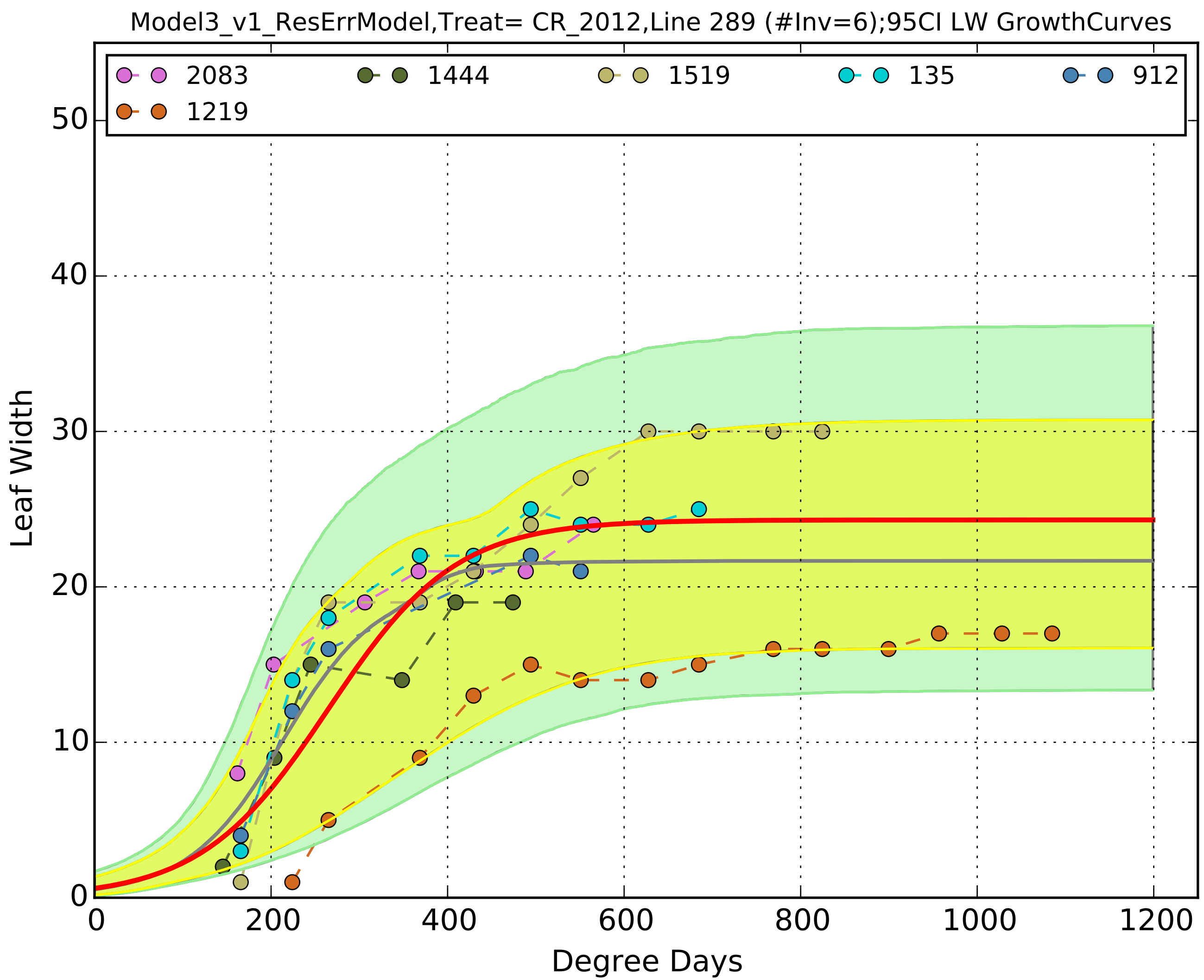

Model3_v1_ResErrModel,Treat= CR_2012,Line 289 (#Inv=6);95CI LW GrowthCurves
2083
1444
1519
135
912
1219
Leaf Width
Degree Days
0
10
20
30
40
50
0
200
400
600
800
1000
1200

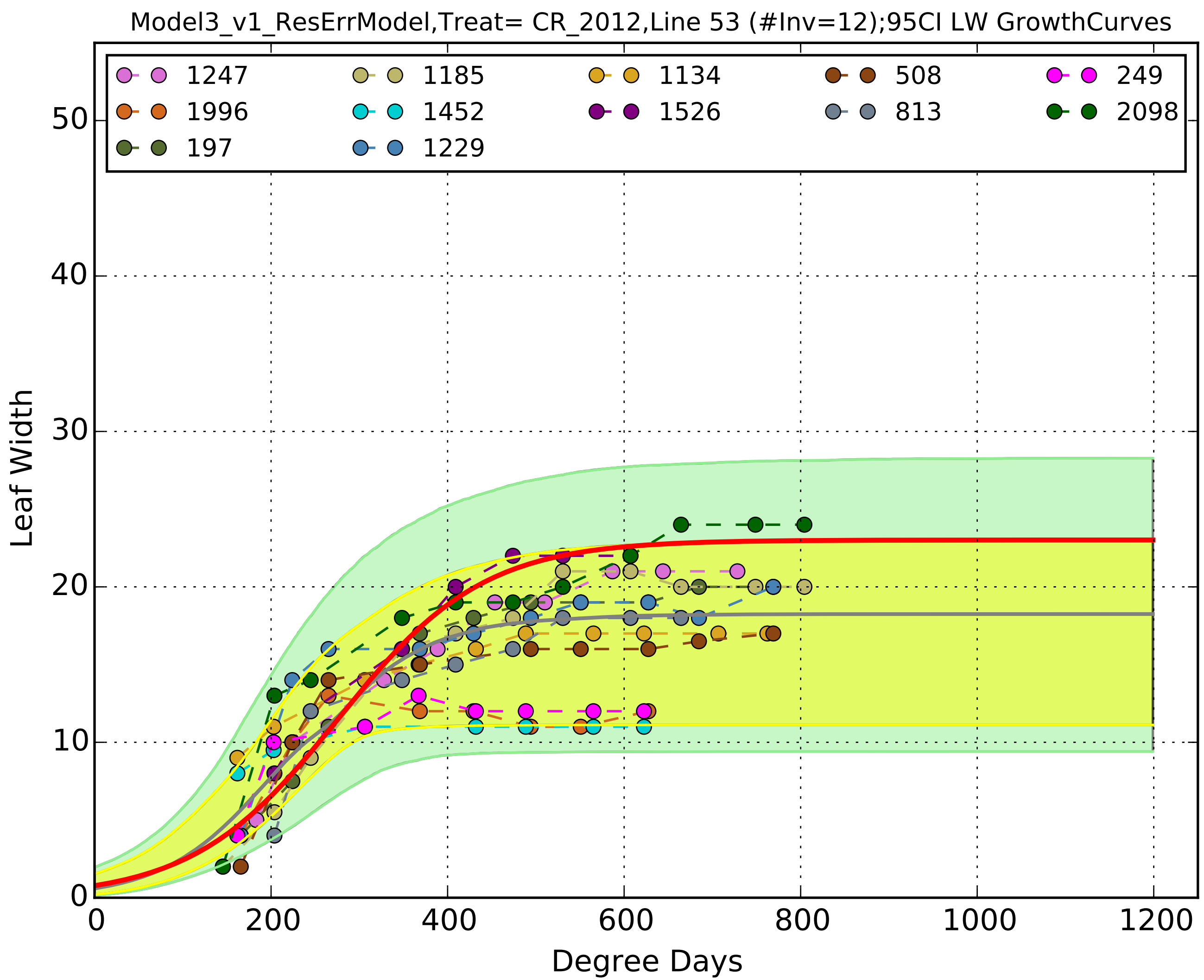
Model3_v1_ResErrModel,Treat= CR_2012,Line 53 (#Inv=12);95CI LW GrowthCurves
1247
1185
1134
508
249
1996
1452
1526
813
2098
197
1229
Leaf Width
Degree Days
0
10
20
30
40
50
0
200
400
600
800
1000
1200

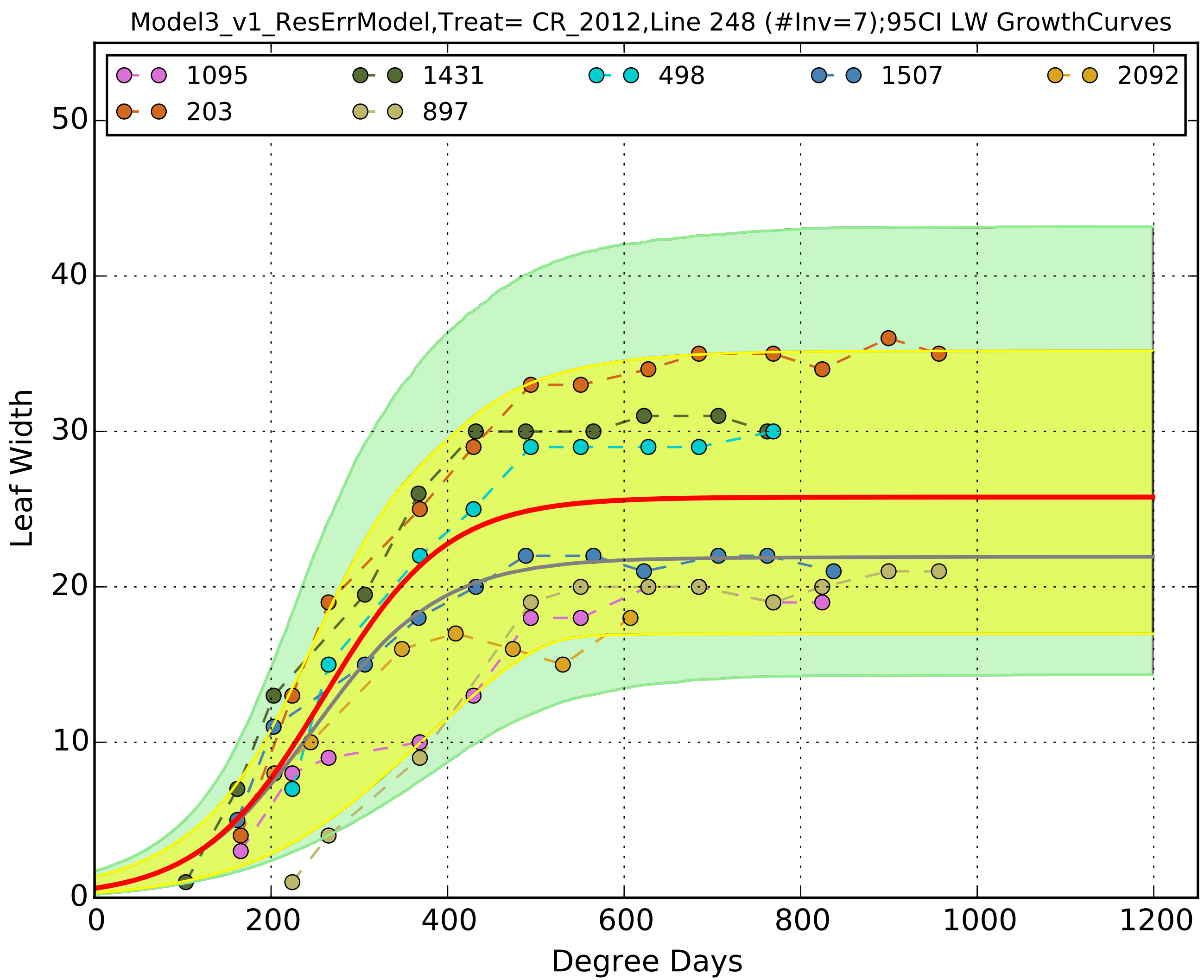

Model3_v1_ResErrModel,Treat= CR_2012,Line 248 (#Inv=7);95CI LW GrowthCurves
1095
1431
498
1507
2092
203
897
Leaf Width
Degree Days
0
10
20
30
40
50
0
200
400
600
800
1000
1200

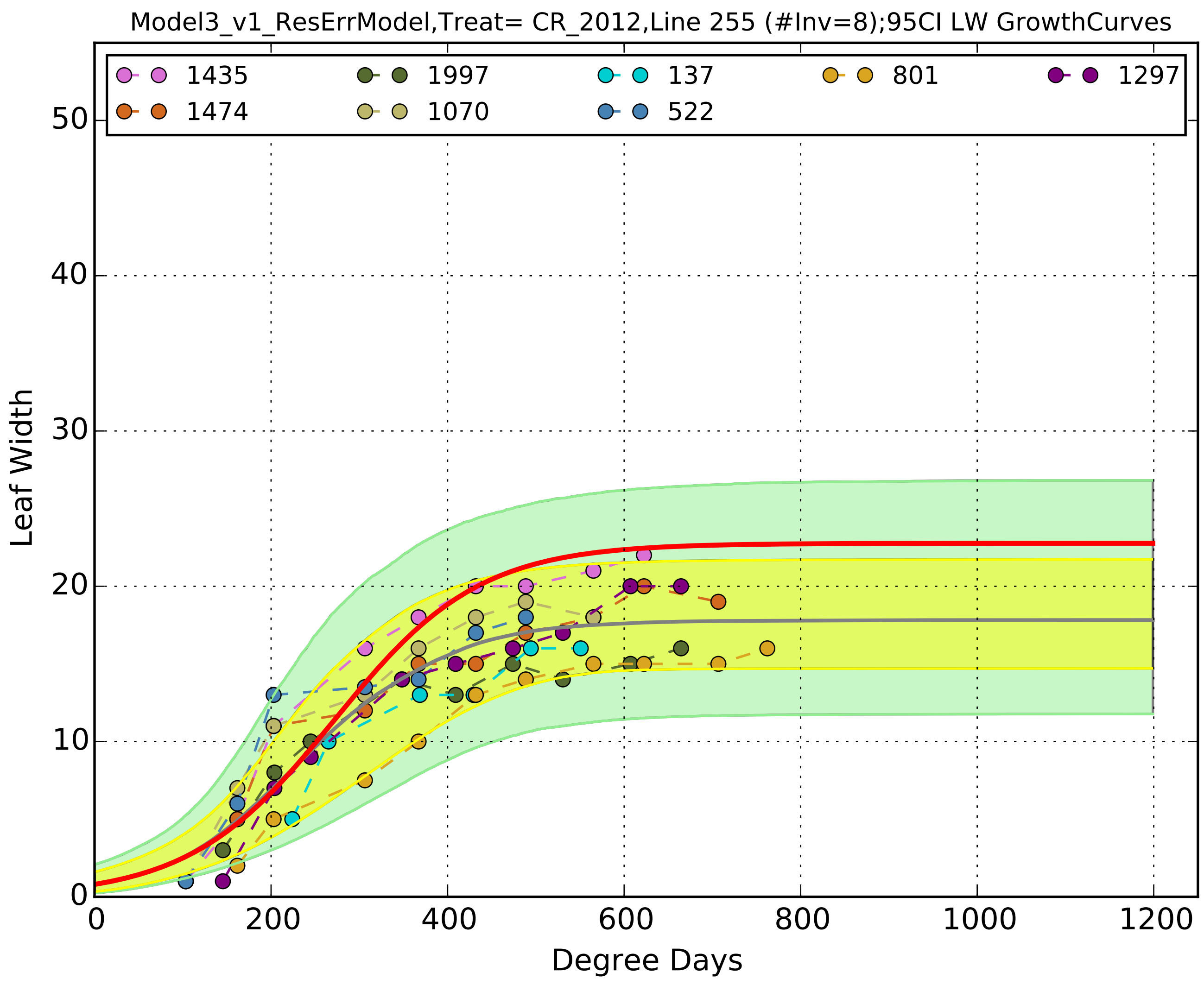
Model3_v1_ResErrModel,Treat= CR_2012,Line 255 (#Inv=8);95CI LW GrowthCurves
1435
1997
137
801
1297
1474
1070
522
Leaf Width
Degree Days
0
10
20
30
40
50
0
200
400
600
800
1000
1200

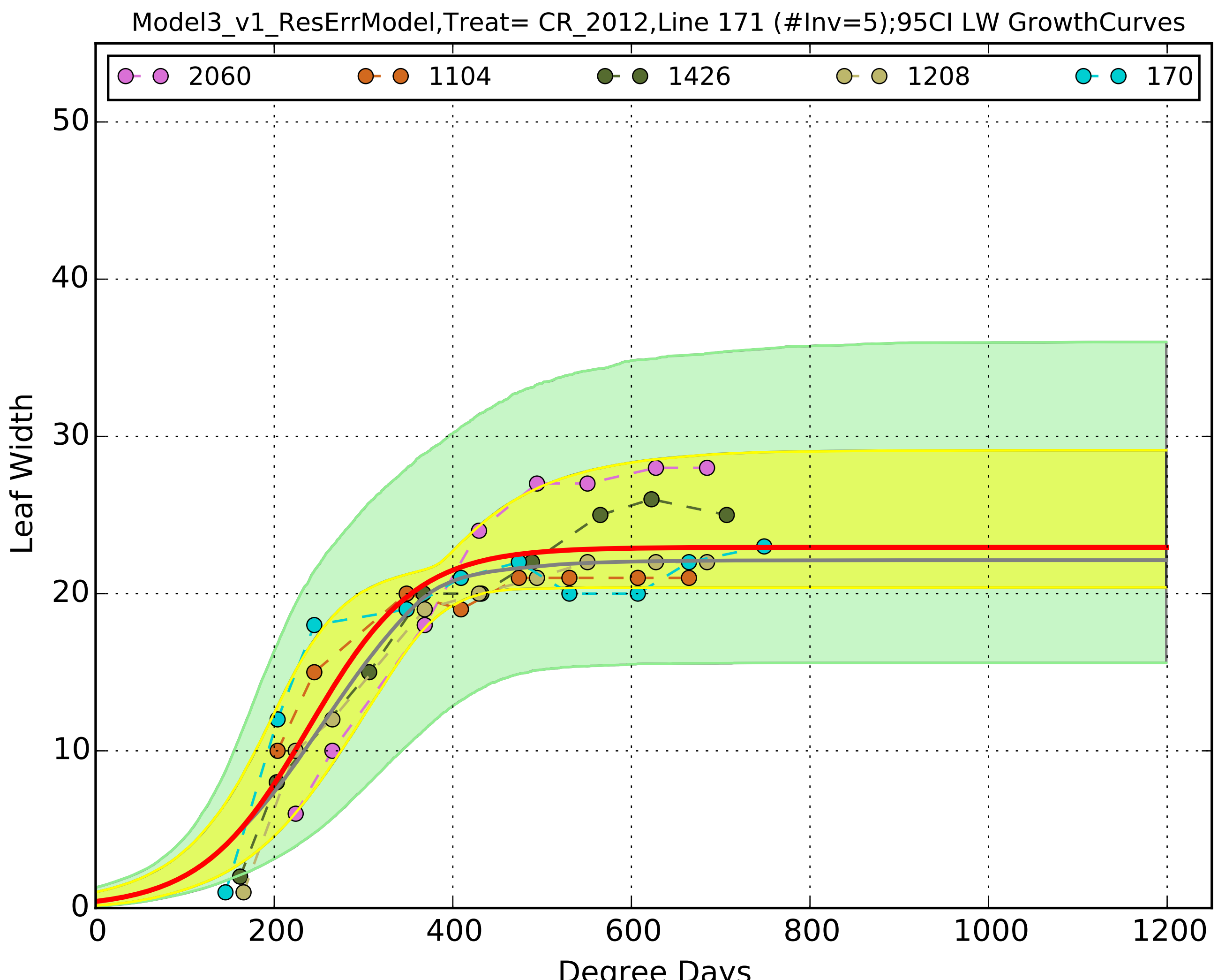

Model3_v1_ResErrModel,Treat= CR_2012,Line 171 (#Inv=5);95CI LW GrowthCurves
2060
1104
1426
1208
170
Leaf Width
Degree Days
0
10
20
30
40
50
0
200
400
600
800
1000
1200

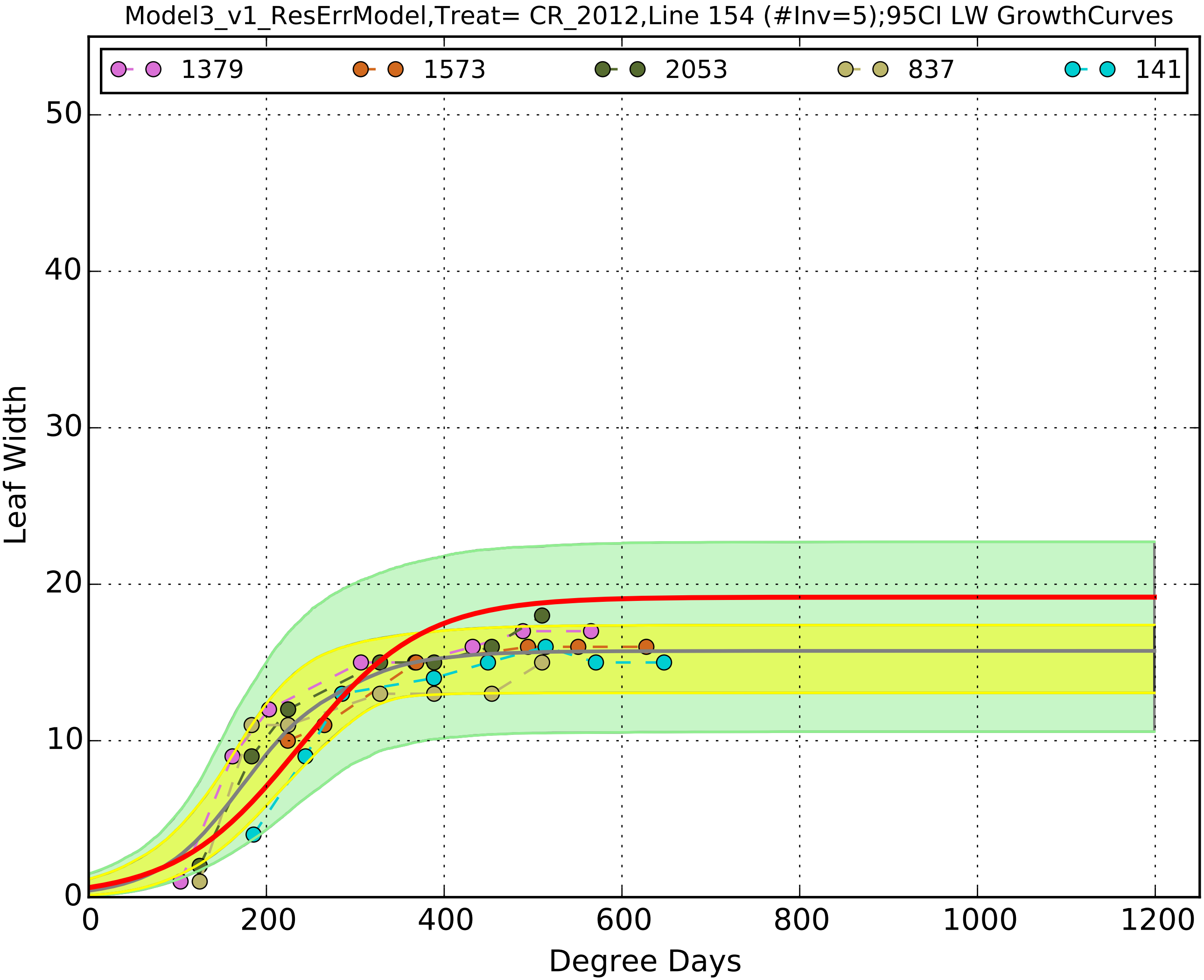

Model3_v1_ResErrModel,Treat= CR_2012,Line 154 (#Inv=5);95CI LW GrowthCurves
1379
1573
2053
837
141
0
10
20
30
40
50
0
200
400
600
800
1000
1200
Leaf Width
Degree Days

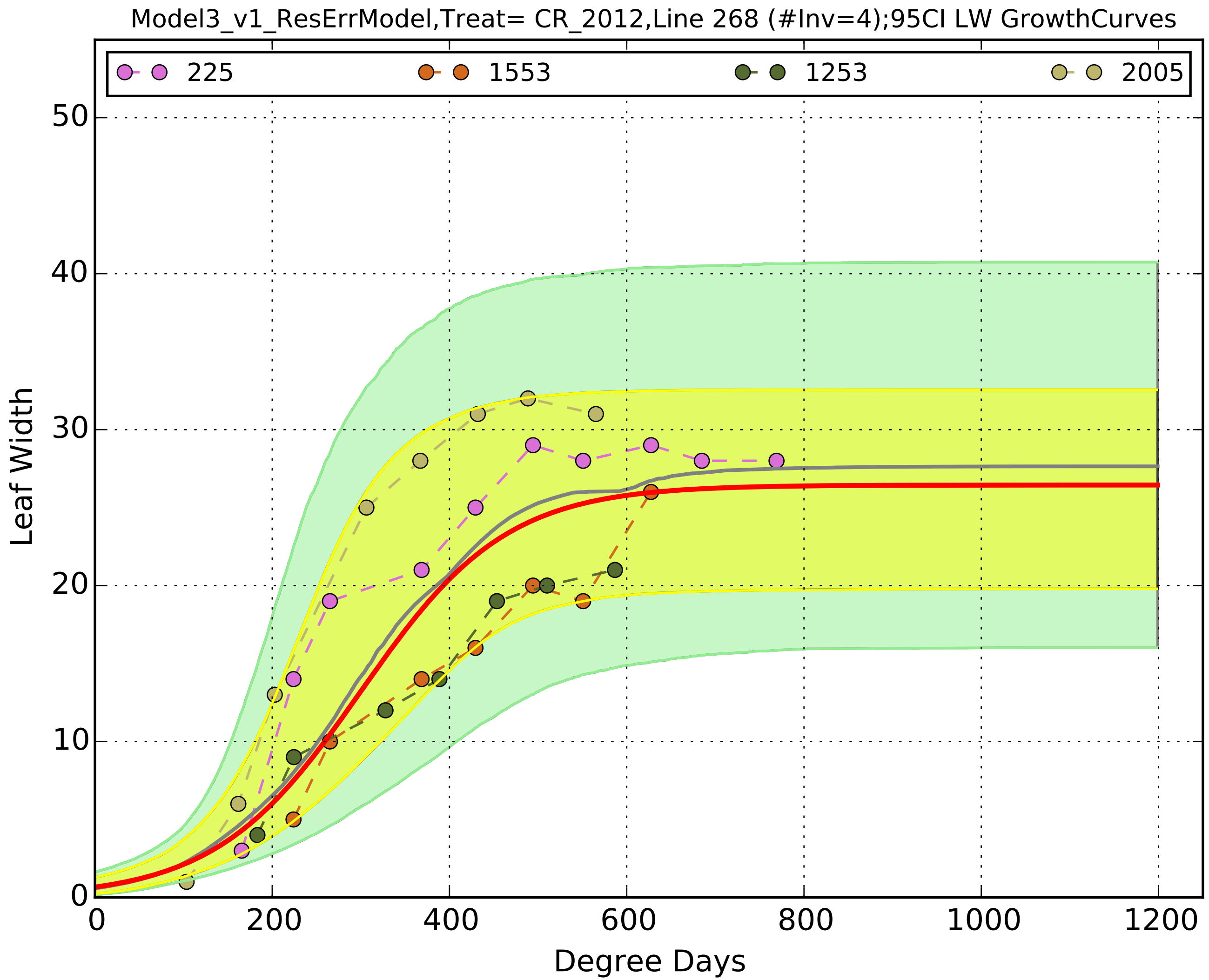

Model3_v1_ResErrModel,Treat= CR_2012,Line 268 (#Inv=4);95CI LW GrowthCurves
225
1553
1253
2005
Leaf Width
Degree Days
0
10
20
30
40
50
0
200
400
600
800
1000
1200

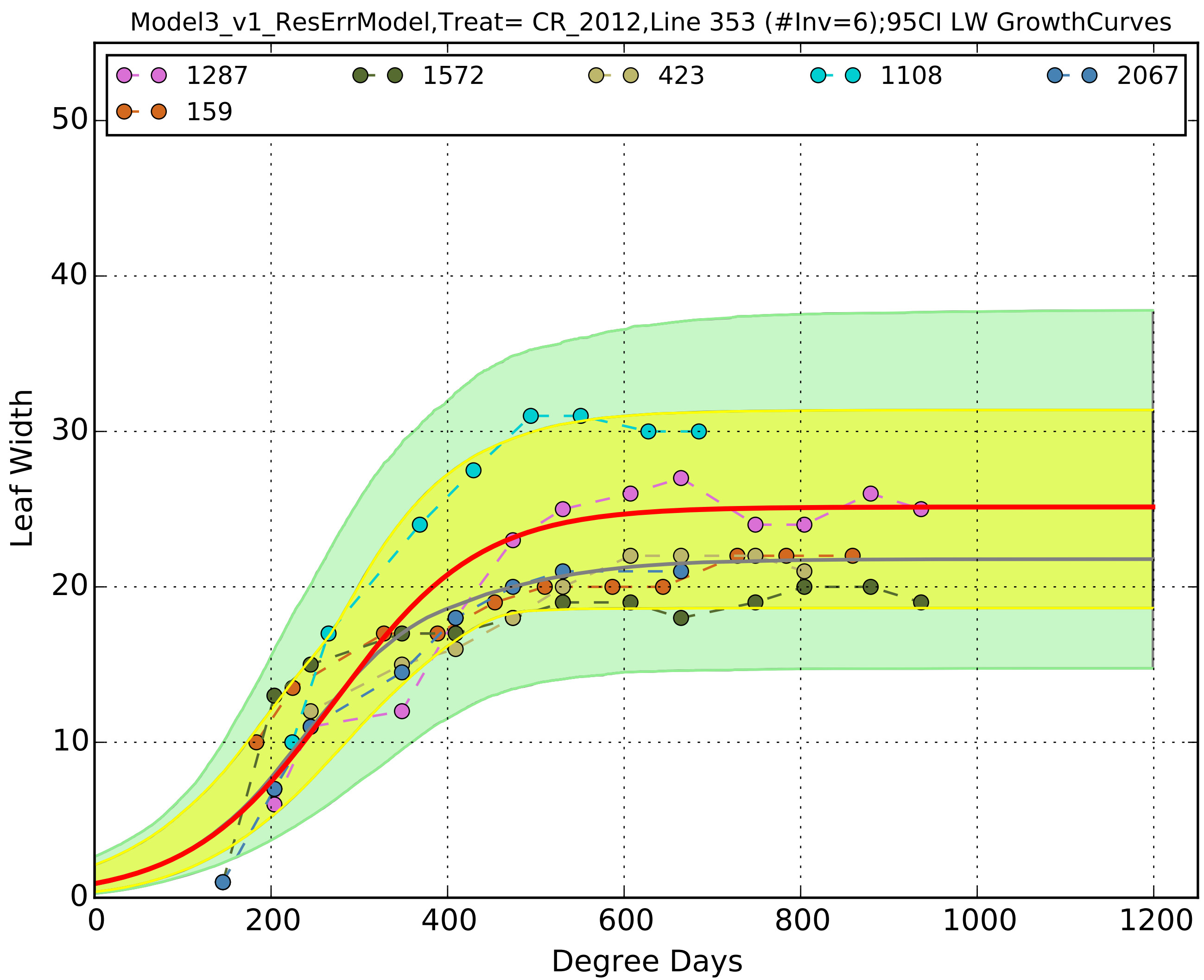

Model3_v1_ResErrModel,Treat= CR_2012,Line 353 (#Inv=6);95CI LW GrowthCurves
1287
1572
423
1108
2067
159
Leaf Width
Degree Days
0
10
20
30
40
50
0
200
400
600
800
1000
1200

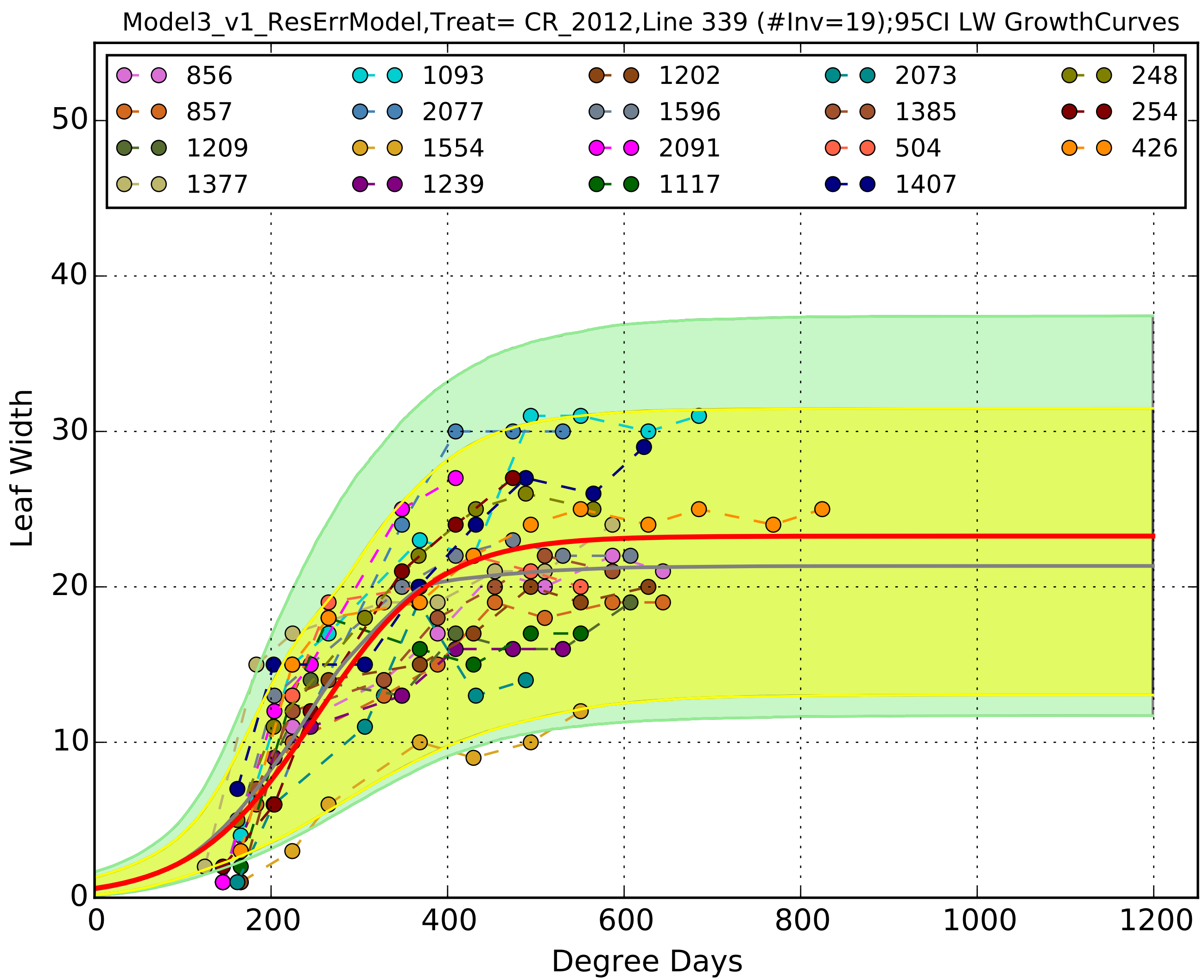
Model3_v1_ResErrModel,Treat= CR_2012,Line 339 (#Inv=19);95CI LW GrowthCurves
856
857
1209
1377
1093
2077
1554
1239
1202
1596
2091
1117
2073
1385
504
1407
248
254
426
Leaf Width
Degree Days
0
10
20
30
40
50
0
200
400
600
800
1000
1200

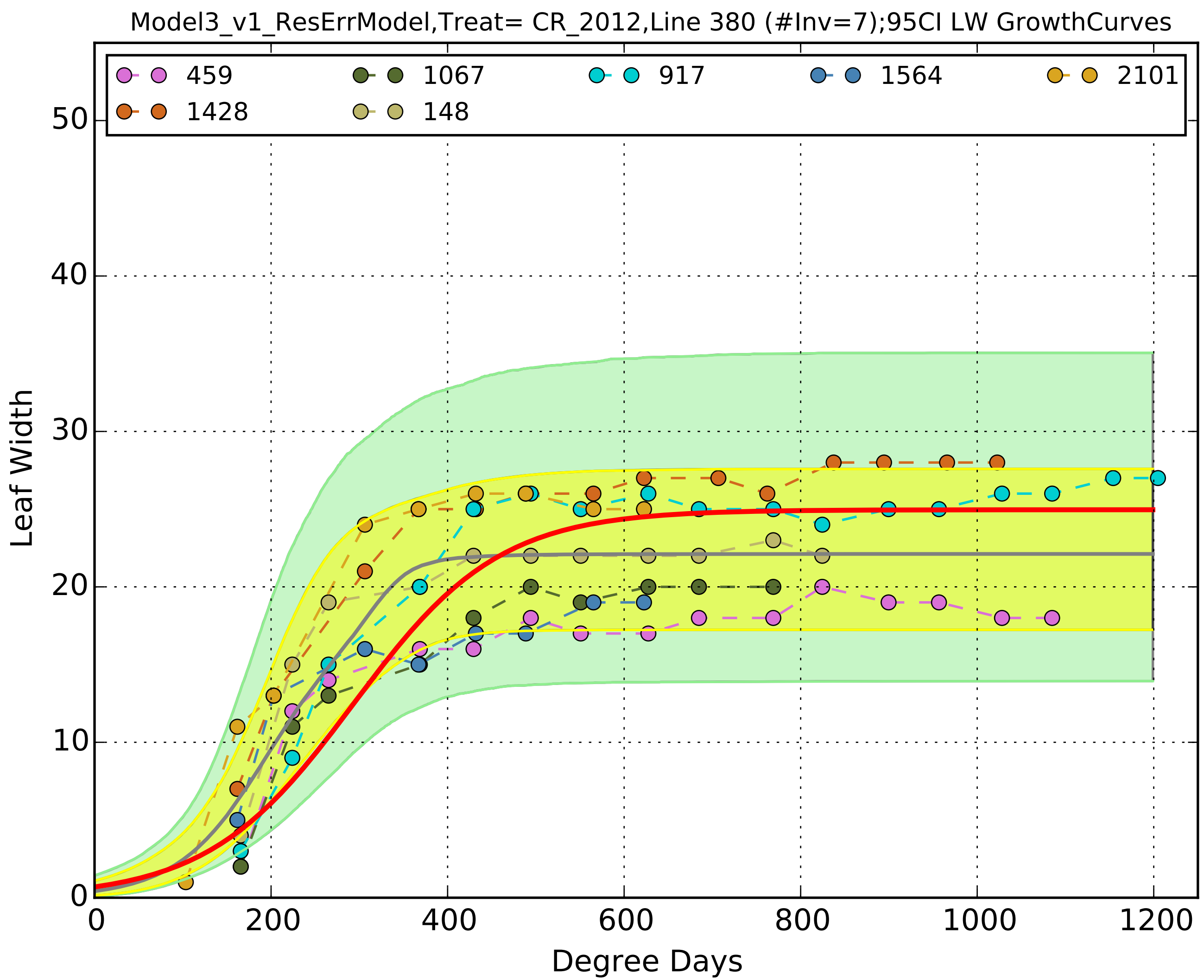

Model3_v1_ResErrModel,Treat= CR_2012,Line 380 (#Inv=7);95CI LW GrowthCurves
459
1067
917
1564
2101
1428
148
Leaf Width
Degree Days
0
10
20
30
40
50
0
200
400
600
800
1000
1200

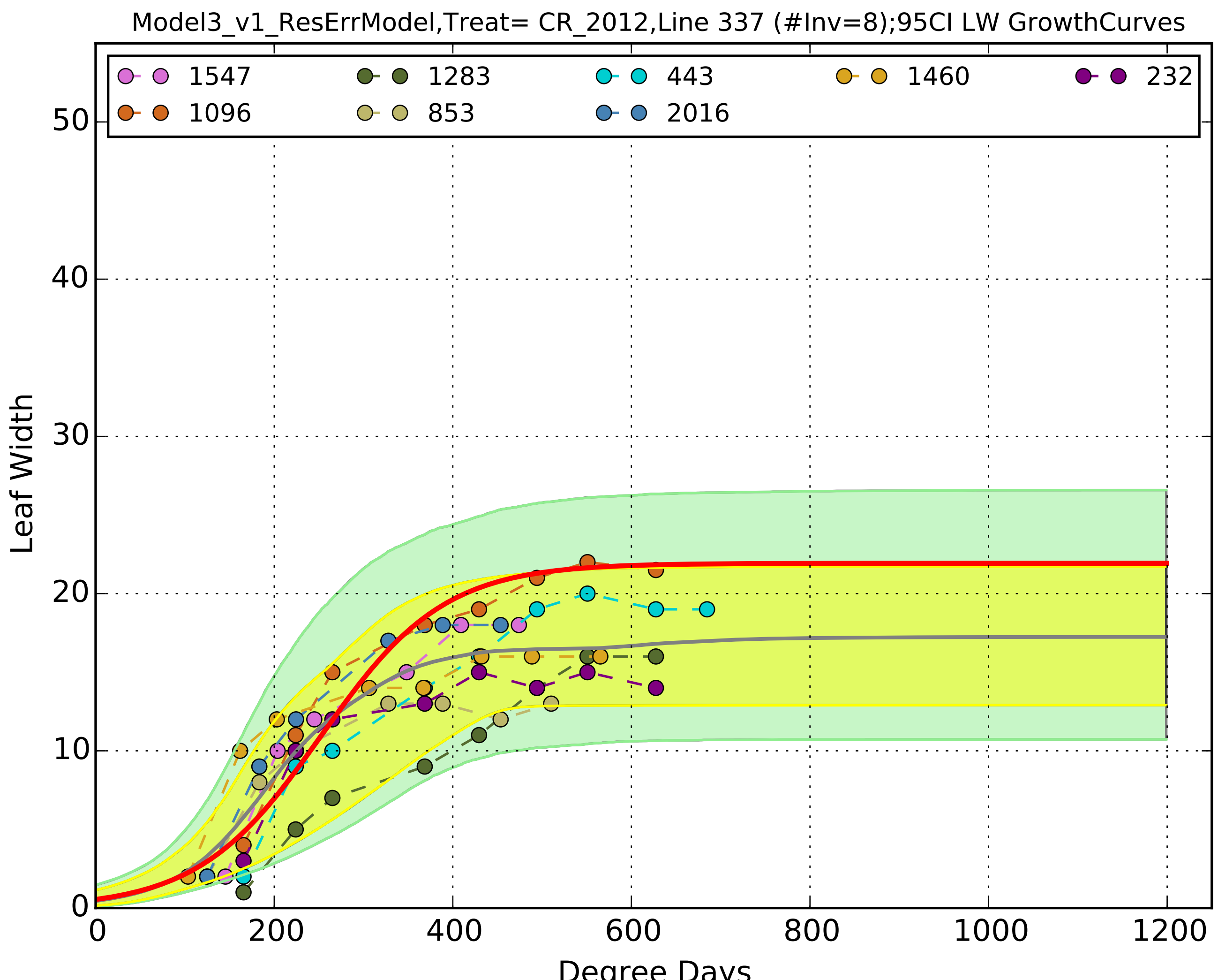
Model3_v1_ResErrModel,Treat= CR_2012,Line 337 (#Inv=8);95CI LW GrowthCurves
1547
1283
443
1460
232
1096
853
2016
Leaf Width
Degree Days
0
10
20
30
40
50
0
200
400
600
800
1000
1200

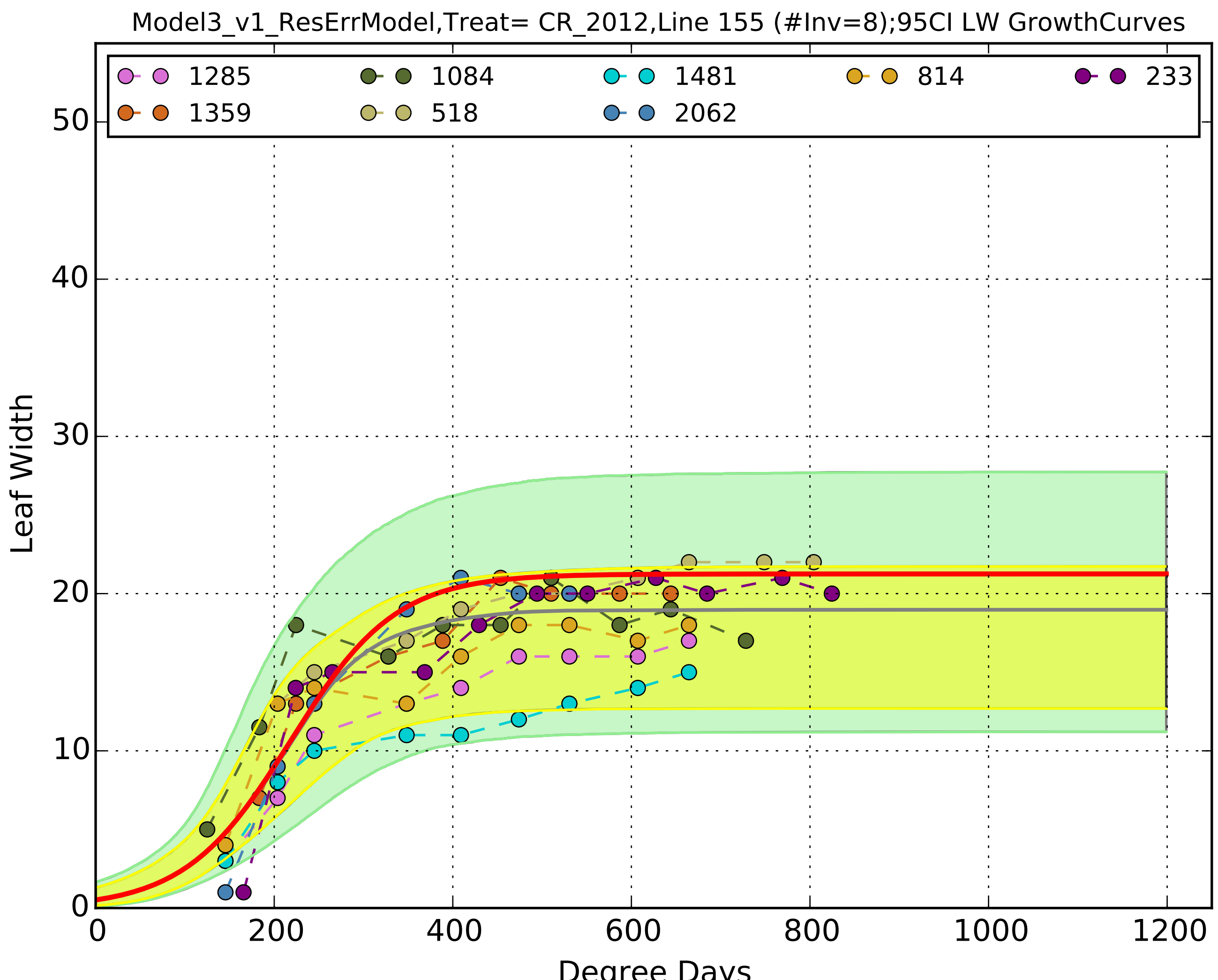

Model3_v1_ResErrModel,Treat= CR_2012,Line 155 (#Inv=8);95CI LW GrowthCurves
1285
1084
1481
814
233
1359
518
2062
Leaf Width
Degree Days
0
10
20
30
40
50
0
200
400
600
800
1000
1200

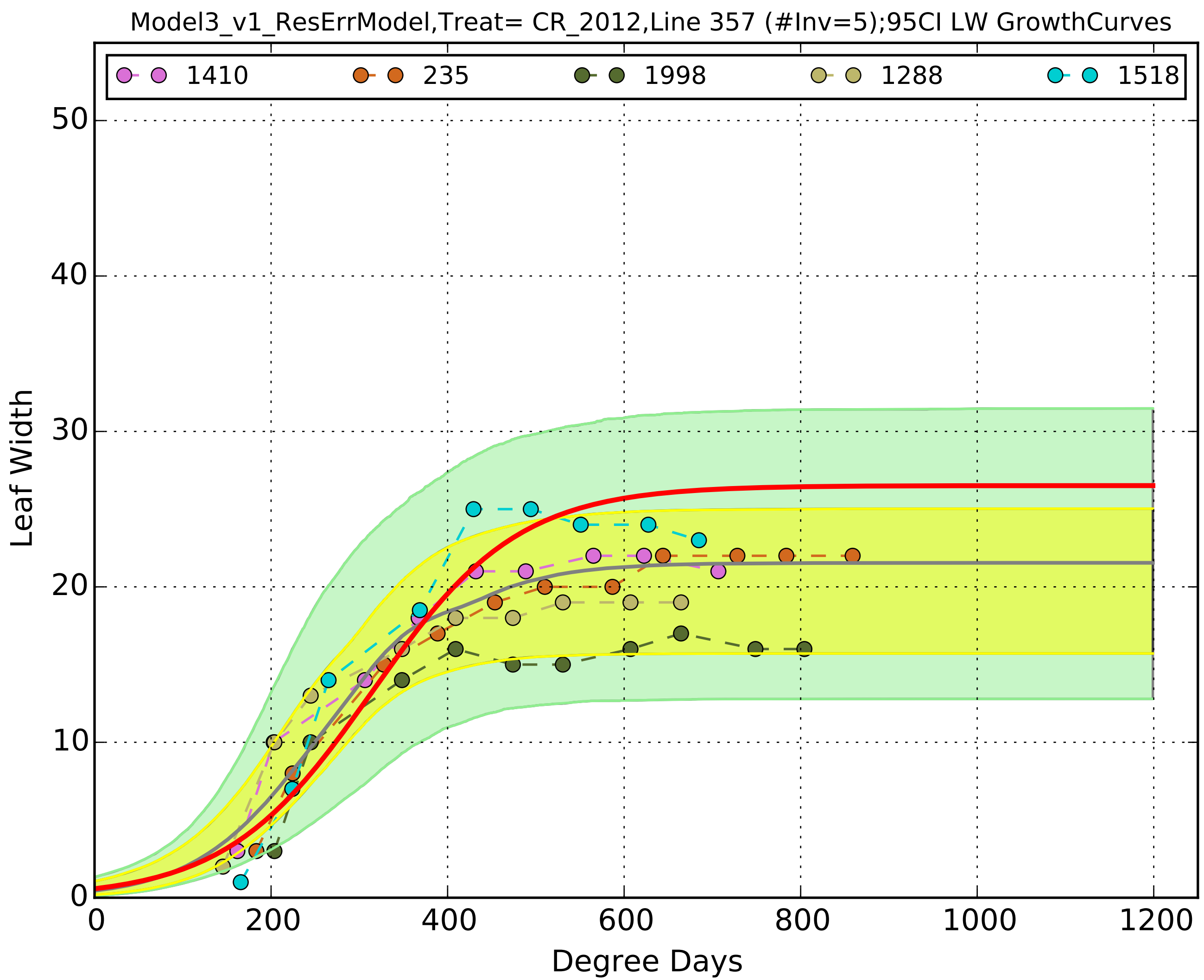

Model3_v1_ResErrModel,Treat= CR_2012,Line 357 (#Inv=5);95CI LW GrowthCurves
1410
235
1998
1288
1518
Leaf Width
Degree Days
0
10
20
30
40
50
0
200
400
600
800
1000
1200

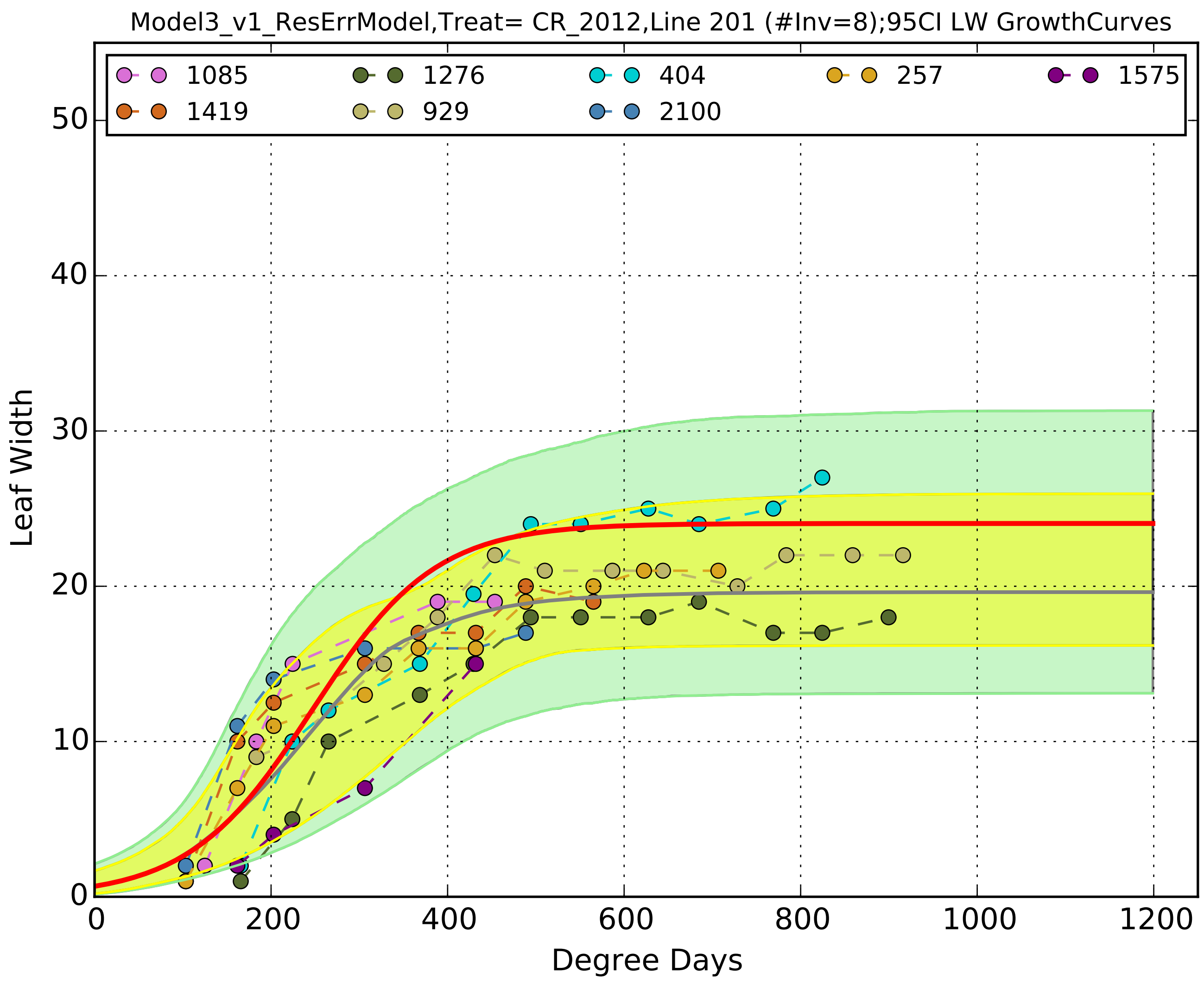

Model3_v1_ResErrModel,Treat= CR_2012,Line 201 (#Inv=8);95CI LW GrowthCurves
1085
1276
404
257
1575
1419
929
2100
Leaf Width
Degree Days
0
10
20
30
40
50
0
200
400
600
800
1000
1200

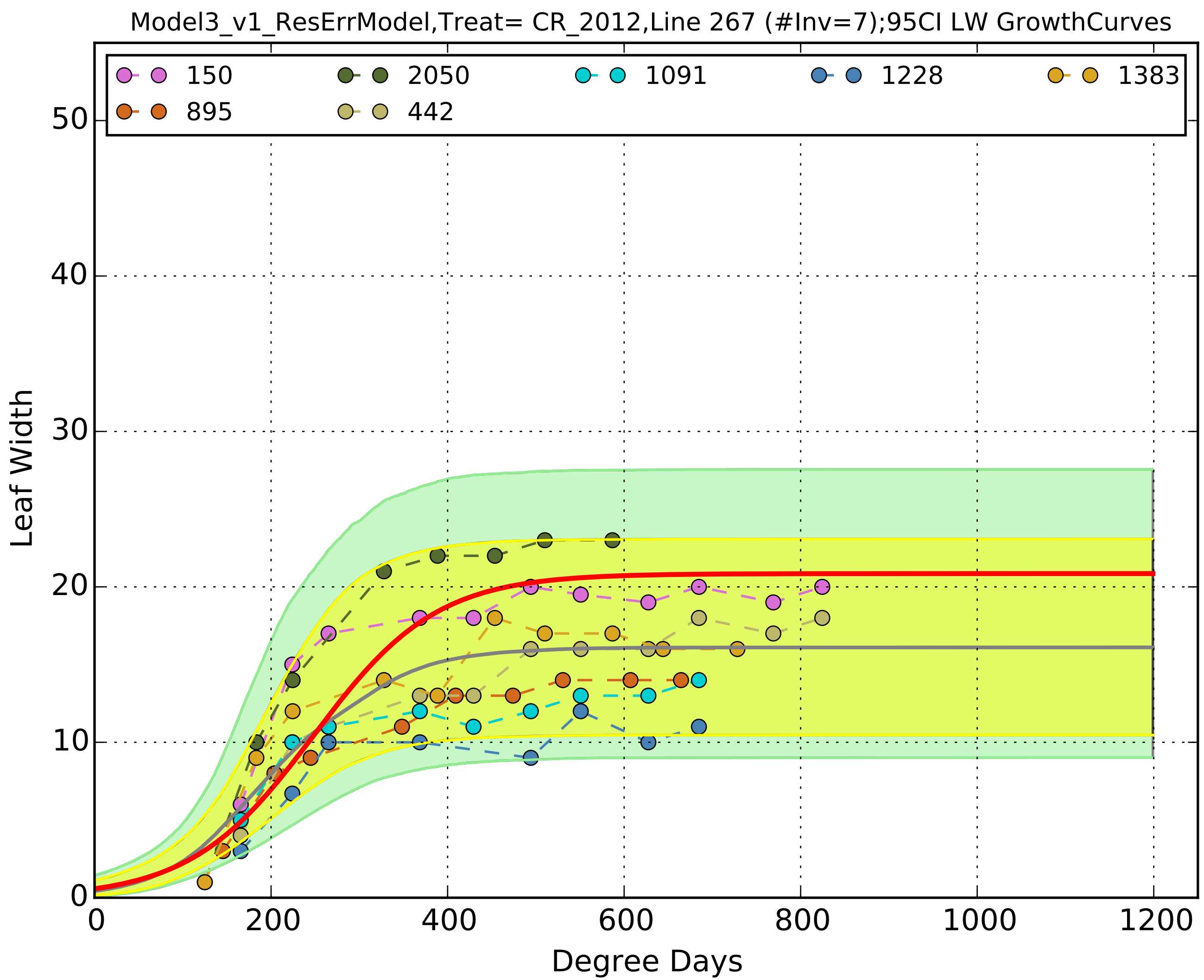

Model3_v1_ResErrModel,Treat= CR_2012,Line 267 (#Inv=7);95CI LW GrowthCurves
150
2050
1091
1228
1383
895
442
Leaf Width
Degree Days
0
10
20
30
40
50
0
200
400
600
800
1000
1200

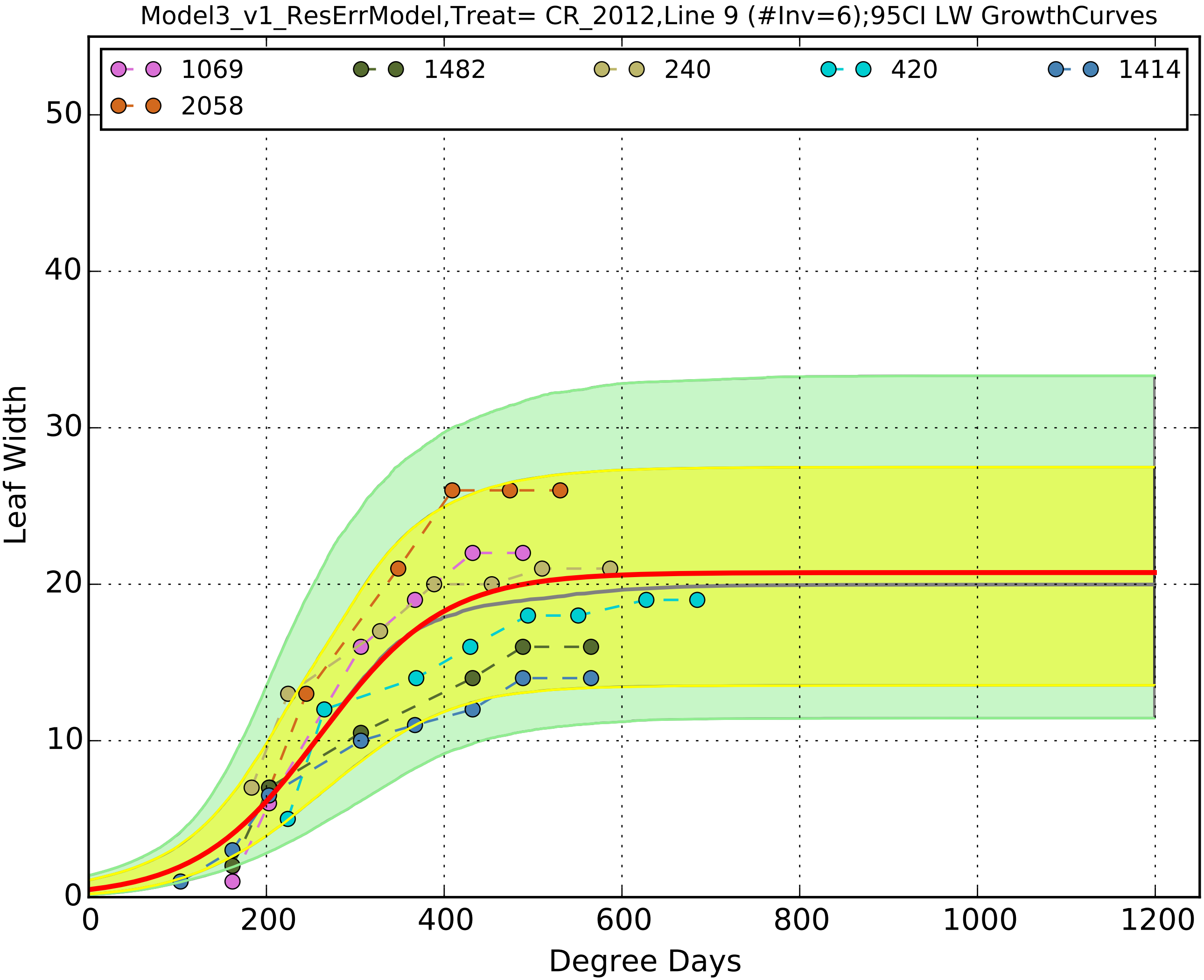

Model3_v1_ResErrModel,Treat= CR_2012,Line 9 (#Inv=6);95CI LW GrowthCurves
1069
1482
240
420
1414
2058
Leaf Width
Degree Days
0
10
20
30
40
50
0
200
400
600
800
1000
1200

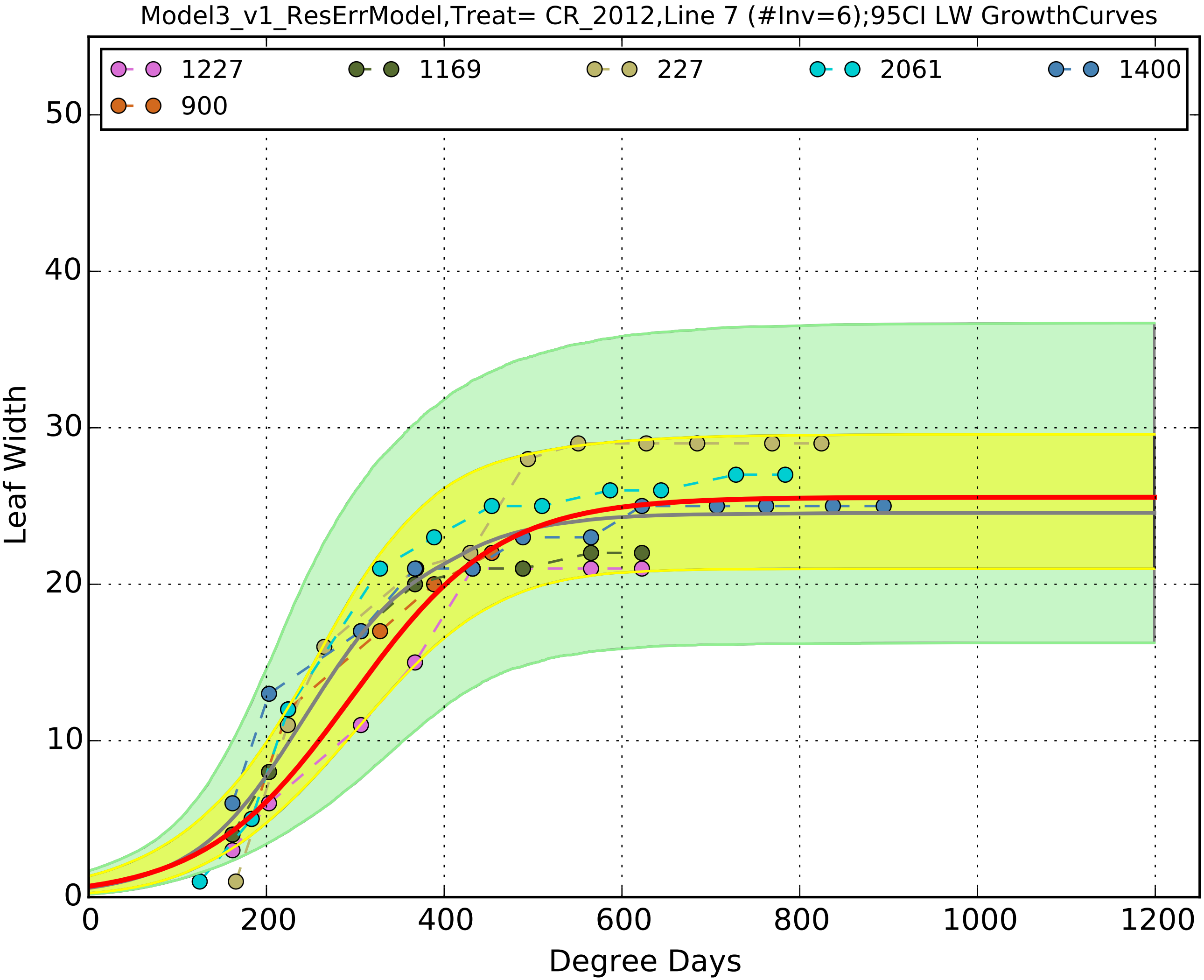

Model3_v1_ResErrModel,Treat= CR_2012,Line 7 (#Inv=6);95CI LW GrowthCurves
1227
1169
227
2061
1400
900
Leaf Width
Degree Days
0
10
20
30
40
50
0
200
400
600
800
1000
1200

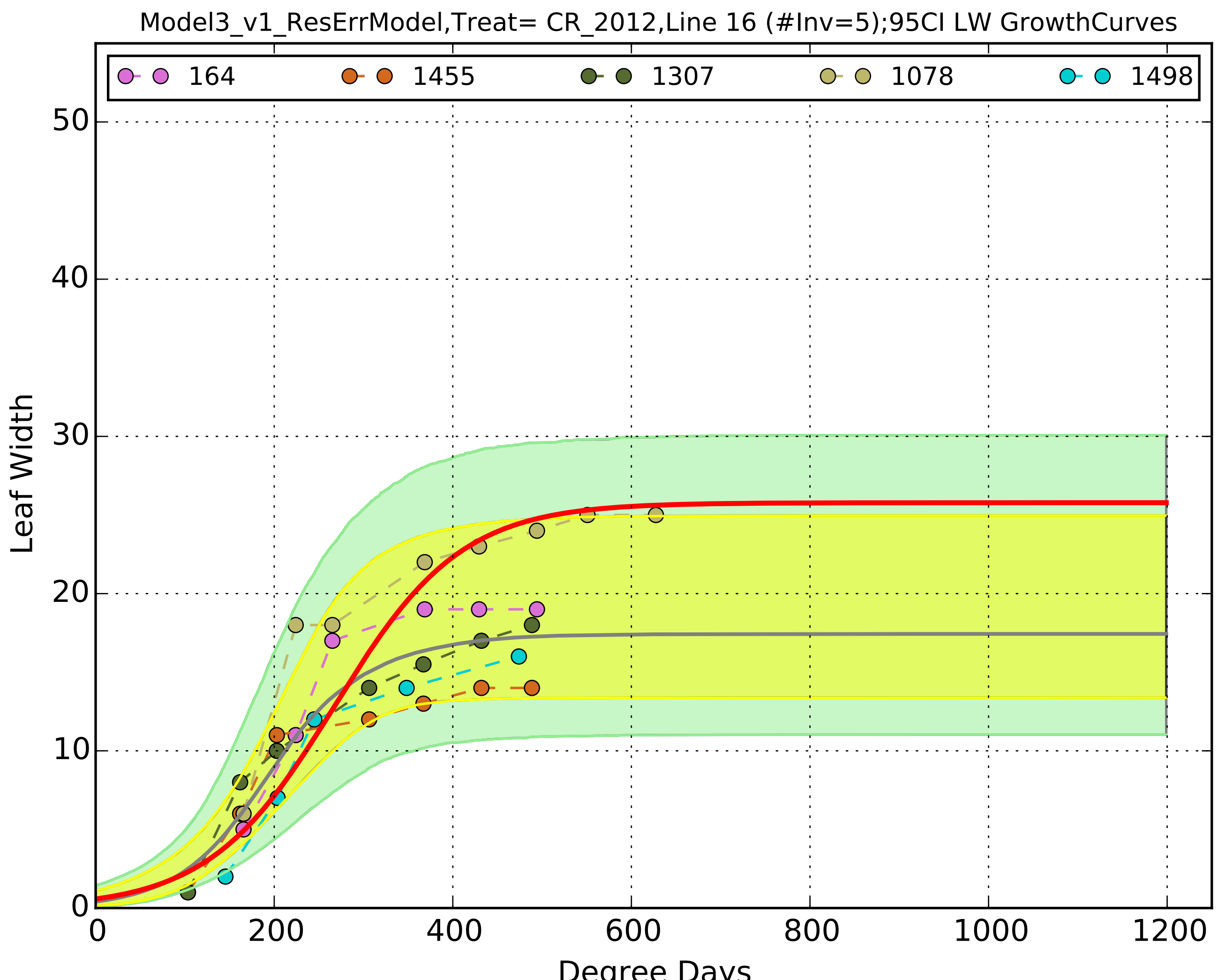

Model3_v1_ResErrModel,Treat= CR_2012,Line 16 (#Inv=5);95CI LW GrowthCurves
164
1455
1307
1078
1498
Leaf Width
Degree Days
0
10
20
30
40
50
0
200
400
600
800
1000
1200

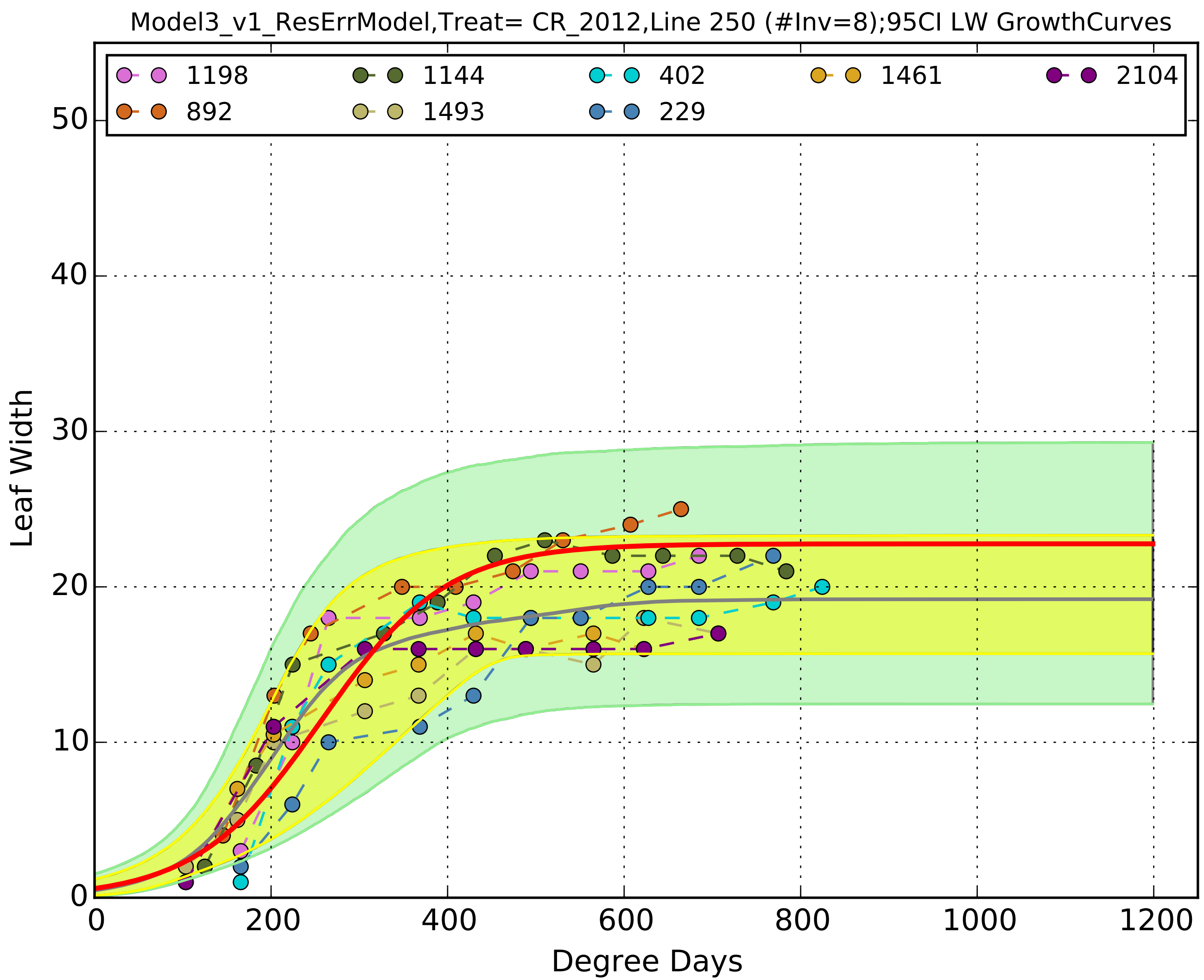
Model3_v1_ResErrModel,Treat= CR_2012,Line 250 (#Inv=8);95CI LW GrowthCurves
1198
1144
402
1461
2104
892
1493
229
Leaf Width
Degree Days
0
10
20
30
40
50
0
200
400
600
800
1000
1200

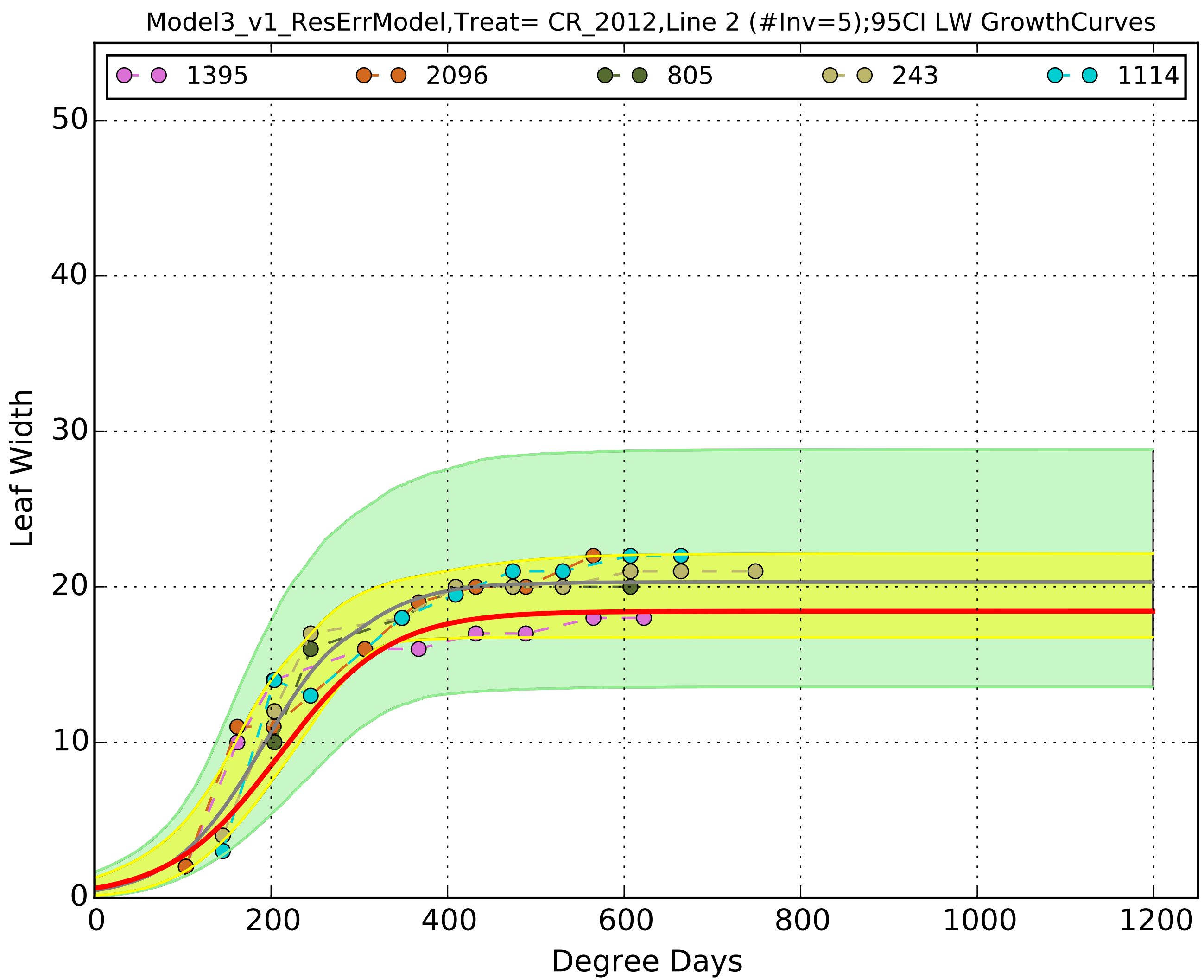
Model3_v1_ResErrModel,Treat= CR_2012,Line 2 (#Inv=5);95CI LW GrowthCurves
1395
2096
805
243
1114
Leaf Width
Degree Days
0
10
20
30
40
50
0
200
400
600
800
1000
1200

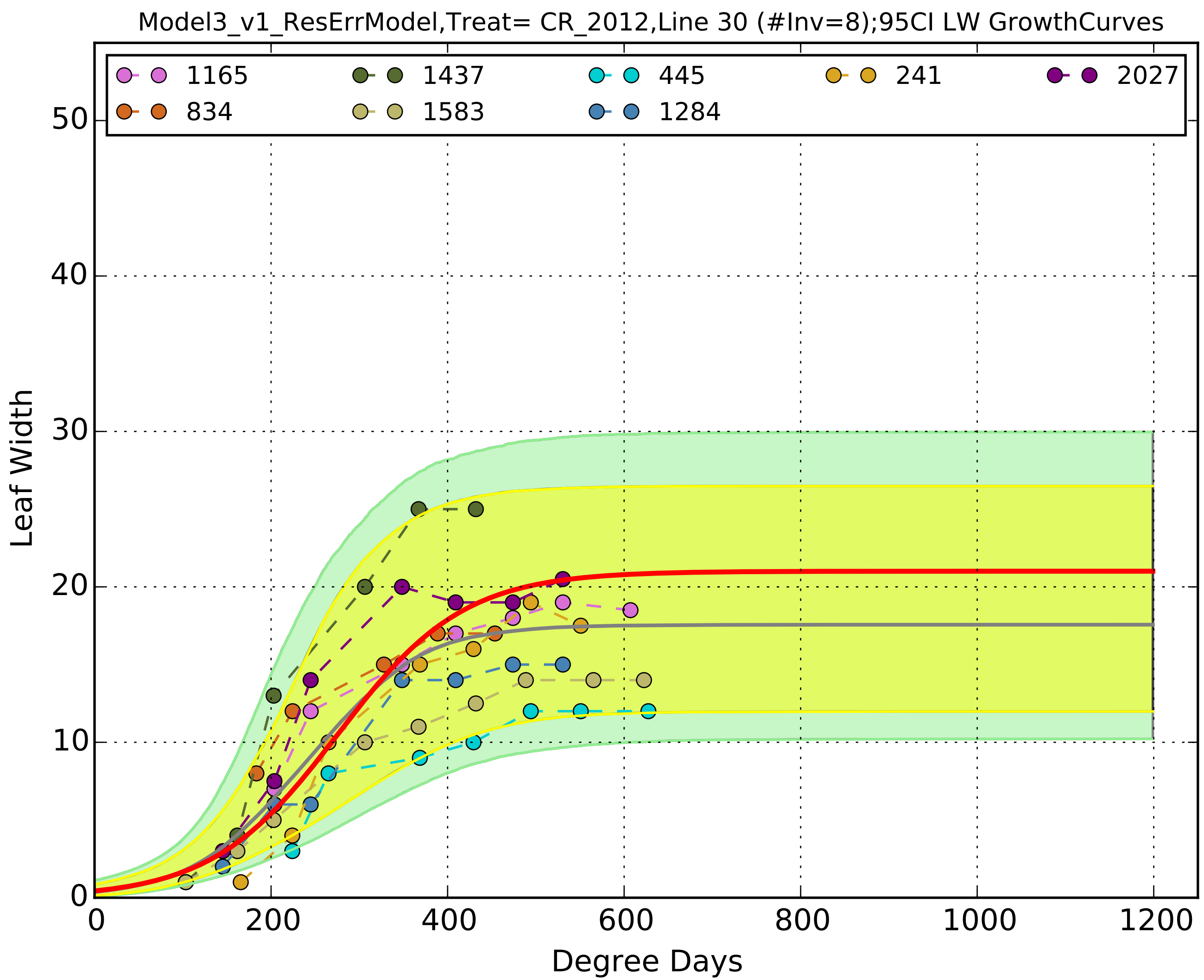

Model3_v1_ResErrModel,Treat= CR_2012,Line 30 (#Inv=8);95CI LW GrowthCurves
1165
1437
445
241
2027
834
1583
1284
Leaf Width
Degree Days
0
10
20
30
40
50
0
200
400
600
800
1000
1200

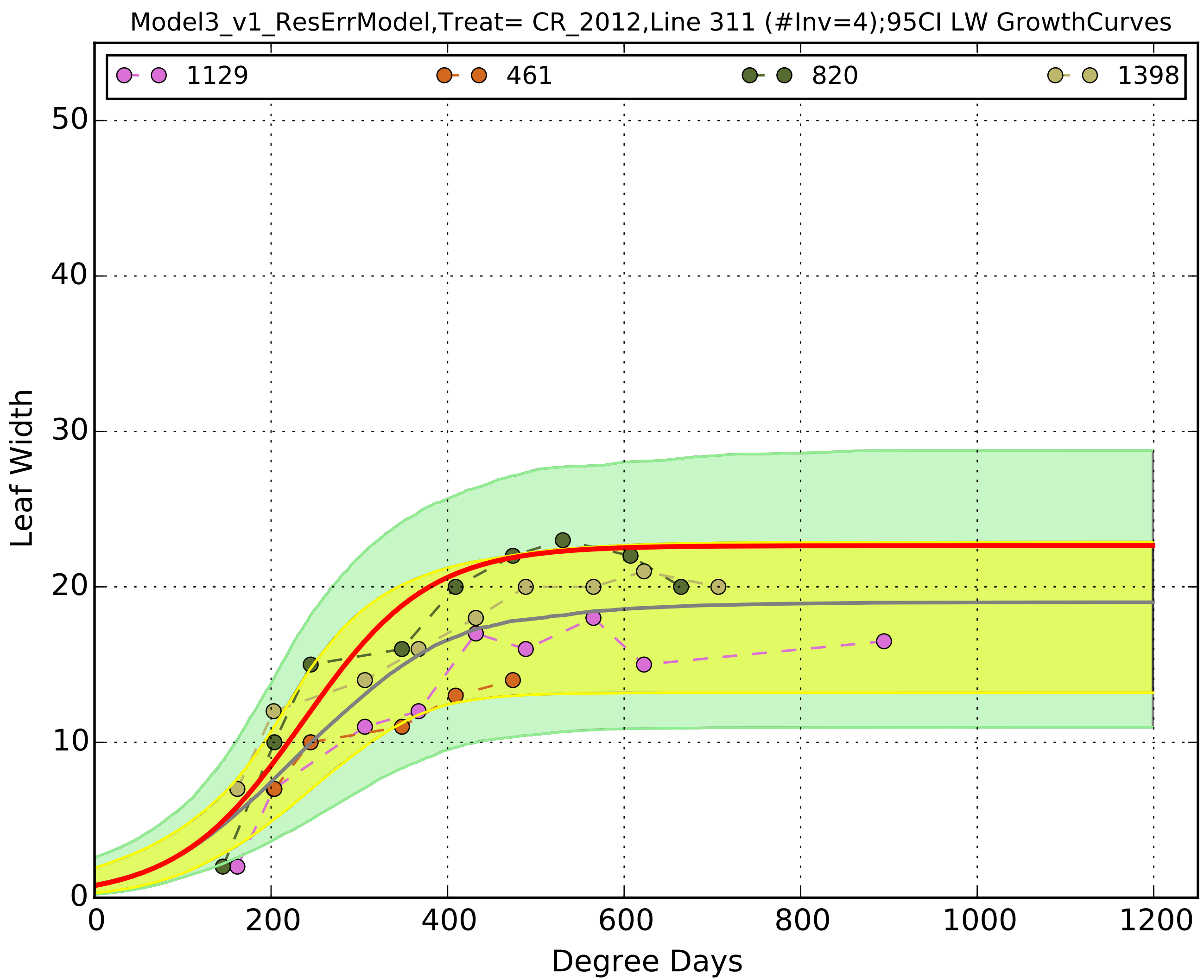

Model3_v1_ResErrModel,Treat= CR_2012,Line 311 (#Inv=4);95CI LW GrowthCurves
1129
461
820
1398
Leaf Width
Degree Days
0
10
20
30
40
50
0
200
400
600
800
1000
1200

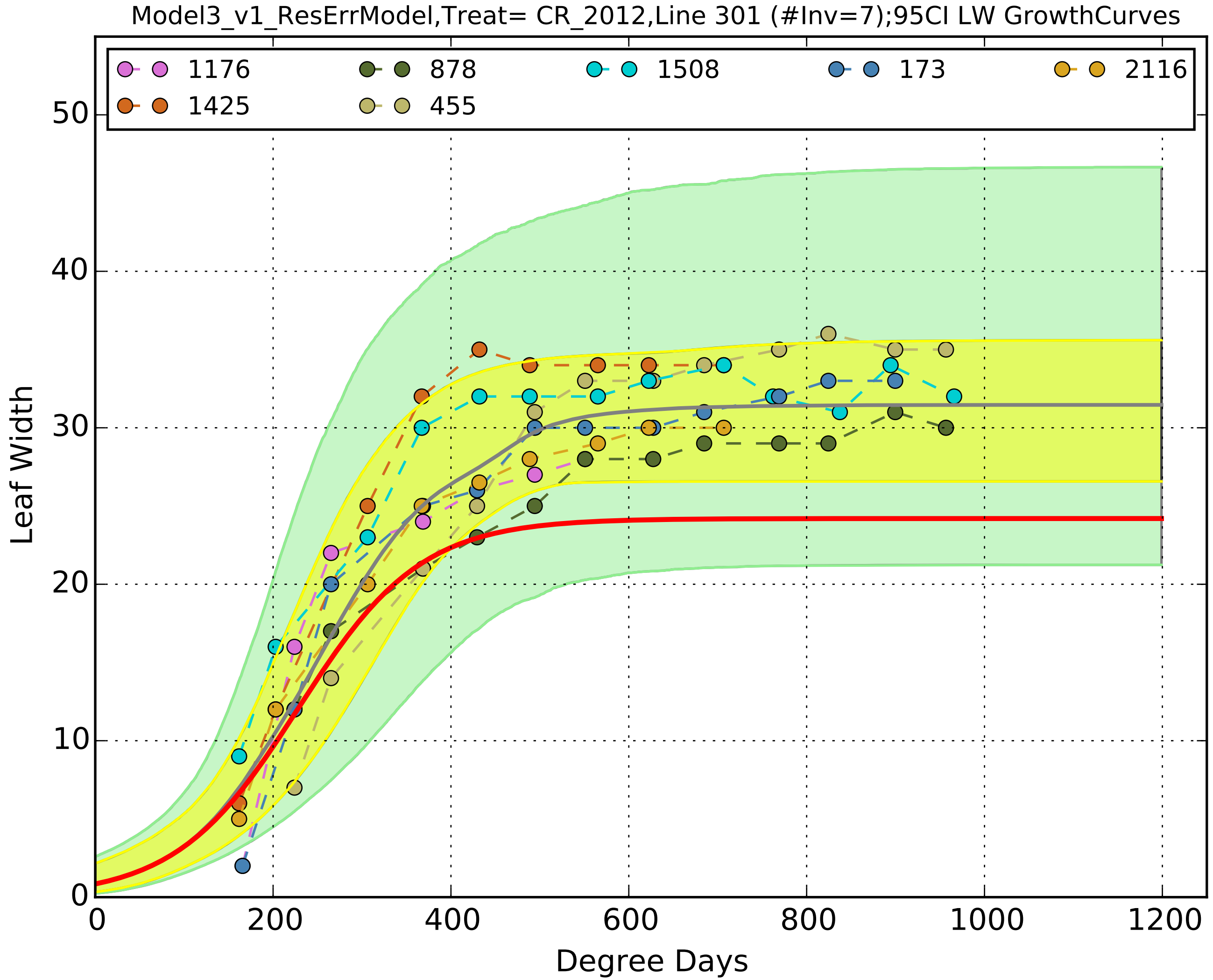
Model3_v1_ResErrModel,Treat= CR_2012,Line 301 (#Inv=7);95CI LW GrowthCurves
1176
878
1508
173
2116
1425
455
Leaf Width
Degree Days
0
10
20
30
40
50
0
200
400
600
800
1000
1200

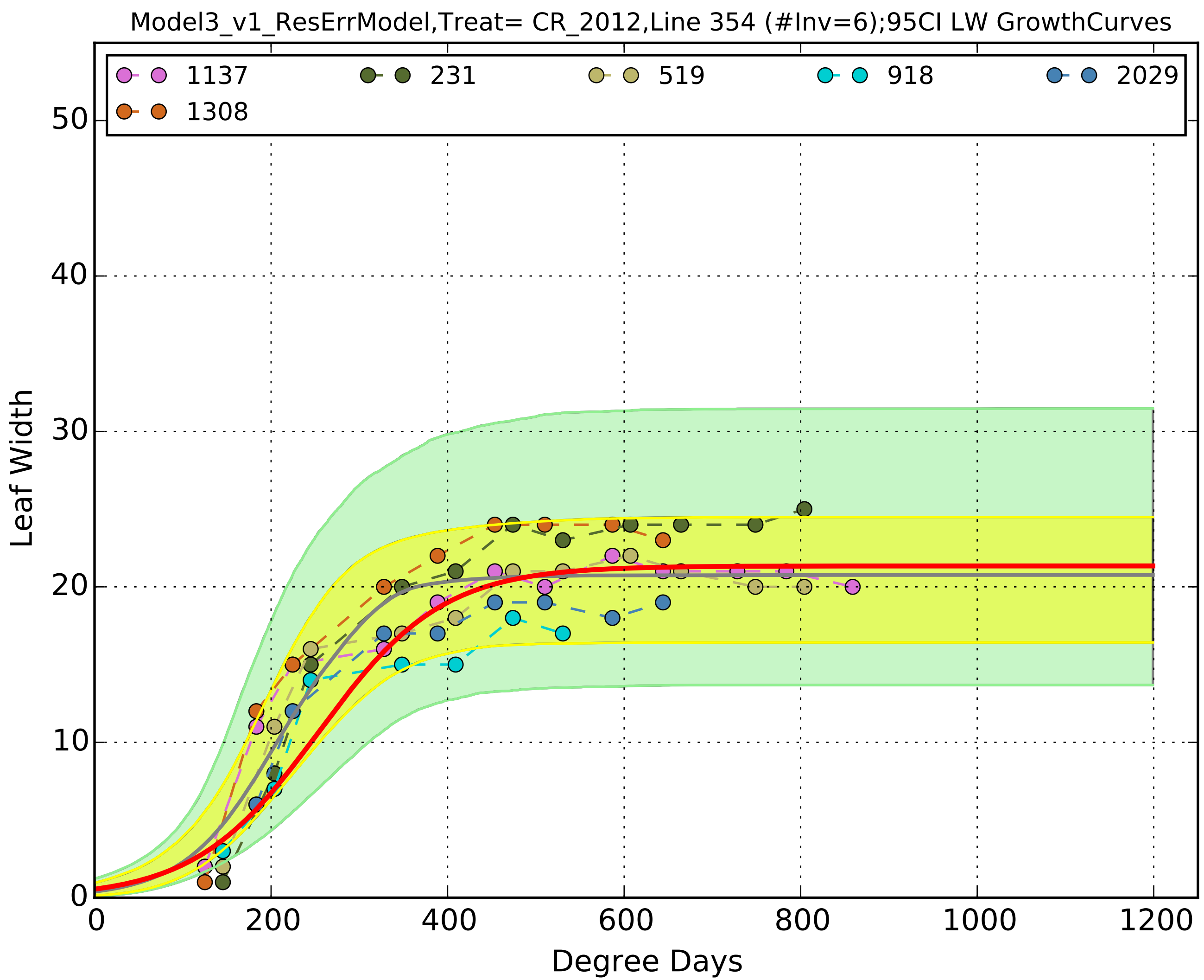
Model3_v1_ResErrModel,Treat= CR_2012,Line 354 (#Inv=6);95CI LW GrowthCurves
1137
231
519
918
2029
1308
Leaf Width
Degree Days
0
10
20
30
40
50
0
200
400
600
800
1000
1200

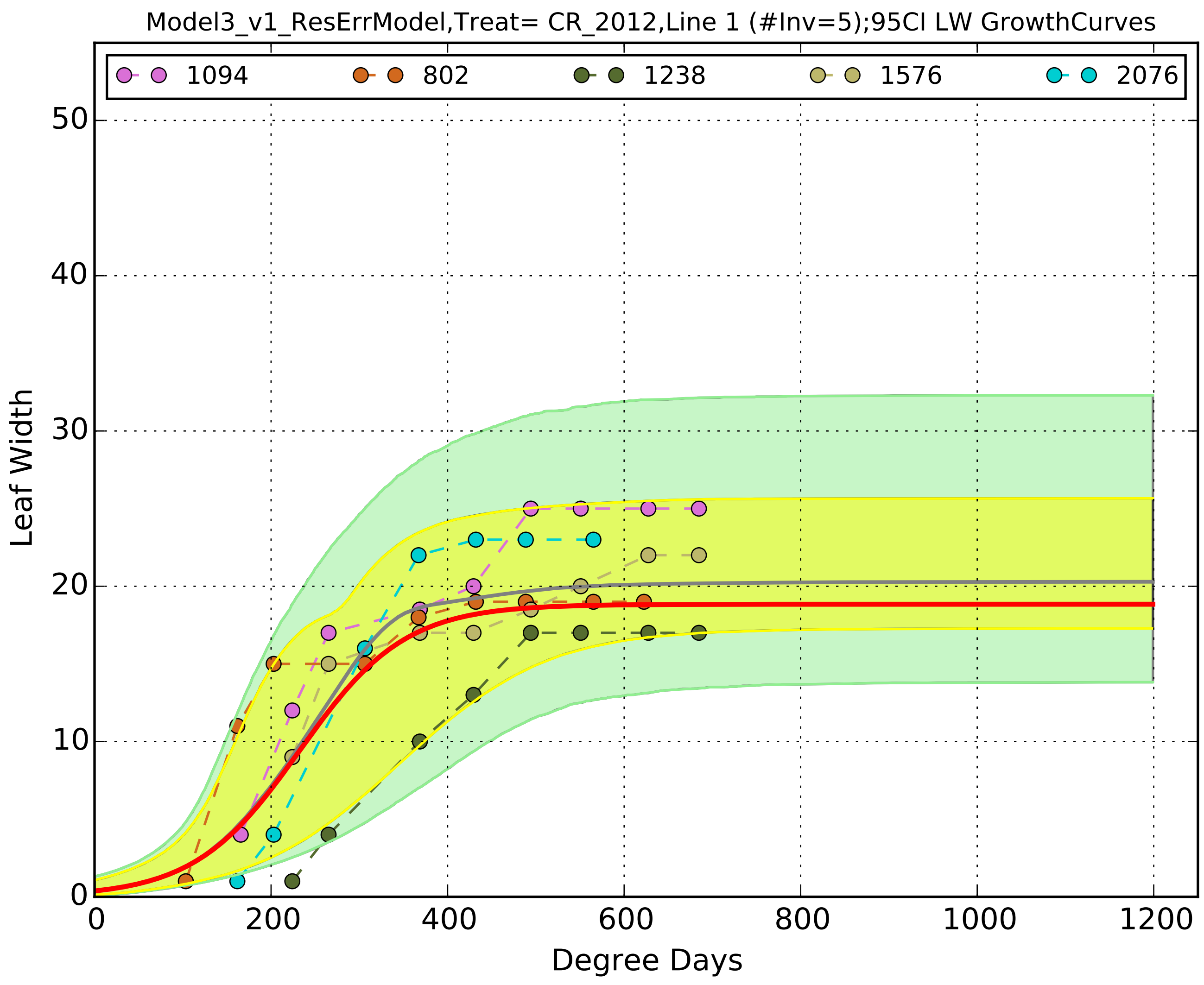

Model3_v1_ResErrModel,Treat= CR_2012,Line 1 (#Inv=5);95CI LW GrowthCurves
1094
802
1238
1576
2076
Leaf Width
Degree Days
0
10
20
30
40
50
0
200
400
600
800
1000
1200

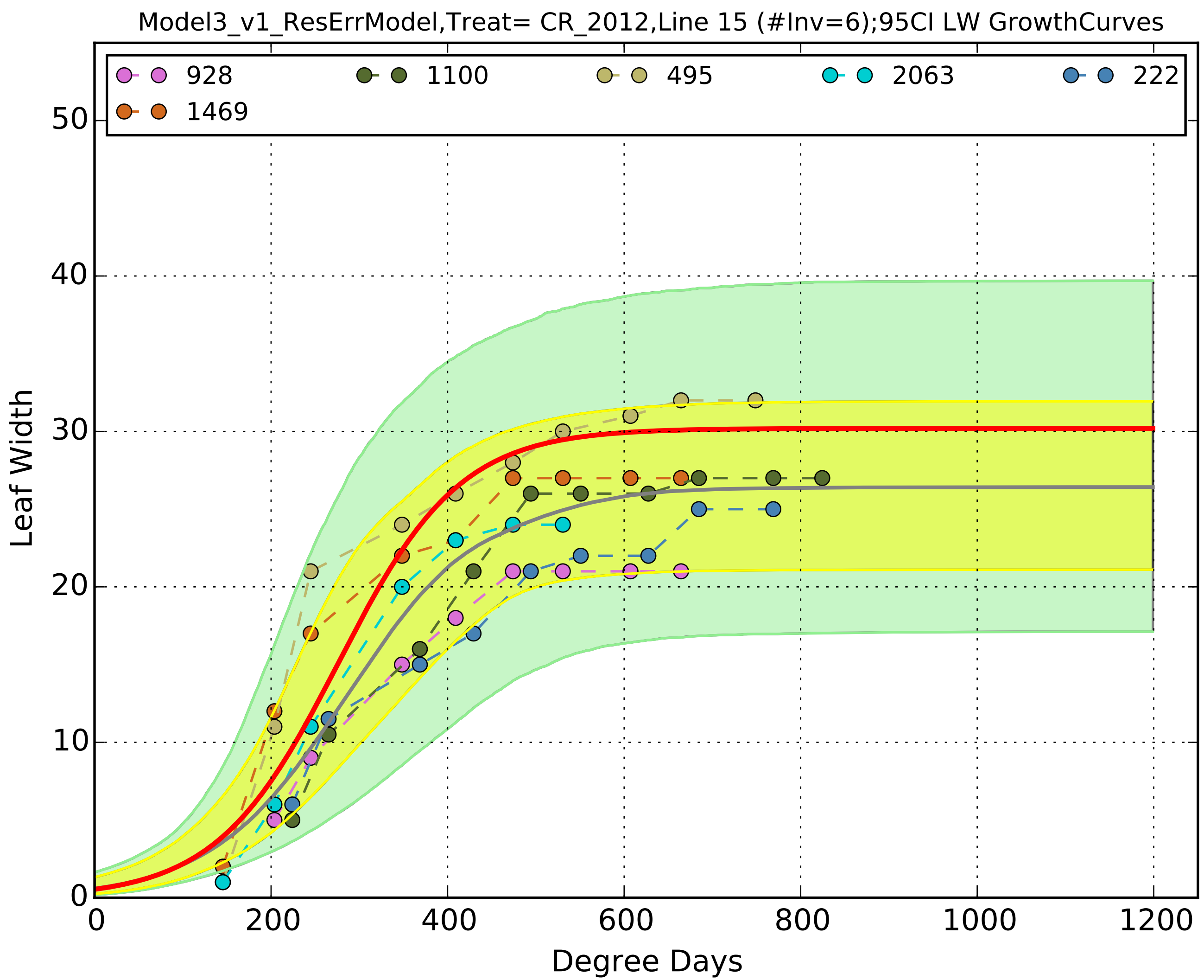
Model3_v1_ResErrModel,Treat= CR_2012,Line 15 (#Inv=6);95CI LW GrowthCurves
928
1100
495
2063
222
1469
Leaf Width
Degree Days
0
10
20
30
40
50
0
200
400
600
800
1000
1200

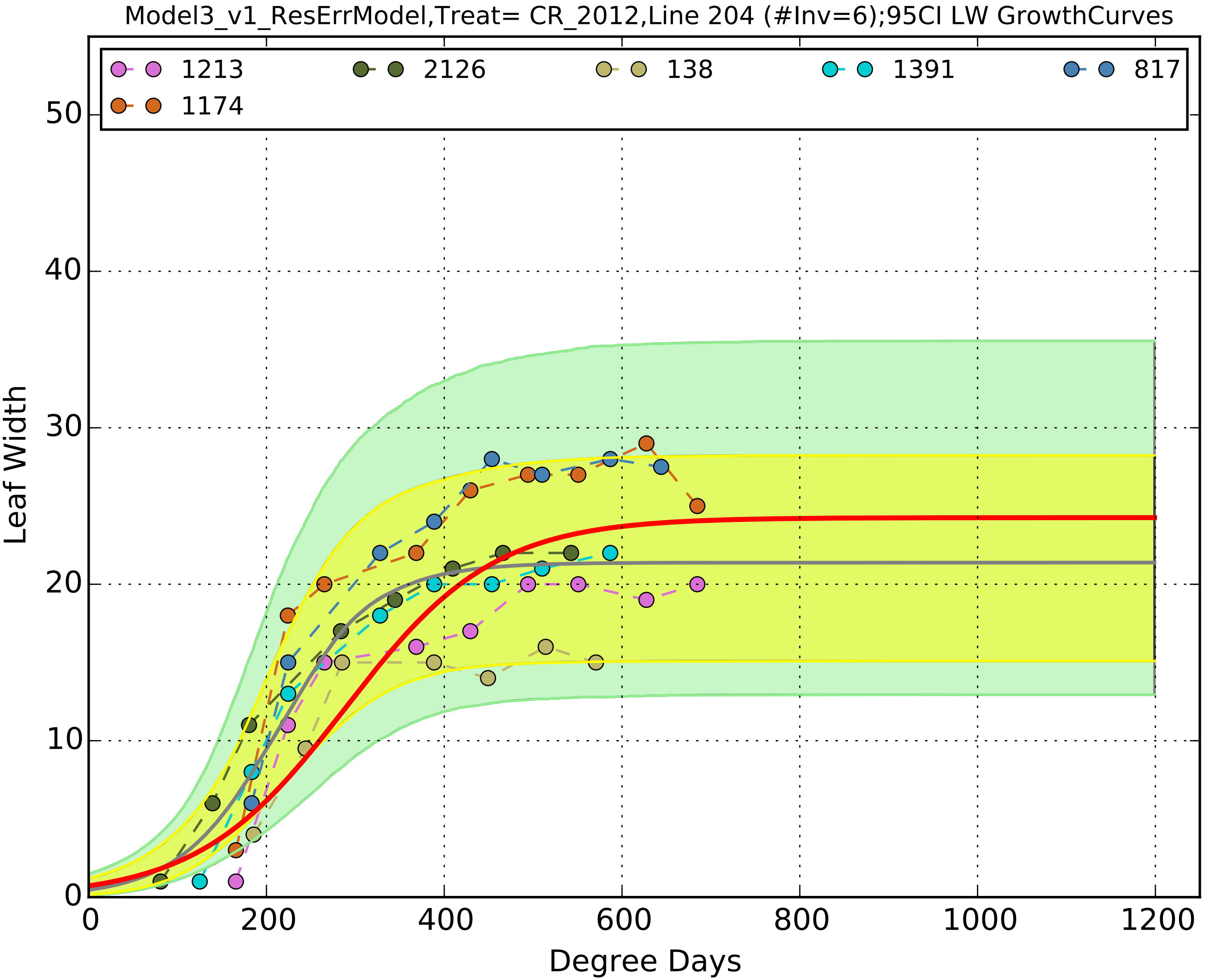

Model3_v1_ResErrModel,Treat= CR_2012,Line 204 (#Inv=6);95CI LW GrowthCurves
1213
2126
138
1391
817
1174
Leaf Width
Degree Days
0
10
20
30
40
50
0
200
400
600
800
1000
1200

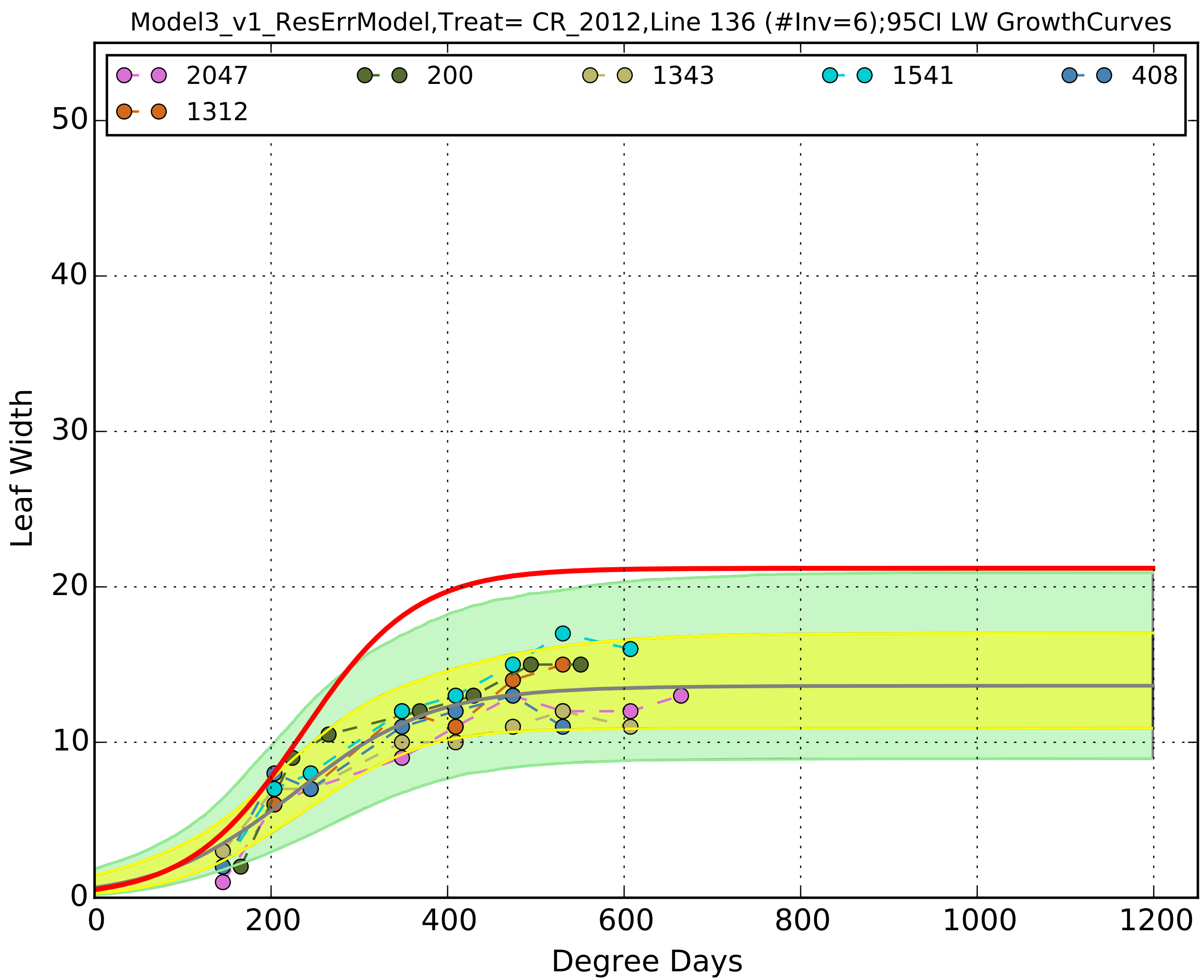

Model3_v1_ResErrModel,Treat= CR_2012,Line 136 (#Inv=6);95CI LW GrowthCurves
2047
200
1343
1541
408
1312
Leaf Width
Degree Days
0
10
20
30
40
50
0
200
400
600
800
1000
1200

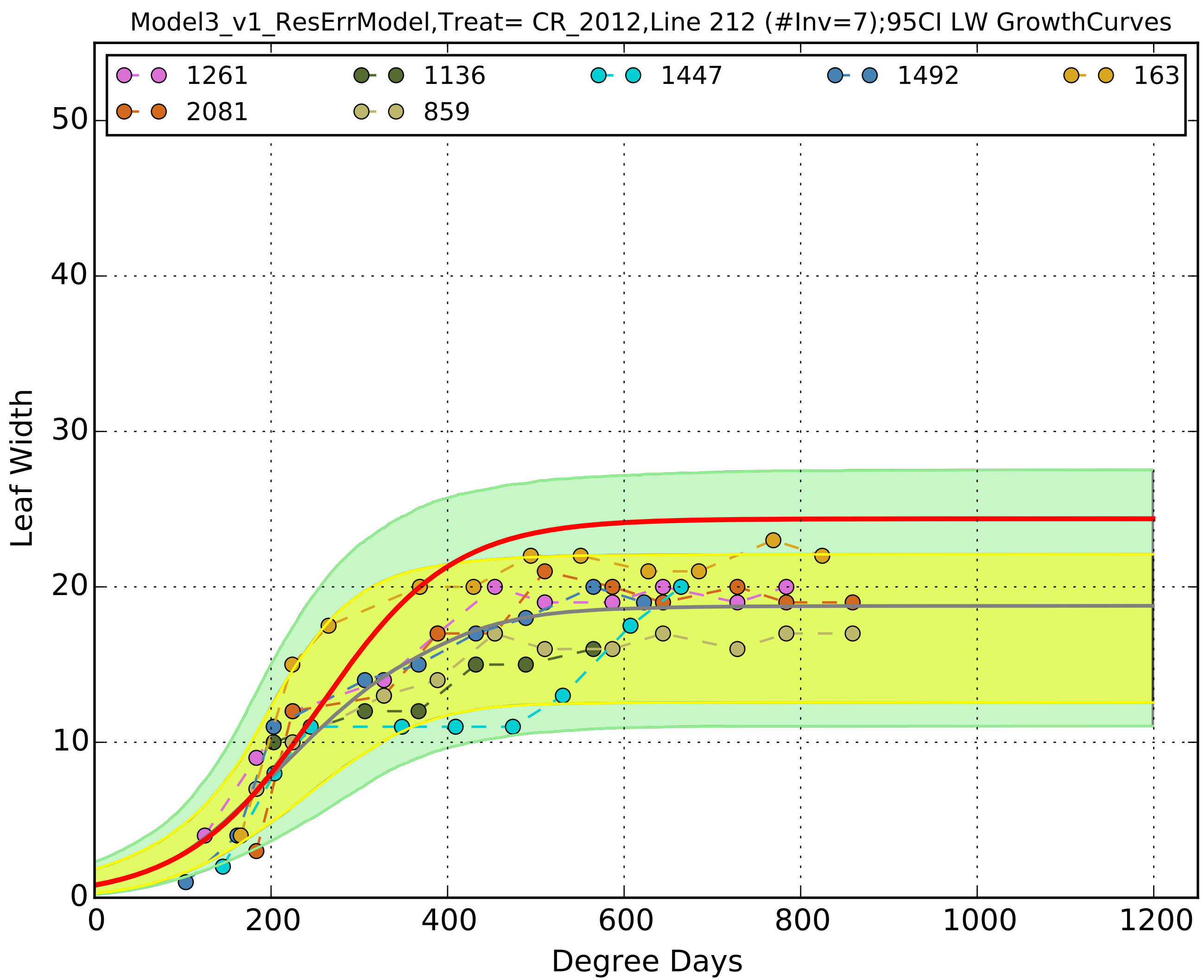

Model3_v1_ResErrModel,Treat= CR_2012,Line 212 (#Inv=7);95CI LW GrowthCurves
1261
1136
1447
1492
163
2081
859
Leaf Width
Degree Days
0
10
20
30
40
50
0
200
400
600
800
1000
1200

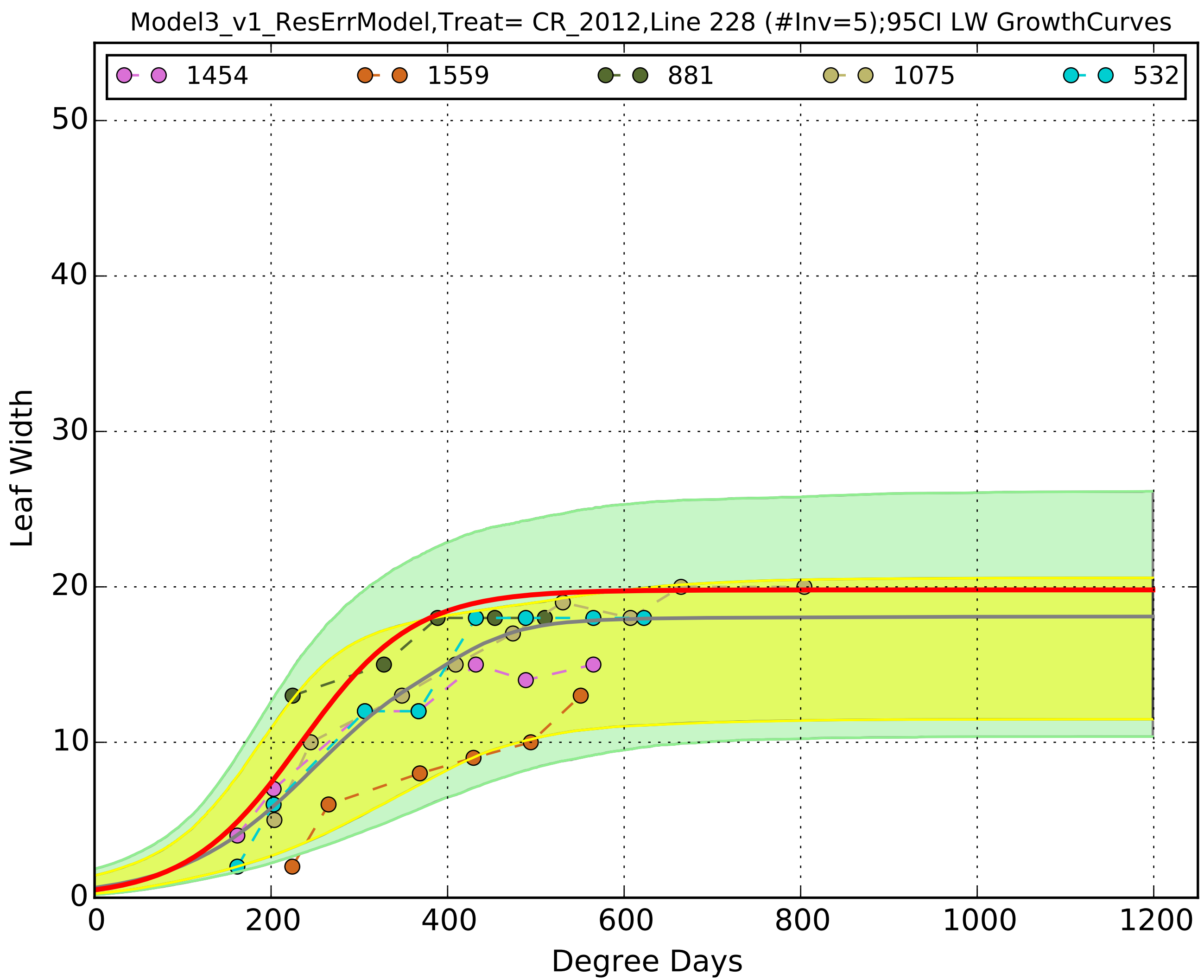
Model3_v1_ResErrModel,Treat= CR_2012,Line 228 (#Inv=5);95CI LW GrowthCurves
1454
1559
881
1075
532
Leaf Width
Degree Days
0
10
20
30
40
50
0
200
400
600
800
1000
1200

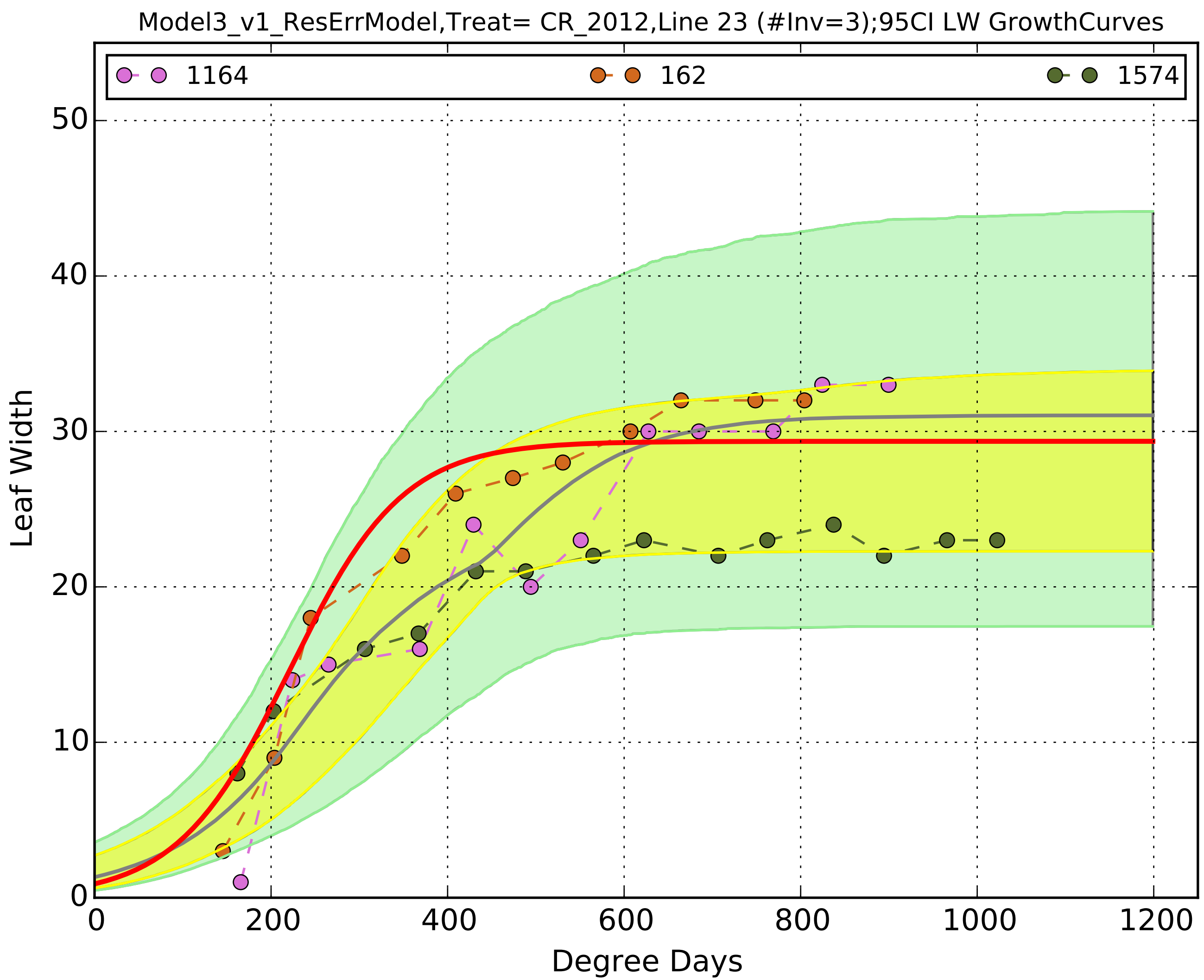

Model3_v1_ResErrModel,Treat= CR_2012,Line 23 (#Inv=3);95CI LW GrowthCurves
1164
162
1574
Leaf Width
Degree Days
0
10
20
30
40
50
0
200
400
600
800
1000
1200

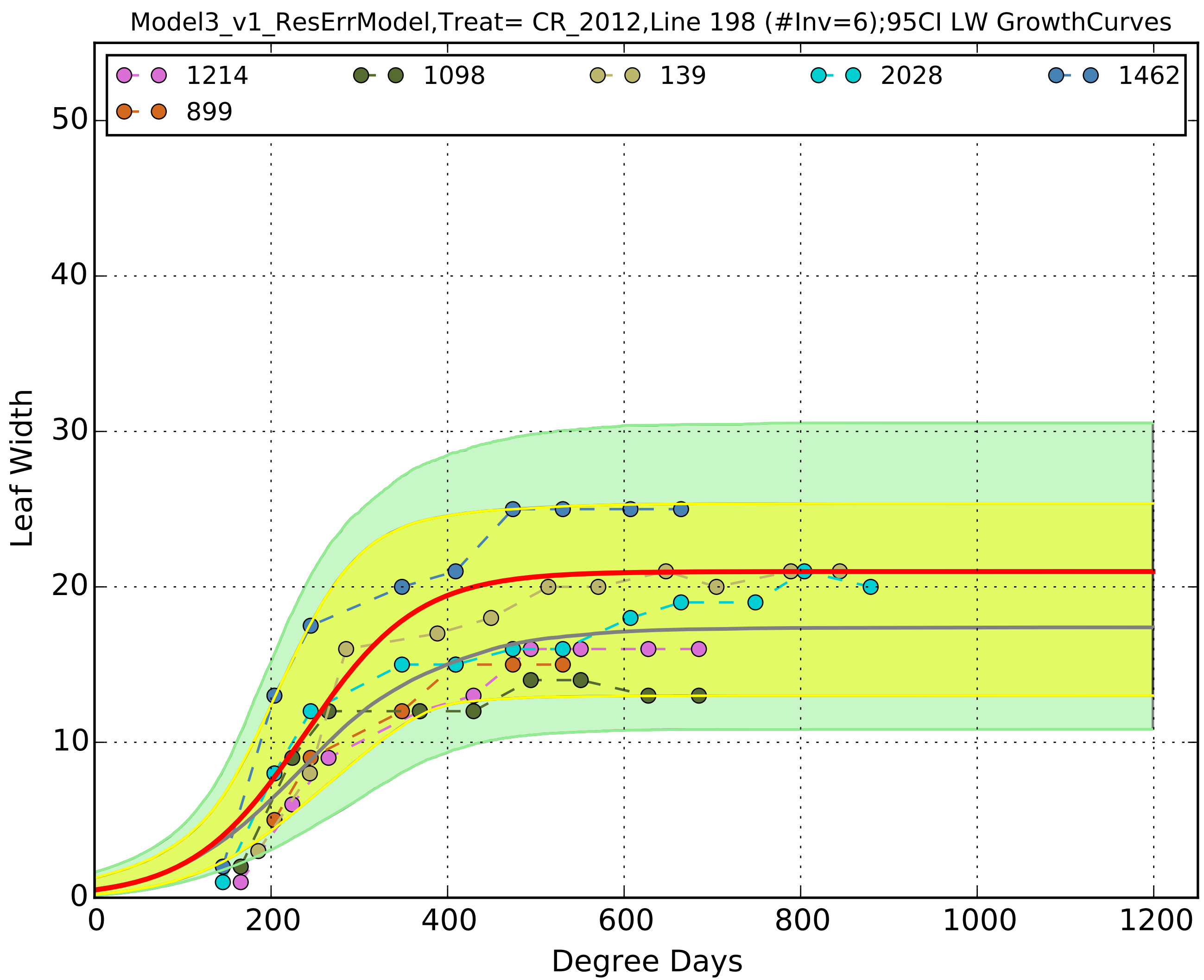
Model3_v1_ResErrModel,Treat= CR_2012,Line 198 (#Inv=6);95CI LW GrowthCurves
1214
1098
139
2028
1462
899
Leaf Width
Degree Days
0
10
20
30
40
50
0
200
400
600
800
1000
1200

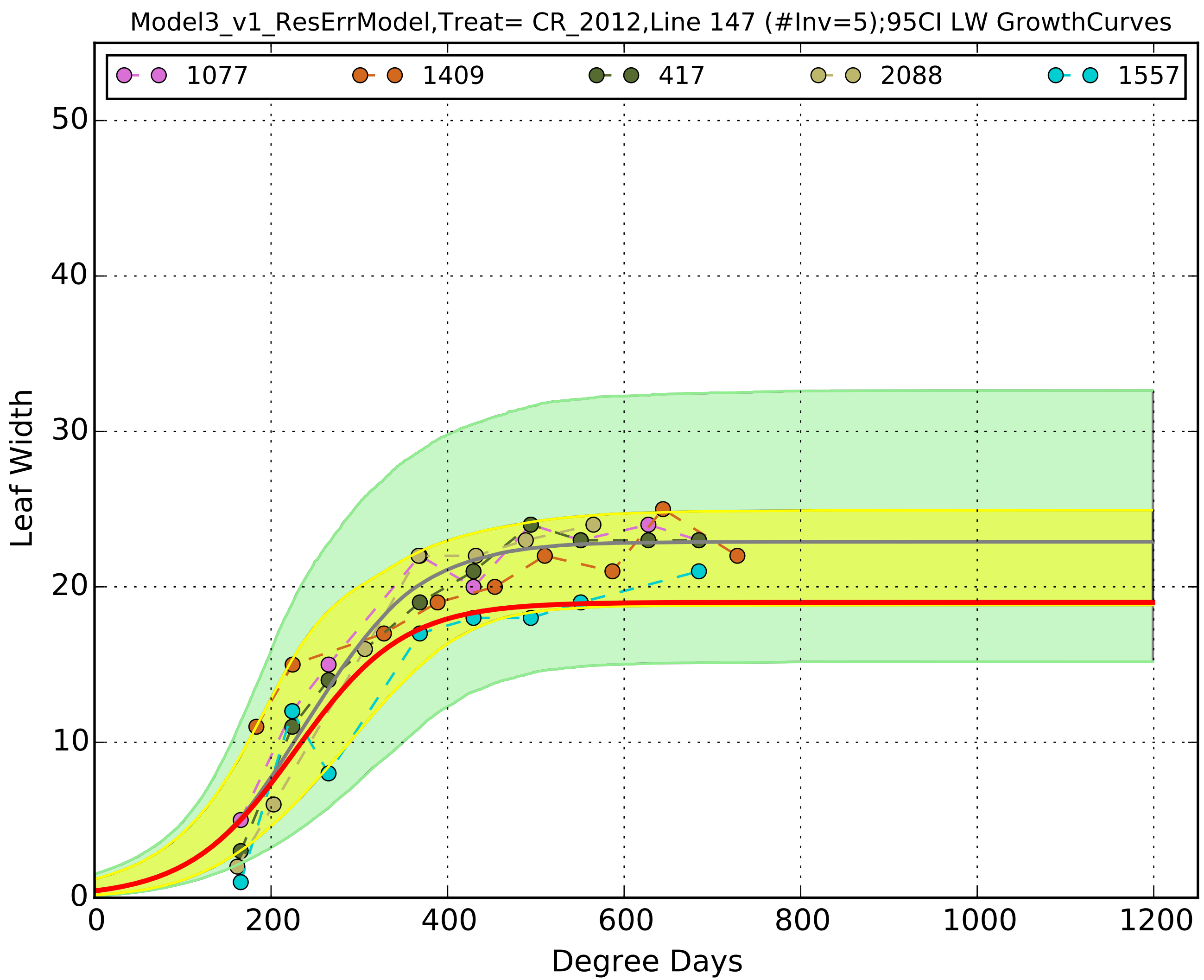

Model3_v1_ResErrModel,Treat= CR_2012,Line 147 (#Inv=5);95CI LW GrowthCurves
1077
1409
417
2088
1557
Leaf Width
Degree Days
0
10
20
30
40
50
0
200
400
600
800
1000
1200

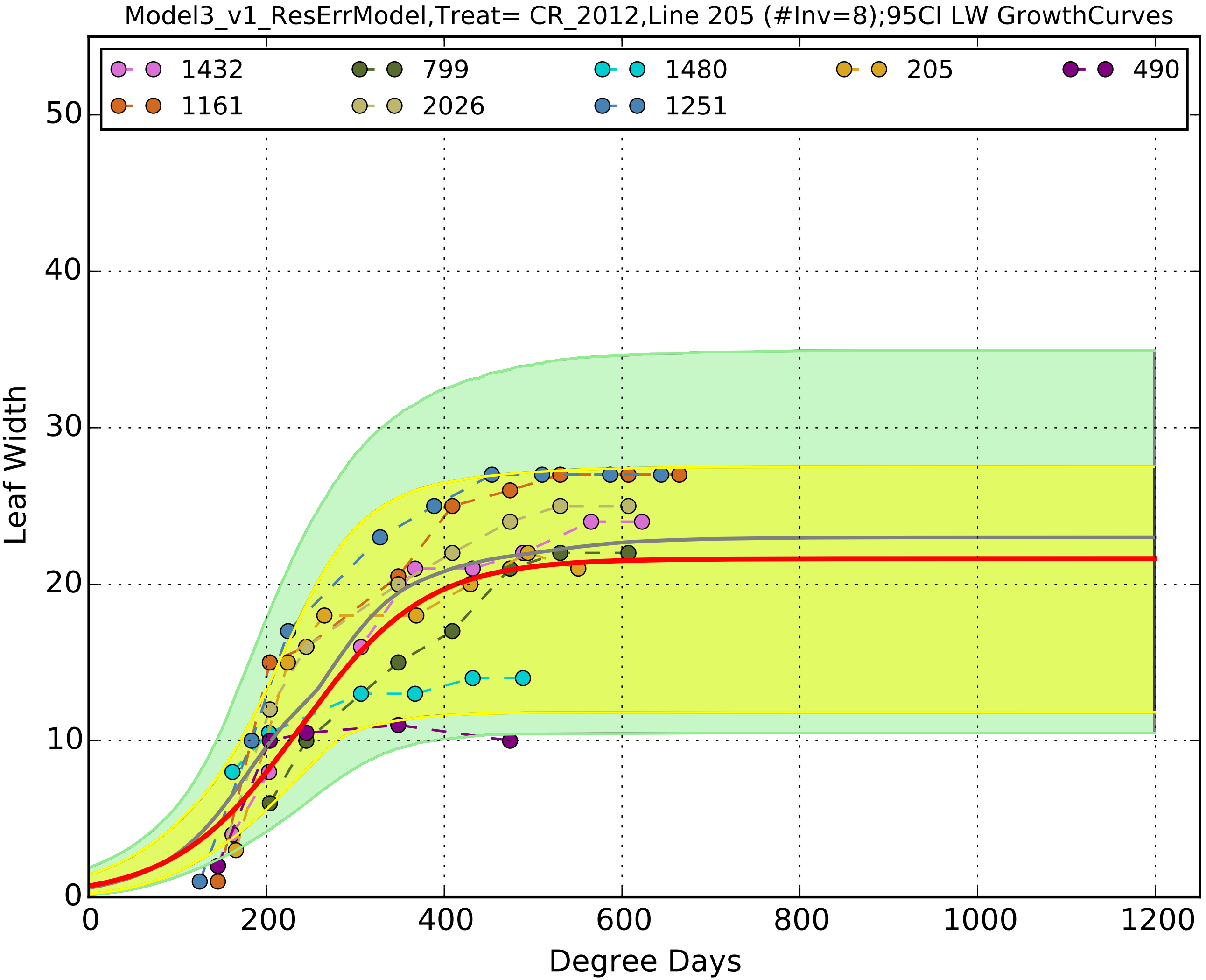

Model3_v1_ResErrModel,Treat= CR_2012,Line 205 (#Inv=8);95CI LW GrowthCurves
1432
1161
799
2026
1480
1251
205
490
Leaf Width
Degree Days
0
10
20
30
40
50
0
200
400
600
800
1000
1200

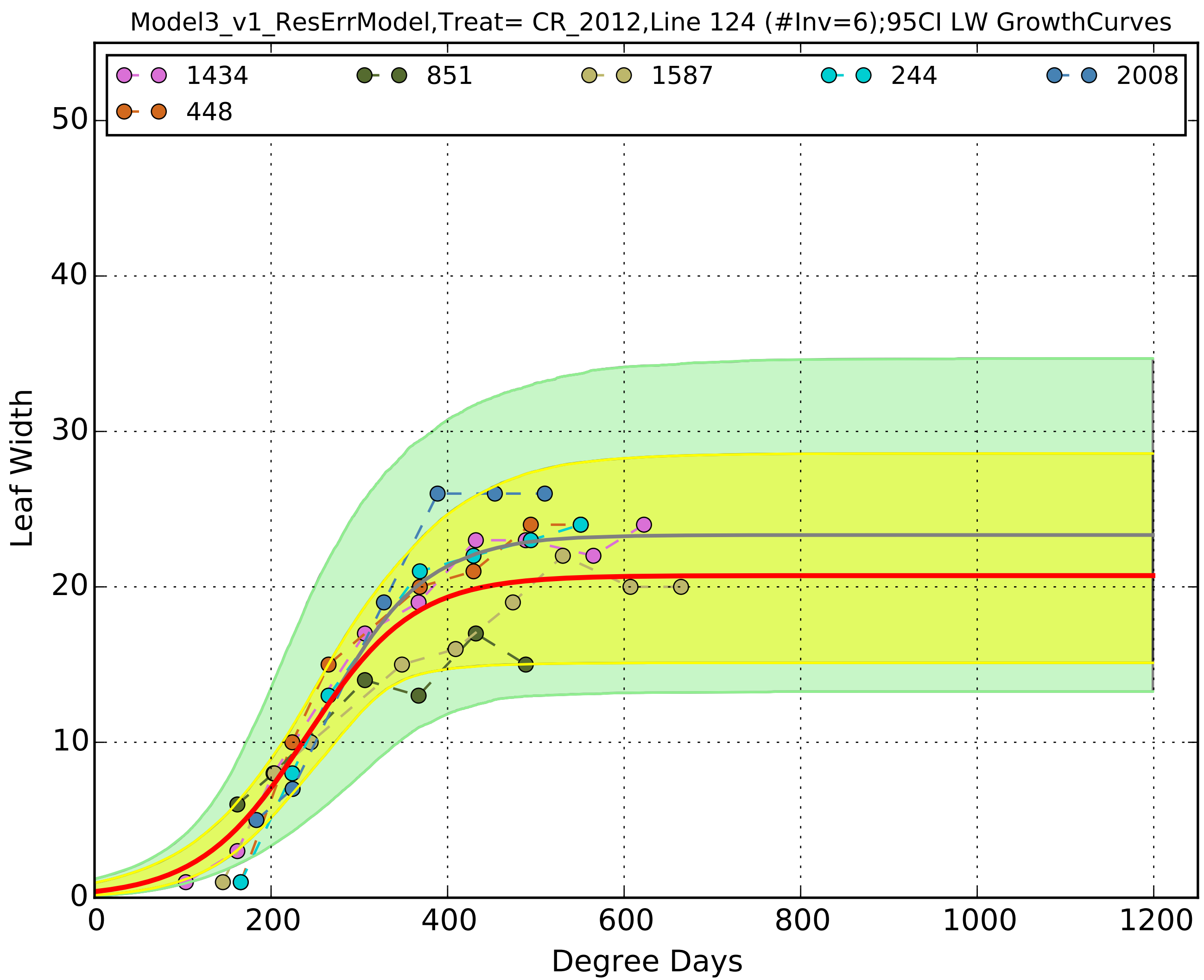

Model3_v1_ResErrModel,Treat= CR_2012,Line 124 (#Inv=6);95CI LW GrowthCurves
1434
851
1587
244
2008
448
Leaf Width
Degree Days
0
10
20
30
40
50
0
200
400
600
800
1000
1200

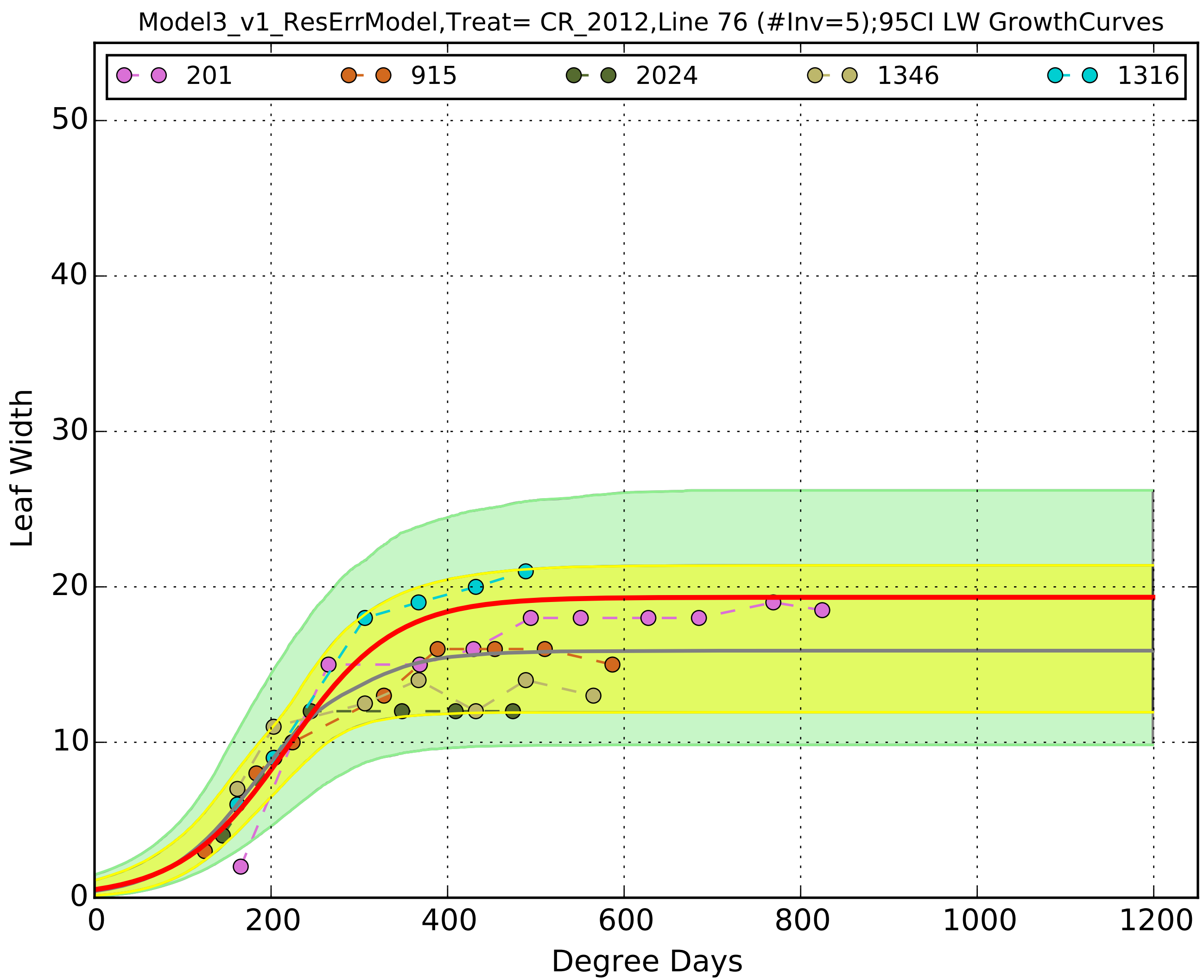
Model3_v1_ResErrModel,Treat= CR_2012,Line 76 (#Inv=5);95CI LW GrowthCurves
201
915
2024
1346
1316
Leaf Width
Degree Days
0
10
20
30
40
50
0
200
400
600
800
1000
1200

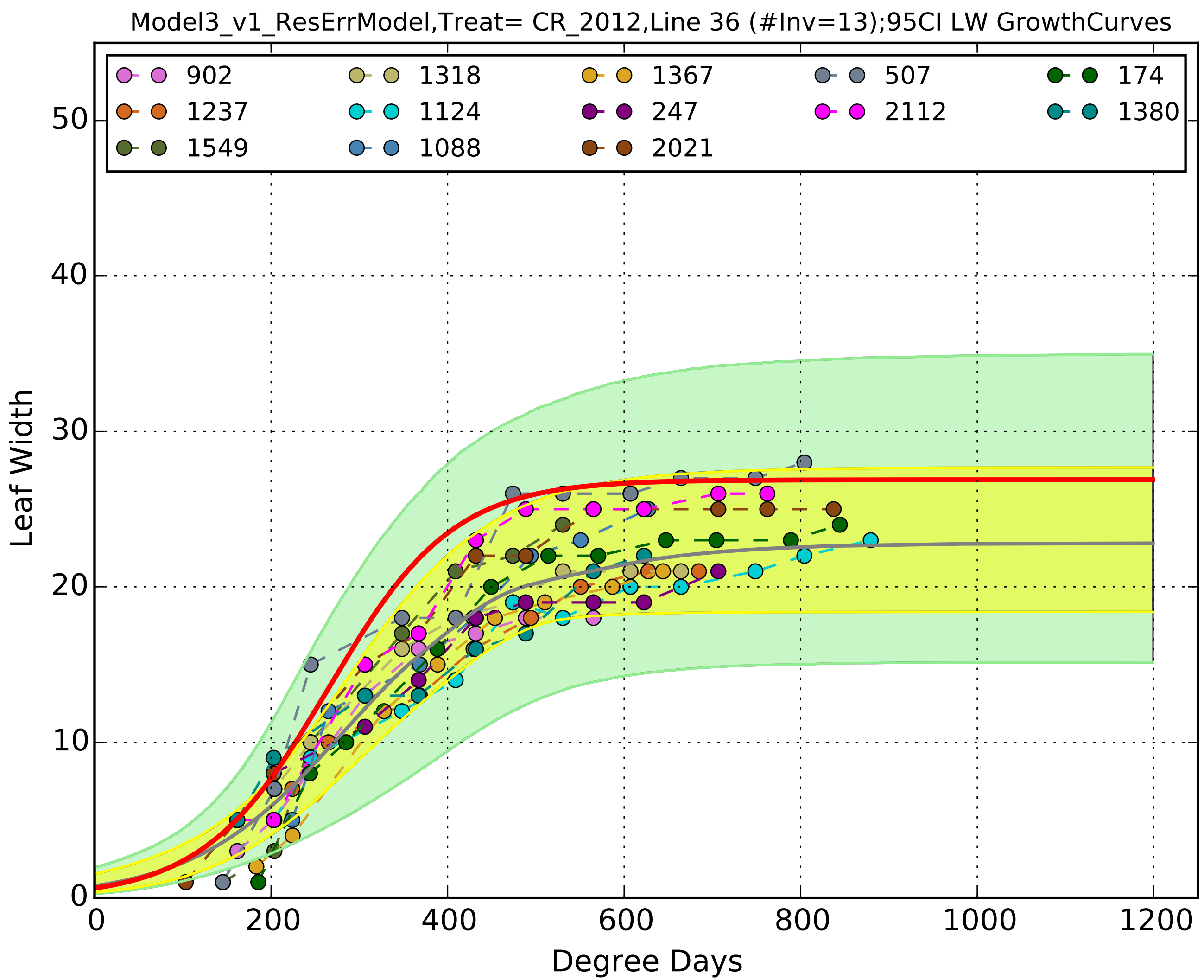

Model3_v1_ResErrModel,Treat= CR_2012,Line 36 (#Inv=13);95CI LW GrowthCurves
902
1318
1367
507
174
1237
1124
247
2112
1380
1549
1088
2021
Leaf Width
Degree Days
0
10
20
30
40
50
0
200
400
600
800
1000
1200

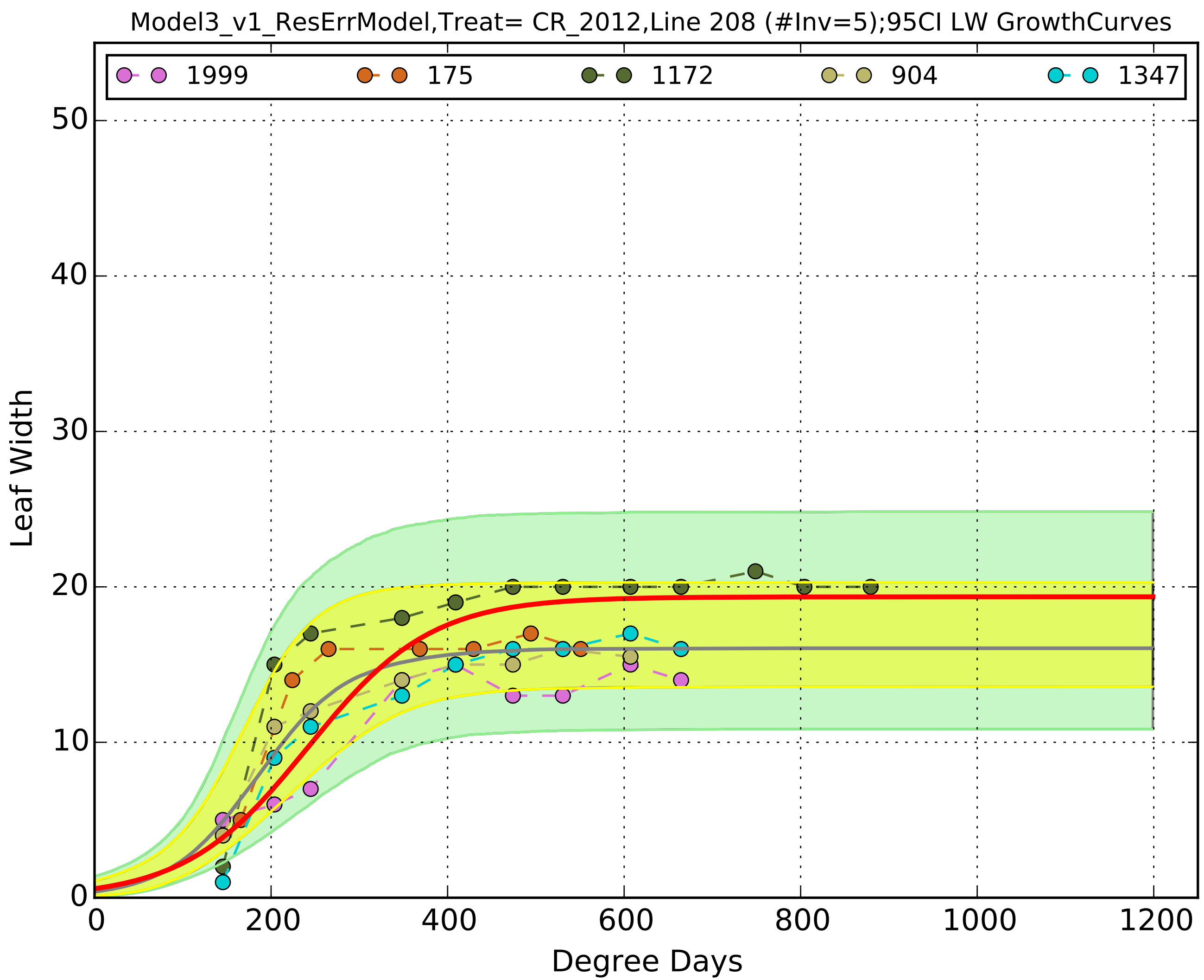
Model3_v1_ResErrModel,Treat= CR_2012,Line 208 (#Inv=5);95CI LW GrowthCurves
1999
175
1172
904
1347
Leaf Width
Degree Days
0
10
20
30
40
50
0
200
400
600
800
1000
1200

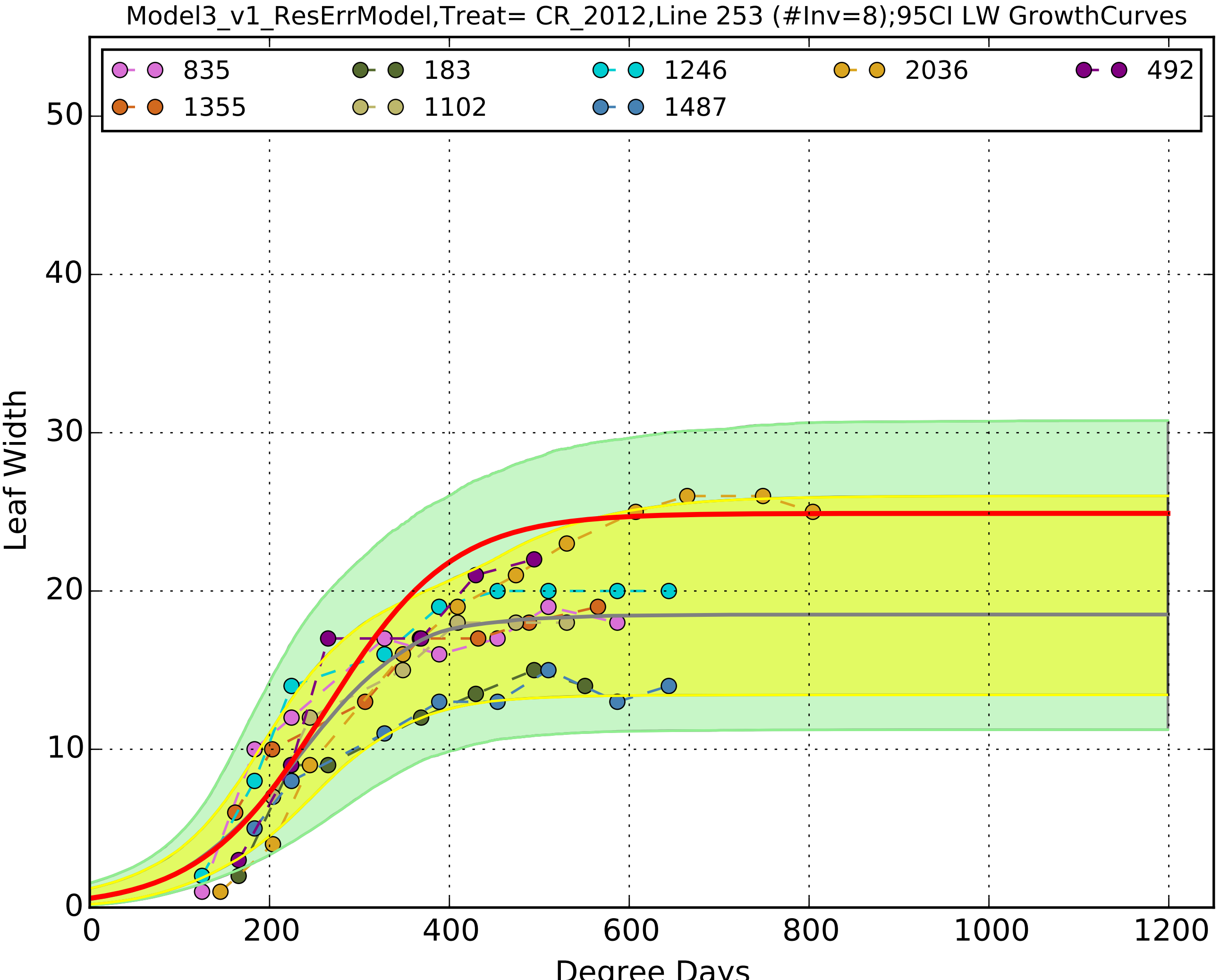

Model3_v1_ResErrModel,Treat= CR_2012,Line 253 (#Inv=8);95CI LW GrowthCurves
835
183
1246
2036
492
1355
1102
1487
Leaf Width
Degree Days
0
10
20
30
40
50
0
200
400
600
800
1000
1200

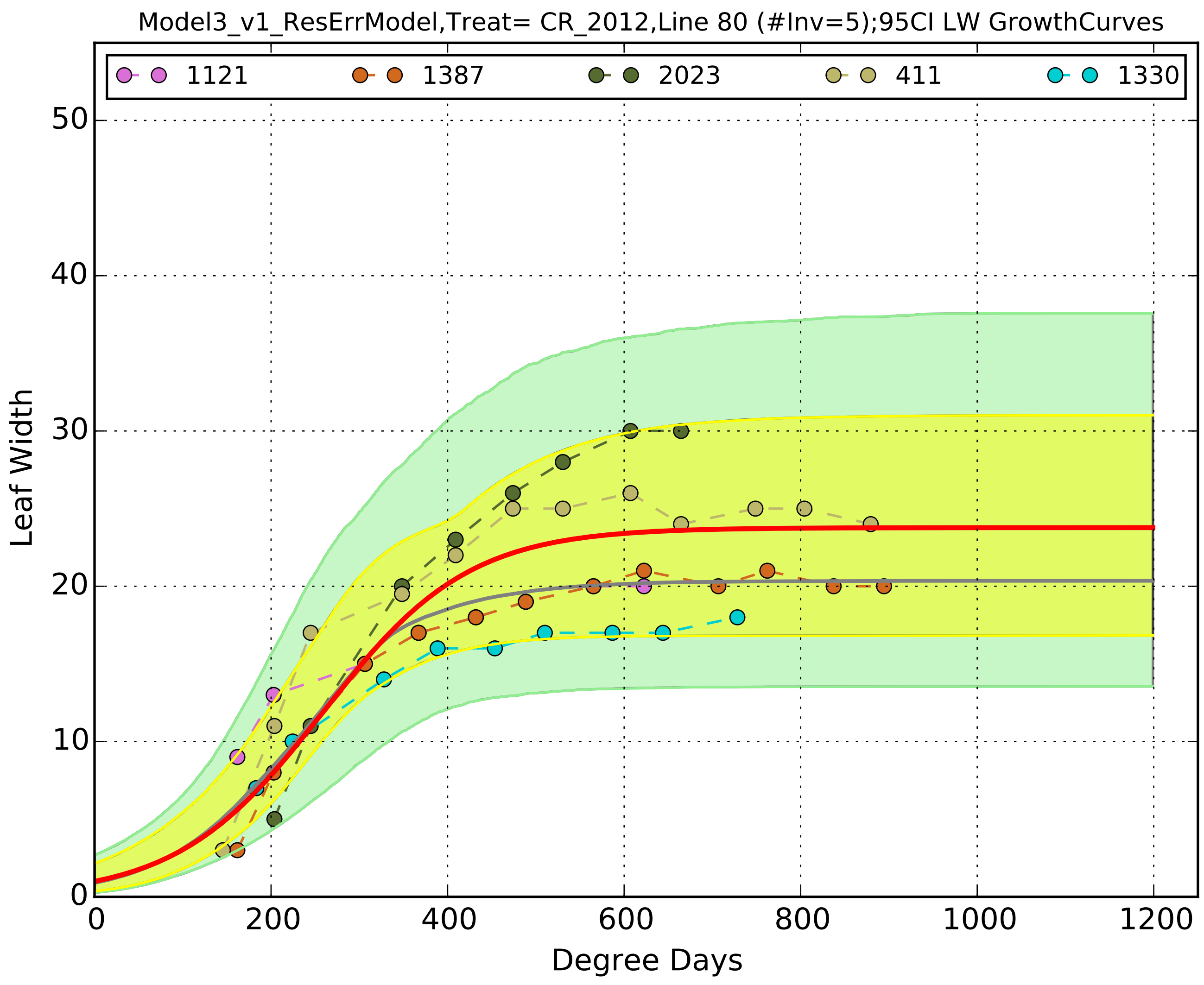

Model3_v1_ResErrModel,Treat= CR_2012,Line 80 (#Inv=5);95CI LW GrowthCurves
1121
1387
2023
411
1330
Leaf Width
Degree Days
0
10
20
30
40
50
0
200
400
600
800
1000
1200

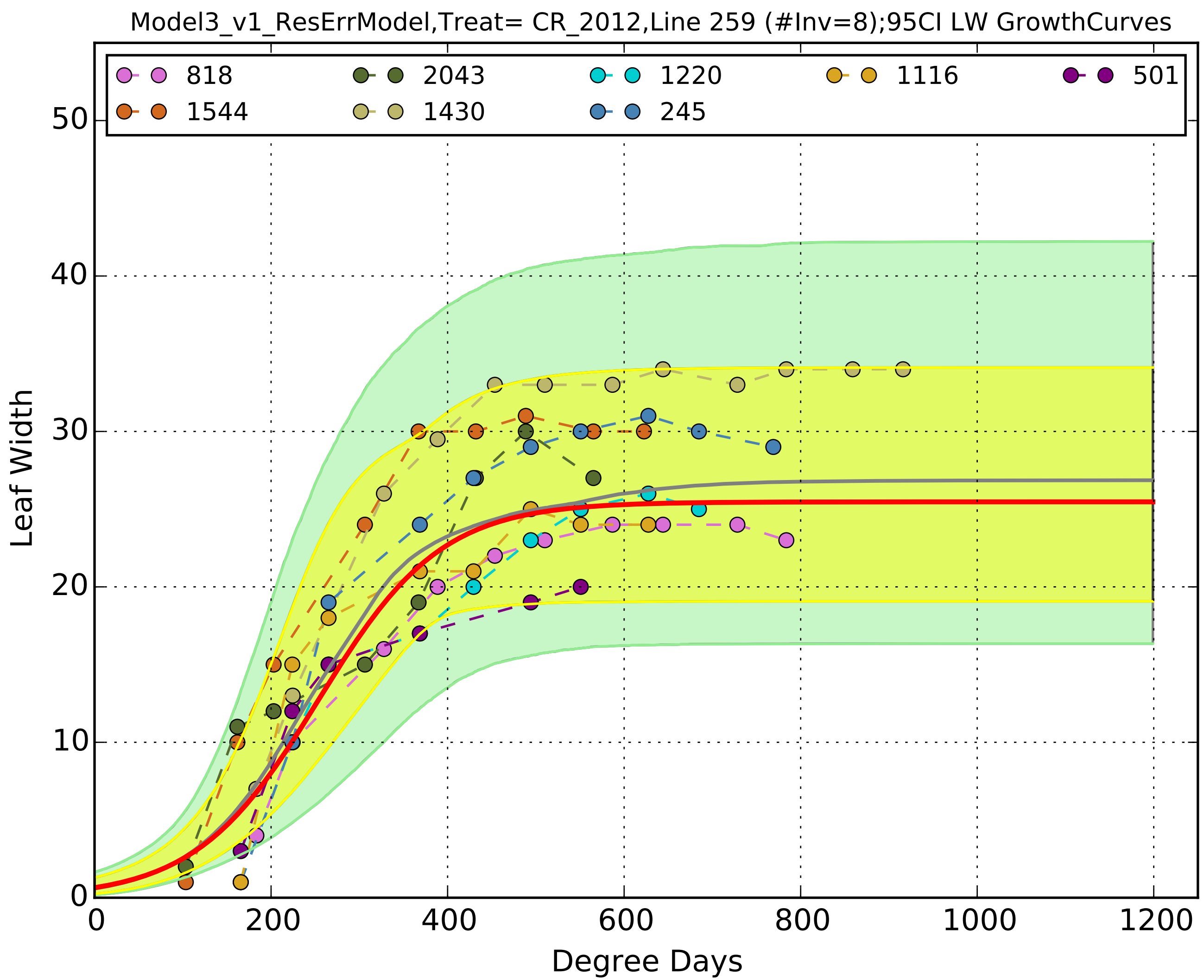
Model3_v1_ResErrModel,Treat= CR_2012,Line 259 (#Inv=8);95CI LW GrowthCurves
818
2043
1220
1116
501
1544
1430
245
Leaf Width
Degree Days
0
10
20
30
40
50
0
200
400
600
800
1000
1200

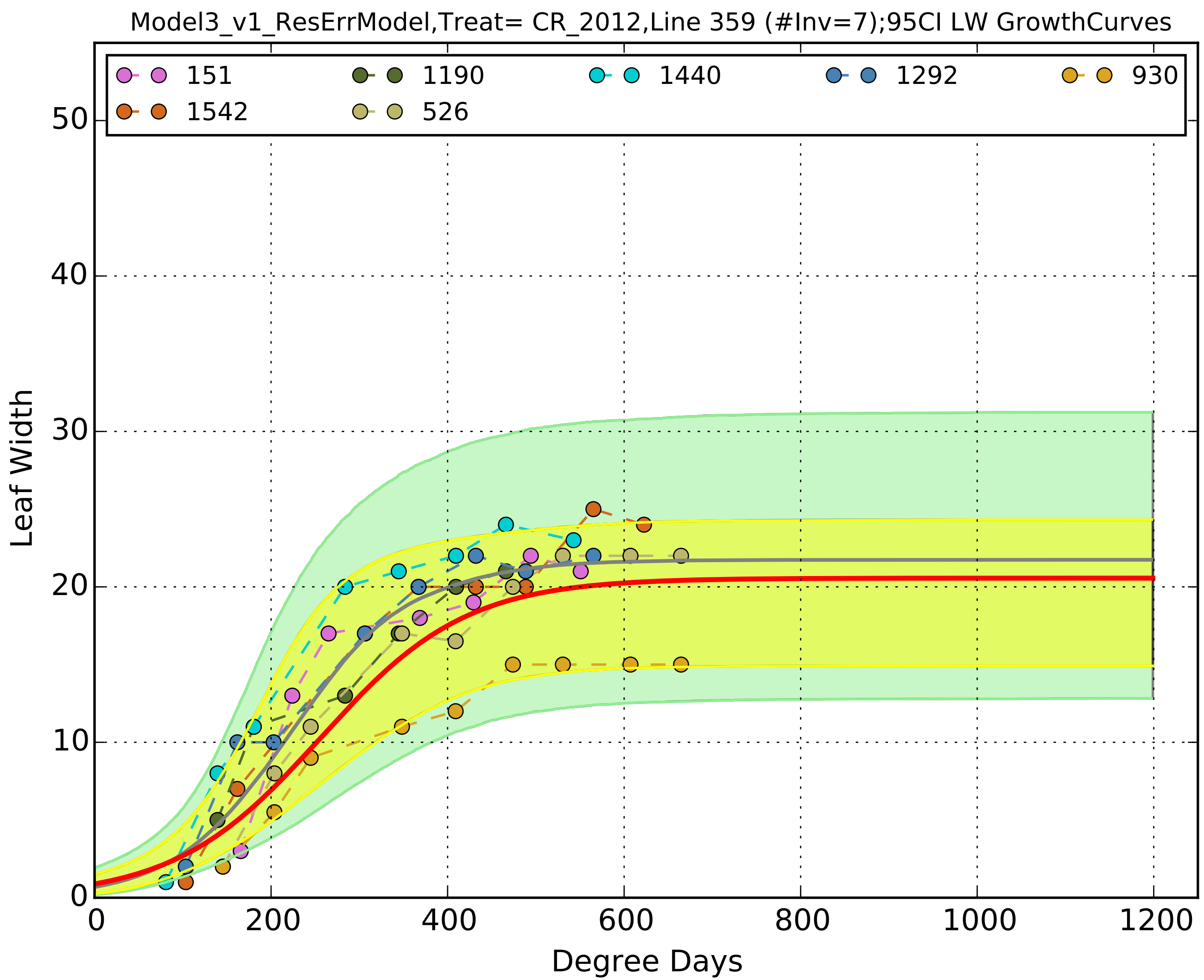
Model3_v1_ResErrModel,Treat= CR_2012,Line 359 (#Inv=7);95CI LW GrowthCurves
151
1190
1440
1292
930
1542
526
Leaf Width
Degree Days
0
10
20
30
40
50
0
200
400
600
800
1000
1200

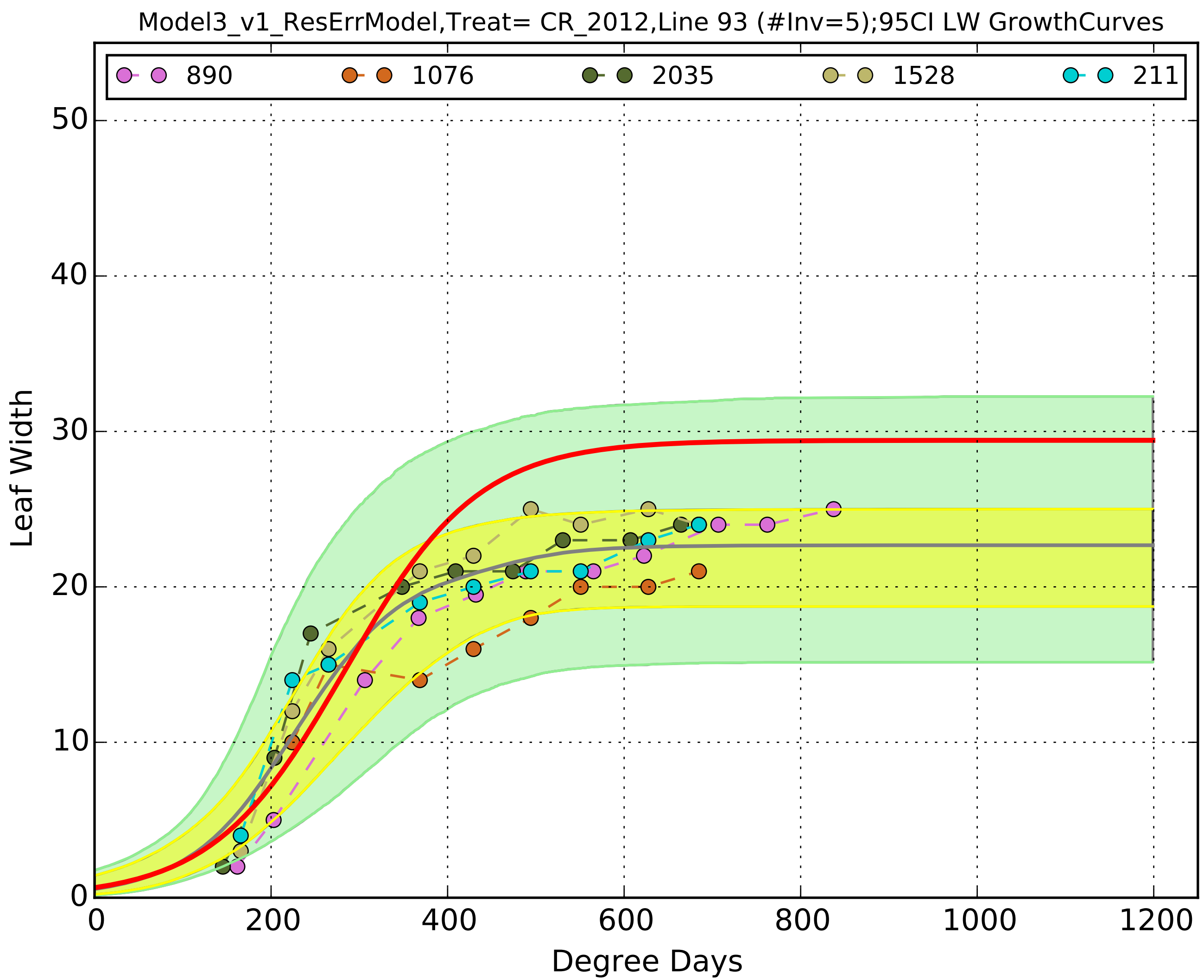
Model3_v1_ResErrModel,Treat= CR_2012,Line 93 (#Inv=5);95CI LW GrowthCurves
890
1076
2035
1528
211
Leaf Width
Degree Days
0
10
20
30
40
50
0
200
400
600
800
1000
1200

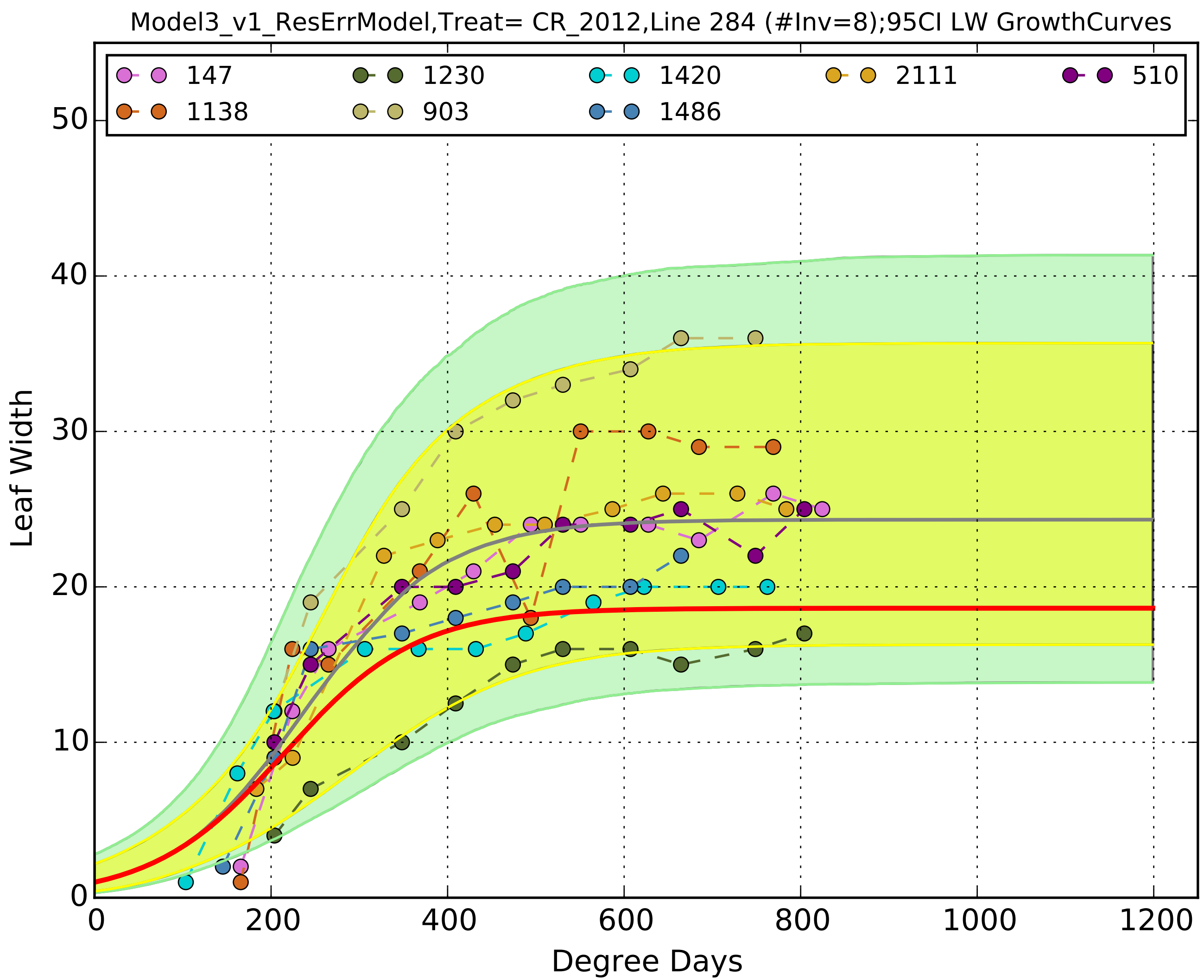

Model3_v1_ResErrModel,Treat= CR_2012,Line 284 (#Inv=8);95CI LW GrowthCurves
147
1230
1420
2111
510
1138
903
1486
Leaf Width
Degree Days
0
10
20
30
40
50
0
200
400
600
800
1000
1200

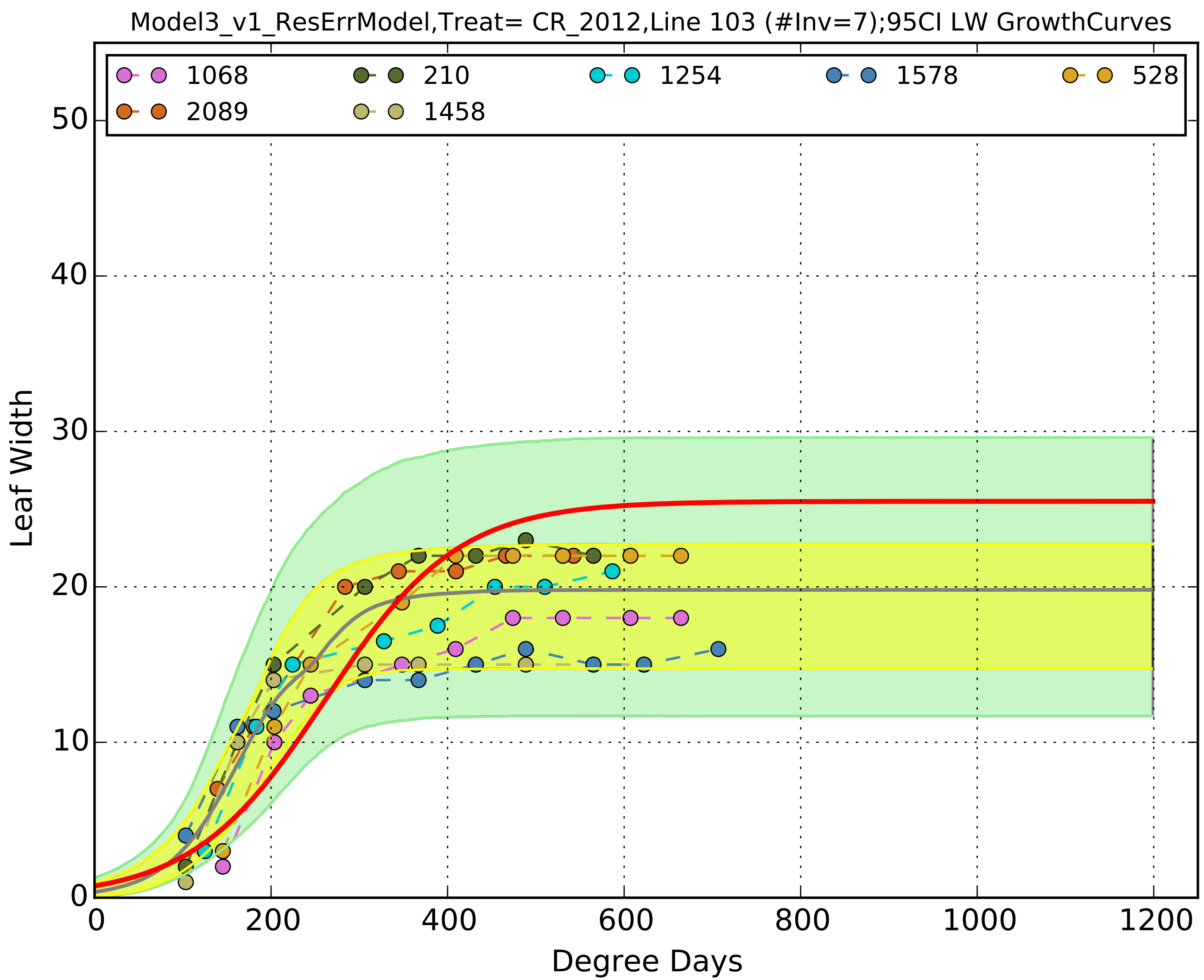
Model3_v1_ResErrModel,Treat= CR_2012,Line 103 (#Inv=7);95CI LW GrowthCurves
1068
210
1254
1578
528
2089
1458
Leaf Width
Degree Days
0
10
20
30
40
50
0
200
400
600
800
1000
1200

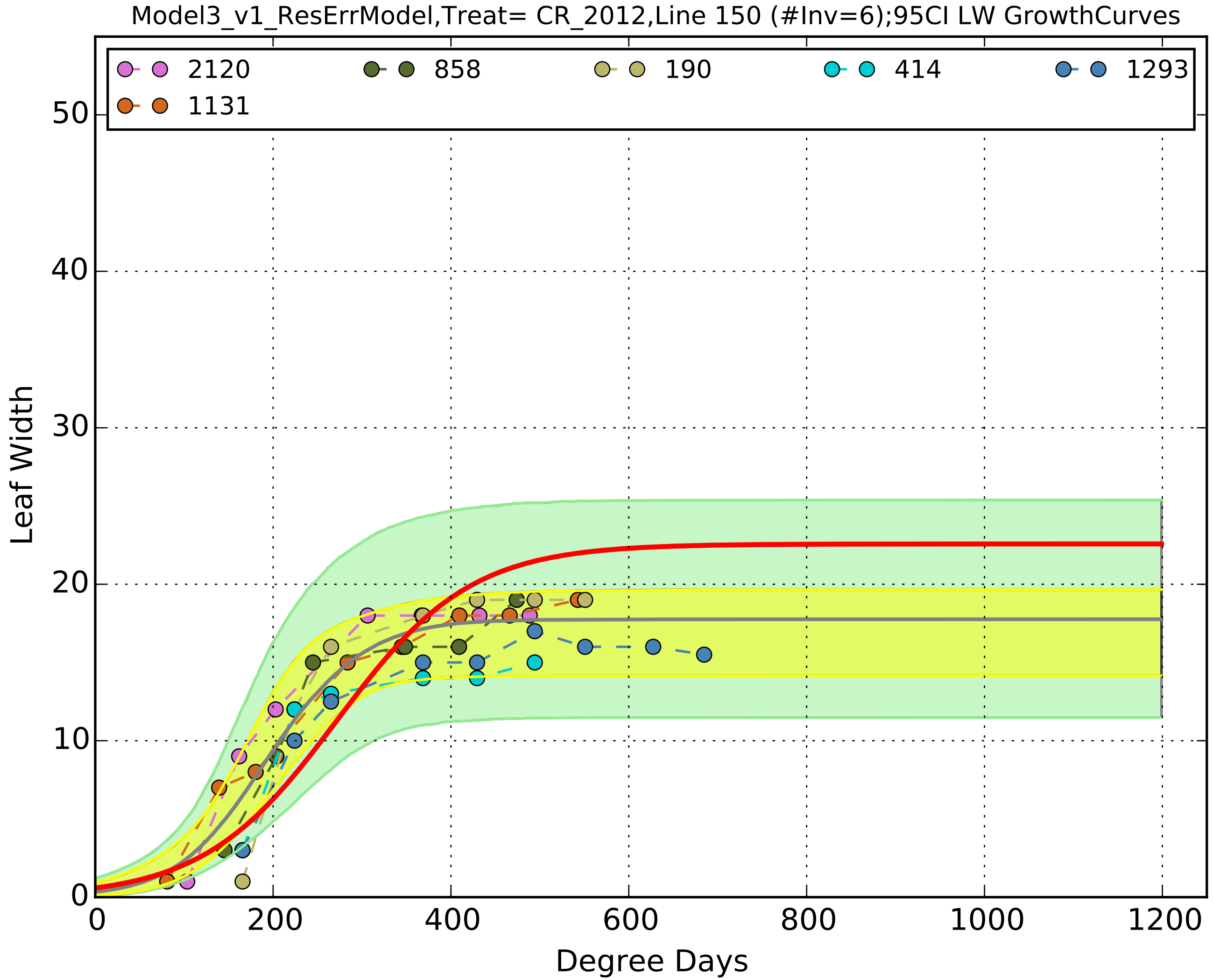
Model3_v1_ResErrModel,Treat= CR_2012,Line 150 (#Inv=6);95CI LW GrowthCurves
2120
858
190
414
1293
1131
Leaf Width
Degree Days
0
10
20
30
40
50
0
200
400
600
800
1000
1200

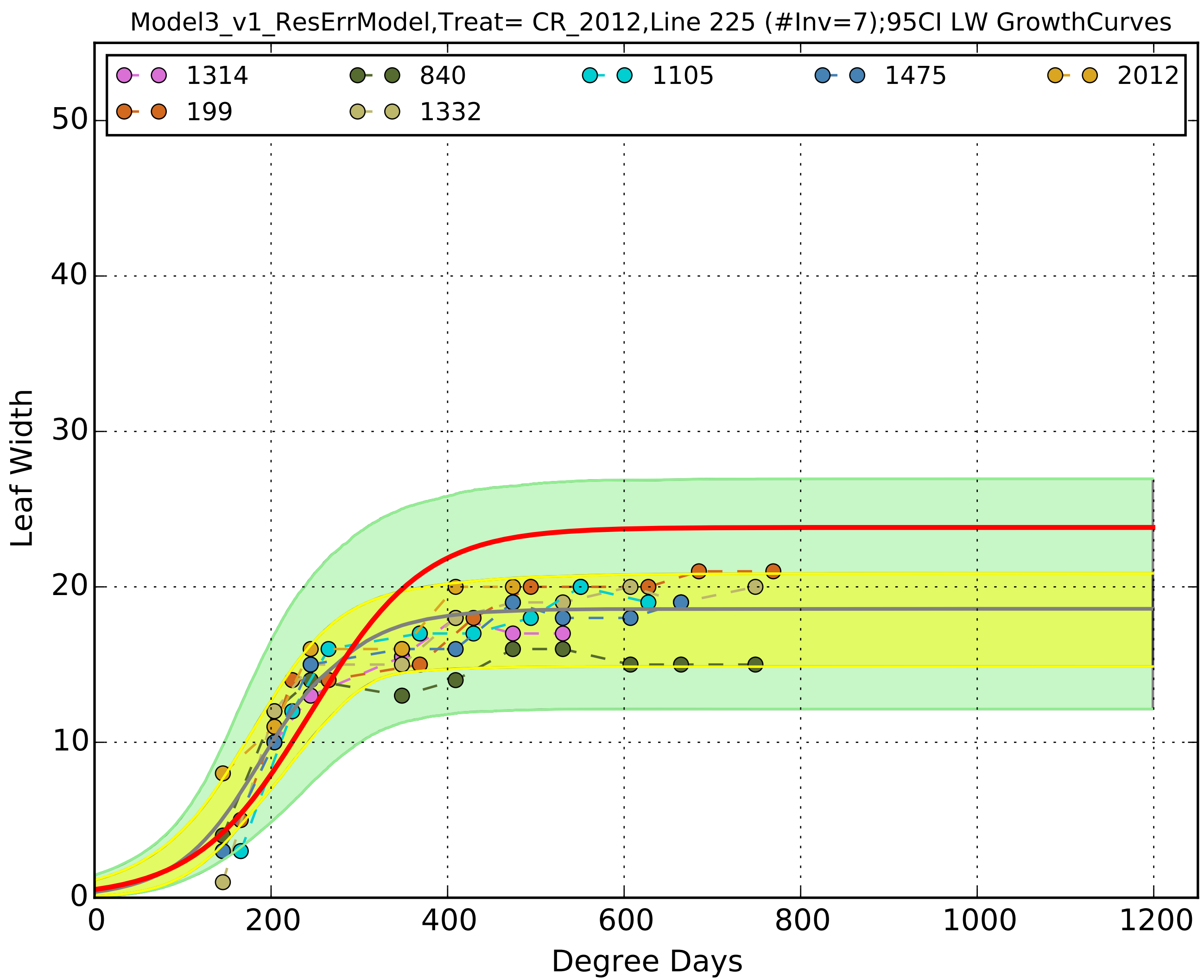

Model3_v1_ResErrModel,Treat= CR_2012,Line 225 (#Inv=7);95CI LW GrowthCurves
1314
840
1105
1475
2012
199
1332
Leaf Width
Degree Days
0
10
20
30
40
50
0
200
400
600
800
1000
1200

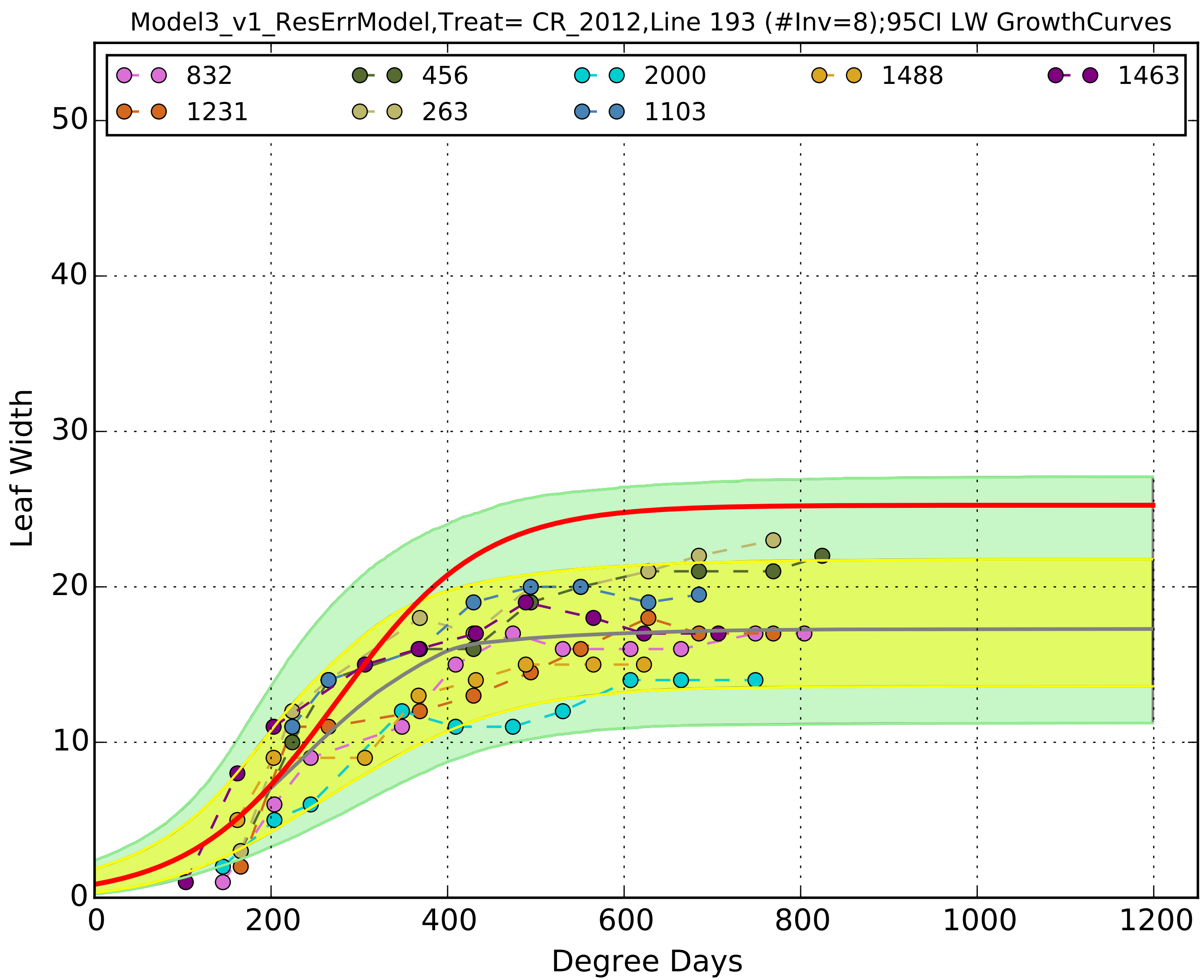

Model3_v1_ResErrModel,Treat= CR_2012,Line 193 (#Inv=8);95CI LW GrowthCurves
832
456
2000
1488
1463
1231
263
1103
Leaf Width
Degree Days
0
10
20
30
40
50
0
200
400
600
800
1000
1200

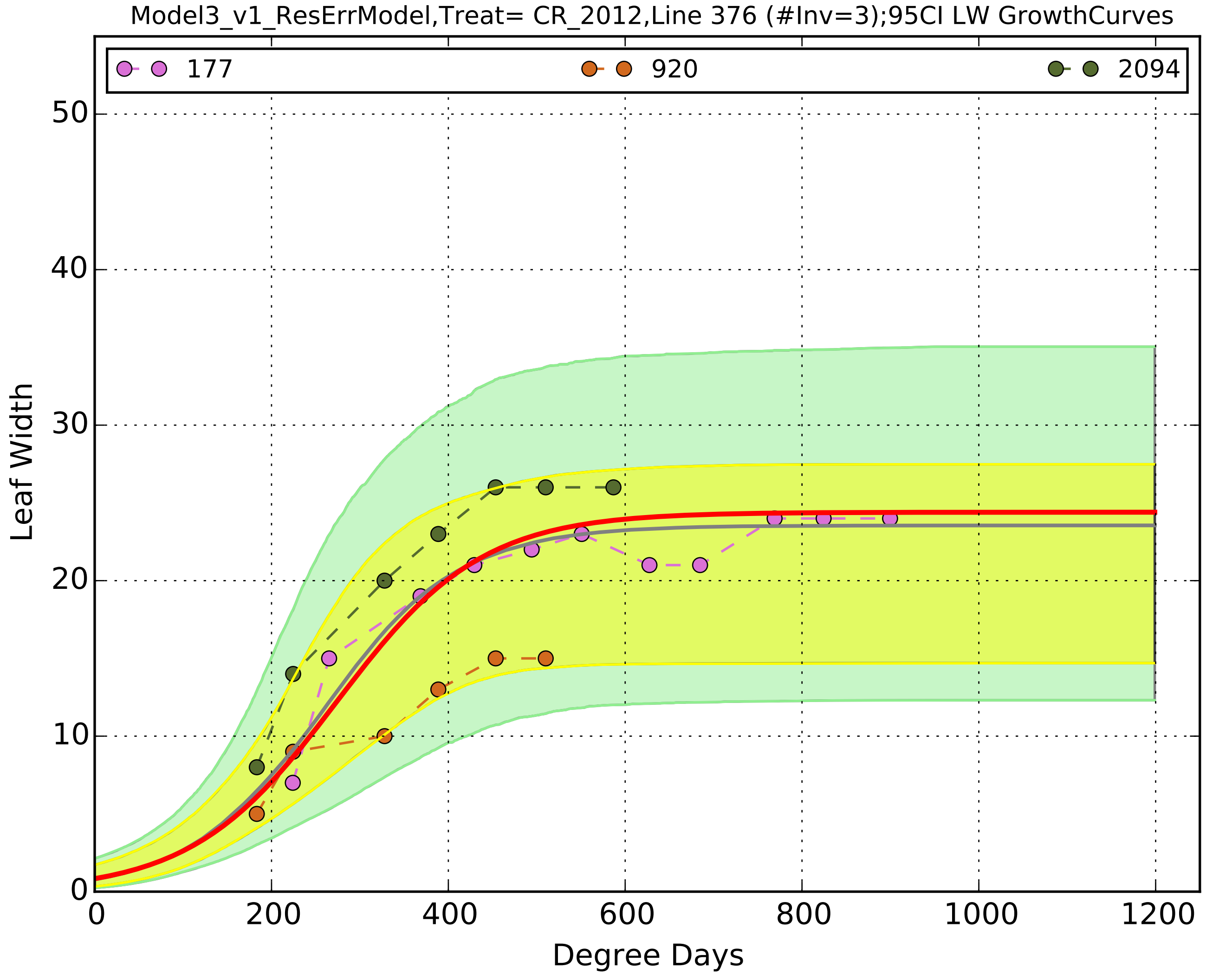

Model3_v1_ResErrModel,Treat= CR_2012,Line 376 (#Inv=3);95CI LW GrowthCurves
177
920
2094
Leaf Width
Degree Days
0
10
20
30
40
50
0
200
400
600
800
1000
1200

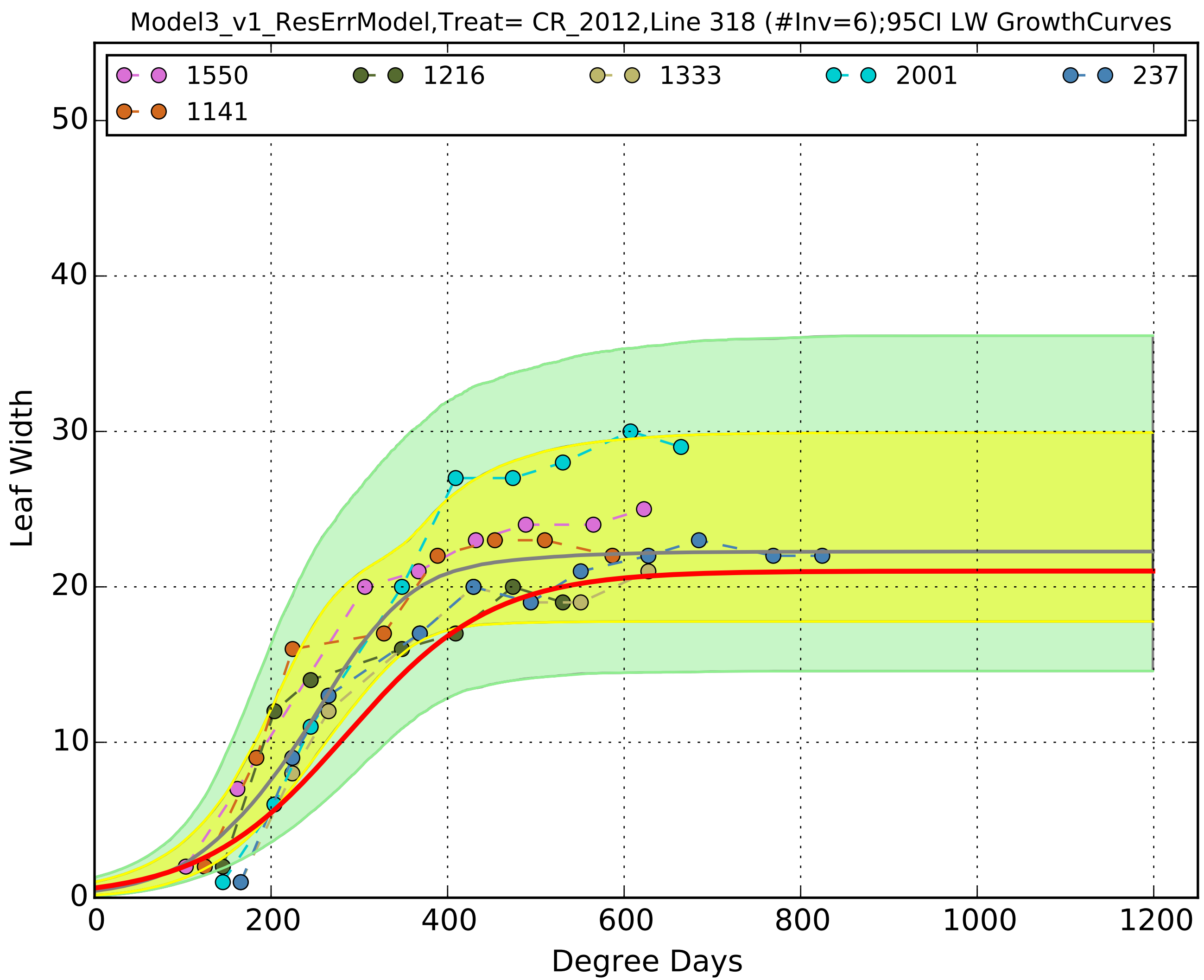
Model3_v1_ResErrModel,Treat= CR_2012,Line 318 (#Inv=6);95CI LW GrowthCurves
1550
1216
1333
2001
237
1141
Leaf Width
Degree Days
0
10
20
30
40
50
0
200
400
600
800
1000
1200

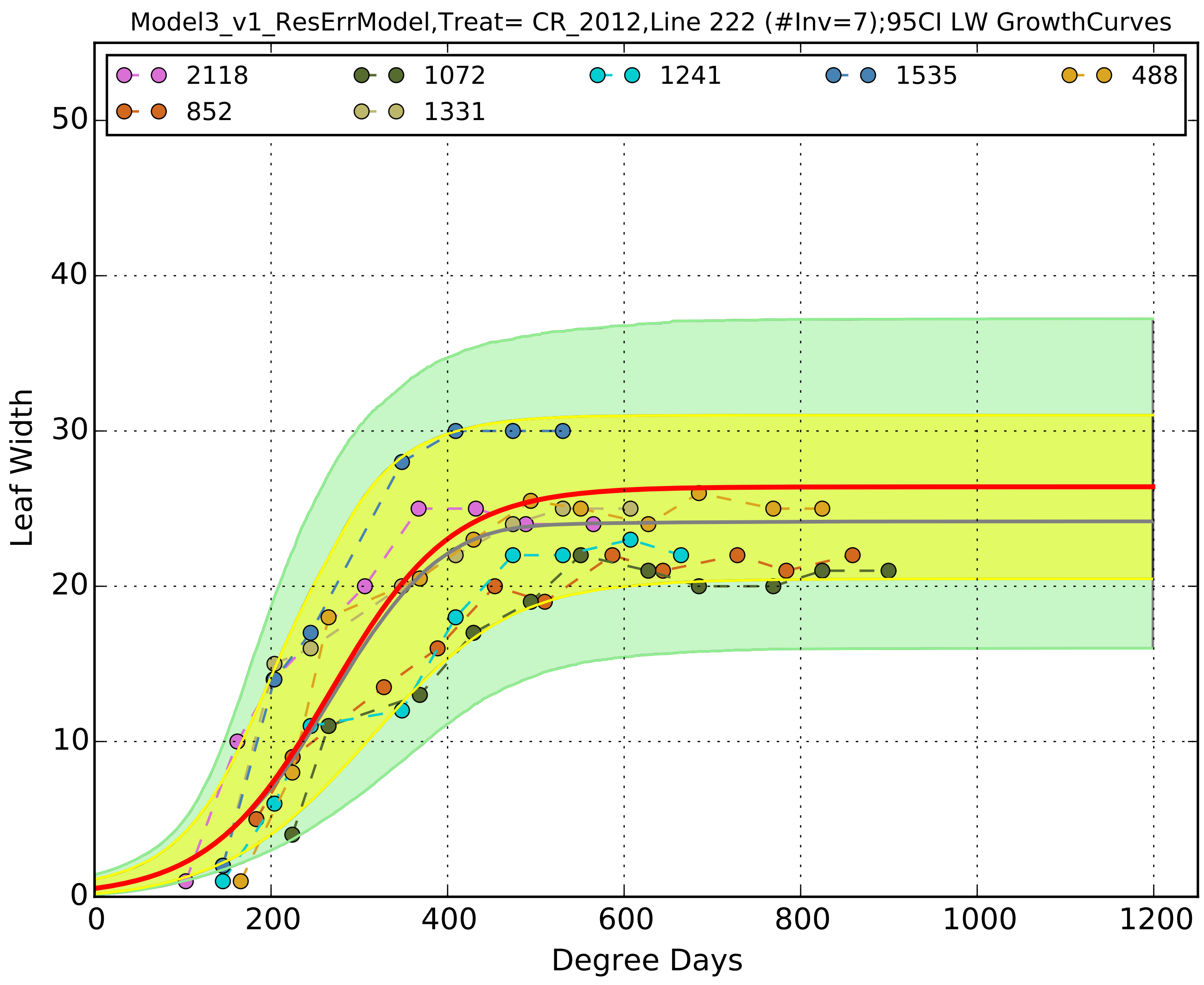

Model3_v1_ResErrModel,Treat= CR_2012,Line 222 (#Inv=7);95CI LW GrowthCurves
2118
1072
1241
1535
488
852
1331
Leaf Width
0
10
20
30
40
50
0
200
400
600
800
1000
1200
Degree Days

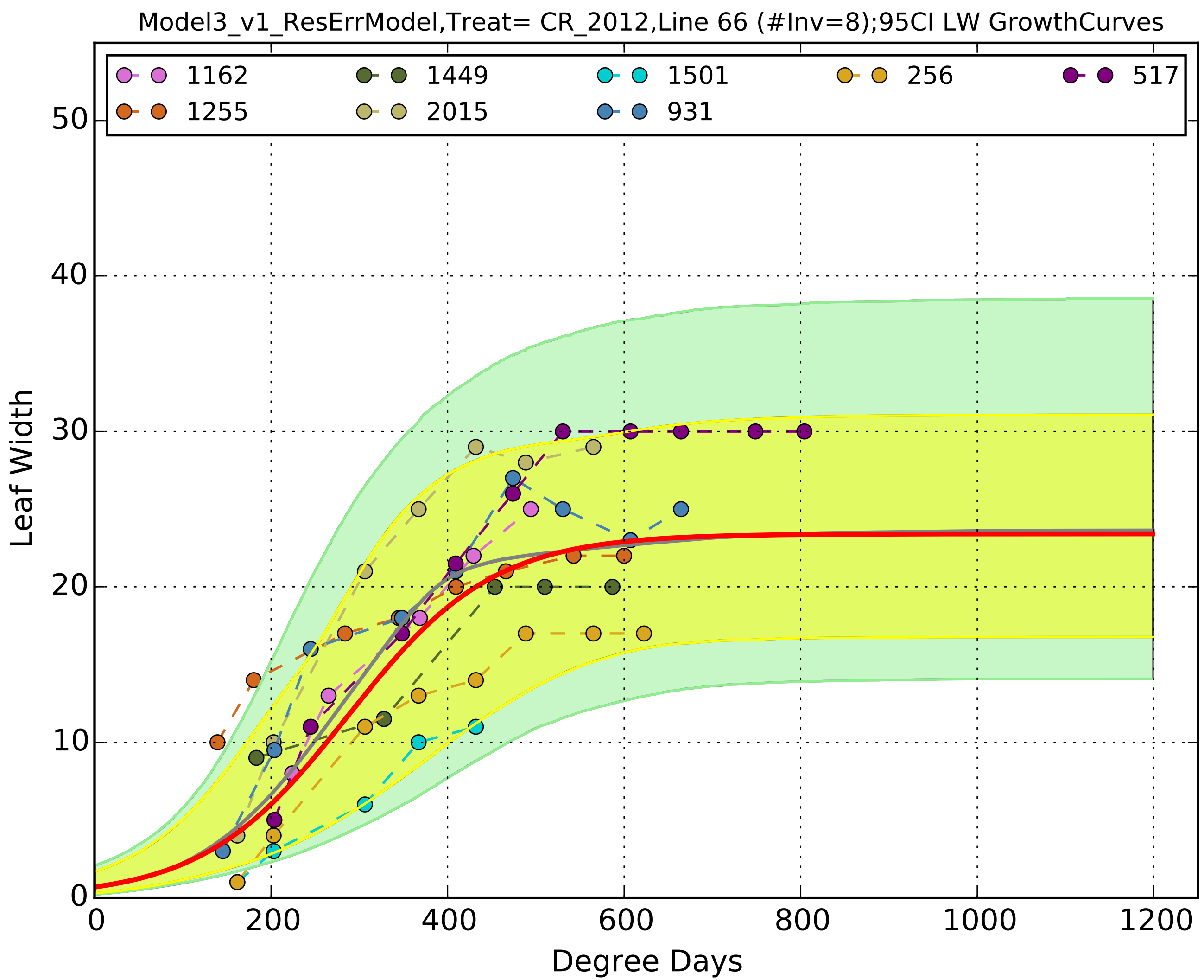

Model3_v1_ResErrModel,Treat= CR_2012,Line 66 (#Inv=8);95CI LW GrowthCurves
1162
1449
1501
256
517
1255
2015
931
Leaf Width
Degree Days
0
10
20
30
40
50
0
200
400
600
800
1000
1200

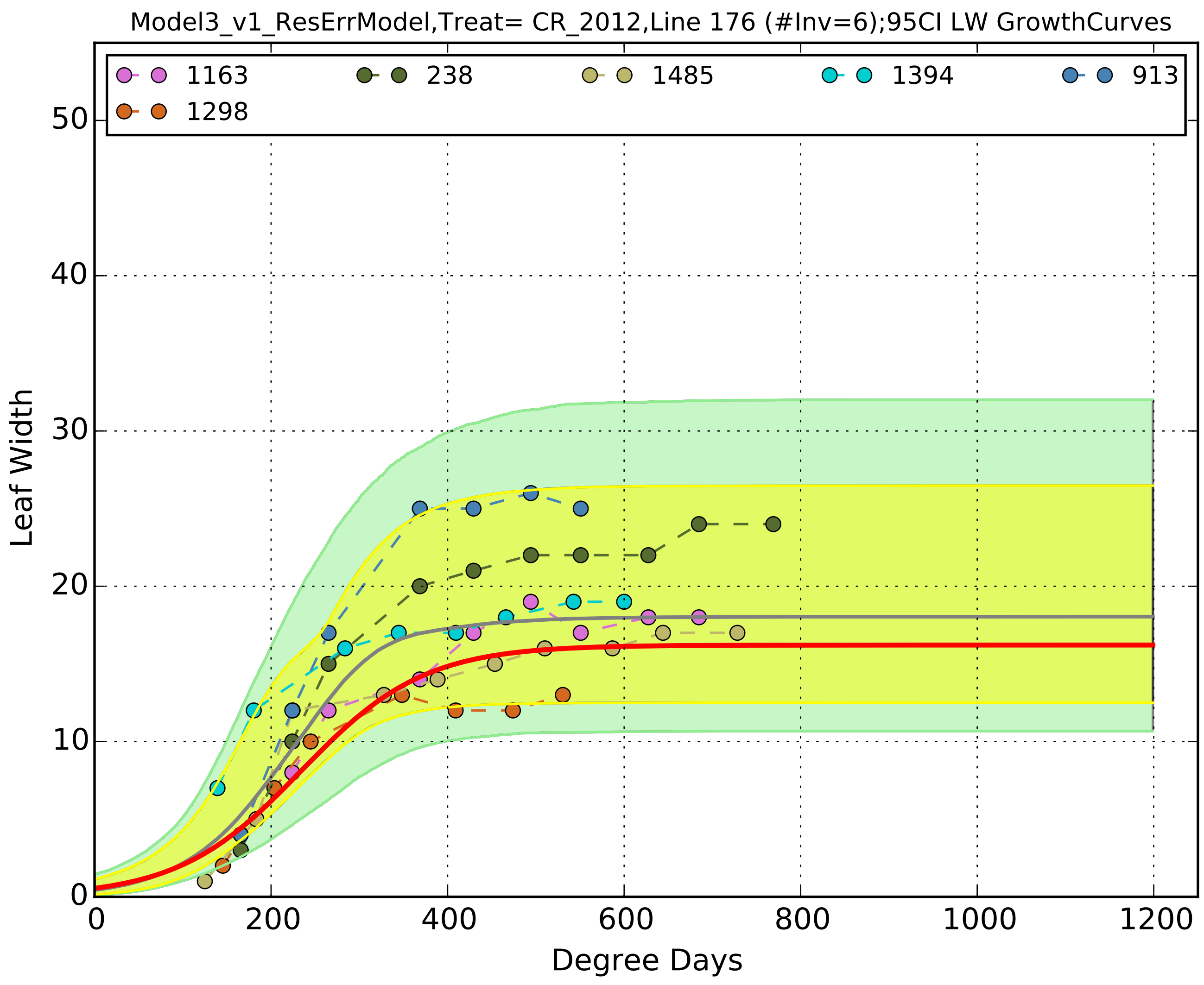
Model3_v1_ResErrModel,Treat= CR_2012,Line 176 (#Inv=6);95CI LW GrowthCurves
1163
238
1485
1394
913
1298
Leaf Width
Degree Days
0
10
20
30
40
50
0
200
400
600
800
1000
1200

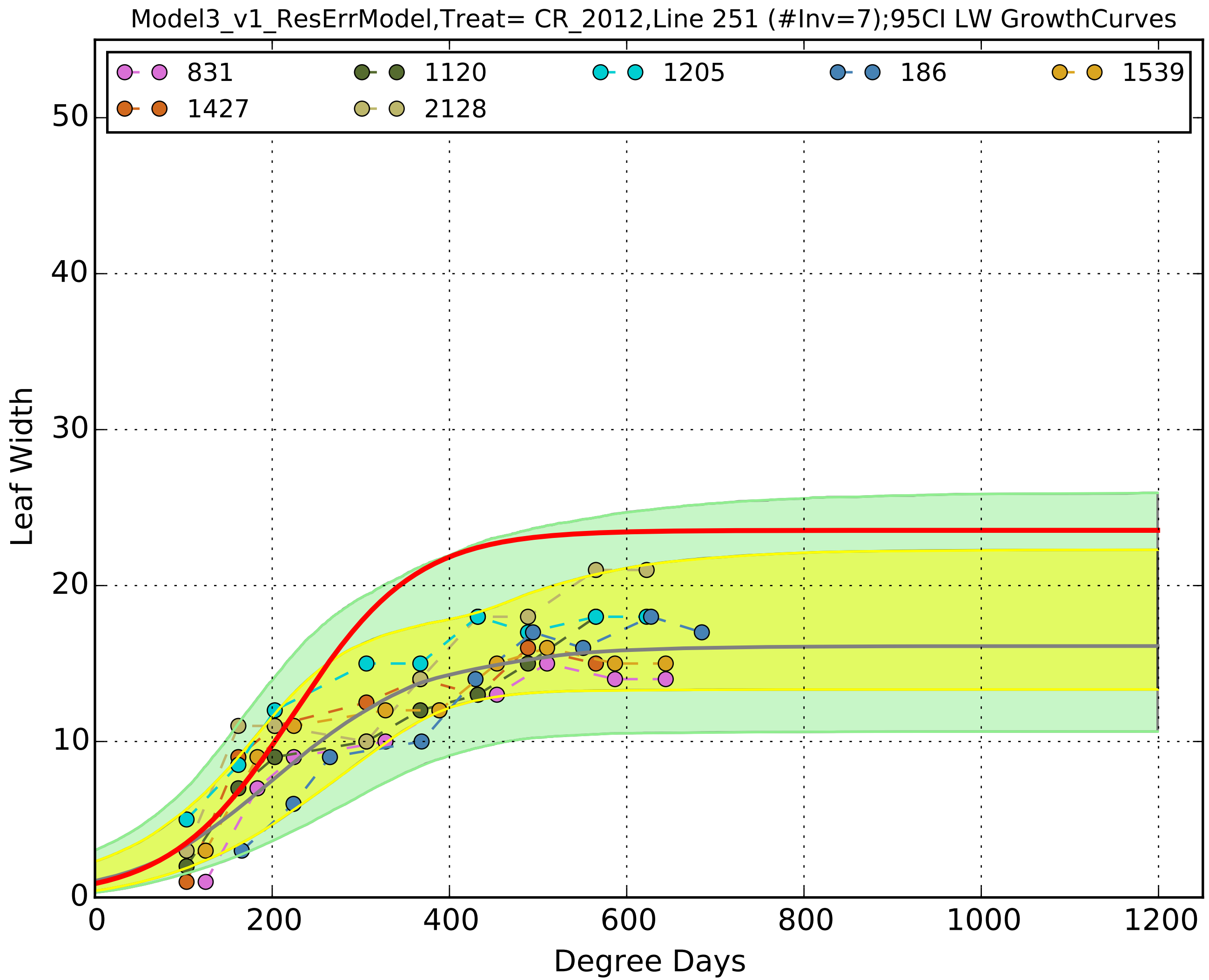
Model3_v1_ResErrModel,Treat= CR_2012,Line 251 (#Inv=7);95CI LW GrowthCurves
831
1120
1205
186
1539
1427
2128
Leaf Width
Degree Days
0
10
20
30
40
50
0
200
400
600
800
1000
1200

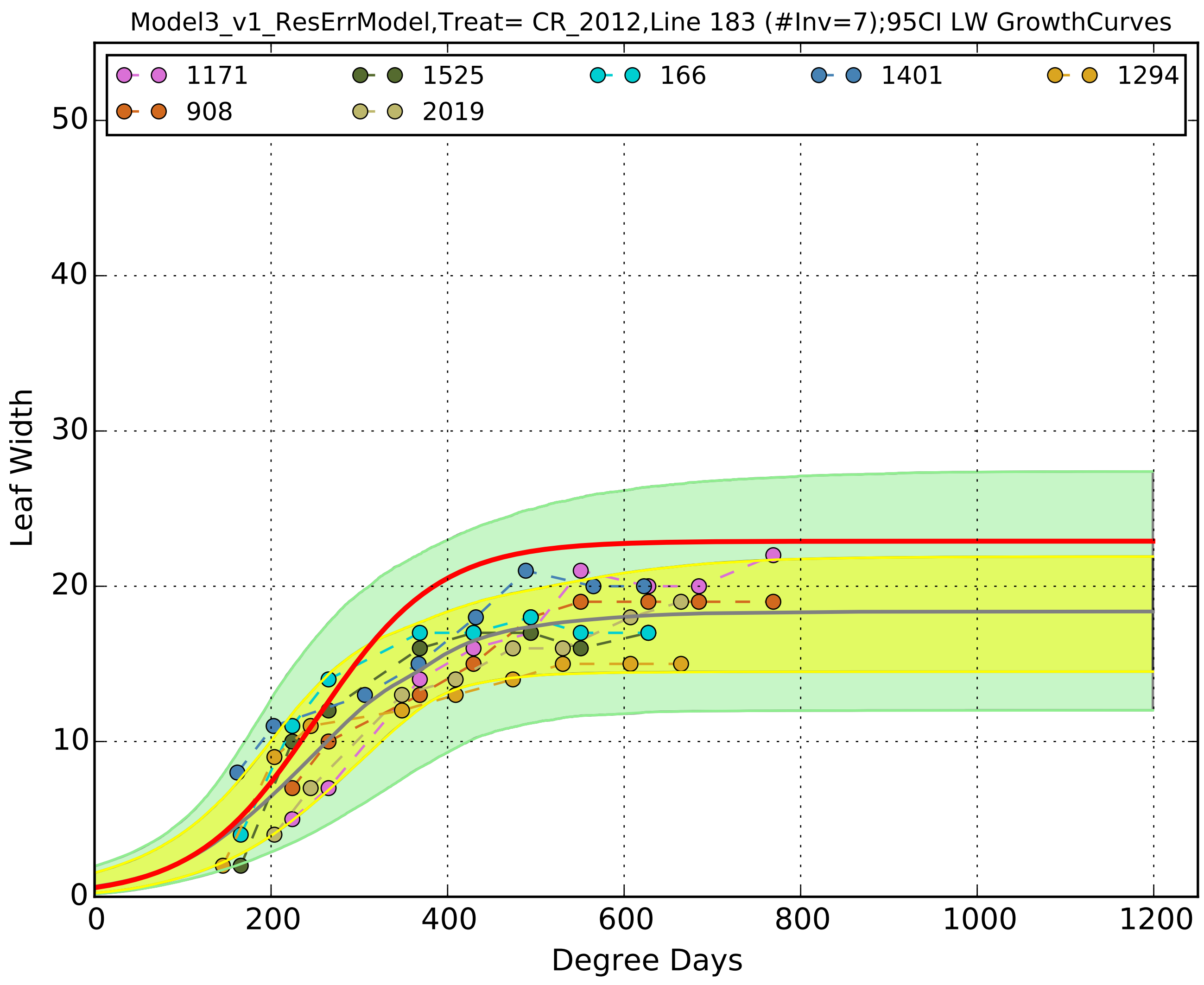

Model3_v1_ResErrModel,Treat= CR_2012,Line 183 (#Inv=7);95CI LW GrowthCurves
1171
1525
166
1401
1294
908
2019
Leaf Width
Degree Days
0
10
20
30
40
50
0
200
400
600
800
1000
1200

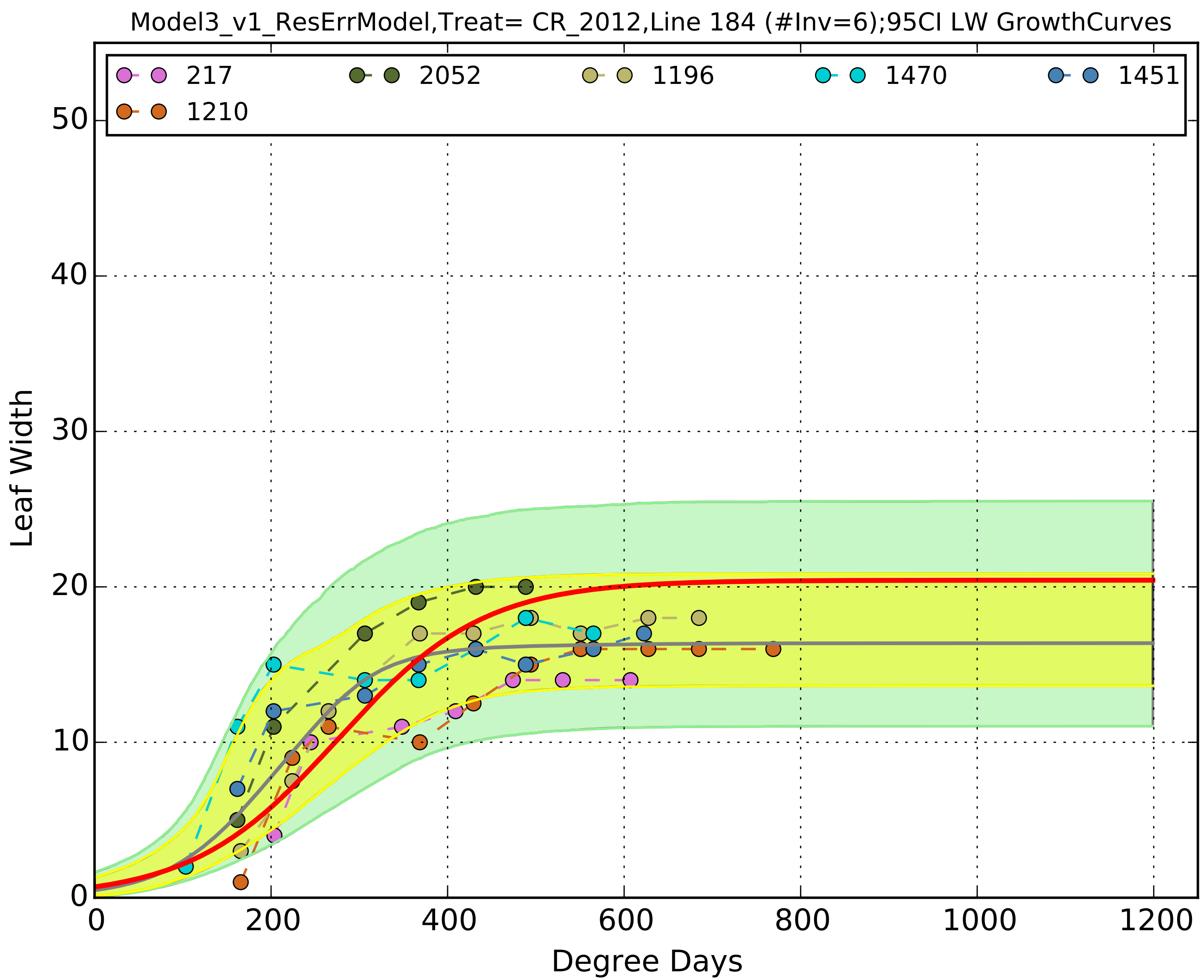

Model3_v1_ResErrModel,Treat= CR_2012,Line 184 (#Inv=6);95CI LW GrowthCurves
217
2052
1196
1470
1451
1210
Leaf Width
Degree Days
0
10
20
30
40
50
0
200
400
600
800
1000
1200

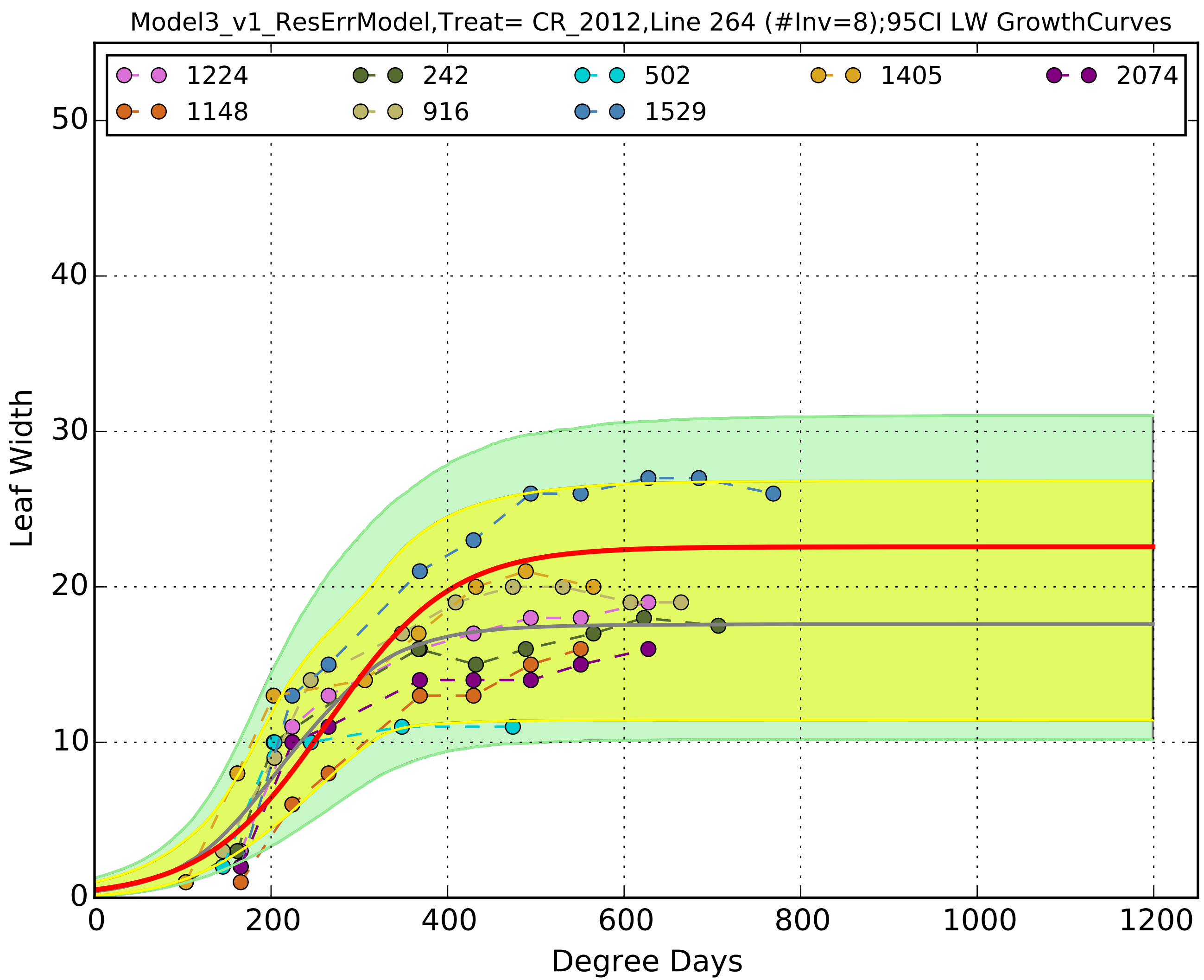

Model3_v1_ResErrModel,Treat= CR_2012,Line 264 (#Inv=8);95CI LW GrowthCurves
1224
242
502
1405
2074
1148
916
1529
Leaf Width
Degree Days
0
10
20
30
40
50
0
200
400
600
800
1000
1200

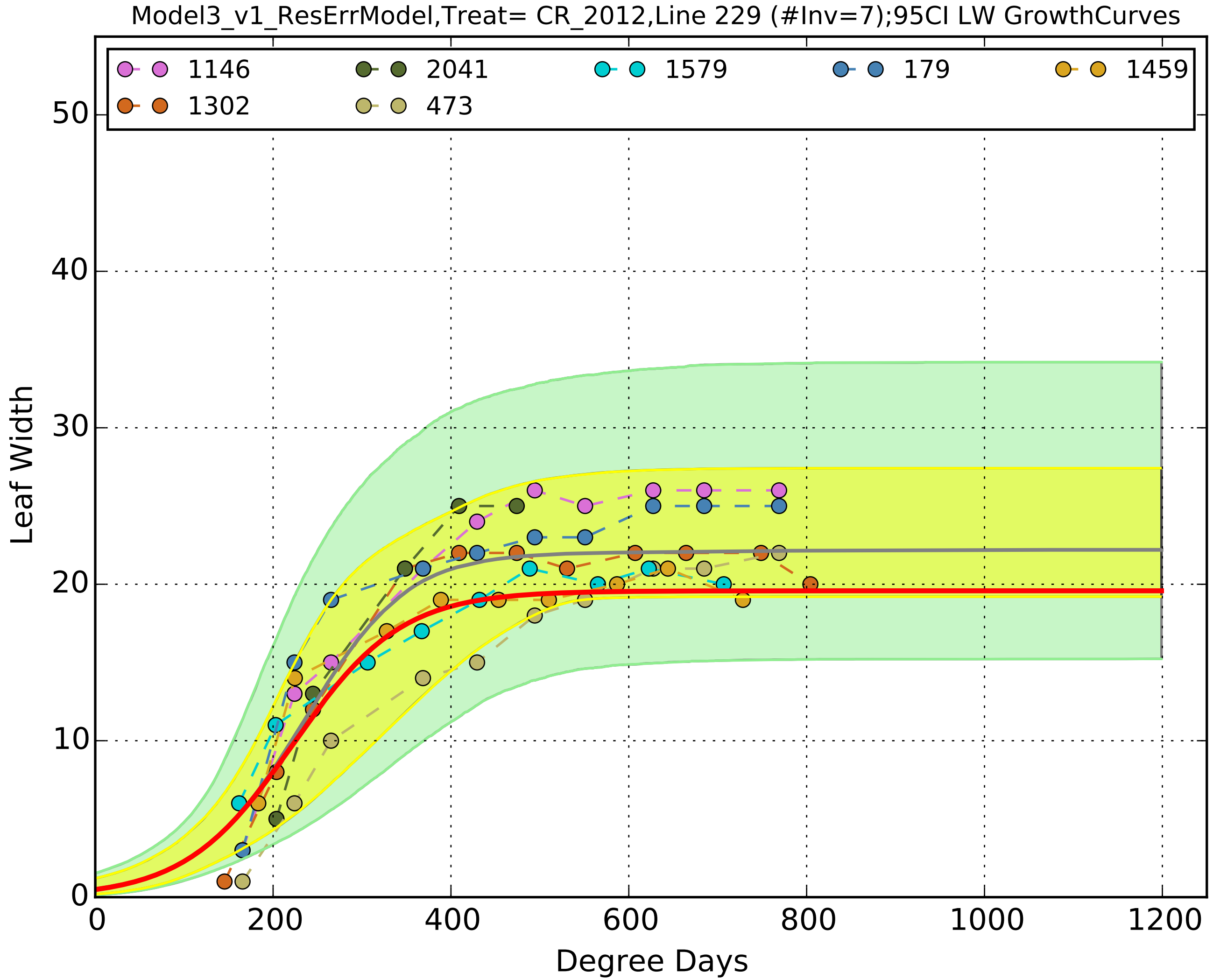

Model3_v1_ResErrModel,Treat= CR_2012,Line 229 (#Inv=7);95CI LW GrowthCurves
1146
2041
1579
179
1459
1302
473
Leaf Width
Degree Days
0
10
20
30
40
50
0
200
400
600
800
1000
1200

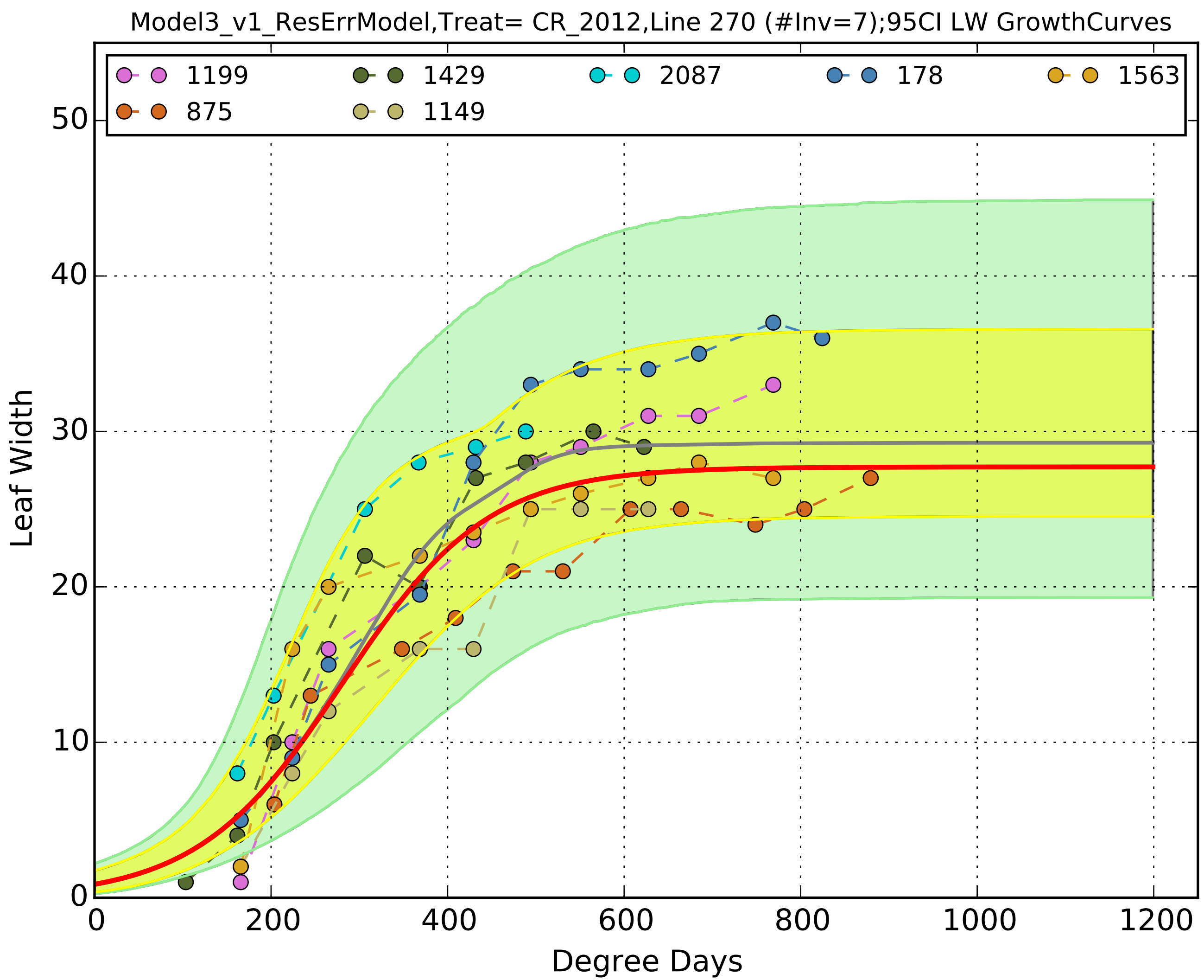

Model3_v1_ResErrModel,Treat= CR_2012,Line 270 (#Inv=7);95CI LW GrowthCurves
1199
1429
2087
178
1563
875
1149
Leaf Width
Degree Days
0
10
20
30
40
50
0
200
400
600
800
1000
1200

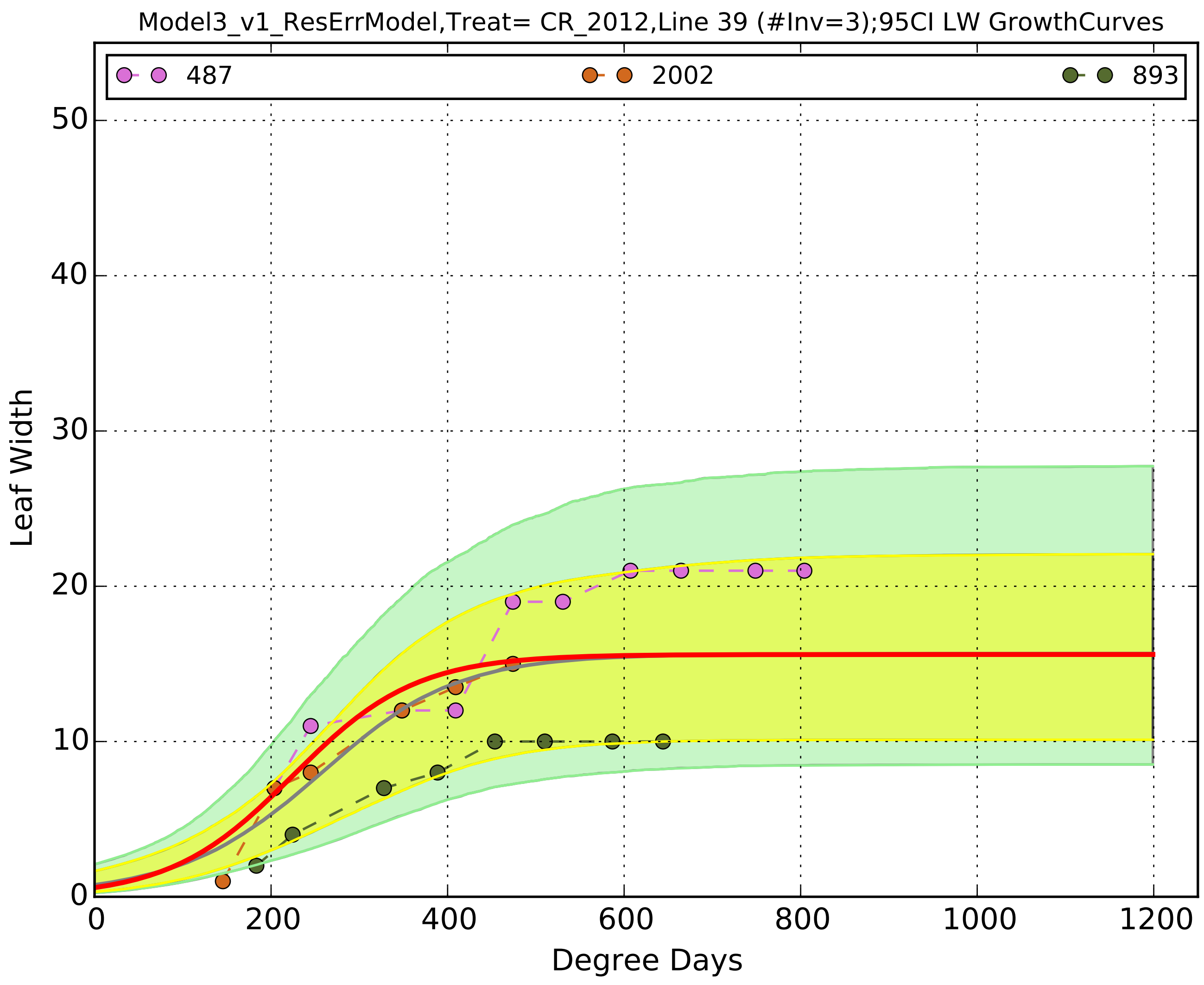

Model3_v1_ResErrModel,Treat= CR_2012,Line 39 (#Inv=3);95CI LW GrowthCurves
487
2002
893
Leaf Width
Degree Days
0
10
20
30
40
50
0
200
400
600
800
1000
1200

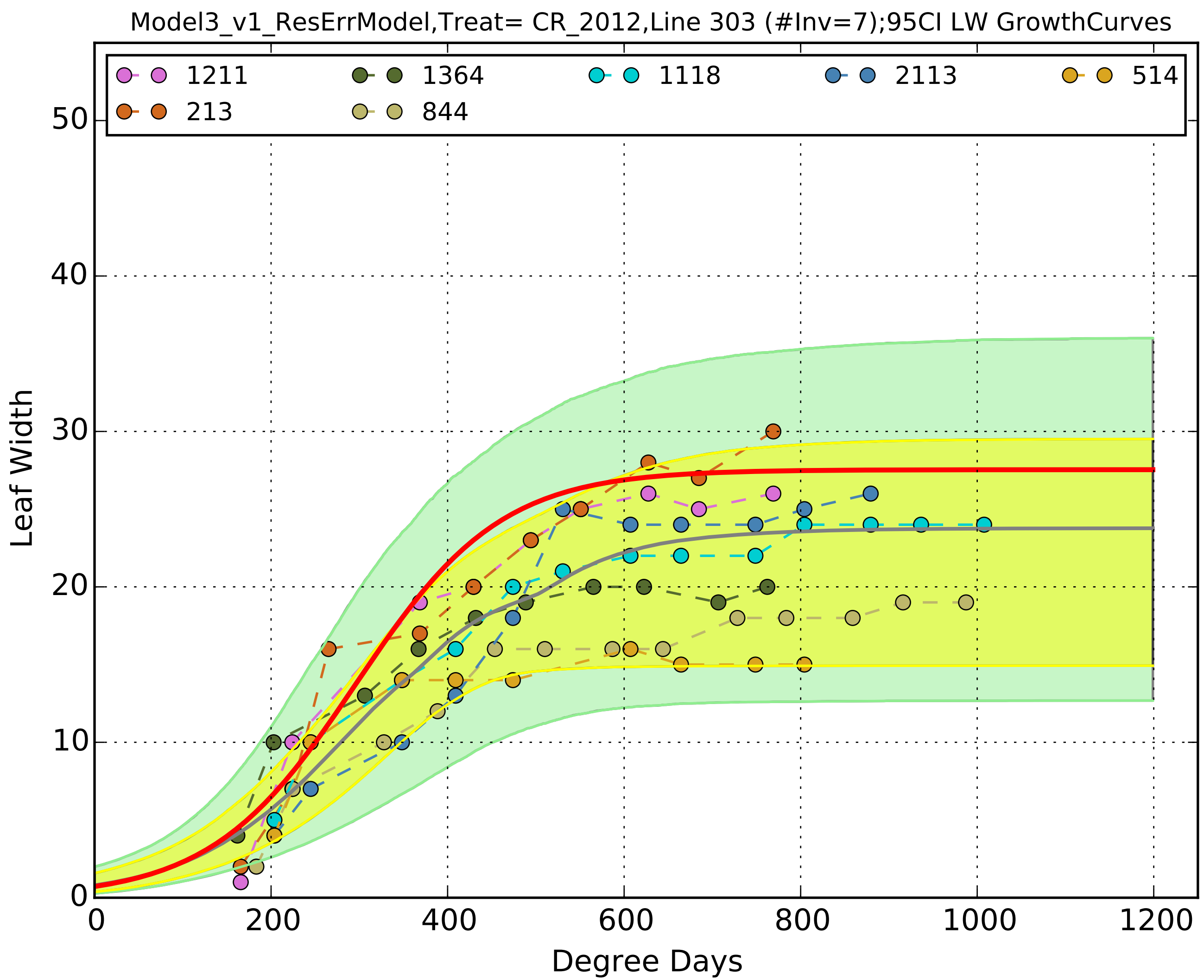
Model3_v1_ResErrModel,Treat= CR_2012,Line 303 (#Inv=7);95CI LW GrowthCurves
1211
1364
1118
2113
514
213
844
Leaf Width
Degree Days
0
10
20
30
40
50
0
200
400
600
800
1000
1200

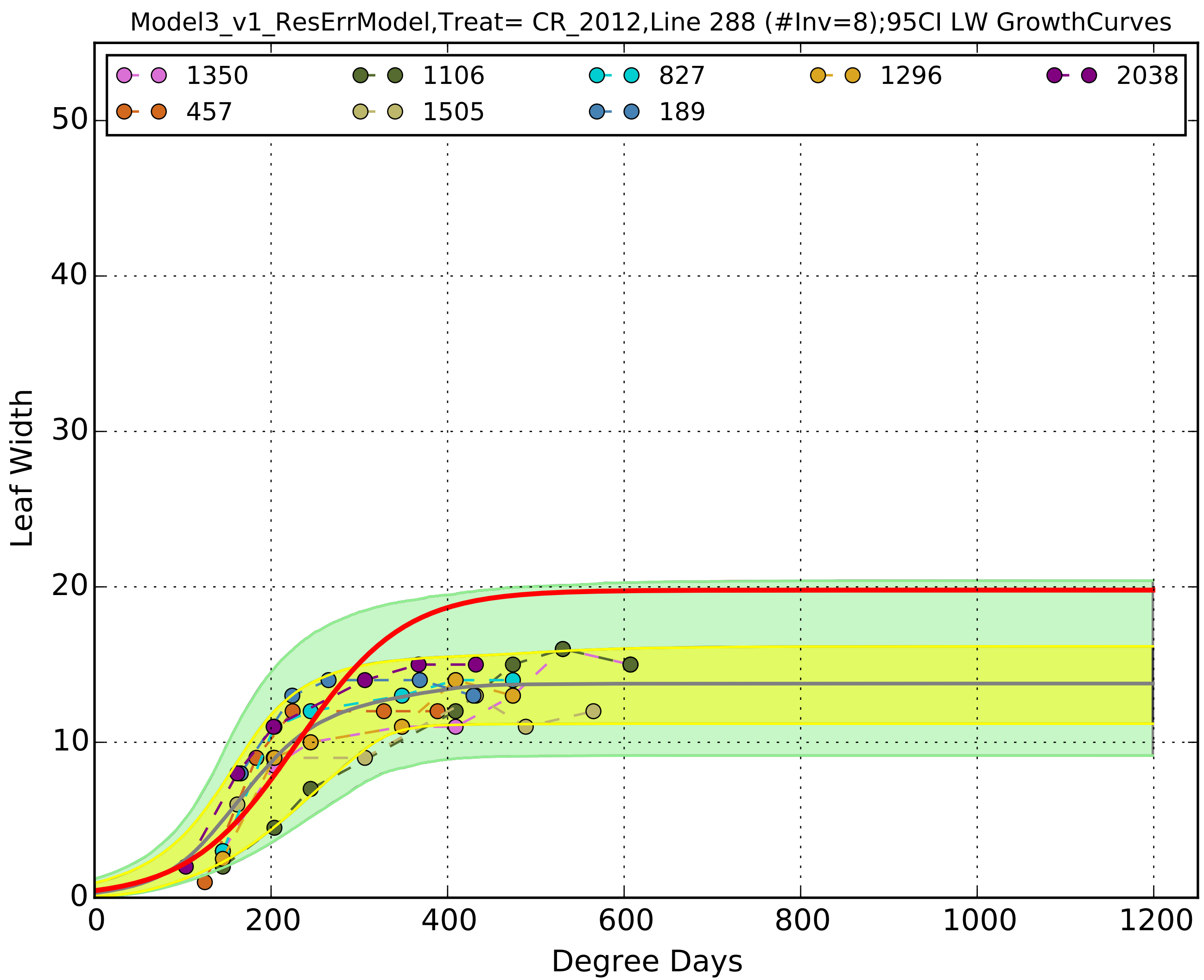
Model3_v1_ResErrModel,Treat= CR_2012,Line 288 (#Inv=8);95CI LW GrowthCurves
1350
1106
827
1296
2038
457
1505
189
Leaf Width
Degree Days
0
10
20
30
40
50
0
200
400
600
800
1000
1200

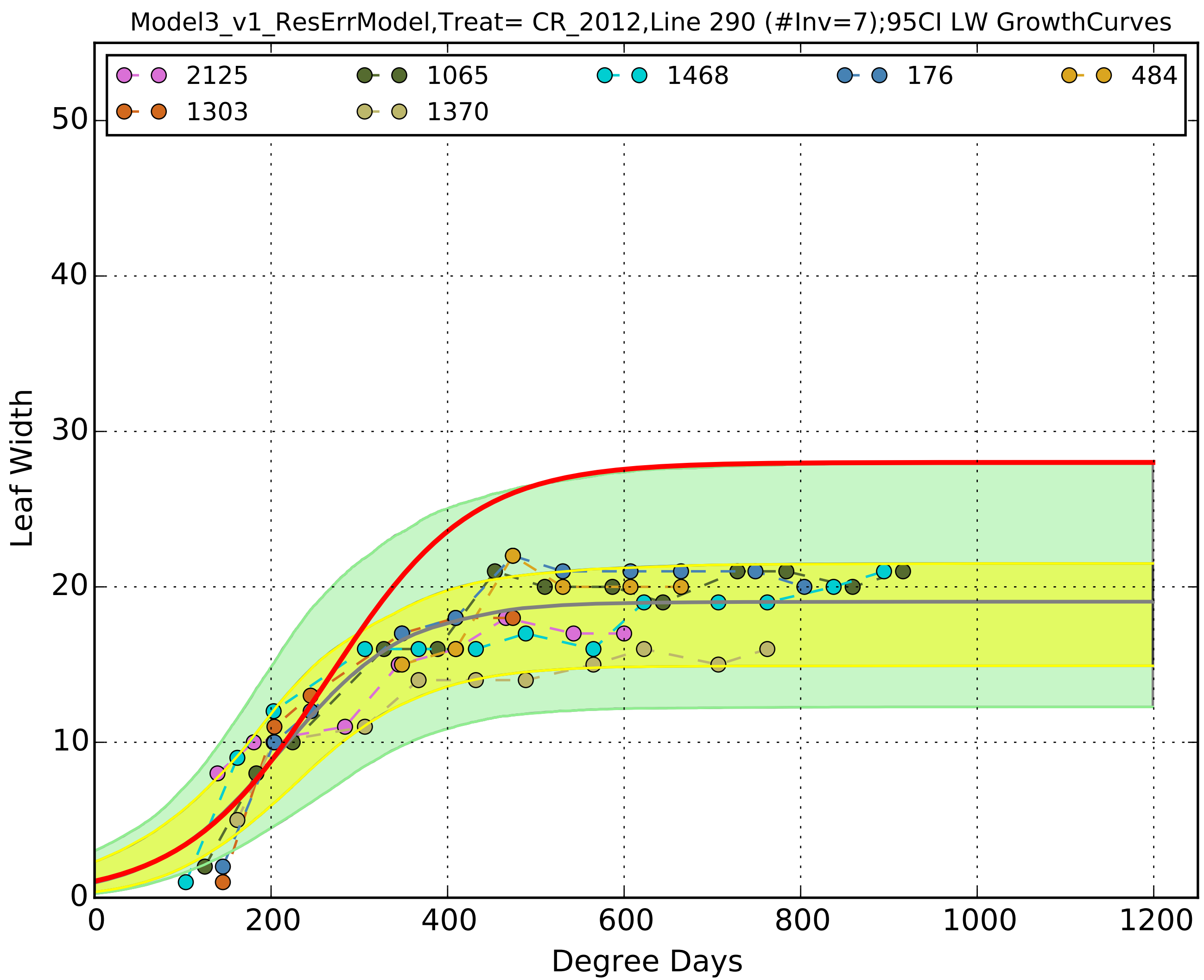

Model3_v1_ResErrModel,Treat= CR_2012,Line 290 (#Inv=7);95CI LW GrowthCurves
2125
1065
1468
176
484
1303
1370
Leaf Width
Degree Days
0
10
20
30
40
50
0
200
400
600
800
1000
1200

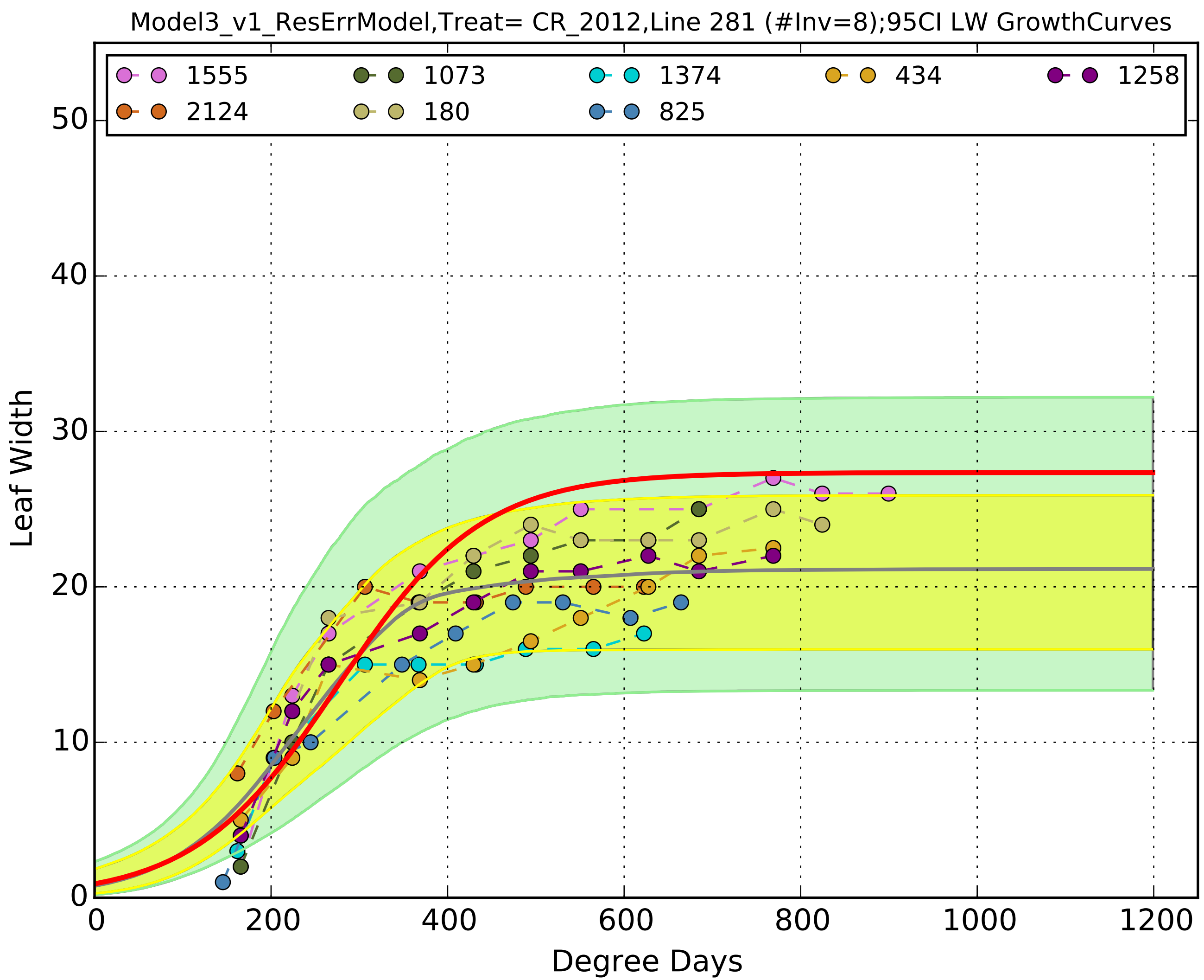
Model3_v1_ResErrModel,Treat= CR_2012,Line 281 (#Inv=8);95CI LW GrowthCurves
1555
1073
1374
434
1258
2124
180
825
Leaf Width
Degree Days
0
10
20
30
40
50
0
200
400
600
800
1000
1200

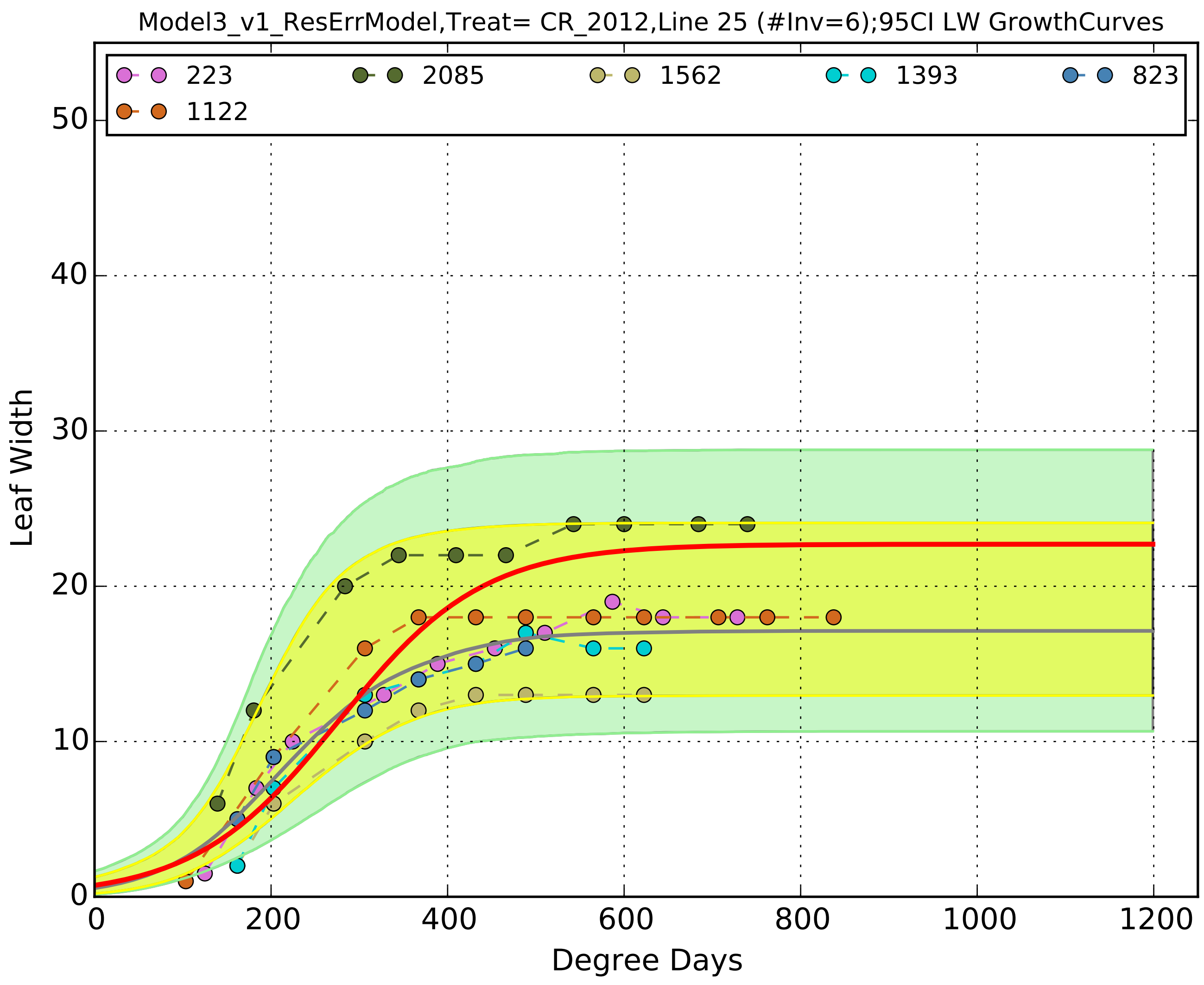
Model3_v1_ResErrModel,Treat= CR_2012,Line 25 (#Inv=6);95CI LW GrowthCurves
223
2085
1562
1393
823
1122
Leaf Width
Degree Days
0
10
20
30
40
50
0
200
400
600
800
1000
1200
